# Metal-Free *S*-Arylation of Phosphorothioate Diesters and Related Compounds with Diaryliodonium Salts

**DOI:** 10.1021/acs.orglett.2c04310

**Published:** 2023-01-20

**Authors:** Sudeep Sarkar, Marcin Kalek

**Affiliations:** † Centre of New Technologies, University of Warsaw, Banacha 2C, 02-097 Warsaw, Poland; ‡ Faculty of Chemistry, University of Warsaw, Pasteura 1, 02-093 Warsaw, Poland

## Abstract

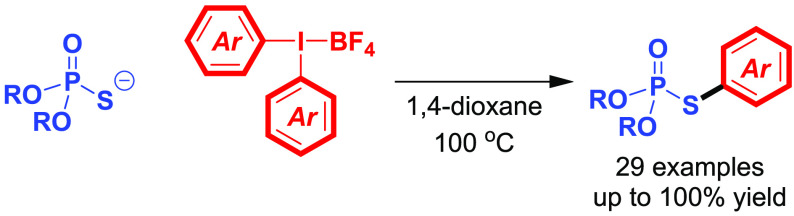

We developed a direct metal-free *S-*arylation of phosphorothioate diesters using diaryliodonium salts. The method allows for the preparation under simple conditions of a broad range of *S-*aryl phosphorothioates, including complex molecules (e.g., dinucleotide or TADDOL derivatives), as well as other related organophosphorus compounds arylated at a chalcogen. The reaction proceeds with a full retention of the stereogenic center at the phosphorus atom, opening convenient access to P-chiral products. The mechanism of the reaction was established using DFT calculations.

Sulfur-containing organophosphorus compounds display an array of interesting and valuable properties from both biological and chemical viewpoints. Accordingly, they have found widespread applications ranging from agrochemicals and pharmaceuticals (including oligonucleotide therapeutics), through building blocks for material and synthetic chemistry, to chiral catalysts.^1,2^

An important subset of the sulfur-containing organophosphorus compounds are *S*-aryl phosphorothioates. Many of them are useful in their own right as pesticides^3^ as well as biologically active agents.^4^ Moreover, due to the intrinsic lability of the P–S–Ar linkage, this class of compounds has received considerable interest as intermediates in synthetic organic chemistry. In this context, the *S*-aryl phosphorothioate moiety has been used, for instance, as a protecting group during the synthesis of modified oligonucleotides.^5^ Their other synthetic applications include serving as a convenient precursor for the construction of diverse classes of organophosphorus compounds, such as phosphates,^6^ pyrophosphates,^7^ phosphine oxides,^8^ and aryl-^2e,9^ and vinylphosphonates.^10^

The traditional approaches for the synthesis of *S*-aryl phosphorothioates involve the construction of the P–S bond,^11^ either via the phosphorylation of aryl thiols^12^ or by the reaction of P(III) species with sulfur-centered electrophiles.^13^ Except for a single isolated example,^14^ these methods do not allow for effective stereoselective access to P-stereogenic molecules. Conversely, in the context of recent developments in the stereoselective preparation of P-chiral phosphorothioate diesters,^15^ the alternative synthetic strategy, that is, via the formation of the S–Ar bond, would provide a superior entry to *S*-aryl phosphorothioates in a stereopure form. Such synthetic pathway has, however, been explored to a much lesser extent. Specifically, there exist few reports on oxidative couplings of phosphorothioate diesters with arylboronic acids or electron-rich arenes as well as Sandmeyer reactions employing diazonium and iodonium salts.^16^ Yet most of these processes employ phosphorothioates generated *in situ* by the sulfurization of corresponding H-phosphonates, which cannot be readily accessed as pure enantiomers. Moreover, probable free-radical mechanisms of some of these reactions create additional challenges for performing them in a stereocontrolled manner. Indeed, the synthesis of even a single example of a chiral *S*-aryl phosphorothioate using the above methods has not been demonstrated.

In this context, the group of Schoenebeck disclosed in 2019 a direct *S*-arylation of phosphorothioate diesters with aryl iodides using a dinuclear Pd(I) catalyst (Scheme 1). Although it has been shown that these cross-coupling conditions preserve the stereochemical configuration of chiral centers in the carbon backbone, the method has not been applied to molecules, in which the phosphorus atom itself is a stereocenter.^17^ Building on previous studies by us and others showing that hypervalent iodine(III) reagents allow for a highly efficient aryl transfer to sulfur-based nucleophiles^18^ as well as on seminal preliminary results by Chen et al.,^19^ herein we report the synthesis of *S*-aryl phosphorothioates by the direct arylation of phosphorothioate diesters with diaryliodonium salts (Scheme 1). The developed method is not only efficient, general, and metal-free, but it also maintains the stereochemical integrity of P-stereogenic compounds, enabling for the first time to harness the potential provided by the access to stereopure phosphorothioate diesters.^15^

**Scheme 1:**
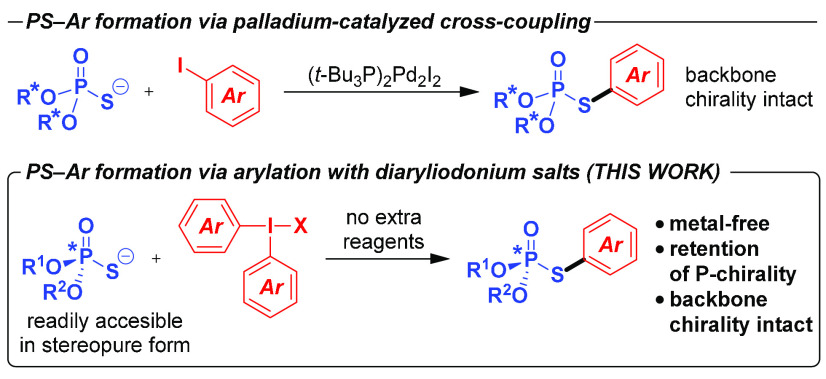
Synthesis of *S*-Aryl Phosphorothioates by Direct *S*-Arylation

We were able to establish a set of conditions, consisting simply of heating the starting materials overnight in 1,4-dioxane at 100 °C under inert atmosphere, under which the arylation of model diphenyl phosphorothioate (**1a**) with diphenyliodonium tetrafluoroborate (**2a**) provides a nearly quantitative yield of the desired product **3a** (see the SI for details). With the optimized reactions conditions in hand, we set out to explore the scope and limitations of this metal-free *S*-arylation of phosphorothioate diesters, first, with regard to the aryl group that can be transferred (Scheme 2). The reaction works well for halide-substituted aryl rings (**3b**–**3e**). Noteworthy, contrary to the palladium-catalyzed counterpart,^17^ aryl bromide is tolerated (**3d**), providing a convenient handle for further functionalization. Aryls containing both diverse electron-withdrawing (**3f**–**3i**) and electron-donating (**3j**–**3l**) substituents in various positions of the ring furnish the desired products with good efficiency. Extended aryl systems, such as 1- and 2-naphthyl, can also be transferred (**3m**,**3n**). Regarding the steric factors, though the considerably hindered mesityl does not interfere with the S–Ar bond formation (**3j**), there is a slight decrease in the yield in the case of the 1-naphthyl moiety (**3n**).

**Scheme 2:**
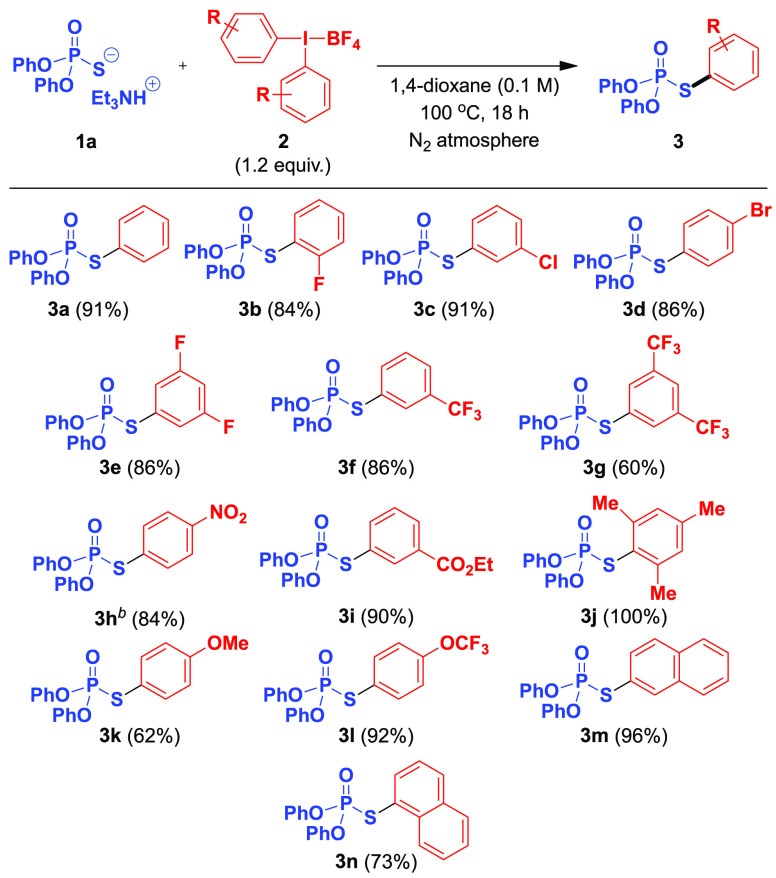
Scope with Regard to the Diaryliodonium Salt Isolated yields. Synthesized using unsymmetrical (4-nitrophenyl)(phenyl)iodonium tetrafluoroborate.

Next, we moved to explore the scope with respect to the phosphorothioate diester (Scheme 3). For simple starting materials, the reaction is uneventful, both in the case of *O*,*O*-diaryl and *O*,*O*-dialkyl substrates (**3a**, **3o**–**3q**). The single limitation is a very sterically hindered *O*,*O*-di-*tert*-butyl phosphorothioate, which was found to be completely unreactive (**3r**). The reaction was then tested using more complex molecules, relevant to asymmetric catalysis and biological applications. To this end, a TADDOL-derived phosphorothioate could be *S*-arylated in a nearly quantitative yield without any disruption to the backbone stereocenters (**3s**). This result demonstrates that the developed method is fully interchangeable with the palladium-catalyzed cross-coupling reported previously,^17^ while it avoids a possible contamination of the chiral product with trace transition metal residues, which may be of importance in downstream catalytic applications. Moreover, the enantiopurity of an axially chiral BINOL-containing substrate also remained intact, although the reaction proceeds in much lower yield in this case (**3t**). Most importantly, however, the *S*-arylation with a diaryliodonium salt could be performed with a complete stereospecificity on dinucleoside phosphorothioates having the opposite sense of chirality at the phosphorus stereocenter (**3u**; Figure 1).^20^ Not only these are the first instances of such transformation, but they also show the applicability of this chemistry for a selective late-stage functionalization of complex, functional-group-rich molecules.

**Scheme 3:**
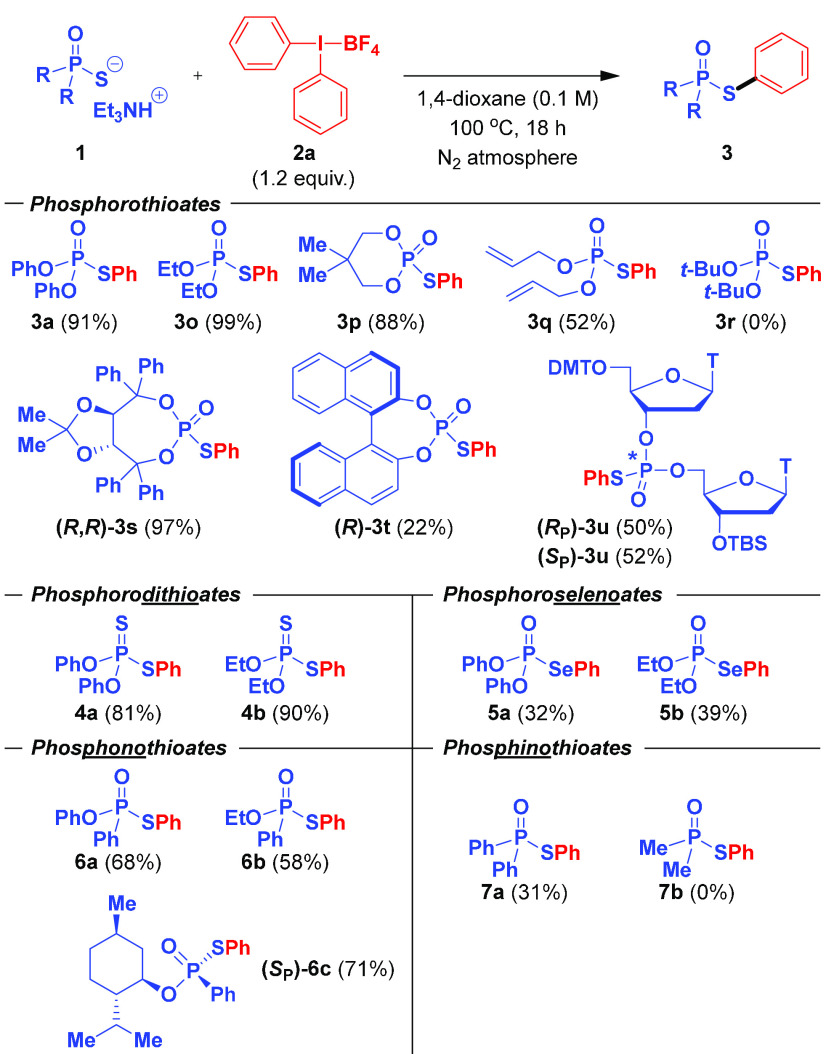
Scope with Regard to the Phosphorothioate Diester and Related Compounds Isolated yields. DMT = 4,4′-dimethoxytrityl, T = thymin-1-yl, TBS = *tert*-butyldimethylsilyl.

**Figure 1:**
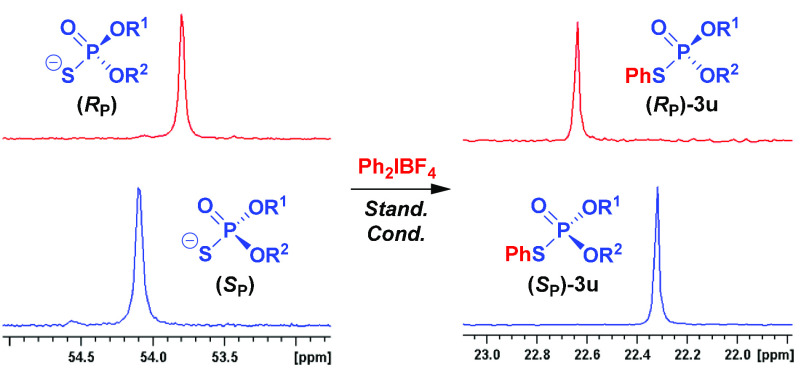
^31^P NMR spectra demonstrating complete stereospecificity of the reaction with P-stereogenic dinucleoside phosphorothioates. R^1^ = 5′-*O*-DMT-thymidin-3′-yl, R^2^ = 3′-*O*-TBS-thymidin-5′-yl.

To further extend the scope, other P–S nucleophiles and related selenium compounds were subjected to the developed arylation conditions. Thus, *S*-aryl phosphorodithioates, both *O*,*O*-diaryl (**4a**) and *O*,*O*-dialkyl (**4b**), were successfully obtained in high yields. Moreover, the aryl transfer to the selenium atom of phosphoroselenoates could also be achieved, although with considerably lower efficacy (**5a**,**5b**). Finally, it was determined that replacing alkoxy groups at phosphorus with carbon substituents gradually decreases the reactivity toward diaryliodonium salts. Namely, the introduction of a single P–C bond into the starting material resulted in a 20–30% drop in the yield of the corresponding *S*-aryl phosphonate products (**6a** vs **3a**; **6b** vs **3o**). However, a synthetically useful yield was obtained in the case of a phosphonate(−)-menthol derivative (**6c**), for which the *S*-arylation was found to also be fully stereospecific. In turn, the presence of two P–C bonds leads to the formation of only 31% of *S*-phenyl diphenylphosphinothioate (**7a**) and a complete loss of the reactivity for dimethylphosphinothioate substrate (**7b**).

To obtain some insight into the mechanism of the *S*-arylation of phosphorothioate diesters with diaryliodonium salts, the reaction between **1a** and **2a** was performed in the presence of either 2,2,6,6-tetramethylpiperidine 1-oxyl (TEMPO) or 1,1-diphenylethylene (DPE) (1 equiv. each). In both cases, the yield was not affected (>95%), speaking against the involvement of radical intermediates.

The mechanism of the reaction was also subject to computational investigations using density functional theory calculations. In particular, we sought to elucidate the details of the S–Ar bond formation and to rationalize the selectivity in terms of *S*- over *O*-arylation. The computed free energy profile for the reaction is depicted in Figure 2.

**Figure 2:**
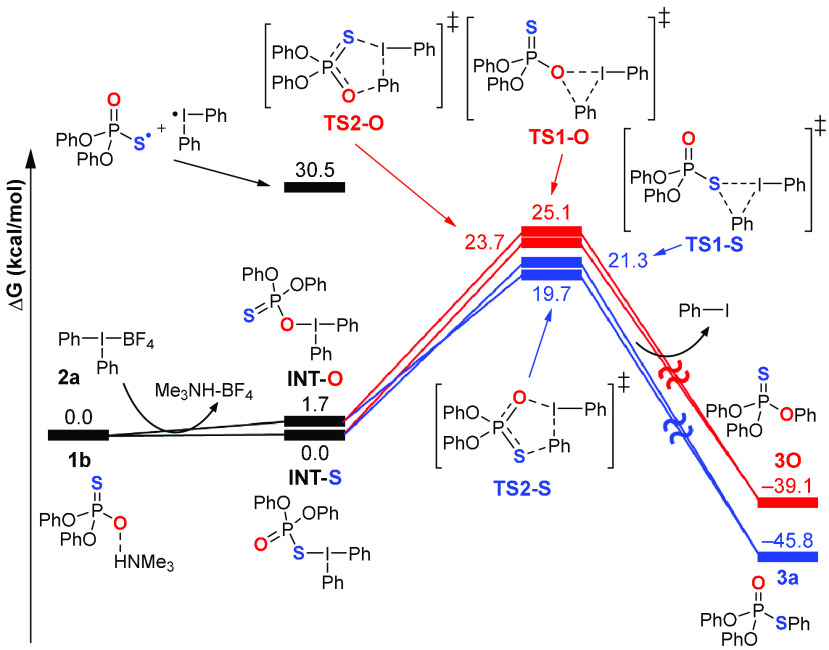
Free energy profile of aryl transfer from diaryliodonium salt to phosphorothioate diester calculated at the B3LYP-D3BJ(SMD)/Def2-QZVP//B3LYP-D3BJ(SMD)/Def2-SVP level of theory in 1,4-dioxane.

Despite multiple attempts, we could not locate a transition state for the outer sphere pathway, that is, a direct nucleophilic attack of model phosphorothioate **1b**, neither with sulfur nor oxygen, on the phenyl ring of **2a**, substituting an iodine-based leaving group in an S_N_2 fashion.^21^ Conversely, the incorporation of phosphorothioate as a ligand into the inner coordination sphere of iodine generates intermediates with either P–S–I or P–O–I linkages (**INT-S** and **INT-O**, respectively), which are relatively close in energy to both **1b** and each other, implying that these species can exist in an equilibrium. A homolytic cleavage of the S/O–I bond in **INT-S**/**INT-O** is calculated to be highly endergonic (∼30 kcal/mol), precluding the radical course of the reaction, as already indicated by the experiments with TEMPO and DPE. From both intermediates, the aryl transfer may take place via two distinct pathways, involving either three- or five-membered cyclic transition states (**TS1** and **TS2**, respectively) that diverge into the *S*- and *O*-arylation products. The S–Ar-forming **TS1-S** (from **INT-S**) and **TS2-S** (from **INT-O**) are clearly energetically preferred to the O–Ar-forming **TS1-O** (from **INT-O**) and **TS2-O** (from **INT-S**), explaining the completely selective *S*-arylation observed experimentally. Interestingly, the five-membered cyclic structures are favored in both pairs of the respective transition states, likely due to their less strained nature. In general, the inner sphere mechanism established by the current computations shares similarities to those found for other aryl transfers employing diaryliodonium salts.^18e,22^ However, the five-membered cyclic TS is unique, attributed to the intrinsic structure of a phosphorothioate diester, bearing two nucleophilic sites in a 1,3-arrangement. The computational studies also indicate that the *S*-arylation of P-chiral phosphorothioates should proceed stereospecifically, as indeed observed experimentally, with the retention of configuration at the phosphorus atom, whose integrity is maintained throughout the mechanistic pathway.

In conclusion, we have successfully developed an efficient protocol for the direct *S*-arylation of phosphorothioate diesters with diaryliodonium salts. The method constitutes an operationally simple and metal-free entry to a variety of *S-*aryl phosphorothioates and related compounds that is also suitable for a late-stage functionalization of complex molecules. Very importantly, the reaction proceeds with a full retention of the stereochemical configuration at the phosphorus atom, as proven experimentally and computationally, thus, benefiting from the easily accessible pool of stereodefined P-stereogenic phosphorothioate diesters. Finally, with the use of DFT calculations, the arylation has been shown to proceed via an inner sphere mechanism, through a five-membered cyclic transition state.

## Data Availability

The data underlying this study are available in the published article and its Supporting Information.

## Supporting Information Available

Experimental procedures, effect of reaction parameters, preparation of starting materials, characterization data and NMR spectra, computational details and data (PDF)
