# Supplementary material for: Metal-Free S-Arylation of Phosphorothioate Diesters and Related Compounds with Diaryliodonium Salts
Source: Org Lett. 2023 Jan 20;25(4):671–5. doi: 10.1021/acs.orglett.2c04310 (PMC9903330; doi:10.1021/acs.orglett.2c04310)

SUPPORTING INFORMATION

Metal-Free *S*-Arylation of Phosphorothioate Diesters and Related Compounds with Diaryliodonium Salts

Sudeep Sarkar[†,‡] and Marcin Kalek*,[†]

[†]Centre of New Technologies, University of Warsaw, Banacha 2C, 02-097 Warsaw, Poland

[‡]Faculty of Chemistry, University of Warsaw, Pasteura 1, 02-093 Warsaw, Poland

*E-mail: m.kalek@cent.uw.edu.pl

Table of Contents

1. General Information 1
2. Effect of Reaction Parameters 2
3. Preparation of Starting Materials 2
4. Arylation of Phosphorothioate Diesters and Related Compounds with Diaryliodonium Salts 11
5. Computational Details 25
6. References 26
7. Structures and Cartesian Coordinates of Stationary Points 29
8. NMR Spectra 36

1. General Information

Unless otherwise noted, all materials were purchased from commercial suppliers and used without purification. Anhydrous tetrahydrofuran was purified prior to use by passage through a column of neutral alumina under nitrogen. Triethylamine was rendered anhydrous by storing over molecular sieves 4Å. Anhydrous 1,4-dioxane was purchased in a septa-sealed bottle and was stored under nitrogen.

^{1}H, ^{13}C, ^{19}F, and ^{31}P NMR spectroscopic data were collected on Varian 400 MHz and Bruker 500 MHz spectrometers at ambient temperature. The chemical shifts are reported in ppm relative to solvent peaks. Mass spectra were recorded on Thermo QExactive mass spectrometer in ESI ionization mode with TOF mass analyzer. IR spectra were recorded on

Shimadzu FT-IR spectrometer equipped with an ATR unit for direct measurements of solid and liquid samples. HPLC analysis of compound **3t** was carried out on Shimazu LC20AD.

2. Effect of Reaction Parameters

Table S1.

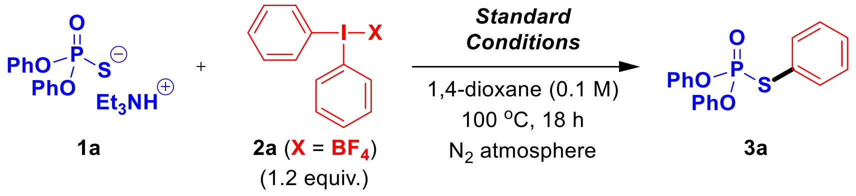

Entry	Change from the Standard Conditions	Yield (%)[a]
1	none	99
2	80 °C, instead of 100 °C	65
3	rt, instead of 100 °C	0
4	toluene, instead of 1,4-dioxane	94
5	CPME, instead of 1,4-dioxane	90
6	DMF, instead of 1,4-dioxane	63
7	DCE, instead of 1,4-dioxane @ 80 °C	43
8	MeCN, instead of 1,4-dioxane @ 80 °C	29
9	Cyclohexane, instead of 1,4-dioxane @ 80 °C	19
10	X = $OOCCF_3$, instead of X = BF_4	96
11	X = OTs, instead of X = BF_4	97
12	X = OTf, instead of X = BF_4	89
13	X = AsF_6, instead of X = BF_4	88
14	X = PF_6, instead of X = BF_4	77
15	X = Cl, instead of X = BF_4	22
16	phenylbenziodoxolone, instead of **2a**	0
17	under air, instead of N_2	90

[a] Yields are the average of two experiments and were determined by ^{1}H NMR spectroscopy; CPME = cyclopentyl methyl ether, DCE = 1,2-dichloroethane.

3. Preparation of Starting Materials

Diaryliodonium salts were prepared according to previously reported procedures (see specific entries in Section 3 for references). Phosphorothioate diesters and related P–S/Se compounds were prepared as described below.

General procedure A

An oven dried round bottom flask was charged with H-phosphonate diester/H-phosphinate/phosphine oxide (1.00 equiv.) and sulfur/selenium (1.05 equiv.). The flask was capped with a rubber septum and evacuated/back-filled with nitrogen. A 1:1 mixture of ethyl acetate:diethyl ether (8 mL/mmol substrate; limiting substrate conc. 0.125 M) was added via syringe and the reaction mixture was cooled down to 0 °C. Anhydrous triethylamine (3 equiv.) was added dropwise, the reaction mixture was allowed to warm up to room temperature, and it was stirred overnight. The solvents were evaporated under reduced pressure and the product was purified by column chromatography or by washing with diethyl ether.

Triethylammonium *O,O*-diphenyl phosphorothioate (1a)

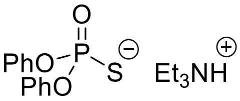

The title compound was prepared according to the general procedure A from diphenyl H-phosphonate (1.38 g, 5.00 mmol), sulfur (169 mg, 5.25 mmol), and anhydrous triethylamine (2.10 mL, 15.0 mmol). After purification by column chromatography (silica; dichloromethane:methanol:triethylamine/93:2:5), the product was obtained as white solid (1.75 g, 95%). **^{1}H NMR** (500 MHz, $CDCl_3$) δ 11.44 (br, 1H), 7.34 – 7.18 (m, 8H), 7.04 (t, J = 7.2 Hz, 2H), 2.99 – 2.88 (m, 6H), 1.17 (t, J = 7.2 Hz, 9H). **^{13}C NMR** (126 MHz, $CDCl_3$): δ 152.8 (d, J = 8.6 Hz), 129.1, 123.6, 121.2 (d, J = 4.8 Hz), 45.7, 8.5. **^{31}P NMR** (203 MHz, $CDCl_3$): δ 49.2. **FT-IR** (ATR): 2493, 1581, 1484, 1207, 1138, 881 cm^{-1}. **HRMS** (ESI) *m/z*: $[M+H]^+$ Calcd for $C_{18}H_{27}NO_3PS$ 368.1444; Found 368.1444.

Triethylammonium *O,O*-diethyl phosphorothioate

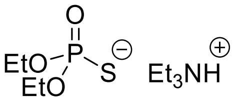

The title compound was prepared according to the General procedure A from diethyl H-phosphonate (324 mg, 2.30 mmol), sulfur (73.7 mg, 2.41 mmol), and anhydrous triethylamine (0.96 mL, 6.90 mmol). After purification by column chromatography (silica; dichloromethane:methanol:triethylamine/93:2:5), the product was obtained as colorless liquid (620 mg, 99%). **^{1}H NMR** (500 MHz, $CDCl_3$) δ 12.29 (br, 1H), 4.01 – 3.85 (m, 4H), 3.10 – 3.02 (m, 6H), 1.27 (t, J = 7.4 Hz, 9H), 1.20 (t, J = 7.1 Hz, 6H). **^{13}C NMR** (126 MHz, $CDCl_3$): δ 61.8 (d, J = 5.9 Hz), 45.5, 16.3 (d, J = 8.2 Hz), 8.6. **^{31}P NMR** (203 MHz, $CDCl_3$): δ 57.4. **FT-IR** (ATR): 1112, 1033, 936, 746 cm^{-1}. **HRMS** (ESI) *m/z*: $[M+H]^+$ Calcd for $C_{10}H_{27}NO_3PS$ 272.1444; Found 272.1440.

Triethylammonium 5,5-dimethyl-1,3,2-dioxaphosphinane-2-thiolate 2-oxide

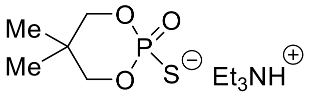

The title compound was prepared according to the General procedure A from 5,5-dimethyl-1,3,2-dioxaphosphorinan-2-one (782 mg, 5.00 mmol), sulfur (168 mg, 5.25 mmol), and anhydrous triethylamine (2.09 mL, 15.00 mmol). After purification by column chromatography (silica; dichloromethane:methanol:triethylamine/93:2:5), the product was obtained as white solid (674 mg, 48%). **^{1}H NMR** (500 MHz, $CDCl_3$) δ 12.12 (br, 1H), 4.30 (dd, *J* = 10.5, 4.6 Hz, 2H), 3.65 (dd, *J* = 24.8, 10.8 Hz, 2H), 3.20 – 3.08 (m, 6H), 1.38 (t, *J* = 7.4 Hz, 9H), 1.22 (s, 3H), 0.81 (s, 3H). **^{13}C NMR** (126 MHz, $CDCl_3$): δ 76.2 (d, *J* = 5.3 Hz), 45.8, 32.8 (d, *J* = 5.0 Hz), 23.0, 21.0, 8.8. **^{31}P NMR** (203 MHz, $CDCl_3$): δ 53.8. **FT-IR** (ATR): 1472, 1133, 1049, 1004, 770 cm^{-1}. **HRMS** (ESI) *m/z*: $[M+H]^+$ Calcd for $C_{11}H_{27}NO_3PS$ 284.1444; Found 284.1440.

Triethylammonium *O,O*-diallyl phosphorothioate

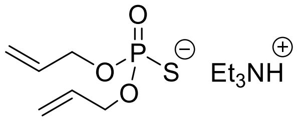

The title compound was prepared according to the General procedure A from diallyl H-phosphonate (500 mg, 3.08 mmol), sulfur (104 mg, 3.24 mmol), and anhydrous triethylamine (0.94 mL, 9.26 mmol). After purification by column chromatography (silica; dichloromethane:methanol:triethylamine/93:2:5), the product was obtained as brown liquid (474 mg, 52%). **^{1}H NMR** (500 MHz, $CDCl_3$) δ 12.07 (br, 1H), 6.01 – 5.89 (m, 2H), 5.30 (dq, *J* = 17.2, 1.7 Hz, 2H), 5.11 (d, *J* = 10.6 Hz, 2H), 4.56 – 4.36 (m, 4H), 3.15 – 3.06 (m, 6H), 1.32 (t, *J* = 7.3 Hz, 9H). **^{13}C NMR** (126 MHz, $CDCl_3$): δ 134.9 (d, *J* = 8.5 Hz), 116.3, 67.1 (d, *J* = 5.4 Hz), 45.7, 8.7. **^{31}P NMR** (203 MHz, $CDCl_3$): δ 58.4. **FT-IR** (ATR): 2921, 1447, 1106, 1009, 808 cm^{-1}. **HRMS** (ESI) *m/z*: $[M+H]^+$ Calcd for $C_{12}H_{27}NO_3PS$ 296.1444; Found 296.1441.

Triethylammonium *O,O*-di-*tert*-butyl phosphorothioate

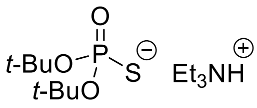

The title compound was prepared according to the General procedure A from di-*tert*-butyl H-phosphonate (500 mg, 2.44 mmol), sulfur (82.1 mg, 2.56 mmol), and anhydrous triethylamine (1.02 mL, 7.32 mmol). After purification by column chromatography (silica; dichloromethane:methanol:triethylamine/93:2:5), the product was obtained as pale orange solid (800 mg, 100%). **^{1}H NMR** (500 MHz, $CDCl_3$) δ 13.03 (br, 1H), 3.13 (q, *J* = 7.3 Hz, 6H), 1.50 (s, 18H), 1.31 (t, *J* = 7.3 Hz, 9H). **^{13}C NMR** (126 MHz, $CDCl_3$): δ 78.9 (d, *J* = 9.3 Hz), 45.3, 30.4 (d, *J* = 4.4 Hz), 8.6. **^{31}P NMR** (203 MHz, $CDCl_3$): δ 44.2. **FT-IR** (ATR): 2980, 1366,

1242, 1175, 1087, 984, 813 cm^{-1}. **HRMS** (ESI) *m/z*: $[M+H]^+$ Calcd for $C_{14}H_{35}NO_3PS$ 328.2070; Found 328.2065.

Triethylammonium (3a*R*,8a*R*)-2,2-dimethyl-4,4,8,8-tetraphenyltetrahydro-[1,3]dioxolo[4,5-e][1,3,2]dioxaphosphepine-6-thiolate 6-oxide

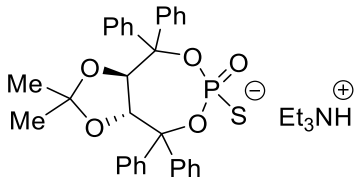

The title compound was prepared according to the General procedure A from (3a*R*,8a*R*)-2,2-dimethyl-4,4,8,8-tetraphenyltetrahydro-[1,3]dioxolo[4,5-e][1,3,2]dioxaphosphepine 6-oxide[1] (310 mg, 0.58 mmol), sulfur (19.5 mg, 0.61 mmol), and anhydrous triethylamine (0.24 mL, 1.74 mmol). The solvents were evaporated under reduced pressure and diethyl ether (50 mL) was added to the residue. The solid was filtered and washed twice with diethyl ether. The product was obtained as white solid (365 mg, 97%). **^{1}H NMR** (500 MHz, $CDCl_3$) δ 11.68 (br, 1H), 7.88 (d, *J* = 7.9 Hz, 2H), 7.72 (d, *J* = 7.9 Hz, 2H), 7.53 – 7.42 (m, 4H), 7.28 – 7.10 (m, 12H), 5.92 (d, *J* = 8.7 Hz, 1H), 4.74 (d, *J* = 8.7 Hz, 1H), 2.46 – 2.25 (m, 6H), 1.46 (s, 3H), 0.83 (t, *J* = 7.3 Hz, 9H), 0.16 (s, 3H). **^{13}C NMR** (126 MHz, $CDCl_3$): δ 148.4, 146.3 (d, *J* = 11.6 Hz), 143.9, 141.7 (d, *J* = 10.4 Hz), 129.7, 128.9, 127.69, 127.66, 127.6, 127.5, 127.14, 127.09, 127.05, 127.0, 126.5, 126.4, 110.8, 85.1 (d, *J* = 10.2 Hz), 83.4 (d, *J* = 11.4 Hz), 83.0, 80.3, 45.4, 28.1, 25.0, 8.7. **^{31}P NMR** (203 MHz, $CDCl_3$): δ 51.9. **FT-IR** (ATR): 2189, 1489, 1447, 1131, 1001, 696 cm^{-1}. **HRMS** (ESI) *m/z*: $[M+H]^+$ Calcd for $C_{37}H_{45}NO_5PS$ 646.2751; Found 646.2759.

Triethylammonium (*R*)-dinaphtho[2,1-d:1',2'-f][1,3,2]dioxaphosphepine-4-thiolate 4-oxide

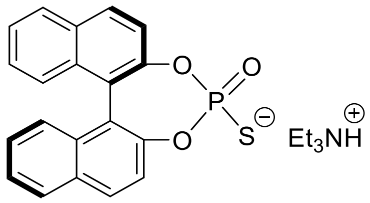

The title compound was prepared according to the General procedure A from (*R*)-1,1'-binaphthyl-2,2'-diyl H-phosphonate[1] (900 mg, 2.60 mmol), sulfur (87.5 mg, 2.73 mmol), and anhydrous triethylamine (1.08 mL, 7.80 mmol). The solvents were evaporated under reduced pressure and diethyl ether (100 mL) was added to the residue. The solid was filtered and washed twice with diethyl ether. The product was obtained as white solid (1.20 g, 99%). **^{1}H NMR** (500 MHz, $CDCl_3$) δ 9.64 (br, 1H), 8.16 – 7.72 (m, 4H), 7.51 – 7.36 (m, 3H), 7.35 – 6.68 (m, 5H), 3.01 (q, *J* = 7.3 Hz, 6H), 1.14 (t, *J* = 7.3 Hz, 9H). **^{13}C NMR** (126 MHz, $CDCl_3$): δ 149.6 (d, *J* = 10.8 Hz), 132.0, 130.5 (d, *J* = 6.3 Hz), 129.6 (d, *J* = 19.2 Hz), 128.4, 128.3 – 125.9 (m), 124.6 (d, *J* = 9.4 Hz), 122.9 (d, *J* = 48.3 Hz), 122.1 (dd, *J* = 27.3, 2.8 Hz), 45.4, 8.5. **^{31}P NMR** (203

MHz, $CDCl_3$): δ 64.6. **FT-IR** (ATR): 2975, 2496, 1505, 1233, 1151, 1070, 952, 750 cm^{-1}. **HRMS** (ESI) *m/z*: $[M+H]^+$ Calcd for $C_{26}H_{29}NO_3PS$ 466.1600; Found 466.1596.

Triethylammonium (*R*P)-3′-*O*-(*tert*-butyldimethylsilyl)thymidin-5′-yl 5′-*O*-dimethoxytritylthymidin-3′-yl phosphorothioate

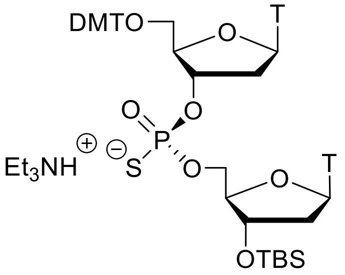

The title compound was prepared according to the General procedure A from (S_P)-3′-*O*-(*tert*-butyldimethylsilyl)thymidin-5′-yl 5′-*O*-dimethoxytritylthymidin-3′-yl H-phosphonate[2] (170 mg, 0.18 mmol), sulfur (6.06 mg, 0.19 mmol), and anhydrous triethylamine (0.08 mL, 0.54 mmol). The solvents were evaporated under reduced pressure and diethyl ether (50 mL) was added to the residue. The solid was filtered and washed twice with diethyl ether. The product was obtained as white solid (190 mg, 98%). **^{1}H NMR** (500 MHz, DMSO-d_6) δ 11.34 (s, 1H), 11.24 (s, 1H), 9.20 (br, 1H), 7.85 (s, 1H), 7.67 – 7.52 (m, 3H), 7.48 (s, 1H), 7.43 – 7.36 (m,2H), 7.34 – 7.17 (m, 7H), 6.92 – 6.83 (m, 4H), 6.27 – 6.14 (m, 2H), 5.21 – 5.06 (m, 1H) 4.55 – 4.43 (m, 1H), 4.24 – 4.16 (m, 1H), 3.98 – 3.80 (m, 3H), 3.73 (s, 6H), 3.18 – 3.01 (m, 7H), 2.40 – 2.15 (m, 3H), 2.03 – 1.93 (m, 1H), 1.79 (s, 3H), 1.30 (s, 3H), 1.17 (t, *J* = 7.2 Hz, 9H), 0.85 (s, 9H), 0.06 (s, 6H). **^{13}C NMR** (126 MHz, DMSO-d_6): δ 163.7 (d, *J* = 24.1 Hz), 158.2, 150.5 (d, *J* = 24.5 Hz), 144.6, 136.2, 135.4, 135.3, 135.1, 129.7, 127.8 (d, *J* = 29.1 Hz), 126.8, 113.3, 109.8 (d, *J* = 26.4 Hz), 86.2, 86.1, 84.7, 83.8, 75.3, 73.2, 64.1 (d, *J* = 42.7 Hz), 55.0, 45.7, 38.3, 25.7, 17.7, 12.1, 11.5, 8.6, -4.9. **^{31}P NMR** (203 MHz, DMSO-d_6): δ 53.8. **FT-IR** (ATR): 1684, 1508, 1459, 829 cm^{-1}. **HRMS** (ESI) *m/z*: $[M+H]^+$ Calcd for $C_{53}H_{75}N_5O_{13}PSSi$ 1080.4584; Found 1080.4583.

Triethylammonium (*S*P)-3′-*O*-(*tert*-butyldimethylsilyl)thymidin-5′-yl 5′-*O*-dimethoxytritylthymidin-3′-yl phosphorothioate

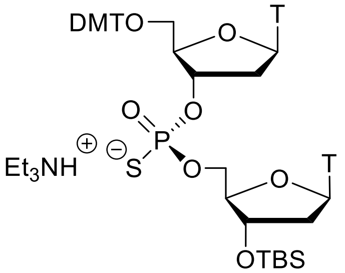

The title compound was prepared according to the General procedure A from (R_P)-3′-*O*-(*tert*-butyldimethylsilyl)thymidin-5′-yl 5′-*O*-Dimethoxytritylthymidin-3′-yl H-phosphonate[2] (230

mg, 0.24 mmol), sulfur (8.18 mg, 0.26 mmol), and anhydrous triethylamine (0.10 mL, 0.73 mmol). The solvents were evaporated under reduced pressure and diethyl ether (50 mL) was added to the residue. The solid was filtered and washed twice with diethyl ether. The product was obtained as white solid (260 mg, 99%). **^{1}H NMR** (500 MHz, DMSO-d_6) δ 11.34 (s, 1H), 11.25 (s, 1H), 9.26 (br, 1H), 7.81 (s, 1H), 7.53 – 7.35 (m, 2H), 7.48 (s, 1H), 7.35 – 7.17 (m, 7H), 6.94 – 6.82 (m, 4H), 6.26 – 6.14 (m, 2H), 5.17 – 5.05 (m, 1H) 4.50 – 4.43 (m, 1H), 4.18 – 4.10 (m, 1H), 3.97 – 3.87 (m, 1H), 3.86 (s, 1H), 3.81 – 3.74 (m, 1H), 3.72 (s, 6H), 3.24 – 3.04 (m, 8H), 2.41 – 2.20 (m, 2H), 2.19 – 2.10 (m, 1H), 1.94 (dd, *J* = 12.9, 5.9 Hz, 1H)1.78 (s, 3H), 1.33 (s, 3H), 1.17 (t, *J* = 7.3 Hz, 9H), 0.85 (s, 9H), 0.04 (s, 6H). **^{13}C NMR** (126 MHz, DMSO-d_6): δ 163.7 (d, *J* = 18.7 Hz), 158.2, 150.5 (d, *J* = 23.8 Hz), 144.6, 136.1, 135.7, 135.4, 135.1, 129.7, 127.8 (d, *J* = 31.3 Hz), 126.8, 113.3, 109.8 (d, *J* = 31.9 Hz), 86.2, 86.1, 84.4, 83.7, 75.1, 73.2, 64.3 (d, *J* = 110.7 Hz), 55.0, 45.7, 38.3, 25.7, 17.6, 12.1, 11.5, 8.6, -4.9. **^{31}P NMR** (203 MHz, DMSO-d_6): δ 54.1. **FT-IR** (ATR): 1653, 1505, 1464, 1248, 1108, 801 cm^{-1}. **HRMS** (ESI) *m/z*: $[M+H]^+$ Calcd for $C_{53}H_{75}N_5O_{13}PSSi$ 1080.4584; Found 1080.4583.

Triethylammonium *O,O*-diphenyl phosphorodithioate

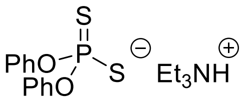

An oven dried round bottom flask was charged with *O*,*O*-diphenyl dithiophosphoric acid[3] (1.13 g, 4.00 mmol). The flask was capped with a rubber septum and evacuated/back-filled with nitrogen. Anhydrous tetrahydofuran (5 mL) was added via syringe, and resulting mixture was cooled down to 0 °C. Anhydrous triethylamine (1.67 mL, 12 mmol) was added dropwise, the reaction mixture was allowed to reach room temperature and stirred overnight. The solvent was evaporated under reduced pressure, and the residue was dissolved in minimal amount of dichloromethane and applied on the top of silica column. After purification by column chromatography (silica; dichloromethane:methanol:triethylamine/93:2:5), the product was obtained as brown liquid (465 mg, 30%). **^{1}H NMR** (500 MHz, $CDCl_3$) δ 9.53 (br, 1H), 7.43 – 7.35 (m, 4H), 7.33 – 7.26 (m, 4H), 7.11 (t, *J* = 7.4 Hz, 2H), 3.15 (q, *J* = 7.3 Hz, 6H), 1.28 (t, *J* = 7.3 Hz, 9H). **^{13}C NMR** (126 MHz, $CDCl_3$): δ 152.7 (d, *J* = 10.2 Hz), 129.0 (d, *J* = 1.3 Hz), 124.1 (d, *J* = 1.6 Hz), 122.4 (d, *J* = 4.7 Hz), 46.3, 8.6. **^{31}P NMR** (203 MHz, $CDCl_3$): δ 107.8. **FT-IR** (ATR): 2954, 2663, 1588, 1486, 1193, 869, 685 cm^{-1}. **HRMS** (ESI) *m/z*: $[M+H]^+$ Calcd for $C_{18}H_{27}NO_2PS_2$ 384.1215; Found 384.1213.

Triethylammonium *O,O*-diethyl phosphorodithioate

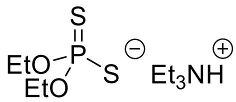

An oven dried round bottom flask was charged with *O*,*O*-diethyl dithiophosphoric acid[4] (745 mg, 4.00 mmol). The flask was capped with a rubber septum and evacuated/back-filled with nitrogen. Anhydrous tetrahydofuran (5 mL) was added via syringe, and resulting mixture was

cooled down to 0 °C. Anhydrous triethylamine (1.67 mL, 12 mmol) was added dropwise, the reaction mixture was allowed to reach room temperature and stirred overnight. The solvent was evaporated under reduced pressure, and the residue was dissolved in minimal amount of dichloromethane and applied on the top of silica column. After purification by column chromatography (silica; dichloromethane:methanol:triethylamine /93:2:5), the product was obtained as pale yellow liquid (1.10 g, 96%). **^{1}H NMR** (500 MHz, $CDCl_3$) δ 10.10 (br, 1H), 4.11 – 3.97 (m, 4H), 3.25 (q, *J* = 7.3 Hz, 6H), 1.37 (t, *J* = 7.3 Hz, 9H), 1.28 (t, *J* = 7.1 Hz, 6H). **^{13}C NMR** (126 MHz, $CDCl_3$): δ 62.0 (d, *J* = 7.6 Hz), 46.2, 16.3 (d, *J* = 8.6 Hz), 8.7. **^{31}P NMR** (203 MHz, $CDCl_3$): δ 110.7. **FT-IR** (ATR): 2974, 1470, 1385, 1020, 923, 754, 657 cm^{-1}. **HRMS** (ESI) *m/z*: $[M+H]^+$ Calcd for $C_{10}H_{27}NO_2PS_2$ 288.1215; Found 288.1216.

Triethylammonium *O,O*-diphenyl phosphoroselenoate

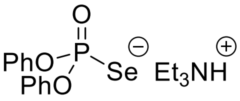

The title compound was prepared according to the General procedure A from diphenyl H-phosphonate (500 mg, 2.21 mmol), selenium (183 mg, 2.32 mmol), and anhydrous triethylamine (0.91 mL, 6.63 mmol). After purification by column chromatography (silica; dichloromethane:methanol:triethylamine/93:2:5), the product was obtained as white solid (853 mg, 93%). **^{1}H NMR** (500 MHz, $CDCl_3$) δ 11.42 (br, 1H), 7.37 – 7.30 (m, 4H), 7.30 – 7.23 (m, 4H), 7.08 (t, *J* = 7.4 Hz, 2H), 3.03 (q, *J* = 7.4 Hz, 6H), 1.24 (t, *J* = 7.4 Hz, 9H). **^{13}C NMR** (126 MHz, $CDCl_3$): δ 152.7 (d, *J* = 9.0 Hz), 129.2, 123.9, 121.7 (d, *J* = 4.6 Hz), 45.9, 8.7. **^{31}P NMR** (203 MHz, $CDCl_3$): δ 42.5. **FT-IR** (ATR): 2987, 2611, 2505, 1587, 1484, 1229, 1189, 1154, 870, 693 cm^{-1}. **HRMS** (ESI) *m/z*: $[M+H]^+$ Calcd for $C_{18}H_{27}NO_3PSe$ 416.0888; Found 416.0891.

Triethylammonium *O,O*-diethyl phosphoroselenoate

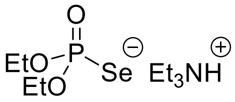

The title compound was prepared according to the General procedure A from diethyl H-phosphonate (500 mg, 3.83 mmol), selenium (318 mg, 4.02 mmol) instead of sulfur and anhydrous triethylamine (1.60 mL, 11.5 mmol). After purification by column chromatography (silica; dichloromethane:methanol:triethylamine/93:2:5), the product was obtained as yellow liquid (1.18 g, 97%). **^{1}H NMR** (500 MHz, $CDCl_3$) δ 12.12 (br, 1H), 4.10 – 3.94 (m, 4H), 3.14 (q, *J* = 7.3 Hz, 6H), 1.33 (t, *J* = 7.3 Hz, 9H), 1.28 (t, *J* = 7.1 Hz, 6H). **^{13}C NMR** (126 MHz, $CDCl_3$): δ 62.4, 45.7, 16.3 (d, *J* = 8.2 Hz), 8.7. **^{31}P NMR** (162 MHz, $CDCl_3$): δ 50.6. **FT-IR** (ATR): 2976, 1457, 1387, 1116, 1031, 934, 745 cm^{-1}. **HRMS** (ESI) *m/z*: $[M+H]^+$ Calcd for $C_{10}H_{27}NO_3PSe$ 320.0888; Found 320.0891.

Triethylammonium *O*-phenyl phenylphosphonothioate

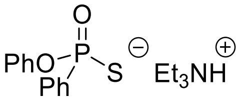

The title compound was prepared according to the General procedure A from phenyl H-phenylphosphinate[5] (557 mg, 2.50 mmol), sulfur (84.2 mg, 2.63 mmol), and anhydrous triethylamine (1.04 mL, 7.5 mmol). After purification by column chromatography (silica; dichloromethane:methanol:triethylamine/93:2:5), the product was obtained as pale yellow liquid (314 mg, 36%). **^{1}H NMR** (500 MHz, $CDCl_3$) δ 12.42 (br, 1H), 8.04 – 7.96 (m, 2H), 7.39 – 7.31 (m, 3H), 7.21 – 7.15 (m, 2H), 7.14 – 7.08 (m, 2H), 6.99 (t, *J* = 7.3 Hz, 1H), 3.13 – 3.04 (m, 6H), 1.27 (t, *J* = 7.4 Hz, 9H). **^{13}C NMR** (126 MHz, $CDCl_3$): δ 152.9 (d, *J* = 8.8 Hz), 140.2 (d, *J* = 140.4 Hz), 130.9 (d, *J* = 10.9 Hz), 130.2 (d, *J* = 2.9 Hz), 128.9, 127.8 (d, *J* = 14.2 Hz), 123.4, 122.2 (d, *J* = 4.5 Hz), 45.5, 8.6. **^{31}P NMR** (203 MHz, $CDCl_3$): δ 68.4. **FT-IR** (ATR): 1487, 1209, 1123, 1091, 1065, 867, 713 cm^{-1}. **HRMS** (ESI) *m/z*: $[M+H]^+$ Calcd for $C_{18}H_{27}NO_2PS$ 352.1495; Found 352.1494.

Triethylammonium *O*-ethyl phenylphosphonothioate

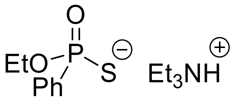

The title compound was prepared according to the General procedure A from ethyl H-phenylphosphinate (900 mg, 5.00 mmol), sulfur (168 mg, 5.25 mmol), and anhydrous triethylamine (2.09 mL, 15.0 mmol). After purification by column chromatography (silica; dichloromethane:methanol:triethylamine/93:2:5), the product was obtained as pale yellow liquid (1.30 g, 86%). **^{1}H NMR** (500 MHz, $CDCl_3$) δ 12.48 (br, 1H), 7.98 – 7.91 (m, 2H), 7.37 – 7.30 (m, 3H), 4.02 – 3.94 (m, 1H), 3.84 – 3.74 (m, 1H), 3.12 (q, *J* = 7.4 Hz, 6H), 1.28 (t, *J* = 7.4 Hz, 9H), 1.20 (t, *J* = 7.1 Hz, 3H). **^{13}C NMR** (126 MHz, $CDCl_3$): δ 139.8 (d, *J* = 138.4 Hz), 130.7 (d, *J* = 10.9 Hz), 130.1 (d, *J* = 3.1 Hz), 127.8 (d, *J* = 13.7 Hz), 61.3 (d, *J* = 5.5 Hz), 45.4, 16.5 (d, *J* = 8.4 Hz), 8.6. **^{31}P NMR** (203 MHz, $CDCl_3$): δ 70.3. **FT-IR** (ATR): 2980, 1436, 1038, 934, 746 cm^{-1}. **HRMS** (ESI) *m/z*: $[M+H]^+$ Calcd for $C_{14}H_{27}NO_2PS$ 304.1495; Found 304.1491.

Triethylammonium *O*-((1*R*,2*S*,5*R*)-2-isopropyl-5-methylcyclohexyl) (*S*)-phenylphosphonothioate

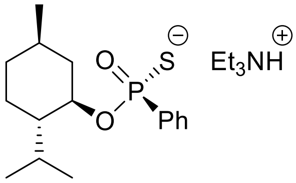

The title compound was prepared according to the General procedure A from (1*R*,2*S*,5*R*)-2-isopropyl-5-methylcyclohexyl (*R*)-H-phenylphosphinate (97:3 dr at phosphorus)[6] (73 mg, 0.25 mmol), sulfur (8.42 mg, 0.26 mmol), and anhydrous triethylamine (0.11 mL, 0.75 mmol). After purification by column chromatography (silica; dichloromethane:methanol:triethylamine/94:1:5), the product was obtained as brown liquid (90 mg, 87%; 97:3 dr at phosphorus). **^{1}H NMR** (500 MHz, $CDCl_3$) δ 12.79 (br, 1H), 8.02 – 7.92 (m, 2H), 7.36 – 7.29 (m, 3H), 4.22 (qd, *J* = 10.5, 4.4 Hz, 1H), 3.26 – 3.00 (m, 6H), 2.41 – 2.33 (m, 1H), 1.98 – 1.87 (m, 1H), 1.63 – 1.51 (m, 2H), 1.47 – 1.36 (m, 1H), 1.31 – 1.23 (m, 1H), 1.28 (t, *J* = 7.3 Hz, 9H), 1.10 (q, *J* = 12.1 Hz, 1H), 0.96 (qd, *J* = 12.9, 3.3 Hz, 1H), 0.84 (d, *J* = 6.5 Hz, 3H), 0.80 (dd, *J* = 12.4, 3.1 Hz, 1H), 0.76 (d, *J* = 7.1 Hz, 3H), 0.53 (d, *J* = 6.9 Hz, 3H). **^{13}C NMR** (126 MHz, $CDCl_3$): δ 141.2 (d, *J* = 144.2 Hz), 130.8 (d, *J* = 10.6 Hz), 129.8 (d, *J* = 2.7 Hz), 127.6 (d, *J* = 13.7 Hz), 76.2 (d, *J* = 7.3 Hz), 48.9 (d, *J* = 8.0 Hz), 45.3, 44.1, 34.7, 31.7, 25.4, 23.1, 22.4, 21.3, 15.9, 8.6. **^{31}P NMR** (203 MHz, $CDCl_3$): δ 67.3. **FT-IR** (ATR): 2951, 2920, 1455, 1120, 1010, 992, 697 cm^{-1}. **HRMS** (ESI) *m/z*: $[M–Et_3NH]^-$ Calcd for $C_{16}H_{24}O_2PS$ 311.1240; Found 311.1234.

Triethylammonium diphenylphosphinothioate

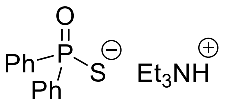

The title compound was prepared according to the General procedure A from diphenylphosphine oxide (500 mg, 2.4 mmol), sulfur (80.8 mg, 2.52 mmol), and anhydrous triethylamine (1.00 mL, 7.20 mmol). After purification by column chromatography (silica; dichloromethane:methanol:triethylamine/93:2:5), the product was obtained as white solid (800 mg, 99%). **^{1}H NMR** (500 MHz, $CDCl_3$) δ 13.28 (br, 1H), 7.96 – 7.87 (m, 4H), 7.34 – 7.27 (m, 6H), 3.10 (q, *J* = 7.4 Hz, 6H), 1.25 (t, *J* = 7.4 Hz, 9H). **^{13}C NMR** (126 MHz, $CDCl_3$): δ 143.0 (d, *J* = 103.1 Hz), 130.8 (d, *J* = 10.2 Hz), 129.5 (d, *J* = 2.8 Hz), 127.8 (d, *J* = 12.4 Hz), 45.1, 8.6. **^{31}P NMR** (203 MHz, $CDCl_3$): δ 57.1. **FT-IR** (ATR): 2135, 1439, 1102, 1047, 758, 699 cm^{-1}. **HRMS** (ESI) *m/z*: $[M+H]^+$ Calcd for $C_{18}H_{27}NOPS$ 336.1546; Found 336.1541.

Triethylammonium dimethylphosphinothioate

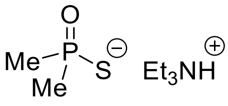

To a solution of dimethylphosphinic chloride (100 mg, 0.86 mmol) in anhydrous tetrahydrofuran (5 mL) was added a solution of hydrogen sulfide in tetrahydrofuran (0.8 M; 1.62 mL, 1.3 mmol) under nitrogen atmosphere at 0 °C. The reaction was stirred for 3 hours at room temperature and the solvents were evaporated under reduced pressure. Anhydrous triethylamine (0.24 mL, 1.73 mmol) was added to the residue at 0 °C and the mixture was stirred for another 3 hours. The reaction mixture was dried under vacuum and used directly without further purification. The product was obtained as colorless liquid (178 mg, 97%). **^{1}H NMR**

(500 MHz, $CDCl_3$) δ 3.10 (q, J = 7.3 Hz, 6H), 1.46 (d, J = 14.2 Hz, 6H), 1.36 (t, J = 7.3 Hz, 9H). **^{13}C NMR** (126 MHz, $CDCl_3$): δ 45.7, 17.3 (d, J = 95.5 Hz), 8.7. **^{31}P NMR** (203 MHz, $CDCl_3$): δ 50.1. **FT-IR** (ATR): 3369, 1646, 1303, 975, 871 cm^{-1}. **HRMS** (ESI) *m/z*: $[M+H]^+$ Calcd for $C_8H_{22}NOPS$ 211.1154; Found 211.0256.

4. Arylation of Phosphorothioate Diesters and Related Compounds with Diaryliodonium Salts

General procedure B

A 20 mL vial was charged with phosphorothioate diester/related P–S/Se compound (0.40 mmol) and diaryliodonium tetrafluoroborate (1.2 equiv., 0.48 mmol). The vial was capped and evacuated/back-filled with nitrogen three times. Anhydrous 1,4-dioxane (8 mL; limiting substrate conc. 0.05 M) was added via syringe and the reaction mixture was stirred in an aluminum heating block at 100 °C for 18 hours. After cooling to room temperature, the solvent was evaporated under reduced pressure. Dichloromethane (2 mL) was added to the residue, followed by a small amount of silica. The solvent was evaporated under reduced pressure, the solid residue was applied on the top of silica column, and the product was purified by column chromatography.

The procedure can also performed in a larger scale (see product **3o** for 1.50 mmol example). In this case, the 20 mL vial is replaced by a round-bottom flask equipped with a reflux condenser, which is closed with a rubber septum and connected via needle to a positive pressure of nitrogen (from balloon or manifold) throughout the reaction.

Triphenyl phosphorothioate (3a)

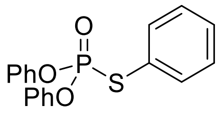

The title compound was prepared according to the General procedure B from triethylammonium *O*,*O*-diphenyl phosphorothioate (**1a**; 147 mg, 0.40 mmol) and diphenyliodonium tetrafluoroborate (**2a**; 177 mg, 0.48 mmol)[8]. After purification by column chromatography (silica; petroleum ether:ethyl acetate/90:10), the product was obtained as pale yellow liquid (124 mg, 91%). **^{1}H NMR** (500 MHz, $CDCl_3$) δ 7.58 – 7.45 (m, 2H), 7.42 – 7.30 (m, 7H), 7.21 (t, J = 7.5 Hz, 6H). **^{13}C NMR** (126 MHz, $CDCl_3$): δ 150.5 (d, J = 8.6 Hz), 135.5 (d, J = 5.3 Hz), 130.0, 129.8 (d, J = 3.4 Hz), 129.7 (d, J = 2.6 Hz), 125.7, 125.2 (d, J = 7.6 Hz), 120.6 (d, J = 5.1 Hz). **^{31}P NMR** (203 MHz, $CDCl_3$): δ 15.1. **FT-IR** (ATR): 1588, 1487, 1269, 1179, 1159, 922, 746 cm^{-1}. **HRMS** (ESI) *m/z*: $[M+H]^+$ Calcd for $C_{18}H_{16}O_3PS$ 343.0552; Found 343.0557. The characterization data is consistent with that reported previously.[7]

***S*-(2-Fluorophenyl) *O,O*-diphenyl phosphorothioate (3b)**

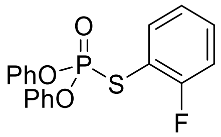

The title compound was prepared according to the General procedure B from triethylammonium *O,O*-diphenyl phosphorothioate (**1a**; 147 mg, 0.40 mmol) and bis(2-fluorophenyl)iodonium tetrafluoroborate (194 mg, 0.48 mmol)[8]. After purification by column chromatography (silica; petroleum ether:ethyl acetate/90:10), the product was obtained as pale yellow liquid (121 mg, 84%). **^{1}H NMR** (500 MHz, $CDCl_3$): δ 7.57 – 7.51 (m, 1H), 7.43 – 7.38 (m, 1H), 7.38 – 7.32 (m, 1H), 7.26 – 7.19 (m, 6H), 7.15 – 7.10 (m, 2H). **^{13}C NMR** (126 MHz, $CDCl_3$): δ 162.9 (dd, *J* = 250.6, 5.7 Hz), 150.5 (d, *J* = 8.6 Hz), 137.9 (d, *J* = 4.6 Hz), 132.4 (dd, *J* = 8.1, 3.5 Hz), 130.0, 125.8, 125.1 (t, *J* = 3.2 Hz), 120.6 (d, *J* = 5.0 Hz), 116.7 (dd, *J* = 22.6, 2.8 Hz), 112.6 (dd, *J* = 18.6, 7.7 Hz). **^{31}P NMR** (203 MHz, $CDCl_3$): δ 13.6. **^{19}F NMR** (376 MHz, $CDCl_3$): δ -104.5 – -104.6 (m). **FT-IR** (ATR): 2921, 1487, 1180, 1158, 934, 756 cm^{-1}. **HRMS** (ESI) *m/z*: $[M+H]^+$ Calcd for $C_{18}H_{15}FO_3PS$ 361.0458; Found 361.0461.

***S*-(3-Chlorophenyl) *O,O*-diphenyl phosphorothioate (3c)**

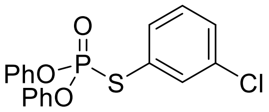

The title compound was prepared according to the General procedure B from triethylammonium *O,O*-diphenyl phosphorothioate (**1a**; 147 mg, 0.40 mmol) and bis(3-chlorophenyl)iodonium tetrafluoroborate (210 mg, 0.48 mmol)[9]. After purification by column chromatography (silica; petroleum ether:ethyl acetate/90:10), the product was obtained as pale yellow liquid (137 mg, 91%). **^{1}H NMR** (500 MHz, $CDCl_3$): δ 7.45 – 7.32 (m, 7H), 7.29 – 7.20 (m, 7H). **^{13}C NMR** (126 MHz, $CDCl_3$): δ 150.4 (d, *J* = 8.3 Hz), 135.1 (d, *J* = 5.5 Hz), 135.1 (d, *J* = 3.2 Hz), 133.5 (d, *J* = 5.5 Hz), 130.6 (d, *J* = 2.7 Hz), 130.10, 130.07, 126.9 (d, *J* = 7.5 Hz), 126.0, 120.6 (d, *J* = 4.8 Hz). **^{31}P NMR** (203 MHz, $CDCl_3$): δ 13.9. **FT-IR** (ATR): 1588, 1487, 1273, 1179, 1159, 933, 764 cm^{-1}. **HRMS** (ESI) *m/z*: $[M+H]^+$ Calcd for $C_{18}H_{15}ClO_3PS$ 377.0163; Found 377.0168. The characterization data is consistent with that reported previously.[7]

***S*-(4-Bromophenyl) *O,O*-diphenyl phosphorothioate (3d)**

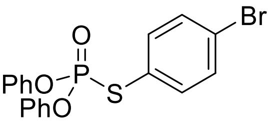

The title compound was prepared according to the General procedure B from triethylammonium *O,O*-diphenyl phosphorothioate (**1a**; 147 mg, 0.40 mmol) and bis(4-bromophenyl)iodonium

tetrafluoroborate (252 mg, 0.48 mmol)[8]. After purification by column chromatography (silica; petroleum ether:ethyl acetate/93:7), the product was obtained as white solid (145 mg, 86%). **^{1}H NMR** (500 MHz, $CDCl_3$): δ 7.48 – 7.43 (m, 2H), 7.38 – 7.32 (m, 6H), 7.25 – 7.19 (m, 6H). **^{13}C NMR** (126 MHz, $CDCl_3$): δ 150.4 (d, *J* = 8.4 Hz), 136.9 (d, *J* = 5.5 Hz), 132.8 (d, *J* = 2.6 Hz), 130.1, 125.9, 124.6 (d, *J* = 3.8 Hz), 124.3 (d, *J* = 8.0 Hz), 120.6 (d, *J* = 4.8 Hz). **^{31}P NMR** (203 MHz, $CDCl_3$): δ 14.1. **FT-IR** (ATR): 1585, 1484, 1264, 1181, 1157, 926, 759 cm^{-1}. **HRMS** (ESI) *m/z*: $[M+H]^+$ Calcd for $C_{18}H_{15}BrO_3PS$ 420.9657 and 422.9637; Found 420.9664 and 422.9635. **Mp.**: 88-89 °C. The characterization data is consistent with that reported previously.[9]

S-(3,5-Difluorophenyl) *O,O*-diphenyl phosphorothioate (3e)

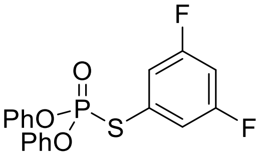

The title compound was prepared according to the General procedure B from triethylammonium *O,O*-diphenyl phosphorothioate (**1a**; 147 mg, 0.40 mmol) and bis(3,5-difluorophenyl)iodonium tetrafluoroborate (211 mg, 0.48 mmol)[10]. After purification by column chromatography (silica; petroleum ether:ethyl acetate/96:4), the product was obtained as white solid (130 mg, 86%). **^{1}H NMR** (500 MHz, $CDCl_3$): δ 7.40 – 7.43 (m, 4H), 7.28 – 7.21 (m, 6H), 7.06 – 7.00 (m, 2H), 6.86 (tq, *J* = 8.7, 2.2 Hz, 1H). **^{13}C NMR** (126 MHz, $CDCl_3$): δ 163.8 (dd, *J* = 12.7, 2.7 Hz), 161.8 (dd, *J* = 12.7, 2.7 Hz), 150.3 (d, *J* = 8.2 Hz), 130.1. 126.1, 120.6 (d, *J* = 5.1 Hz), 118.4 – 118.0 (m, 6H), 105.8 (td, *J* = 25.1, 2.9 Hz). **^{31}P NMR** (203 MHz, $CDCl_3$): δ 13.0. **^{19}F NMR** (376 MHz, $CDCl_3$): δ -107.6 – -107.8 (m). **FT-IR** (ATR): 3059, 1591, 1434, 1269, 1186, 947, 847, 760 cm^{-1}. **HRMS** (ESI) *m/z*: $[M+H]^+$ Calcd for $C_{18}H_{14}F_2O_3PS$ 379.0364; Found 379.0367. **Mp.**: 51-53 °C.

S-(3-Trifluoromethylphenyl) *O,O*-diphenyl phosphorothioate (3f)

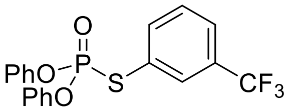

The title compound was prepared according to the General procedure B from triethylammonium *O,O*-diphenyl phosphorothioate (**1a**; 147 mg, 0.40 mmol) and bis(3-(trifluoromethyl)phenyl)iodonium tetrafluoroborate (242 mg, 0.48 mmol)[8]. After purification by column chromatography (silica; petroleum ether:ethyl acetate/90:10), the product was obtained as pale yellow liquid (141 mg, 86%). **^{1}H NMR** (500 MHz, $CDCl_3$): δ 7.71 (d, *J* = 7.7 Hz, 1H), 7.67 – 7.61 (m, 2H), 7.47 (t, *J* = 7.9 Hz, 1H), 7.39 – 7.33 (m, 4H), 7.26 – 7.17 (m, 6H). **^{13}C NMR** (126 MHz, $CDCl_3$): δ 150.3 (d, *J* = 8.3 Hz), 138.8 (d, *J* = 5.1 Hz), 132.3 – 132.0 (m), 130.10, 130.07, 126.8 (d, *J* = 7.5 Hz), 125.6 (quintet, *J* = 3.6 Hz), 126.0, 124.5 (q, *J* = 273.1 Hz), 120.5 (d, *J* = 5.4 Hz). **^{31}P NMR** (203 MHz, $CDCl_3$): δ 13.5. **^{19}F NMR** (376 MHz,

$CDCl_3$): δ -62.8. **FT-IR** (ATR): 1588, 1486, 1321, 1272, 1179, 1158, 938, 762 cm^{-1}. **HRMS** (ESI) *m/z*: $[M+H]^+$ Calcd for $C_{19}H_{15}F_3O_3PS$ 411.0426; Found 411.0429.

S-(3,5-Bis(trifluoromethyl)phenyl) O,O-diphenyl phosphorothioate (3g)

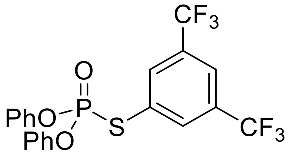

The title compound was prepared according to the General procedure B from triethylammonium *O,O*-diphenyl phosphorothioate (**1a**; 147 mg, 0.40 mmol) and bis(3,5-bis(trifluoromethyl)phenyl)iodonium tetrafluoroborate (307 mg, 0.48 mmol)[11]. After purification by column chromatography (silica; petroleum ether:ethyl acetate/98:2), the product was obtained as white solid (114 mg, 60%). **^{1}H NMR** (500 MHz, $CDCl_3$): δ 7.90 – 7.81 (m, 3H), 7.34 (t, J = 7.9 Hz, 4H), 7.29 – 7.19 (m, 6H). **^{13}C NMR** (126 MHz, $CDCl_3$): δ 150.1 (d, *J* = 8.1 Hz), 135.4, 132.8 (qd, *J* = 34.3, 2.5 Hz), 130.2, 129.0 (d, *J* = 7.4 Hz), 126.3, 123.7 – 123.5 (m), 122.7 (q, *J* = 272.9 Hz), 120.5 (d, *J* = 5.4 Hz). **^{31}P NMR** (203 MHz, $CDCl_3$): δ 12.0. **^{19}F NMR** (376 MHz, $CDCl_3$): δ -63.0. **FT-IR** (ATR): 1588, 1484, 1348, 1273, 1118, 935, 681 cm^{-1}. **HRMS** (ESI) *m/z*: $[M+H]^+$ Calcd for $C_{20}H_{14}F_6O_3PS$ 479.0300; Found 479.0301. **Mp.**: 51-52 °C.

S-(4-Nitrophenyl) O,O-diphenyl phosphorothioate (3h)

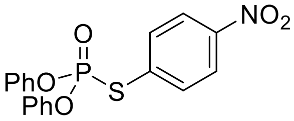

The title compound was prepared according to the General procedure B from triethylammonium *O,O*-diphenyl phosphorothioate (**1a**; 220 mg, 0.40 mmol) and (4-nitrophenyl)(phenyl)iodonium tetrafluoroborate (198 mg, 0.48 mmol)[12]. After purification by column chromatography (silica; petroleum ether:ethyl acetate/90:10), the product was obtained as yellow solid (130 mg, 84%). **^{1}H NMR** (500 MHz, $CDCl_3$): δ 8.16 (d, *J* = 8.8 Hz, 2H), 7.70 – 7.63 (m, 2H), 7.40 – 7.35 (m, 4H), 7.28 – 7.20 (m, 6H). **^{13}C NMR** (126 MHz, $CDCl_3$): δ 150.1 (d, *J* = 8.5 Hz), 148.4 (d, *J* = 2.8 Hz), 135.5 (d, *J* = 6.0 Hz), 134.4 (d, *J* = 7.2 Hz), 130.2, 126.2 (d, *J* = 1.4 Hz), 124.3 (d, *J* = 1.7 Hz), 120.6 (d, *J* = 5.3 Hz). **^{31}P NMR** (203 MHz, $CDCl_3$): δ 12.6. **FT-IR** (ATR): 1589, 1487, 1274, 1175, 1156, 932, 847, 741 cm^{-1}. **HRMS** (ESI) *m/z*: $[M+H]^+$ Calcd for $C_{18}H_{15}NO_5PS$ 388.0403; Found 388.0405. **Mp.**: 70-71 °C.

S-(3-Ethoxycarbonylphenyl) O,O-diphenyl phosphorothioate (3i)

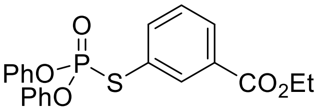

The title compound was prepared according to the General procedure B from triethylammonium *O,O*-diphenyl phosphorothioate (**1a**; 147 mg, 0.40 mmol) and bis(3-(ethoxycarbonyl)phenyl)iodonium tetrafluoroborate (246 mg, 0.48 mmol)[10]. After purification by column chromatography (silica; petroleum ether:ethyl acetate/85:15), the product was obtained as white solid (100 mg, 90%). **^{1}H NMR** (500 MHz, $CDCl_3$): δ 8.12 (q, *J* = 1.9 Hz, 1H), 8.09 – 8.04 (m, 1H), 7.72 – 7.66 (m, 1H), 7.42 (t, *J* = 7.8 Hz, 1H), 7.38 – 7.33 (m, 4H), 7.25 – 7.20 (m, 6H), 4.38 (q, *J* = 7.1 Hz, 2H), 1.39 (t, *J* = 7.1 Hz, 3H). **^{13}C NMR** (126 MHz, $CDCl_3$): δ 165.5, 150.4 (d, *J* = 8.3 Hz), 139.6 (d, *J* = 5.1 Hz), 136.4 (d, *J* = 5.7 Hz), 132.0 (d, *J* = 2.1 Hz), 130.9 (d, *J* = 3.2 Hz), 130.0, 129.7 (d, *J* = 2.3 Hz), 126.0 (d, *J* = 7.9 Hz), 125.9, 120.6 (d, *J* = 5.1 Hz), 61.6, 14.5. **^{31}P NMR** (203 MHz, $CDCl_3$): δ 14.2. **FT-IR** (ATR): 1719, 1266, 1127, 932, 774 cm^{-1}. **HRMS** (ESI) *m/z*: $[M+H]^+$ Calcd for $C_{21}H_{20}O_5PS$ 415.0764; Found 415.0768. **Mp.**: 83-84 °C. The characterization data is consistent with that reported previously.[7]

***S*-Mesityl *O,O*-diphenyl phosphorothioate (3j)**

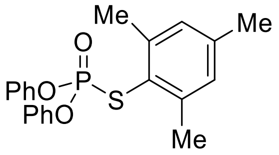

The title compound was prepared according to the General procedure B from triethylammonium *O,O*-diphenyl phosphorothioate (**1a**; 147 mg, 0.40 mmol) and dimesityliodonium tetrafluoroborate (217 mg, 0.48 mmol)[13]. After purification by column chromatography (silica; petroleum ether:ethyl acetate/93:7), the product was obtained as pale yellow liquid (154 mg, 100%). **^{1}H NMR** (500 MHz, $CDCl_3$): δ 7.33 – 7.29 (m, 4H), 7.21 – 7.13 (m, 6H), 6.94 (s, 2H), 2.41 (d, *J* = 1.5 Hz, 6H), 2.27 (d, *J* = 2.8 Hz, 3H). **^{13}C NMR** (126 MHz, $CDCl_3$): δ 150.8 (d, *J* = 9.9 Hz), 144.4 (d, *J* = 5.0 Hz), 140.2 (d, *J* = 4.3 Hz), 129.9, 129.8 (d, *J* = 3.6 Hz), 125.5, 120.7 (d, *J* = 4.7 Hz), 120.4 (d, *J* = 8.2 Hz), 22.5, 21.2. **^{31}P NMR** (203 MHz, $CDCl_3$): δ 16.4. **FT-IR** (ATR): 2931, 1589, 1487, 1270, 1180, 922, 764 cm^{-1}. **HRMS** (ESI) *m/z*: $[M+H]^+$ Calcd for $C_{21}H_{22}O_3PS$ 385.1022; Found 385.1026.

***S*-(4-Methoxyphenyl) *O,O*-diphenyl phosphorothioate (3k)**

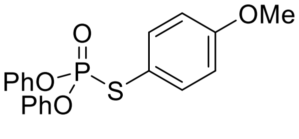

The title compound was prepared according to the General procedure B from triethylammonium *O,O*-diphenyl phosphorothioate (**1a**; 147 mg, 0.40 mmol) and bis(4-methoxyphenyl)iodonium tetrafluoroborate (205 mg, 0.48 mmol)[8]. After purification by column chromatography (silica; petroleum ether:ethyl acetate/90:10), the product was obtained as pale yellow liquid (93 mg, 62%). **^{1}H NMR** (500 MHz, $CDCl_3$): δ 7.41 – 7.32 (m, 6H), 7.24 – 7.18 (m, 6H), 6.88 – 6.84 (m, 2H), 3.81 (s, 3H). **^{13}C NMR** (126 MHz, $CDCl_3$): δ 161.1 (d, *J* = 2.9 Hz), 150.6 (d, *J* = 8.7

Hz), 137.2 (d, J = 5.0 Hz), 130.0, 125.6, 120.6 (d, J = 5.0 Hz), 115.2 (d, J = 2.7 Hz), 115.1 (d, J = 7.8 Hz), 55.6. **^{31}P NMR** (203 MHz, $CDCl_3$): δ 15.6. **FT-IR** (ATR): 1589, 1487, 1250, 1175, 1155, 922, 748 cm^{-1}. **HRMS** (ESI) *m/z*: $[M+H]^+$ Calcd for $C_{19}H_{18}O_4PS$ 373.0658; Found 373.0662. The characterization data is consistent with that reported previously.[9]

S-(4-Trifluoromethoxyphenyl) *O,O*-diphenyl phosphorothioate (3l)

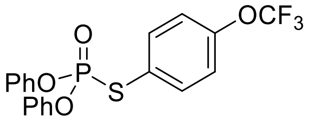

The title compound was prepared according to the General procedure B from triethylammonium *O,O*-diphenyl phosphorothioate (**1a**; 147 mg, 0.40 mmol) and bis(4-(trifluoromethoxy)phenyl)iodonium tetrafluoroborate (257 mg, 0.48 mmol)[10]. After purification by column chromatography (silica; petroleum ether:ethyl acetate/91:9), the product was obtained as pale yellow liquid (156 mg, 92%). **^{1}H NMR** (500 MHz, $CDCl_3$): δ 7.54 – 7.48 (m, 2H), 7.39 – 7.33 (m, 4H), 7.25 – 7.13 (m, 8H). **^{13}C NMR** (126 MHz, $CDCl_3$): δ 150.41 (d, J = 1.8 Hz), 150.40 (d, J = 8.6 Hz), 137.1 (d, J = 5.4 Hz), 130.1, 125.9, 123.7 (d, J = 7.8 Hz), 121.9, 120.6 (d, J = 4.8 Hz), 120.5 (d, J = 258.9 Hz). **^{31}P NMR** (203 MHz, $CDCl_3$): δ 14.2. **^{19}F NMR** (376 MHz, $CDCl_3$): δ -57.8. **FT-IR** (ATR): 1574, 1487, 1260, 1196, 1152, 393, 902, 764 cm^{-1}. **HRMS** (ESI) *m/z*: $[M+H]^+$ Calcd for $C_{19}H_{15}F_3O_4PS$ 427.0375; Found 427.0378.

S-(Naphth-2-yl) *O,O*-diphenyl phosphorothioate (3m)

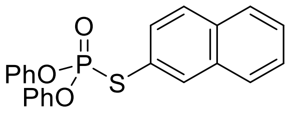

The title compound was prepared according to the General procedure B from triethylammonium *O,O*-diphenyl phosphorothioate (**1a**; 147 mg, 0.40 mmol) and bis(2-naphthyl)iodonium tetrafluoroborate (225 mg, 0.48 mmol)[14]. After purification by column chromatography (silica; petroleum ether:ethyl acetate/90:10), the product was obtained as yellow solid (150 mg, 96%). **^{1}H NMR** (500 MHz, $CDCl_3$): δ 7.96 (s, 1H), 7.84 (d, J = 7.6 Hz, 1H), 7.81 (d, J = 8.5 Hz, 1H), 7.74 (d, J = 8.1 Hz, 1H), 7.57 – 7.49 (m, 3H), 7.37 – 7.30 (m, 4H), 7.25 – 7.18 (m, 6H). **^{13}C NMR** (126 MHz, $CDCl_3$): δ 150.6 (d, J = 8.2 Hz), 135.8 (d, J = 7.1 Hz), 133.7 (d, J = 2.7 Hz), 133.5 (d, J = 2.5 Hz), 131.5 (d, J = 3.9 Hz), 130.0, 129.3 (d, J = 2.2 Hz), 128.0 (d, J = 10.0 Hz), 127.6 (d, J = 1.3 Hz), 127.0, 125.8 (d, J = 1.2 Hz), 122.2 (d, J = 8.3 Hz), 120.7 (d, J = 5.3 Hz). **^{31}P NMR** (203 MHz, $CDCl_3$): δ 14.9. **FT-IR** (ATR): 1487, 1260, 1178, 1159, 913, 764 cm^{-1}. **HRMS** (ESI) *m/z*: $[M+H]^+$ Calcd for $C_{22}H_{18}O_3PS$ 393.0709; Found 393.0708. **Mp.**: 62-63 °C.

S-(Naphth-1-yl) O,O-diphenyl phosphorothioate (3n)

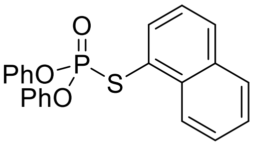

The title compound was prepared according to the General procedure B from triethylammonium *O,O*-diphenyl phosphorothioate (**1a**; 147 mg, 0.40 mmol) and bis(1-naphthyl)iodonium tetrafluoroborate (225 mg, 0.48 mmol)[8]. After purification by column chromatography (silica; petroleum ether:ethyl acetate/90:10), the product was obtained as yellow liquid (114 mg, 73%). **^{1}H NMR** (500 MHz, $CDCl_3$) δ 8.27 (d, *J* = 8.5 Hz, 1H), 7.92 (d, *J* = 8.5 Hz, 1H), 7.88 – 7.82 (m, 2H), 7.53 – 7.48 (m, 1H), 7.48 – 7.41 (m, 2H), 7.33 – 7.26 (m, 4H), 7.17 (t, *J* = 7.4 Hz, 2H), 7.14 – 7.08 (m, 4H). **^{13}C NMR** (126 MHz, $CDCl_3$): δ 150.7 (d, *J* = 8.5 Hz), 136.2 (d, *J* = 5.8 Hz), 134.9 (d, *J* = 4.0 Hz), 134.5 (d, *J* = 2.6 Hz), 131.1 (d, *J* = 3.8 Hz), 129.9, 128.6, 127.4, 126.7, 126.2, 125.8 (d, *J* = 3.7 Hz), 125.6 (d, *J* = 1.1 Hz), 122.4 (d, *J* = 8.5 Hz), 120.5 (d, *J* = 5.4 Hz). **^{31}P NMR** (203 MHz, $CDCl_3$): δ 14.7. **FT-IR** (ATR): 1487, 1260, 1178, 1159, 913, 764 cm^{-1}. **HRMS** (ESI) *m/z*: $[M+H]^+$ Calcd for $C_{22}H_{18}O_3PS$ 393.0709; Found 393.0709. The characterization data is consistent with that reported previously.[7]

O,O-Diethyl S-phenyl phosphorothioate (3o)

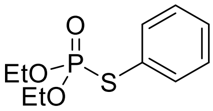

The title compound was prepared according to the General procedure B from triethylammonium *O,O*-diethyl phosphorothioate (108 mg, 0.40 mmol) and diphenyliodonium tetrafluoroborate (**2a**; 177 mg, 0.48 mmol)[8]. After purification by column chromatography (silica; petroleum ether:ethyl acetate/80:20), the product was obtained as pale yellow liquid (97 mg, 99%). **^{1}H NMR** (500 MHz, $CDCl_3$): δ 7.60 – 7.52 (m, 2H), 7.40 – 7.30 (m, 3H), 4.26 – 4.12 (m, 4H), 1.30 (td, *J* = 7.1, 0.7 Hz, 6H). **^{13}C NMR** (126 MHz, $CDCl_3$): δ 134.7 (d, *J* = 5.2 Hz), 129.5 (d, *J* = 2.4 Hz), 129.2 (d, *J* = 2.7 Hz), 126.8 (d, *J* = 7.4 Hz), 64.2 (d, *J* = 6.4 Hz), 16.2 (d, *J* = 7.2 Hz). **^{31}P NMR** (203 MHz, $CDCl_3$): δ 23.1. **FT-IR** (ATR): 1441, 1253, 1008, 970, 745 cm^{-1}. **HRMS** (ESI) *m/z*: $[M+H]^+$ Calcd for $C_{10}H_{16}O_3PS$ 247.0552; Found 247.0555. The characterization data is consistent with that reported previously.[15]
This product was also synthesized in a larger scale, from triethylammonium *O,O*-diethyl phosphorothioate (407 mg, 1.50 mmol) and diphenyliodonium tetrafluoroborate (**2a**; 662 mg, 1.80 mmol), affording 332 mg (90%) of **3o**.

5,5-Dimethyl-2-(phenylthio)-1,3,2-dioxaphosphinane 2-oxide (3p)

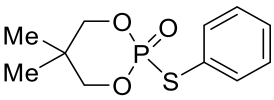

The title compound was prepared according to the General procedure B from triethylammonium 5,5-dimethyl-1,3,2-dioxaphosphinane-2-thiolate 2-oxide (113 mg, 0.40 mmol) and diphenyliodonium tetrafluoroborate (**2a**; 177 mg, 0.48 mmol)[8]. After purification by column chromatography (silica; petroleum ether:ethyl acetate/60:40), the product was obtained as white solid (91 mg, 88%). **^{1}H NMR** (500 MHz, $CDCl_3$): δ 7.68 – 7.58 (m, 2H), 7.40 – 7.32 (m, 3H), 4.20 (dd, *J* = 10.1, 3.7 Hz, 2H), 3.99 – 3.87 (m, 2H). 1.27 (s, 3H), 0.87 (s, 3H). **^{13}C NMR** (126 MHz, $CDCl_3$): δ 134.9 (d, *J* = 5.3 Hz), 129.7 (d, *J* = 2.2 Hz), 129.4 (d, *J* = 2.7 Hz), 124.9 (d, *J* = 6.4 Hz), 78.4 (d, *J* = 7.4 Hz), 32.7 (d, *J* = 6.7 Hz), 22.2, 20.6. **^{31}P NMR** (203 MHz, $CDCl_3$): δ 15.1. **FT-IR** (ATR): 1476, 1464, 1267, 1045, 990, 972, 782 cm^{-1}. **HRMS** (ESI) *m/z*: $[M+H]^+$ Calcd for $C_{11}H_{16}O_3PS$ 259.0552; Found 259.0553. **Mp.**: 120-121 °C.

***O,O*-Diallyl *S*-phenyl phosphorothioate (3q)**

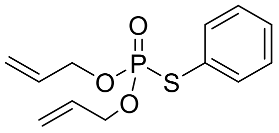

The title compound was prepared according to the General procedure B from triethylammonium *O,O*-diallyl phosphorothioate (118 mg, 0.40 mmol) and diphenyliodonium tetrafluoroborate (**2a**; 177 mg, 0.48 mmol)[8]. After purification by column chromatography (silica; petroleum ether:ethyl acetate/85:15), the product was obtained as colorless liquid (56 mg, 52%). **^{1}H NMR** (500 MHz, $CDCl_3$): δ 7.61 – 7.53 (m, 2H), 7.40 – 7.30 (m, 3H), 5.93 – 5.85 (m, 2H), 5.32 (dq, *J* = 17.1, 1.4 Hz, 2H), 5.23 (d, *J* = 10.3 Hz, 2H), 4.67 – 4.54 (m, 4H). **^{13}C NMR** (126 MHz, $CDCl_3$): δ 135.0 (d, *J* = 5.4 Hz), 132.2 (d, *J* = 7.5 Hz), 129.6 (d, *J* = 2.5 Hz), 129.4 (d, *J* = 2.7 Hz), 126.2 (d, *J* = 7.2 Hz), 118.8, 68.5 (d, *J* = 5.7 Hz). **^{31}P NMR** (203 MHz, $CDCl_3$): δ 23.9. **FT-IR** (ATR): 2917, 1259, 922, 796, 745 cm^{-1}. **HRMS** (ESI) *m/z*: $[M+H]^+$ Calcd for $C_{12}H_{16}O_3PS$ 271.0552; Found 271.0552.

(3a*R*,8a*R*)-2,2-Dimethyl-4,4,8,8-tetraphenyl-6-(phenylthio)tetrahydro-[1,3]dioxolo[4,5-e][1,3,2]dioxaphosphepine 6-oxide (3s)

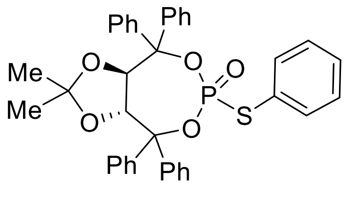

The title compound was prepared according to the General procedure B from triethylammonium (3a*R*,8a*R*)-2,2-dimethyl-4,4,8,8-tetraphenyltetrahydro-[1,3]dioxolo[4,5-e][1,3,2]dioxaphosphepine-6-thiolate 6-oxide (258 mg, 0.40 mmol) and diphenyliodonium tetrafluoroborate (**2a**; 177 mg, 0.48 mmol)[8]. After purification by column chromatography (silica; petroleum ether:ethyl acetate/83:17), the product was obtained as white solid (239 mg, 97%). **^{1}H NMR** (500 MHz, $CDCl_3$) δ 7.56 – 7.52 (m, 2H), 7.49 – 7.42 (m, 4H), 7.42 – 7.37 (m,

5H), 7.37 – 7.14 (m, 12H), 6.99 – 6.94 (m, 2H), 5.38 (d, *J* = 8.0 Hz, 1H), 5.09 (d, *J* = 8.0 Hz, 1H) 0.72 (s, 3H), 0.51 (s, 3H). **^{13}C NMR** (126 MHz, $CDCl_3$): δ 144.0 (d, *J* = 6.1 Hz), 143.3, 139.7 (d, *J* = 10.9 Hz), 139.6 (d, *J* = 3.6 Hz), 135.7 (d, *J* = 5.5 Hz), 129.9, 129.3 (d, *J* = 2.6 Hz), 129.2 (d, *J* = 3.1 Hz), 128.7, 128.5, 128.4 (d, *J* = 3.9 Hz), 127.87, 127.86 (d, *J* = 12.9 Hz), 127.3 (d, *J* = 2.4 Hz), 126.8, 126.1 (d, *J* = 8.6 Hz), 114.2, 92.0 (d, *J* = 13.6 Hz), 88.9 (d, *J* = 10.0 Hz), 79.3 (d, *J* = 1.4 Hz), 79.1 (d, *J* = 2.3 Hz), 27.0, 26.6. **^{31}P NMR** (203 MHz, $CDCl_3$): δ 15.4. **FT-IR** (ATR): 1255, 1014, 968, 744, 696 cm^{-1}. **HRMS** (ESI) *m/z*: $[M+H]^+$ Calcd for $C_{37}H_{34}O_5PS$ 621.1859; Found 621.1860. **Mp.**: 165-167 °C (decomposition). The characterization data is consistent with that reported previously.[7]

The NMR spectra (^{1}H, ^{13}C, ^{31}P) of the title compound contain a single set of signals indicating that no epimerization has occurred, demonstrating the stability of the stereocenters under the reaction conditions.

(*R*)-4-(Phenylthio)dinaphtho[2,1-d:1',2'-f][1,3,2]dioxaphosphepine 4-oxide ((*R*)-3t)

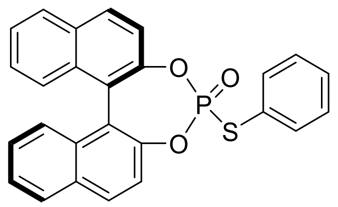

The title compound was prepared according to the General procedure B from triethylammonium (*R*)-dinaphtho[2,1-d:1',2'-f][1,3,2]dioxaphosphepine-4-thiolate 4-oxide (186 mg, 0.40 mmol) and diphenyliodonium tetrafluoroborate (**2a**; 177 mg, 0.48 mmol)[8]. After purification by column chromatography (silica; petroleum ether:ethyl acetate/75:25), the product was obtained as white solid (38 mg, 22%). **^{1}H NMR** (500 MHz, DMSO-d_6) δ 8.29 (dd, *J* = 13.2, 9.0 Hz, 2H), 8.15 (dd, *J* = 8.1, 3.2 Hz, 2H), 7.78 – 7.68 (m, 4H), 7.63 – 7.55 (m, 2H), 7.54 – 7.45 (m, 3H), 7.44 – 7.38 (m, 2H), 7.25 – 7.18 (m, 2H). **^{13}C NMR** (126 MHz, DMSO-d_6): δ 146. 4 (d, *J* = 11.7 Hz), 145.7 (d, *J* = 11.6 Hz), 135.5 (d, *J* = 5.3 Hz), 131.9 (d, *J* = 10.5 Hz), 131.6, 131.5, 130.2 (d, *J* = 2.8 Hz), 130.0 (d, *J* = 2.1 Hz), 128.8 (d, *J* = 8.6 Hz), 127.4 (d, *J* = 10.6 Hz), 126.3, 126.2 (d, *J* = 21.1 Hz), 122.8 (d, *J* = 6.3 Hz), 121.1 – 120.6 (m). **^{31}P NMR** (203 MHz, DMSO-d_6): δ 32.0. **FT-IR** (ATR): 1466, 1286, 1220, 1069, 945, 813, 745 cm^{-1}. **HRMS** (ESI) *m/z*: $[M+H]^+$ Calcd for $C_{26}H_{18}O_3PS$ 441.0709; Found 441.0709. **Mp.**: 80-83 °C (decomposition). **HPLC** (CHIRALART Cellulose-SC (equivalent to CHIRALPAK IC), hexanes/2-propanol = 9/1, flow rate = 1.0 mL/min, λ = 227 nm, 30 °C): t_R = 7.2 min (*rac*-**3t**: t_R = 7.2 min, 8.9 min).
The HPLC analysis shows that the compound is enantiopure, demonstrating the stability of the axial chirality under the reaction conditions.

==== Shimadzu LabSolutions Multi-Chromatogram ====

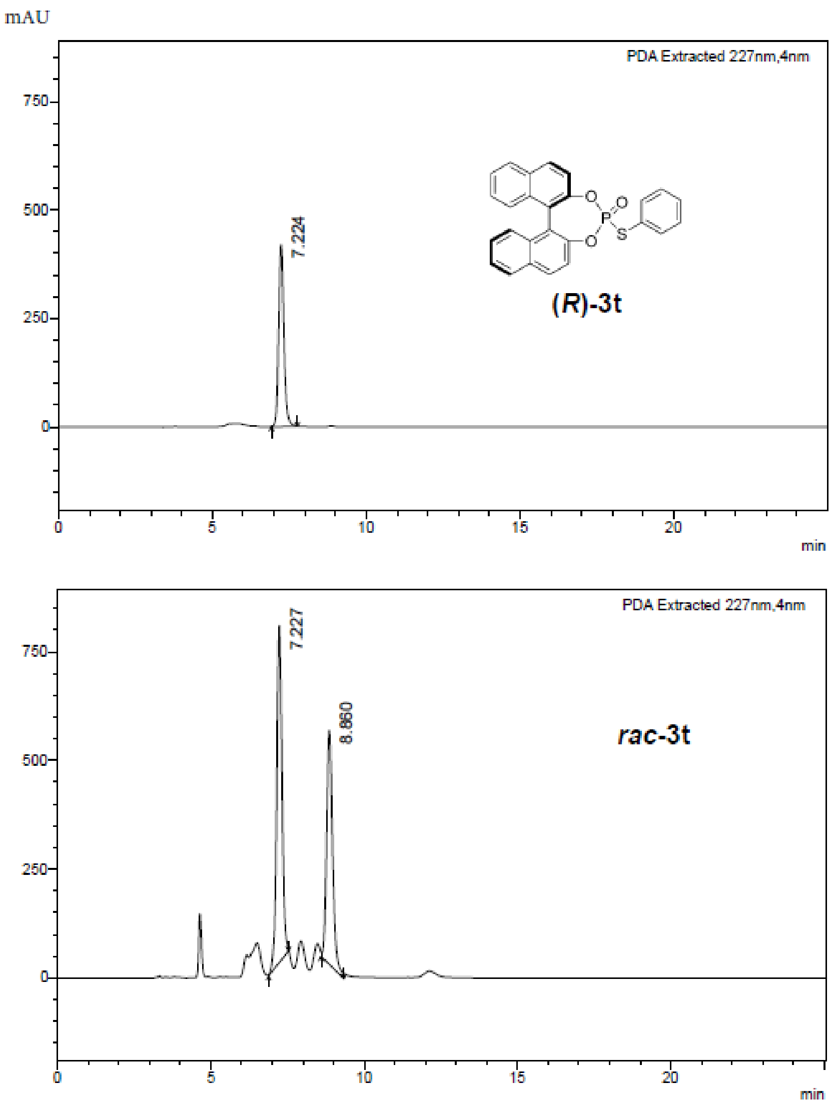

(R_P)-3′-*O*-(*tert*-Butyldimethylsilyl)thymidin-5′-yl 5′-*O*-dimethoxytritylthymidin-3′-yl *S*-phenyl phosphorothioate (R_P-3u)

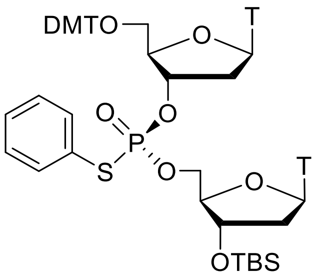

The title compound was prepared according to the General procedure B from triethylammonium (R_P)-3′-*O*-(*tert*-Butyldimethylsilyl)thymidin-5′-yl 5′-*O*-Dimethoxytritylthymidin-3′-yl phosphorothioate (108 mg, 0.10 mmol) and diphenyliodonium tetrafluoroborate (**2a**; 44.2 mg, 0.12 mmol)[8]. After purification by column chromatography (silica; petroleum ether:ethyl acetate/20:80), the product was obtained as white solid (53 mg, 50%). **^{1}H NMR** (500 MHz,

DMSO-d_6) δ 11.40 (s, 1H), 11.34 (s, 1H), 7.58 – 7.53 (m, 2H), 7.47 – 7.39 (m, 3H), 7.39 – 7.33 (m, 4H), 7.33 – 7.27 (m, 2H), 7.27 – 7.20 (m, 5H), 6.92 – 6.84 (m, 4H), 6.20 – 6.11 (m, 2H), 5.21 – 5.11 (m. 1H), 4.39 – 4.32 (m, 1H), 4.32 – 4.19 (m, 2H), 4.14 – 4.08 (m, 1H), 3.91 – 3.84 (m, 1H), 3.72 (s, 6H), 3.24 (dd, *J* = 27.5, 10.9 Hz, 2H), 2.49 – 2.42 (m, 1H), 2.32 (dd, *J* = 14.3, 6.0 Hz, 1H), 2.28 – 2.19 (m, 1H), 2.04 (ddd, *J* = 13.6, 6.4, 3.4 Hz, 1H), 1.72 (d, *J* = 1.0 Hz, 3H), 1.45 (d, *J* = 1.0 Hz, 3H), 0.84 (s, 9H), 0.04 (d, *J* = 6.0 Hz, 6H). **^{13}C NMR** (126 MHz, DMSO-d_6): δ 163.6 (d, *J* = 6.4 Hz), 158.2 (d, *J* = 1.2 Hz), 150.4 (d, *J* = 11.9 Hz), 144.5, 136.0, 135.5, 135.2, 135.1, 134.6 (d, *J* = 5.1 Hz), 129.73, 129.70 (d, *J* = 1.5 Hz), 127.8 (d, *J* = 35.1 Hz), 126.9, 124.9 (d, *J* = 7.2 Hz), 113.3, 109.9 (d, *J* = 16.5 Hz), 86.2, 84.2, 84.1, 83.7, 83.6 (d, *J* = 4.8 Hz), 78.6 (d, *J* = 5.5 Hz), 71.7, 67.0 (d, *J* = 5.9 Hz), 63.1, 59.8, 55.1, 25.6, 21.1, 17.6, 14.1, 12.1, 11.7, -4.9 (d, *J* = 15.4 Hz). **^{31}P NMR** (203 MHz, DMSO-d_6): δ 22.6. **FT-IR** (ATR): 1683, 1248, 1030, 991, 828 cm^{-1}. **HRMS** (ESI) *m/z*: [M+H]$^+$ Calcd for $C_{53}H_{64}N_4O_{13}PSSi$ 1055.3692; Found 1055.3688. **Mp.**: 115-117 °C (decomposition).

The NMR spectra (^{1}H, ^{13}C, ^{31}P) of the title compound contain a single set of signals indicating that no epimerization has occurred, demonstrating the stability of the stereocenters (both at carbons and at phosphorus) under the reaction conditions.

(S_P)-3′-*O*-(*tert*-Butyldimethylsilyl)thymidin-5′-yl 5′-*O*-dimethoxytritylthymidin-3′-yl *S*-phenyl phosphorothioate (S_P-3u)

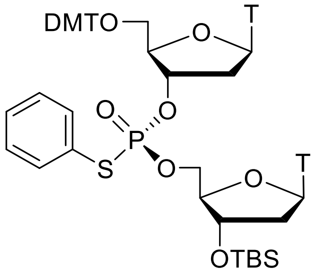

The title compound was prepared according to the General procedure B from triethylammonium (S_P)-3′-*O*-(*tert*-Butyldimethylsilyl)thymidin-5′-yl 5′-*O*-Dimethoxytritylthymidin-3′-yl phosphorothioate (108 mg, 0.10 mmol) and diphenyliodonium tetrafluoroborate (**2a**; 44.2 mg, 0.12 mmol)[8]. After purification by column chromatography (silica; petroleum ether:ethyl acetate/20:80), the product was obtained as white solid (55 mg, 52%). **^{1}H NMR** (500 MHz, DMSO-d_6) δ 11.39 (s, 1H), 11.32 (s, 1H), 7.49 – 7.44 (m, 3H), 7.44 – 7.28 (m, 8H), 7.27 – 7.20 (m, 5H), 6.91 – 6.86 (m, 4H), 6.21 – 6.15 (m, 1H), 6.12 (t, *J* = 6.9 Hz, 1H), 5.18 – 5.05 (m. 1H), 4.36 – 4.26 (m, 2H), 4.26 – 4.19 (m, 1H), 4.06 – 3.98 (m, 1H), 3.91 – 3.85 (m, 1H), 3.72 (s, 6H), 3.22 (dd, *J* = 26.5, 10.9 Hz, 2H), 2.53 – 2.36 (m, 2H), 2.26 – 2.16 (m, 1H), 2.10 – 2.02 (m, 1H), 1.68 (d, *J* = 0.9 Hz, 3H), 1.46 (d, *J* = 0.8 Hz, 3H), 0.85 (s, 9H), 0.06 (d, *J* = 2.8 Hz, 6H). **^{13}C NMR** (126 MHz, DMSO-d_6): δ 163.6 (d, *J* = 5.8 Hz), 158.2 (d, *J* = 1.8 Hz), 150.3 (d, *J* = 3.9 Hz), 144.5, 135.8, 135.5, 135.2, 135.0, 134.4 (d, *J* = 4.8 Hz), 129.9 – 129.5 (m), 127.8 (d, *J* = 39.3 Hz), 126.9, 124.9 (d, *J* = 7.3 Hz), 113.3, 109.9 (d, *J* = 20.0 Hz), 86.2, 84.02, 83.96, 83.7, 83.3 (d, *J* = 7.1 Hz), 78.7 (d, *J* = 6.1 Hz), 71.4, 66.8 (d, *J* = 6.3 Hz), 66.3, 63.2, 59.8, 55.0, 25.6, 20.8, 17.6, 14.1, 12.1, 11.7, -4.9 (d, *J* = 19.2 Hz). **^{31}P NMR** (203 MHz, DMSO-d_6): δ

22.3. **FT-IR** (ATR): 1680, 1249, 997, 830 cm^{-1}. **HRMS** (ESI) *m/z*: $[M+H]^+$ Calcd for $C_{53}H_{64}N_4O_{13}PSSi$ 1055.3692; Found 1055.3687. **Mp.**: 114-116 °C (decomposition).
The NMR spectra (^1H, ^{13}C, ^{31}P) of the title compound contain a single set of signals indicating that no epimerization has occurred, demonstrating the stability of the stereocenters (both at carbons and at phosphorus) under the reaction conditions.

Triphenyl phosphorodithioate (4a)

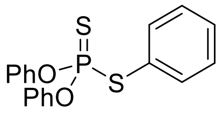

The title compound was prepared according to the General procedure B from triethylammonium *O,O*-diphenyl phosphorodithioate (153 mg, 0.40 mmol) and diphenyliodonium tetrafluoroborate (**2a**; 177 mg, 0.48 mmol)[8]. After purification by column chromatography (silica; petroleum ether:ethyl acetate/95:5), the product was obtained as pale yellow solid (116 mg, 81%).
^{1}H NMR (500 MHz, $CDCl_3$) δ 7.62 – 7.54 (m, 2H), 7.46 – 7.32 (m, 7H), 7.25 – 7.17 (m, 6H). **^{13}C NMR** (126 MHz, $CDCl_3$): δ 150.8 (d, *J* = 9.6 Hz), 135.6 (d, *J* = 5.3 Hz), 130.0 (d, *J* = 3.6 Hz), 129.8 (d, *J* = 1.5 Hz), 129.6 (d, *J* = 2.8 Hz), 127.6 (d, *J* = 8.2 Hz), 125.8 (d, *J* = 1.7 Hz), 121.6 (d, *J* = 4.9 Hz). **^{31}P NMR** (203 MHz, $CDCl_3$): δ 82.5. **FT-IR** (ATR): 1486, 1177, 1151, 913, 897, 791, 667 cm^{-1}. **HRMS** (ESI) *m/z*: $[M+H]^+$ Calcd for $C_{18}H_{16}O_2PS_2$ 359.0324; Found 359.0324. **Mp.**: 74-75 °C. The characterization data is consistent with that reported previously (only the Mp. 66-70 °C).[16]

***O,O*-Diethyl *S*-phenyl phosphorodithioate (4b)**

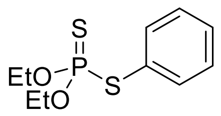

The title compound was prepared according to the General procedure B from triethylammonium *O,O*-diethyl phosphorodithioate (115 mg, 0.40 mmol) and diphenyliodonium tetrafluoroborate (**2a**; 177 mg, 0.48 mmol)[8]. After purification by column chromatography (silica; petroleum ether:ethyl acetate/95:5), the product was obtained as pale yellow liquid (94 mg, 90%). **^{1}H NMR** (500 MHz, $CDCl_3$): δ 7.55 – 7.48 (m, 2H), 7.41 – 7.33 (m, 3H), 4.30 – 4.13 (m, 4H), 1.31 (td, *J* = 7.1, 0.8 Hz, 6H). **^{13}C NMR** (126 MHz, $CDCl_3$): δ 134.9 (d, *J* = 5.1 Hz), 129.5 – 129.4 (m), 128.5 (d, *J* = 7.5 Hz), 64.4 (d, *J* = 5.6 Hz), 15.9 (d, *J* = 8.6 Hz). **^{31}P NMR** (203 MHz, $CDCl_3$): δ 88.3. **FT-IR** (ATR): 2924, 1440, 1009, 955, 797, 744, 648 cm^{-1}. **HRMS** (ESI) *m/z*: $[M+H]^+$ Calcd for $C_{10}H_{16}O_2PS_2$ 263.0324; Found 263.0323. The characterization data is consistent with that reported previously.[17]

***O,O,Se*-Triphenyl phosphoroselenoate (5a)**

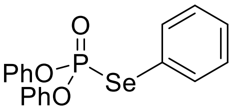

The title compound was prepared according to the General procedure B from triethylammonium *O,O*-diphenyl phosphoroselenoate (166 mg, 0.40 mmol) and diphenyliodonium tetrafluoroborate (**2a**; 177 mg, 0.48 mmol)[8]. After purification by column chromatography (silica; petroleum ether:ethyl acetate/90:10), the product was obtained as yellow solid (49 mg, 32%). **^{1}H NMR** (500 MHz, $CDCl_3$) δ 7.59 – 7.52 (m, 2H), 7.41 – 7.27 (m, 7H), 7.24 – 7.18 (m, 6H). **^{13}C NMR** (126 MHz, $CDCl_3$): δ 150.5 (d, *J* = 8.3 Hz), 136.4 (d, *J* = 5.0 Hz), 130.0, 129.8 (d, *J* = 2.6 Hz), 129.5 (d, *J* = 3.2 Hz), 125.8 (d, *J* = 1.3 Hz), 122.9 (d, *J* = 9.1 Hz), 120.8 (d, *J* = 5.2 Hz). **^{31}P NMR** (203 MHz, $CDCl_3$): δ 9.6. **FT-IR** (ATR): 1586, 1489, 1254, 1179, 1025, 924, 744 cm^{-1}. **HRMS** (ESI) *m/z*: $[M+H]^+$ Calcd for $C_{18}H_{16}O_3PSe$ 390.9997; Found 390.9998. **Mp.**: 51-53 °C. The characterization data is consistent with that reported previously.[18]

***O,O*-Diethyl *Se*-phenyl phosphoroselenoate (5b)**

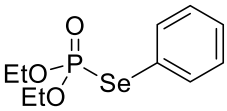

The title compound was prepared according to the General procedure B from triethylammonium *O,O*-diethyl phosphoroselenoate (128 mg, 0.40 mmol) and diphenyliodonium tetrafluoroborate (**2a**; 177 mg, 0.48 mmol)[8]. After purification by column chromatography (silica; petroleum ether:ethyl acetate/80:20), the product was obtained as yellow liquid (45 mg, 39%). **^{1}H NMR** (500 MHz, $CDCl_3$): δ 7.68 – 7.61 (m, 2H), 7.39 – 7.28 (m, 3H), 4.28 – 4.10 (m, 4H), 1.31 (t, *J* = 7.1 Hz, 6H). **^{13}C NMR** (126 MHz, $CDCl_3$): δ 135.7 (d, *J* = 4.6 Hz), 129.7 (d, *J* = 1.7 Hz), 129.0 (d, *J* = 2.6 Hz), 124.0 (d, *J* = 8.5 Hz), 64.0 (d, *J* = 6.1 Hz), 16.1 (d, *J* = 7.5 Hz). **^{31}P NMR** (203 MHz, $CDCl_3$): δ 18.1. **FT-IR** (ATR): 2992, 2922, 1439, 1248, 1007, 957, 739 cm^{-1}. **HRMS** (ESI) *m/z*: $[M+H]^+$ Calcd for $C_{10}H_{16}O_3PSe$ 294.9997; Found 294.9997. The characterization data is consistent with that reported previously.[19]

***O,S*-Diphenyl phenylphosphonothioate (6a)**

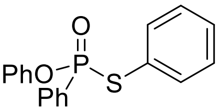

The title compound was prepared according to the General procedure B from triethylammonium *O*-phenyl phenylphosphonothioate (140 mg, 0.40 mmol) and diphenyliodonium tetrafluoroborate (**2a**; 177 mg, 0.48 mmol)[8]. After purification by column chromatography (silica; petroleum ether:ethyl acetate/82:18), the product was obtained as white solid (89 mg, 68%). **^{1}H NMR** (500 MHz, $CDCl_3$) δ 7.84 – 7.74 (m, 2H), 7.57 – 7.51 (m, 1H), 7.45 – 7.39 (m,

2H), 7.37 – 7.32 (m, 2H), 7.32 – 7.24 (m, 5H), 7.23 – 7.16 (m, 3H). **^{13}C NMR** (126 MHz, $CDCl_3$): δ 150.9 (d, *J* = 9.4 Hz), 136.0 (d, *J* = 4.5 Hz), 133.1 (d, *J* = 3.4 Hz), 131.9 (d, *J* = 10.9 Hz), 131.2 (d, *J* = 150.5 Hz), 129.9, 129.4 (d, *J* = 2.8 Hz), 129.3 (d, *J* = 2.5 Hz), 128.5 (d, *J* = 15.1 Hz), 125.9 (d, *J* = 5.6 Hz), 125.4, 121.0 (d, *J* = 4.8 Hz). **^{31}P NMR** (203 MHz, $CDCl_3$): δ 39.5. **FT-IR** (ATR): 1684, 1496, 1440, 1236, 922, 748, 687 cm^{-1}. **HRMS** (ESI) *m/z*: $[M+H]^+$ Calcd for $C_{18}H_{16}O_2PS$ 327.0603; Found 327.0604. **Mp.**: 58-59 °C.

***O*-Ethyl *S*-phenyl phenylphosphonothioate (6b)**

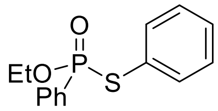

The title compound was prepared according to the General procedure B from triethylammonium *O*-ethyl phenylphosphonothioate (121 mg, 0.40 mmol) and diphenyliodonium tetrafluoroborate (**2a**; 177 mg, 0.48 mmol)[8]. After purification by column chromatography (silica; petroleum ether:ethyl acetate/73:27), the product was obtained as pale yellow liquid (64 mg, 58%). **^{1}H NMR** (500 MHz, $CDCl_3$): δ 7.69 – 7.60 (m, 2H), 7.52 – 7.47 (m, 1H), 7.40 – 7.39 (m, 2H), 7.32 – 7.26 (m, 3H), 7.24 – 7.17 (m, 2H), 4.42 – 4.28 (m, 2H), 1.40 (t, *J* = 7.1 Hz, 6H). **^{13}C NMR** (126 MHz, $CDCl_3$): δ 135.7 (d, *J* = 4.0 Hz), 132.7 (d, *J* = 3.4 Hz), 131.7 (d, *J* = 151.3 Hz), 131.6 (d, *J* = 10.3 Hz), 129.3 (d, *J* = 1.8 Hz), 129.1 (d, *J* = 2.7 Hz), 128.4 (d, *J* = 14.8 Hz), 126.8 (d, *J* = 5.6 Hz), 62.6 (d, *J* = 7.1 Hz), 16.5 (d, *J* = 6.5 Hz). **^{31}P NMR** (203 MHz, $CDCl_3$): δ 41.8. **FT-IR** (ATR): 1439, 1231, 1118, 1018, 952, 745, 690 cm^{-1}. **HRMS** (ESI) *m/z*: $[M+H]^+$ Calcd for $C_{14}H_{16}O_2PS$ 279.0603; Found 279.0604. The characterization data is consistent with that reported previously.[20]

***O*-((1*R*,2*S*,5*R*)-2-isopropyl-5-methylcyclohexyl) *S*-phenyl (*S*)-phenylphosphonothioate (*S*$_P$-6c)**

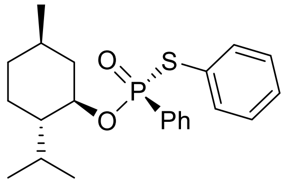

The title compound was prepared according to the General procedure B from triethylammonium *O*-((1*R*,2*S*,5*R*)-2-isopropyl-5-methylcyclohexyl) (*S*)-phenylphosphonothioate (97:3 dr at phosphorus; 62.0 mg, 0.15 mmol) and diphenyliodonium tetrafluoroborate (**2a**; 66.2 mg, 0.18 mmol)[8]. After purification by column chromatography (silica; petroleum ether:ethyl acetate/88:12), the product was obtained as pale yellow liquid (41 mg, 71%; 97:3 dr at phosphorus). **^{1}H NMR** (500 MHz, $CDCl_3$) δ 7.75 – 7.64 (m, 2H), 7.52 – 7.44 (m, 1H), 7.41 – 7.33 (m, 4H), 7.29 – 7.24 (m, 1H), 7.23 – 7.17 (m, 2H), 4.56 – 4.47 (m, 1H), 2.28 – 2.11 (m, 2H), 1.74 – 1.62 (m, 2H), 1.50 – 1.38 (m, 2H), 1.17 (q, *J* = 11.7 Hz, 1H), 1.06 (qd, *J* = 13.1, 3.2 Hz, 1H), 0.95 (d, *J* = 7.0 Hz, 3H), 0.88 (d, *J* = 6.8 Hz, 3H), 0.85 (d, *J* = 6.6 Hz, 3H), 0.84 – 0.80

(m, 1H). **^{13}C NMR** (126 MHz, $CDCl_3$): δ 135.6 (d, *J* = 4.4 Hz), 133.4 (d, *J* = 149.4 Hz), 132.4 (d, *J* = 3.0 Hz), 131.6 (d, *J* = 10.7 Hz), 129.2 (d, *J* = 2.2 Hz), 128.9 (d, *J* = 2.6 Hz), 128.3 (d, *J* = 15.2 Hz), 126.9 (d, *J* = 4.9 Hz), 80.0 (d, *J* = 8.2 Hz), 48.9 (d, *J* = 7.1 Hz), 43.6, 34.2, 31.8, 25.8, 23.1, 22.1, 21.4, 16.2. **^{31}P NMR** (203 MHz, $CDCl_3$): δ 39.9. **FT-IR** (ATR): 2921, 1456, 1439, 1233, 1117, 975, 690 cm^{-1}. **HRMS** (ESI) *m/z*: $[M+H]^+$ Calcd for $C_{22}H_{30}O_2PS$ 389.1699; Found 389.1691. The characterization data is consistent with that reported previously.[21]
The NMR spectra (^{1}H, ^{31}P) of the title compound contain two sets of signals in 97:3 ratio, corresponding exactly to the dr of the starting material, indicating that no epimerization has occurred, demonstrating the stability of the stereocenters (both at carbons and at phosphorus) under the reaction conditions.

***S*-Phenyl diphenylphosphinothioate (7a)**

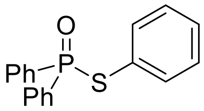

The title compound was prepared according to the General procedure B from triethylammonium diphenylphosphinothioate (134 mg, 0.40 mmol) and diphenyliodonium tetrafluoroborate (**2a**; 177 mg, 0.48 mmol)[8]. After purification by column chromatography (silica; petroleum ether:ethyl acetate/95:5), the product was obtained as white solid (38 mg, 31%). **^{1}H NMR** (500 MHz, $CDCl_3$): δ 7.92 – 7.77 (m, 4H), 7.55 – 7.37 (m, 8H), 7.27 – 7.14 (m, 3H). **^{13}C NMR** (126 MHz, $CDCl_3$): δ 135.5 (d, *J* = 3.8 Hz), 137.7 (d, *J* = 106.8 Hz), 132.5 (d, *J* = 2.8 Hz), 131.8 (d, *J* = 10.1 Hz), 129.3, 129.1 (d, *J* = 1.7 Hz), 128.7 (d, *J* = 13.2 Hz), 126.3 (d, *J* = 5.0 Hz). **^{31}P NMR** (203 MHz, $CDCl_3$): δ 41.5. **FT-IR** (ATR): 1438, 1192, 1097, 740, 691 cm^{-1}. **HRMS** (ESI) *m/z*: $[M+H]^+$ Calcd for $C_{18}H_{16}OPS$ 311.0654; Found 311.0654. **Mp.**: 90-92 °C. The characterization data is consistent with that reported previously.[20]

5. Computational Details

The calculations were carried out with Gaussian 16 software package[22] using B3LYP functional,[23] including the D3 dispersion correction with the BJ dumping.[24] Geometries were optimized with SMD solvation model (in 1,4-dioxane),[25] using Def2-SVP basis set,[26] including a pseudopotential for iodine[27].[28] For each stationary point a thorough conformational analysis was performed in order to locate the conformer with the lowest energy. This was done by identifying key rotatable bonds and manually building possible starting geometries for optimizations.

The identified lowest energy stationary points were then characterized by frequency calculations to confirm their character as minima (no imaginary frequencies) or transition states (a single imaginary frequency). The final free energies were obtained from single-point calculations on the optimized geometries with a larger Def2-QZVP basis set[26] (with SMD solvation) and were corrected for the thermodynamic effects at 100 °C using the quasi-harmonic approximation[29] (100 cm^{-1} cut-off) as implemented in GoodVibes program.[30]

Table S1. Energies and Energy Corrections of Stationary Points (in atomic units)

Stationary point	B3LYP-D3BJ/ Def2-SVP (1,4-dioxane) optimization	B3LYP-D3BJ/ Def2-QZVP (1,4-dioxane) single-point	Thermal correction to Gibbs free energy at 100 °C
**1b**	-1602.975209	-1602.704331	0.270878
**2a**	-1184.879847	-1184.739631	0.140216
**3a**	-1659.474866	-1659.245371	0.229495
**3O**	-1659.474889	-1659.245270	0.229619
**INT-O**	-2188.728742	-2190.397469	0.308658
**INT-S**	-2188.728478	-2190.398980	0.307500
**Me_3N-BF_4**	-599.130961	-599.022880	0.108081
**PhI**	-529.299466	-529.246901	0.052565
**Ph_2I·**	-760.724504	-760.597933	0.126571
**$(PhO)_2P(O)S$·**	-1427.913790	-1427.770084	0.143706
**TS1-O**	-2188.691159	-2188.385911	0.305248
**TS1-S**	-2188.692471	-2188.388697	0.303774
**TS2-O**	-2188.694516	-2188.388631	0.305885
**TS2-S**	-2188.697204	-2188.392661	0.304543

6. References

(1) Linghu, X.; Potnick, J. R.; Johnson, J. S. Metallophosphites as Umpolung Catalysts: The Enantioselective Cross Silyl Benzoin Reaction. *J. Am. Chem. Soc.* **2004**, *126*, 3070-3071.

(2) Seela, F.; Kretschmer, U. Diastereomerically Pure Rp and Sp Dinucleoside H-Phosphonates: The Stereochemical Course of Their Conversion into P-Methylphosphonates, Phosphorothioates, and [Oxygen-18] Chiral Phosphates. *J. Org. Chem.* **1991**, *56*, 3861-3869.

(3) Lai, C.; Xie, B.; Guo, X. Research on Hydroxyethyl Ammonium *O,O′*-Diphenyl Dithiophosphate: Synthesis, Characterization, Surface Activity and Corrosion Inhibition Performance. *Phosphorus Sulfur Silicon Relat. Elem.* **2020**, *195*, 107-114.

(4) Burn, A. J.; Dewan, S. K.; Gosney, I.; Tan, P. S. G. Phosphorus-31 Nuclear Magnetic Resonance Study of the Mechanism and Kinetics of the Hydrolysis of Zinc(II) *O,O*-Diethyl Dithiophosphate and Some Related Compounds. *J. Chem. Soc. Perkin Trans. 2* **1990**, 753.

(5) Unoh, Y.; Hirano, K.; Miura, M. Metal-Free Electrophilic Phosphination/Cyclization of Alkynes. *J. Am. Chem. Soc.* **2017**, *139*, 6106-6109.

(6) Wang, W.-M.; Liu, L.-J.; Zhao, C.-Q.; Han, L.-B. Diastereoselective Hydrolysis of Asymmetric P-Cl Species and Synthesis of Optically Pure (R_P)-(–)-Menthyl H-Phenylphosphinate: Diastereoselective Hydrolysis of Asymmetric P-Cl Species. *Eur. J. Org. Chem.* **2015**, 2342-2345.

(7) Chen, X.-Y.; Pu, M.; Cheng, H.-G.; Sperger, T.; Schoenebeck, F. Arylation of Axially Chiral Phosphorothioate Salts by Dinuclear Pd^{I} Catalysis. *Angew. Chem. Int. Ed.* **2019**, *58*, 11395-11399.

(8) Bielawski, M.; Aili, D.; Olofsson, B. Regiospecific One-Pot Synthesis of Diaryliodonium Tetrafluoroborates from Arylboronic Acids and Aryl Iodides. *J. Org. Chem.* **2008**, *73*, 4602-4607.

(9) Liu, Y.-C.; Lee, C.-F. N-Chlorosuccinimide-Promoted Synthesis of Thiophosphates from Thiols and Phosphonates under Mild Conditions. *Green Chem.* **2014**, *16*, 357-364.

(10) Beaud, R.; Phipps, R. J.; Gaunt, M. J. Enantioselective Cu-Catalyzed Arylation of Secondary Phosphine Oxides with Diaryliodonium Salts toward the Synthesis of P-Chiral Phosphines. *J. Am. Chem. Soc.* **2016**, *138*, 13183-13186.

(11) Ghosh, M. K.; Rzymkowski, J.; Kalek, M. Transition-Metal-Free Aryl–Aryl Cross-Coupling: C−H Arylation of 2-Naphthols with Diaryliodonium Salts. *Chem. Eur. J.* **2019**, *25*, 9619-9623.

(12) Reitti, M.; Gurubrahamam, R.; Walther, M.; Lindstedt, E.; Olofsson, B. Synthesis of Phenols and Aryl Silyl Ethers via Arylation of Complementary Hydroxide Surrogates. *Org. Lett.* **2018**, *20*, 1785-1788.

(13) Lindstedt, E.; Stridfeldt, E.; Olofsson, B. Mild Synthesis of Sterically Congested Alkyl Aryl Ethers. *Org. Lett.* **2016**, *18*, 4234-4237.

(14) Gao, H.; Xu, Q.-L.; Keene, C.; Kürti, L. Scalable, Transition-Metal-Free Direct Oxime *O* -Arylation: Rapid Access to *O*-Arylhydroxylamines and Substituted Benzo[*b*]furans. *Chem. Eur. J.* **2014**, *20*, 8883-8887.

(15) Handoko; Benslimane, Z.; Arora, P. S. Diselenide-Mediated Catalytic Functionalization of Hydrophosphoryl Compounds. *Org. Lett.* **2020**, *22*, 5811-5816.

(16) Miller, B. Phosphorothioates. II. The Effect of the Nucleophilicity of the Attacking Anion upon Rates of Displacement on *O,O*-Diphenyl Phosphorochloridothioate. *J. Am. Chem. Soc.* **1962**, *84*, 403-409.

(17) Shen, B.-R.; Annamalai, P.; Wang, S.-F.; Bai, R.; Lee, C.-F. Blue LED-Promoted Syntheses of Phosphorothioates and Phosphorodithioates. *J. Org. Chem.* **2022**, *87*, 8858-8870.

(18) Bhunia, S. K.; Das, P.; Jana, R. Atom-Economical Selenation of Electron-Rich Arenes and Phosphonates with Molecular Oxygen at Room Temperature. *Org. Biomol. Chem.* **2018**, *16*, 9243-9250.

(19) Mondal, M.; Saha, A. Benign Synthesis of Thiophosphates, Thiophosphinates and Selenophosphates in Neat Condition Using *N*-Chalcogenoimides as the Source of Electrophilic Sulfur/Selenium. *Tetrahedron Lett.* **2019**, *60*, 150965.

(20) Wang, J.; Huang, X.; Ni, Z.; Wang, S.; Wu, J.; Pan, Y. TBPB-Promoted Metal-Free Synthesis of Thiophosphinate/phosphonothioate by Direct P–S Bond Coupling. *Green Chem.* **2015**, *17*, 314-319.

(21) Wang, W.-M.; Liu, L.-J.; Yao, L.; Meng, F.-J.; Sun, Y.-M.; Zhao, C.-Q.; Xu, Q.; Han, L.-B. Stereospecific Preparations of P-Stereogenic Phosphonothioates and Phosphonoselenoates. *J. Org. Chem.* **2016**, *81*, 6843-6847.

(22) Gaussian 16, Revision A.03, M. J. Frisch, G. W. Trucks, H. B. Schlegel, G. E. Scuseria, M. A. Robb, J. R. Cheeseman, G. Scalmani, V. Barone, G. A. Petersson, H. Nakatsuji, X.

Li, M. Caricato, A. V. Marenich, J. Bloino, B. G. Janesko, R. Gomperts, B. Mennucci, H. P. Hratchian, J. V. Ortiz, A. F. Izmaylov, J. L. Sonnenberg, D. Williams-Young, F. Ding, F. Lipparini, F. Egidi, J. Goings, B. Peng, A. Petrone, T. Henderson, D. Ranasinghe, V. G. Zakrzewski, J. Gao, N. Rega, G. Zheng, W. Liang, M. Hada, M. Ehara, K. Toyota, R. Fukuda, J. Hasegawa, M. Ishida, T. Nakajima, Y. Honda, O. Kitao, H. Nakai, T. Vreven, K. Throssell, J. A. Montgomery, Jr., J. E. Peralta, F. Ogliaro, M. J. Bearpark, J. J. Heyd, E. N. Brothers, K. N. Kudin, V. N. Staroverov, T. A. Keith, R. Kobayashi, J. Normand, K. Raghavachari, A. P. Rendell, J. C. Burant, S. S. Iyengar, J. Tomasi, M. Cossi, J. M. Millam, M. Klene, C. Adamo, R. Cammi, J. W. Ochterski, R. L. Martin, K. Morokuma, O. Farkas, J. B. Foresman, and D. J. Fox, Gaussian, Inc., Wallingford CT, 2016.

(23) Lee, C.; Yang, W.; Parr, R. G. Development of the Colle-Salvetti Correlation-Energy Formula into a Functional of the Electron Density. *Phys. Rev. B* **1988**, *37*, 785-789; (b) Becke, A. D. Density-Functional Exchange-Energy Approximation with Correct Asymptotic Behavior. *Phys. Rev. A* **1988**, *38*, 3098-3100; (c) Becke, A. D. Density-Functional Thermochemistry. I. The Effect of the Exchange-Only Gradient Correction. *J. Chem. Phys.* **1992**, *96*, 2155-2160; (d) Becke, A. D. Density-Functional Thermochemistry. II. The Effect of the Perdew–Wang Generalized-Gradient Correlation Correction. *J. Chem. Phys.* **1992**, *97*, 9173-9177; (e) Becke, A. D. Density-Functional Thermochemistry. III. The Role of Exact Exchange. *J. Chem. Phys.* **1993**, *98*, 5648-5652.

(24) (a) Grimme, S.; Antony, J.; Ehrlich, S.; Krieg, H. A Consistent and Accurate *ab initio* Parametrization of Density Functional Dispersion Correction (DFT-D) for the 94 Elements H-Pu. *J. Chem. Phys.* **2010**, *132*, 154104; (b) Grimme, S.; Ehrlich, S.; Goerigk, L. Effect of the Damping Function in Dispersion Corrected Density Functional Theory. *J. Comput. Chem.* **2011**, *32*, 1456-1465.

(25) Marenich, A. V.; Cramer, C. J.; Truhlar, D. G. Universal Solvation Model Based on Solute Electron Density and on a Continuum Model of the Solvent Defined by the Bulk Dielectric Constant and Atomic Surface Tensions. *J. Phys. Chem. B* **2009**, *113*, 6378-6396.

(26) Weigend, F.; Ahlrichs, R. Balanced Basis Sets of Split Valence, Triple Zeta Valence and Quadruple Zeta Valence Quality for H to Rn: Design and Assessment of Accuracy. *Phys. Chem. Chem. Phys.* **2005**, *7*, 3297.

(27) Peterson, K. A.; Figgen, D.; Goll, E.; Stoll, H.; Dolg, M. Systematically Convergent Basis Sets with Relativistic Pseudopotentials. II. Small-Core Pseudopotentials and Correlation Consistent Basis Sets for the Post-d Group 16-18 Elements. *J. Chem. Phys.* **2003**, *119*, 11113-11123.

(28) Pritchard, B. P.; Altarawy, D.; Didier, B.; Gibsom, T. D.; Windus, T. L. A New Basis Set Exchange: An Open, Up-to-date Resource for the Molecular Sciences Community. *J. Chem. Inf. Model.* **2019**, *59*, 4814-4820.

(29) Grimme, S. Supramolecular Binding Thermodynamics by Dispersion-Corrected Density Functional Theory. *Chem. Eur. J.* **2012**, *18*, 9955-9964.

(30) GoodVibes, verison 2.0.1, Funes-Ardoiz, I.; Paton, R. S., 2017 (DOI: 10.5281/zenodo.884527).

7. Structures and Cartesian Coordinates of Stationary Points

1b

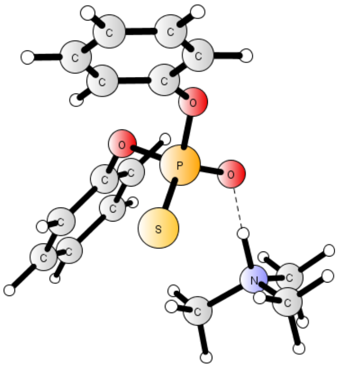

P	-0.39800400	-0.40408900	0.25662400
O	-1.82852300	-0.51596300	1.05239300
O	-0.10516700	1.21927100	0.55616500
C	1.16489500	1.70260000	0.35046800
C	2.10834500	1.62436800	1.38180200
C	1.50588700	2.26421700	-0.88438000
C	3.39728800	2.12261700	1.17507500
H	1.81412100	1.16919900	2.32820300
C	2.79834800	2.75733700	-1.08189200
H	0.75515100	2.29375300	-1.67489400
C	3.74743100	2.68784100	-0.05660500
H	4.13182800	2.07168100	1.98310100
H	3.06483800	3.19882300	-2.04550700
H	4.75536600	3.07894700	-0.21425400
C	-3.05644400	-0.14356000	0.55739300
C	-4.12226400	-1.02587900	0.75532100
C	-3.25738800	1.08123800	-0.08826900
C	-5.39864500	-0.67845000	0.30882800
H	-3.93192100	-1.97595300	1.25801500
C	-4.53740500	1.41399500	-0.53691500
H	-2.41732500	1.76134300	-0.22797000
C	-5.61143200	0.54074500	-0.34167200
H	-6.23059200	-1.36968500	0.46583300
H	-4.69398900	2.36982000	-1.04335600
H	-6.60984700	0.80909200	-0.69482200
H	1.79795600	-1.66411500	0.44433400
N	2.67548700	-2.08418300	-0.07825800
C	3.14828800	-1.05971800	-1.03982500
H	2.31433900	-0.79522700	-1.70142600
H	3.46612000	-0.16814000	-0.48614300
H	3.99215500	-1.45910400	-1.62174900
C	3.68899700	-2.39587400	0.94944100
H	3.26010500	-3.09717300	1.67715600
H	4.57793900	-2.84446700	0.48136700
H	3.97037700	-1.46803700	1.46443200
C	2.16227600	-3.29332800	-0.76154800
H	2.96467300	-3.76764300	-1.34560600
H	1.78548900	-3.99485800	-0.00603400
H	1.33349000	-2.99089100	-1.41524700
S	-0.53613900	-0.76204600	-1.67305500
O	0.59500700	-1.19117100	1.10626200

2a

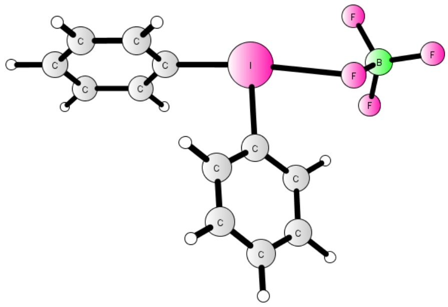

I	0.01492800	-0.72626700	-0.31593400
C	-2.08252700	-0.79351100	0.05676700
C	-2.94898800	-1.02074100	-1.01520600
C	-2.54676000	-0.60950600	1.36187900
C	-4.32471100	-1.05905300	-0.76576500
H	-2.56558200	-1.16251500	-2.02736600
C	-3.92432100	-0.64467000	1.59002400
H	-1.85188700	-0.43649900	2.18560600
C	-4.80953100	-0.86915800	0.53068300
H	-5.01669100	-1.23613000	-1.59223700
H	-4.30500100	-0.49958200	2.60358200
H	-5.88515300	-0.89764600	0.71870600
C	0.12147900	1.41901200	-0.15824400
C	-0.91801000	2.17405200	-0.69354000
C	1.23002900	1.96606600	0.47994500
C	-0.83651800	3.56489800	-0.57463400
H	-1.77340200	1.70833200	-1.18468300
C	1.28479000	3.36111400	0.57990300
H	2.02655500	1.33751100	0.87955400
C	0.26116700	4.15558700	0.05787500
H	-1.63734300	4.18345800	-0.98631000
H	2.14283300	3.82061900	1.07569100
H	0.31851000	5.24296500	0.14342700
B	3.32354500	-1.08118600	0.17070700
F	2.55064500	-0.35262900	-0.81864800
F	3.23404400	-0.36050100	1.36944700
F	2.71352700	-2.32910400	0.31403400
F	4.62419500	-1.18298700	-0.26740000

3a

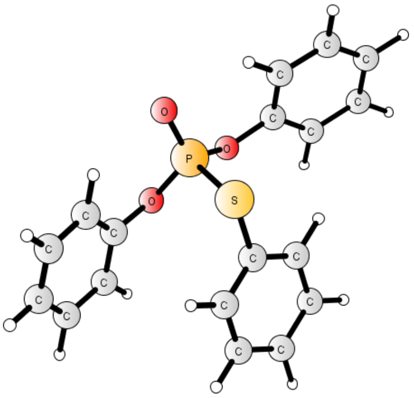

P	-0.21382200	-1.29838300	-0.00424800
O	-0.34773700	-2.72789900	0.37282300

O	-1.37726500	-0.67465800	-0.95233800
O	0.99296500	-0.89444700	-1.01188500
S	-0.04610400	-0.09920500	1.71407000
C	2.34101500	-0.97962900	-0.70145800
C	2.85210300	-1.95769700	0.15509100
C	3.17506300	-0.03302900	-1.30006900
C	4.22436500	-1.96805800	0.42267800
H	2.18640400	-2.70035700	0.59650600
C	4.54378500	-0.06326100	-1.03138700
H	2.73566100	0.71865000	-1.95722700
C	5.07251600	-1.02610200	-0.16478300
H	4.62969400	-2.72811100	1.09480100
H	5.19992900	0.67523900	-1.49826500
H	6.14368100	-1.04410100	0.04804000
C	-2.71783400	-0.58967400	-0.61239100
C	-3.43607500	0.45618200	-1.19776300
C	-3.33724100	-1.49601400	0.25251900
C	-4.79408100	0.59898100	-0.90884600
H	-2.91903800	1.14063000	-1.87231500
C	-4.69603600	-1.33396000	0.53781800
H	-2.76619300	-2.31913600	0.68294700
C	-5.42782600	-0.29180700	-0.03646200
H	-5.35854900	1.41425500	-1.36774200
H	-5.18413200	-2.03882500	1.21508600
H	-6.48968900	-0.17560600	0.19127700
C	0.30797800	1.49782900	0.96654900
C	1.63168300	1.95198800	0.92397800
C	-0.73339100	2.28829300	0.46499100
C	1.91318200	3.19778200	0.35867000
H	2.43404700	1.32865500	1.32042800
C	-0.44089000	3.52884700	-0.10454300
H	-1.76299100	1.93247200	0.51955000
C	0.88043500	3.98342900	-0.16038400
H	2.94636300	3.55076800	0.31993900
H	-1.25120300	4.14452800	-0.50222200
H	1.10480500	4.95539400	-0.60625700

3O

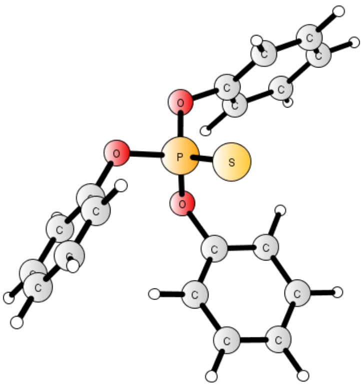

P	-0.12822900	-0.92468100	0.21579600
O	-1.36454100	-1.72131400	-0.46871800
O	0.04797700	0.35676400	-0.78158600
O	1.02162900	-1.93928300	-0.31912100
S	-0.21898900	-0.51635000	2.09208100
C	2.35878900	-1.55093900	-0.30151100
C	2.93871800	-1.11301900	-1.49277600
C	3.08832300	-1.60864900	0.88649200
C	4.28170300	-0.72842300	-1.49204400
H	2.33213300	-1.07303500	-2.39859000
C	4.42926200	-1.21592800	0.87479600
H	2.60175700	-1.95027300	1.80035200
C	5.02726300	-0.77507900	-0.30962200
H	4.74476000	-0.38618600	-2.42050800
H	5.00840400	-1.25558500	1.80029200
H	6.07608200	-0.46983700	-0.31205000
C	0.24629700	1.66525900	-0.35268300
C	-0.86143500	2.48507700	-0.14168800
C	1.54725300	2.13290500	-0.17021800
C	-0.65656300	3.81090300	0.24726300
H	-1.86500200	2.07833000	-0.27746300
C	1.73841000	3.46080600	0.21891500
H	2.39019000	1.45938200	-0.33193400
C	0.64048400	4.30110100	0.42717200
H	-1.51784600	4.46167700	0.41565500
H	2.75367800	3.83756600	0.36366700
H	0.79561900	5.33797200	0.73403500
C	-2.65822100	-1.21154900	-0.39410900
C	-3.47284200	-1.55436500	0.68488900
C	-3.11467800	-0.38127400	-1.41895400
C	-4.77750600	-1.05703700	0.73052200
H	-3.07812500	-2.19808600	1.47166700
C	-4.42187500	0.10917700	-1.36134300
H	-2.44567300	-0.13316100	-2.24398000
C	-5.25341900	-0.22589900	-0.28824600
H	-5.42418200	-1.32050500	1.57071000
H	-4.79027300	0.75600900	-2.16111400
H	-6.27456200	0.15981300	-0.24669400

INT-O

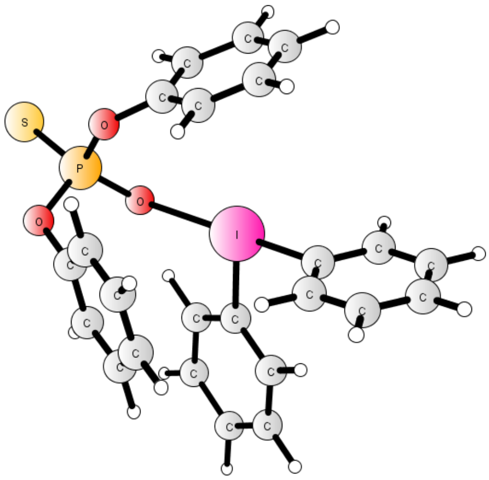

I	0.56614100	0.29317000	-0.42104400
C	2.52764500	1.03445500	0.00128700
C	3.24940600	1.68811100	-1.00109700
C	3.03200000	0.89404300	1.29741700
C	4.51330400	2.20045600	-0.69569500
H	2.83589700	1.79665400	-2.00568700
C	4.29719000	1.41348700	1.58828800
H	2.44944100	0.38090900	2.06523100
C	5.03522400	2.06298800	0.59448300
H	5.09000400	2.71133400	-1.47035300
H	4.70498700	1.30908800	2.59649200
H	6.02293300	2.46759800	0.82719400
C	1.26501500	-1.61912300	-1.14724700
C	2.57586400	-2.01030100	-0.89051800
C	0.35135200	-2.41786100	-1.82922000
C	2.98453400	-3.26965900	-1.34339500
H	3.27231100	-1.36925700	-0.35031900
C	0.78558700	-3.67252400	-2.27036400

H	-0.67385300	-2.07664000	-1.98012200
C	2.09413100	-4.09947200	-2.02952000
H	4.00895400	-3.59739400	-1.15112700
H	0.08449200	-4.31728600	-2.80564400
H	2.42157000	-5.08176400	-2.37744500
P	-3.04233200	-0.42957400	-0.11055200
O	-2.86061100	1.05248100	0.68701200
O	-2.90035500	-1.38312300	1.24810300
C	-2.20130600	2.10421000	0.12254400
C	-1.23029000	2.76065900	0.89448800
C	-2.43913600	2.51164400	-1.19759800
C	-0.49158200	3.81147900	0.34130900
H	-1.06146900	2.42752200	1.92011100
C	-1.69033200	3.55713600	-1.74051100
H	-3.20100200	1.99652400	-1.78484500
C	-0.71164600	4.20801900	-0.98115400
H	0.26584000	4.31448100	0.94775800
H	-1.87513800	3.86513000	-2.77270600
H	-0.12925700	5.02350900	-1.41573900
C	-1.67542200	-1.51971700	1.85609800
C	-1.28701200	-0.62309400	2.85841400
C	-0.82094300	-2.55468000	1.46220900
C	-0.03186100	-0.76032800	3.45621800
H	-1.97534100	0.17166500	3.14677700
C	0.43353400	-2.68226100	2.06144000
H	-1.14556800	-3.23695300	0.67620500
C	0.83550100	-1.78349800	3.05522400
H	0.27012300	-0.06244900	4.24150700
H	1.10325800	-3.48347700	1.74058100
H	1.81606700	-1.88920900	3.52557700
O	-1.76832200	-0.59879300	-0.94597500
S	-4.80156000	-0.65087000	-0.89322200

INT-S

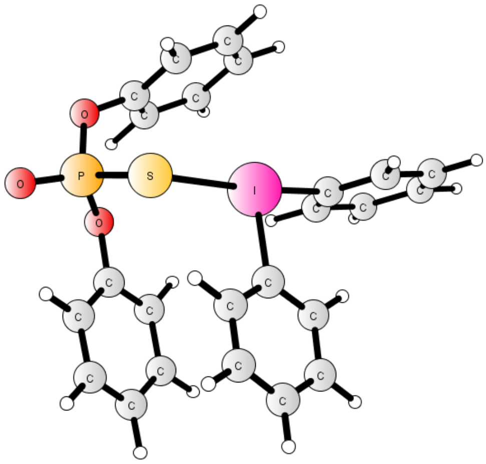

I	0.83607000	-0.04276200	-0.81213300
C	2.73639800	0.62868000	-0.01722100
C	2.91240800	0.64614500	1.36819900
C	3.77403900	0.95071700	-0.89398400
C	4.15866600	1.01209600	1.88494900
H	2.09310600	0.37813700	2.03798400
C	5.01642300	1.31334200	-0.36363900
H	3.62017000	0.92619300	-1.97492700
C	5.20712100	1.34427700	1.02098000
H	4.31040800	1.03392900	2.96674100
H	5.83710800	1.57101000	-1.03736800
H	6.17995000	1.62678000	1.42988400
C	0.01396500	1.97717300	-0.85330600
C	0.79406100	3.03219400	-0.38947800
C	-1.28394100	2.14457400	-1.32140700
C	0.23455500	4.31523200	-0.39909000
H	1.80836500	2.88328800	-0.02226600
C	-1.81897200	3.43630000	-1.32107600
H	-1.87870100	1.29178300	-1.65299300
C	-1.06592300	4.51961700	-0.86385500
H	0.83108900	5.15583400	-0.03656800
H	-2.84158500	3.58198500	-1.67501700
H	-1.49496100	5.52406400	-0.86429400
P	-2.45006500	-2.09823500	-0.22891800
O	-2.03759500	-1.14422300	1.08066700
O	-1.48301800	-3.37572300	0.19384300
C	-2.46690100	0.14300100	1.26703400
C	-1.60577300	1.00982100	1.95000400
C	-3.71600700	0.59306900	0.82036500
C	-1.98811100	2.33186100	2.17778200
H	-0.64097900	0.63003200	2.29050800
C	-4.08382000	1.92073100	1.05025000
H	-4.38455500	-0.10175800	0.31058600
C	-3.22624300	2.79550600	1.72360800
H	-1.30781300	3.00731000	2.70191700
H	-5.05725200	2.27138800	0.69801000
H	-3.52008200	3.83369100	1.89277900
C	-0.12306400	-3.27007100	0.34050400
C	0.41762900	-2.94897300	1.59118500
C	0.71410400	-3.48130700	-0.76098800
C	1.80108500	-2.82572200	1.73315200
H	-0.26000000	-2.78501500	2.42935500
C	2.09916700	-3.34764900	-0.60955400
H	0.26589100	-3.72455500	-1.72445000
C	2.64622300	-3.01348600	0.63363000
H	2.22303100	-2.57300500	2.70898600
H	2.75231800	-3.50550800	-1.47135800
H	3.72664800	-2.90082600	0.74623200
S	-1.72381900	-1.20696800	-1.89943400
O	-3.86992900	-2.53457100	-0.21128900

Me_3N-BF_4

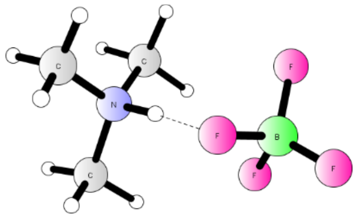

H	0.65244500	0.01576300	0.53559500
N	1.59092900	0.00115400	0.05709200
C	1.46325600	-0.87756700	-1.13725900
H	1.14502400	-1.87402200	-0.80981600
H	0.69509800	-0.45483000	-1.79260200
H	2.43361700	-0.93039900	-1.64967100
C	1.87094100	1.41158400	-0.31887900

H	1.90782300	2.01992100	0.59382600
H	2.83268900	1.46506200	-0.84753500
H	1.05164000	1.76111100	-0.95664700
C	2.56372400	-0.52958800	1.04298600
H	3.56873300	-0.53946300	0.59904800
H	2.55505900	0.11012700	1.93464800
H	2.26757500	-1.54913500	1.32073900
B	-1.64938400	-0.00889400	0.05719100
F	-0.77719300	0.15560800	1.21102000
F	-1.56181800	-1.34009800	-0.33487000
F	-2.92495400	0.36600500	0.39395300
F	-1.11234600	0.81689400	-0.95168800

PhI

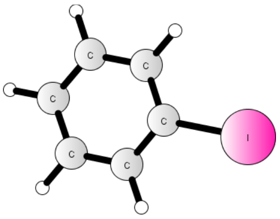

I	-1.56385800	0.00000800	0.00003600
C	0.57092500	-0.00001700	-0.00023200
C	1.25882900	-1.21902700	-0.00020100
C	1.25873800	1.21884300	-0.00010400
C	2.65882400	-1.21022700	0.00012600
H	0.71523500	-2.16588800	-0.00024800
C	2.65887900	1.21028900	0.00005800
H	0.71514400	2.16571800	-0.00059400
C	3.36056600	0.00007600	0.00008500
H	3.20005500	-2.16026600	0.00001100
H	3.19990000	2.16043000	0.00026200
H	4.45358900	-0.00001700	0.00026200

$Ph_2I\cdot$

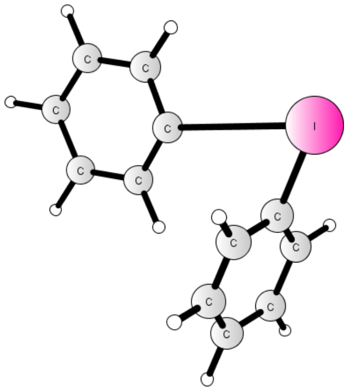

I	-1.71493200	-1.35156200	-0.01814000
C	-1.26401500	0.72521500	0.03045700
C	-1.21965100	1.44551600	-1.16593100
C	-0.99814100	1.34075500	1.25643600
C	-0.90526900	2.80758700	-1.12719000
H	-1.42112800	0.95297200	-2.11835600
C	-0.68566100	2.70314000	1.27862500
H	-1.02510800	0.76611100	2.18332400
C	-0.63849300	3.43879900	0.09108800
H	-0.86785300	3.37487400	-2.06051400
H	-0.47424900	3.18809000	2.23484100
H	-0.39263100	4.50291100	0.11481600
C	1.75905000	-0.50383300	-0.04075000
C	2.28337700	0.76478900	-0.17615100
C	2.48528400	-1.66802800	0.09692600
C	3.68536900	0.86781400	-0.17303200
H	1.65152700	1.65011700	-0.27939000
C	3.88551400	-1.54048900	0.09819300
H	2.00709000	-2.64594900	0.20057600
C	4.47620000	-0.27895700	-0.03636900
H	4.15595800	1.84975500	-0.27741000
H	4.51120600	-2.43142500	0.20437200
H	5.56521300	-0.18851000	-0.03462600

$(PhO)_2P(O)S\cdot$

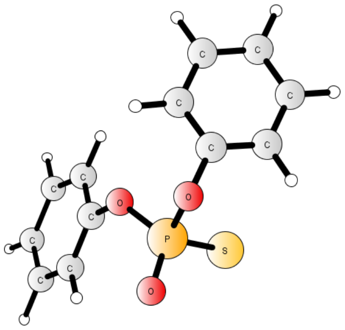

P	-0.00545600	1.31007100	0.23468800
O	0.12611800	1.52036300	1.70783400
O	-1.50650900	1.25082100	-0.36363700
O	0.53974200	-0.11527600	-0.33035400
S	0.92565600	2.82847900	-0.76012900
C	1.80926470	-0.63831241	-0.13423392
C	2.45599238	-0.26481652	1.04591615
C	2.42750586	-1.47476410	-1.06807759
C	3.74446563	-0.73782847	1.29600620
H	1.93926515	0.39028326	1.74943217
C	3.71552039	-1.94481523	-0.79901694
H	1.90906267	-1.74075855	-1.98927625
C	4.37719749	-1.58138808	0.37680408
H	4.25473081	-0.44769369	2.21747200
H	4.20494847	-2.59936683	-1.52407909
H	5.38490963	-1.95247034	0.57621053
C	-1.79057105	1.06088086	-1.71489614
C	-2.02612512	2.20086469	-2.48246633
C	-1.86600720	-0.21952498	-2.26778027
C	-2.34584660	2.05429831	-3.83386115
H	-1.95553167	3.18373112	-2.01356158
C	-2.17845987	-0.34924190	-3.62317281
H	-1.68963165	-1.09446720	-1.64052664
C	-2.41967199	0.78160363	-4.40832306
H	-2.53384963	2.94286479	-4.44117058

H	-2.23870598	-1.34710134	-4.06395738
H	-2.66721759	0.67153562	-5.46642460

TS1-O

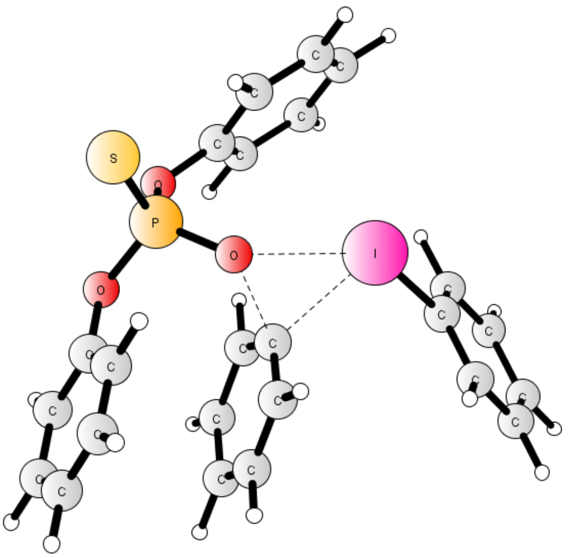

I	-1.70603900	0.13938600	-1.17184000
C	-3.10081100	-1.27717600	-0.44585700
C	-3.90979100	-0.91692100	0.63603500
C	-3.14094200	-2.55920600	-1.00092600
C	-4.78792600	-1.86769100	1.16355000
H	-3.85306100	0.08688500	1.06147300
C	-4.02002900	-3.49891200	-0.45568600
H	-2.49544100	-2.82558700	-1.83938700
C	-4.84255200	-3.15481900	0.62105300
H	-5.42795200	-1.59889400	2.00727000
H	-4.05951100	-4.50588000	-0.87774700
H	-5.52843100	-3.89435200	1.04057000
C	0.21090400	-0.98000700	-0.12266300
C	0.11079800	-0.99557100	1.25601400
C	0.79386100	-1.97586500	-0.88772400
C	0.62907200	-2.12102200	1.91130600
H	-0.34757000	-0.17851400	1.80891300
C	1.30112500	-3.08492800	-0.20060400
H	0.87721800	-1.88849800	-1.97036500
C	1.21963800	-3.16213500	1.19230200
H	0.56523100	-2.16570600	3.00141000
H	1.78518600	-3.88013200	-0.77223500
H	1.63141000	-4.02614200	1.71684400
P	1.89672300	1.68469300	-0.07984900
O	2.80335700	0.71348200	0.89773800
O	0.99455500	2.26647900	1.19199400
C	3.58403100	-0.33300600	0.47928400
C	4.05675700	-1.18678800	1.48225200
C	3.90347300	-0.57539500	-0.86261900
C	4.83626400	-2.29205400	1.14278500
H	3.79105500	-0.97219800	2.51848500
C	4.68433000	-1.68703300	-1.18817100
H	3.54852800	0.09722000	-1.64300700
C	5.14970700	-2.55276400	-0.19508900
H	5.19774300	-2.95687600	1.93143500
H	4.93062600	-1.87297800	-2.23672600
H	5.75920900	-3.41969500	-0.46033400
C	-0.29348400	2.70851000	1.03897900
C	-0.70572900	3.46882700	-0.06366200
C	-1.21194500	2.36170600	2.03741600
C	-2.04714000	3.84150800	-0.17657600
H	0.02323300	3.75648700	-0.82256900
C	-2.54896500	2.74666200	1.91614100
H	-0.86086300	1.79654400	2.90283100
C	-2.97638700	3.47716600	0.80247900
H	-2.36577700	4.42695500	-1.04267400
H	-3.25992000	2.47400500	2.70035200
H	-4.02344800	3.77280300	0.70579500
S	2.91233800	3.05092700	-1.01784300
O	0.96693700	0.77045500	-0.91719600

TS1-S

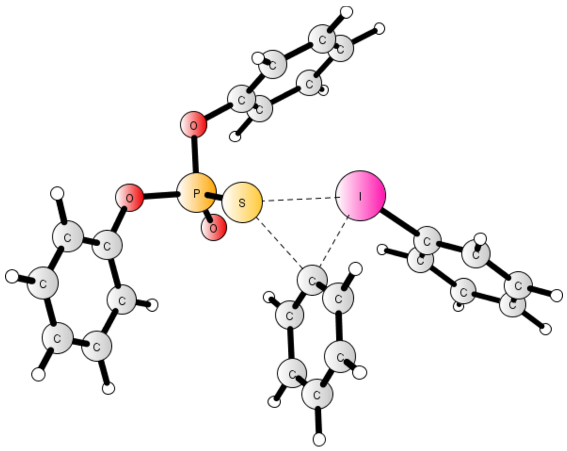

I	-1.75178300	0.31446000	-0.62647200
C	-3.42354900	-0.74284800	0.14458900
C	-3.61245800	-0.77275800	1.52990800
C	-4.26361600	-1.44375000	-0.72582600
C	-4.67611900	-1.51637400	2.04812900
H	-2.94022400	-0.22699700	2.19454800
C	-5.32166000	-2.18320000	-0.19045500
H	-4.09600600	-1.41731200	-1.80393300
C	-5.52862300	-2.21931500	1.19170800
H	-4.83520700	-1.54648700	3.12868000
H	-5.98557500	-2.73496100	-0.86028800
H	-6.35714800	-2.80009300	1.60353300
C	-0.17215500	-1.61665600	-0.60427500
C	0.13726600	-2.03306800	0.67719400
C	-0.36056300	-2.45502300	-1.69202300
C	0.29512500	-3.41294900	0.86647900
H	0.29944100	-1.30696200	1.47518400
C	-0.19488700	-3.82786800	-1.46492400
H	-0.60810500	-2.07340300	-2.68308700
C	0.12944300	-4.30942000	-0.19333700
H	0.55435800	-3.77709000	1.86434800
H	-0.32472700	-4.51801600	-2.30287100
H	0.25278000	-5.38192600	-0.02919400

P	1.90350300	1.12813300	0.38634400
O	1.70068400	2.76019600	0.23105300
O	3.54046000	1.22054200	0.47671600
C	0.41141700	3.24953400	0.18286600
C	-0.11263100	3.65506500	-1.04807500
C	-0.35958200	3.31439000	1.34803900
C	-1.42613200	4.12704900	-1.11097700
H	0.51242600	3.57867000	-1.93878300
C	-1.67075800	3.78766300	1.27315300
H	0.07316900	2.96996400	2.28725100
C	-2.20998300	4.18911500	0.04607100
H	-1.84086700	4.44116400	-2.07191200
H	-2.27786500	3.83703500	2.18048800
H	-3.23812900	4.55454500	-0.00774000
C	4.34418200	0.10169400	0.42077400
C	5.51147500	0.18779000	-0.34224500
C	4.02651100	-1.07346600	1.11150600
C	6.37092100	-0.91016100	-0.41012400
H	5.72561500	1.11604000	-0.87488000
C	4.88947400	-2.16831700	1.02537100
H	3.11690500	-1.11481200	1.71089300
C	6.06234900	-2.09312300	0.26917200
H	7.28481900	-0.84183600	-1.00552200
H	4.63937600	-3.08807400	1.56006700
H	6.73400500	-2.95259100	0.20892600
S	1.40935600	0.25326100	-1.37788700
O	1.25642500	0.59908100	1.62999800

TS2-O

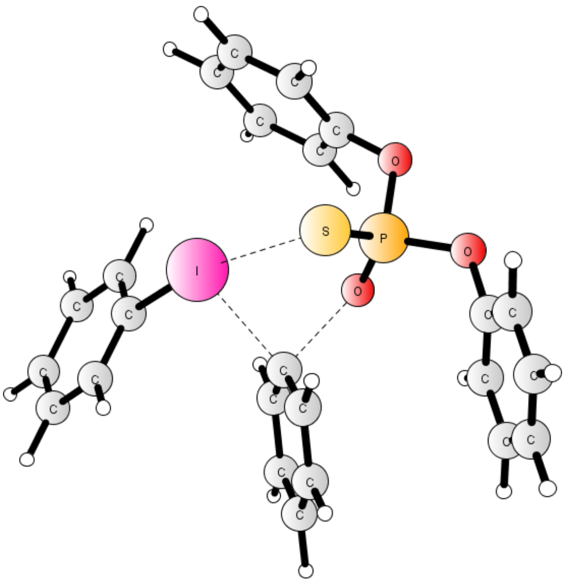

I	1.37325200	0.02864700	-1.18042700
C	3.04892000	-1.07237300	-0.45089400
C	3.22711000	-2.40285400	-0.84013200
C	3.91520100	-0.45551900	0.45632600
C	4.29812900	-3.12580500	-0.30792600
H	2.53543400	-2.87467000	-1.54038900
C	4.98610500	-1.18965200	0.97589700
H	3.75317900	0.58141500	0.75678600
C	5.17664600	-2.52152600	0.59669600
H	4.44424000	-4.16774100	-0.60299800
H	5.67056500	-0.71669000	1.68438500
H	6.01245000	-3.09179800	1.00876100
C	-0.12196400	-1.47938700	0.19212800
C	-1.11545000	-2.07220500	-0.55907100
C	0.56546600	-2.06723800	1.23402100
C	-1.42312700	-3.40133000	-0.24028100
H	-1.63832300	-1.52598000	-1.34515400
C	0.23201500	-3.39783700	1.52459800
H	1.32193900	-1.53036400	1.80531700
C	-0.75414200	-4.06569200	0.79315100
H	-2.20400900	-3.90729700	-0.81362700
H	0.75579600	-3.90230400	2.34067100
H	-1.00618200	-5.10088600	1.03219900
P	-1.60639600	1.35498300	0.24228300
O	-1.14962600	2.80881700	0.86715100
O	-3.16013700	1.45156100	0.75362500
C	-4.01425400	0.38960800	0.50108400
C	-3.84706100	-0.82279700	1.17644000
C	-5.03855700	0.55990000	-0.43222900
C	-4.72885700	-1.87394300	0.91434700
H	-3.02401600	-0.93038900	1.88262200
C	-5.91829800	-0.49622800	-0.67998600
H	-5.12659500	1.51426200	-0.95356600
C	-5.76552800	-1.71470800	-0.01017000
H	-4.59844500	-2.82481100	1.43663700
H	-6.72381000	-0.36710200	-1.40705800
H	-6.45384100	-2.53930800	-0.21011200
C	0.20000200	3.11438300	0.82664600
C	0.70299700	3.86206600	-0.24060700
C	1.04287500	2.64686300	1.83854800
C	2.07018100	4.14460700	-0.29198000
H	0.01931600	4.19536200	-1.02204500
C	2.40722000	2.93941800	1.77983500
H	0.61931000	2.04820500	2.64518500
C	2.92532000	3.68302300	0.71369000
H	2.46899200	4.72682300	-1.12623500
H	3.06915700	2.58228400	2.57279800
H	3.99387000	3.90654300	0.66927300
S	-1.47865300	1.30128800	-1.73732300
O	-0.85807500	0.26164100	1.01563800

TS2-S

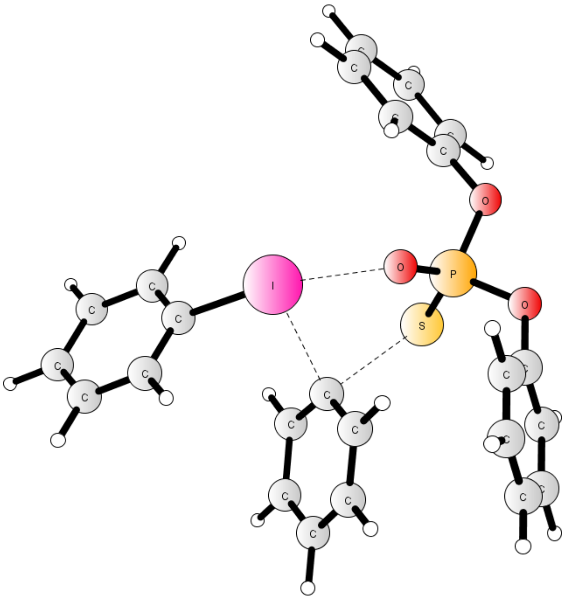

I	1.37886800	0.07725800	-0.97053000
C	3.24340400	-0.80541700	-0.47124800
C	3.50303100	-2.12595200	-0.85349300
C	4.16445600	-0.07773000	0.28992000
C	4.70758800	-2.72043100	-0.46968200
H	2.77068100	-2.68855200	-1.43540300
C	5.36927400	-0.68260100	0.66048000
H	3.94445900	0.94736500	0.59492300
C	5.64045900	-2.00098900	0.28343600
H	4.91571500	-3.75260600	-0.76164600
H	6.09522600	-0.11970200	1.25223100
H	6.58135800	-2.47067700	0.57956600
C	0.22495000	-1.52334000	0.59274500
C	-0.72736500	-2.27884700	-0.07141600
C	1.08920700	-2.01310600	1.55603400
C	-0.83773000	-3.62458500	0.29740500
H	-1.37137700	-1.84587400	-0.83594400
C	0.95321400	-3.36521000	1.90238700
H	1.83271900	-1.38121900	2.04236000
C	-0.00435600	-4.17002200	1.28033200
H	-1.59044600	-4.24036400	-0.20065200
H	1.61067100	-3.77801300	2.67208500
H	-0.10000000	-5.22182600	1.55803100
S	-0.96778400	0.47688200	1.70185300
O	-1.16671100	0.82741400	-1.25359300
P	-1.83952600	1.20556400	0.05078900
O	-2.06249200	2.82524000	0.19032300
O	-3.44386100	0.85573700	0.00559400
C	-3.82180400	-0.46226500	-0.18173500
C	-4.10729200	-1.25889500	0.92924100
C	-3.90256100	-0.97755100	-1.47833600
C	-4.48739200	-2.58876500	0.73486400
H	-4.01307400	-0.83043400	1.92736600
C	-4.28367100	-2.30921400	-1.66080600
H	-3.65307600	-0.33175800	-2.32099000
C	-4.57567300	-3.11726700	-0.55679700
H	-4.70835200	-3.21712400	1.60093700
H	-4.35034900	-2.71687700	-2.67250900
H	-4.87273000	-4.15842100	-0.70347400
C	-0.95941300	3.65950500	0.23382400
C	-0.19592100	3.87420400	-0.91753200
C	-0.63553500	4.28537400	1.43904500
C	0.90103900	4.73671400	-0.85610900
H	-0.47049800	3.36013700	-1.83904600
C	0.46053100	5.14984600	1.48683200
H	-1.24424400	4.08194400	2.32133200
C	1.23179100	5.37662300	0.34247500
H	1.49932500	4.91057700	-1.75402700
H	0.71553300	5.64488800	2.42709500
H	2.08901000	6.05268700	0.38457100

8. NMR Spectra

^{1}H NMR, 500 MHz, $CDCl_3$

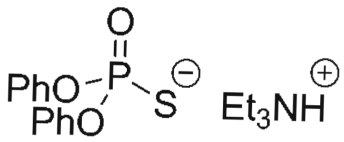

1a

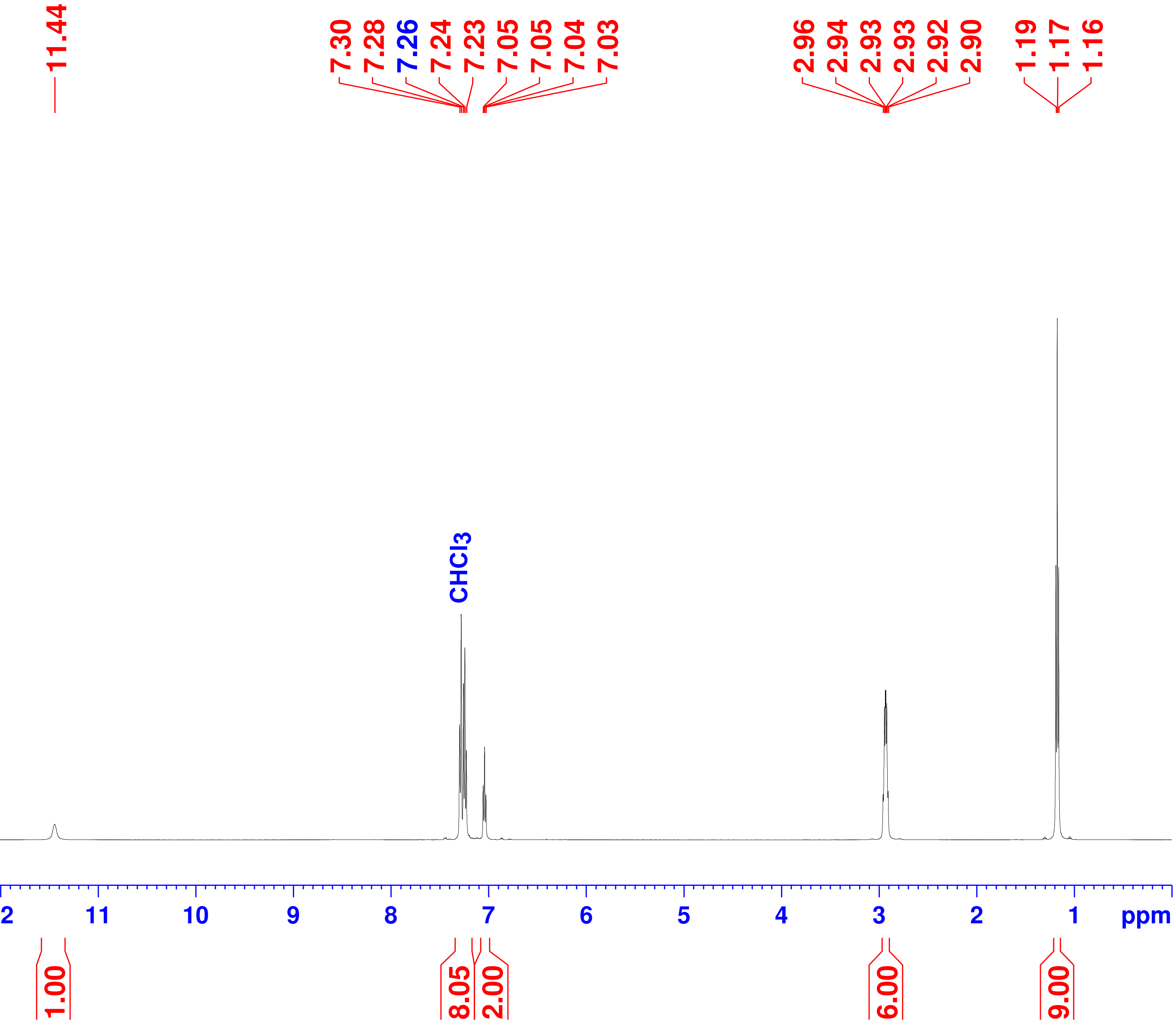

^{13}C NMR, 126 MHz, $CDCl_3$

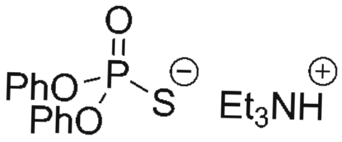

1a

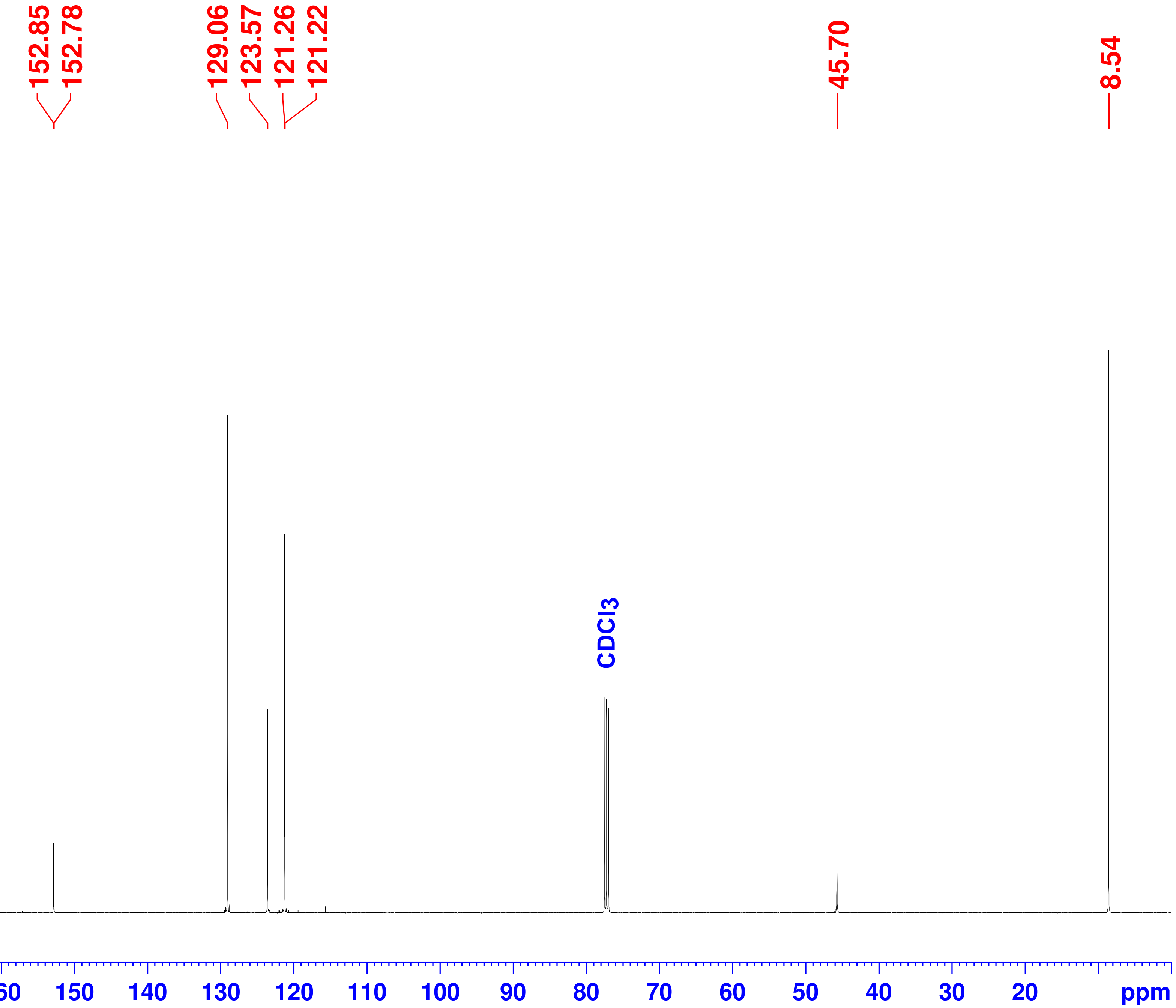

^{31}P NMR, 203 MHz, $CDCl_3$

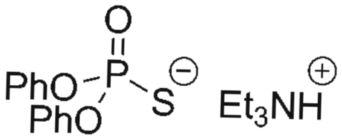

1a

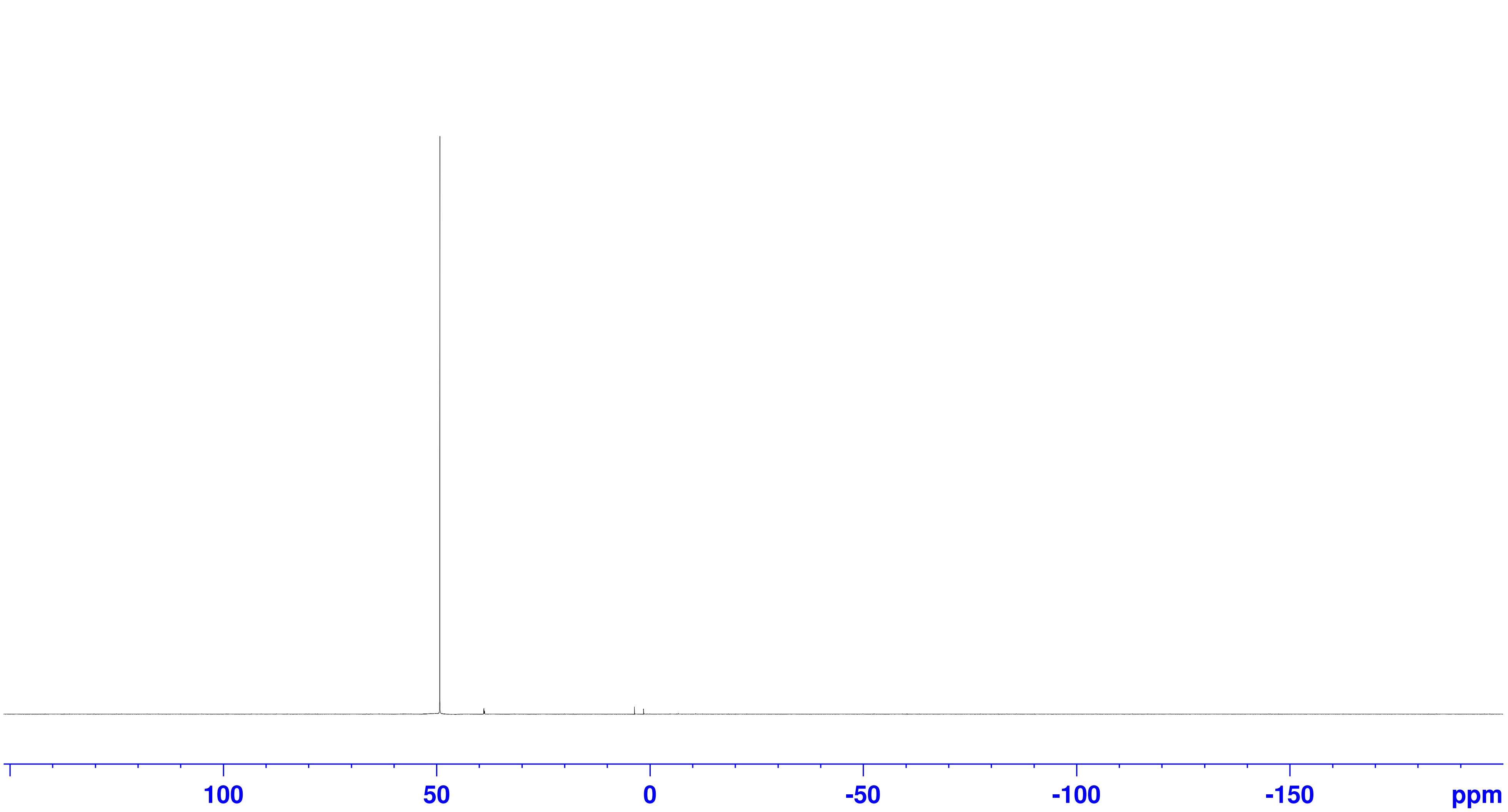

^{1}H NMR, 500 MHz, $CDCl_3$

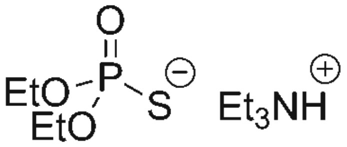

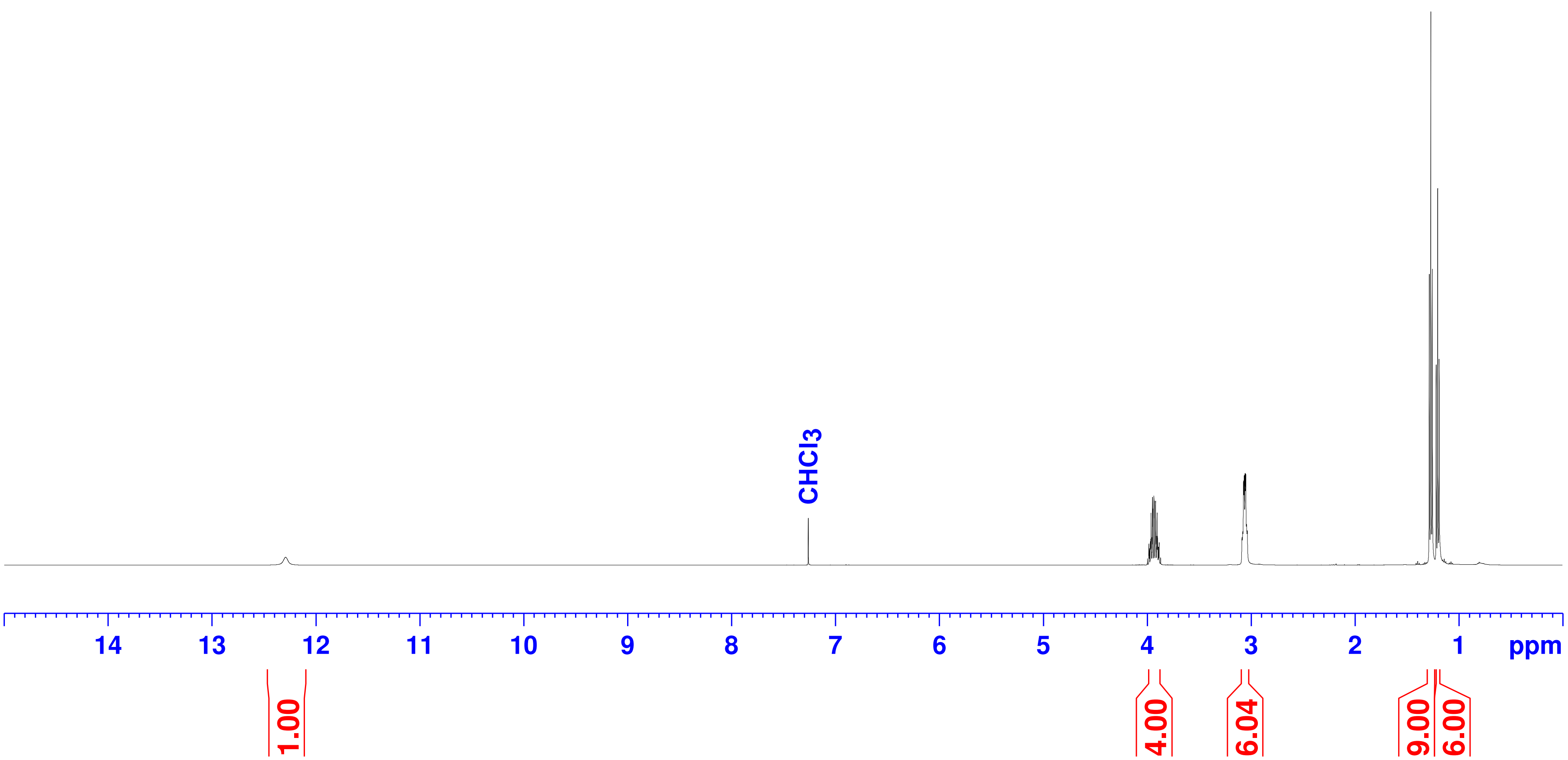

^{13}C NMR, 126 MHz, $CDCl_3$

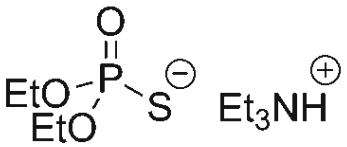

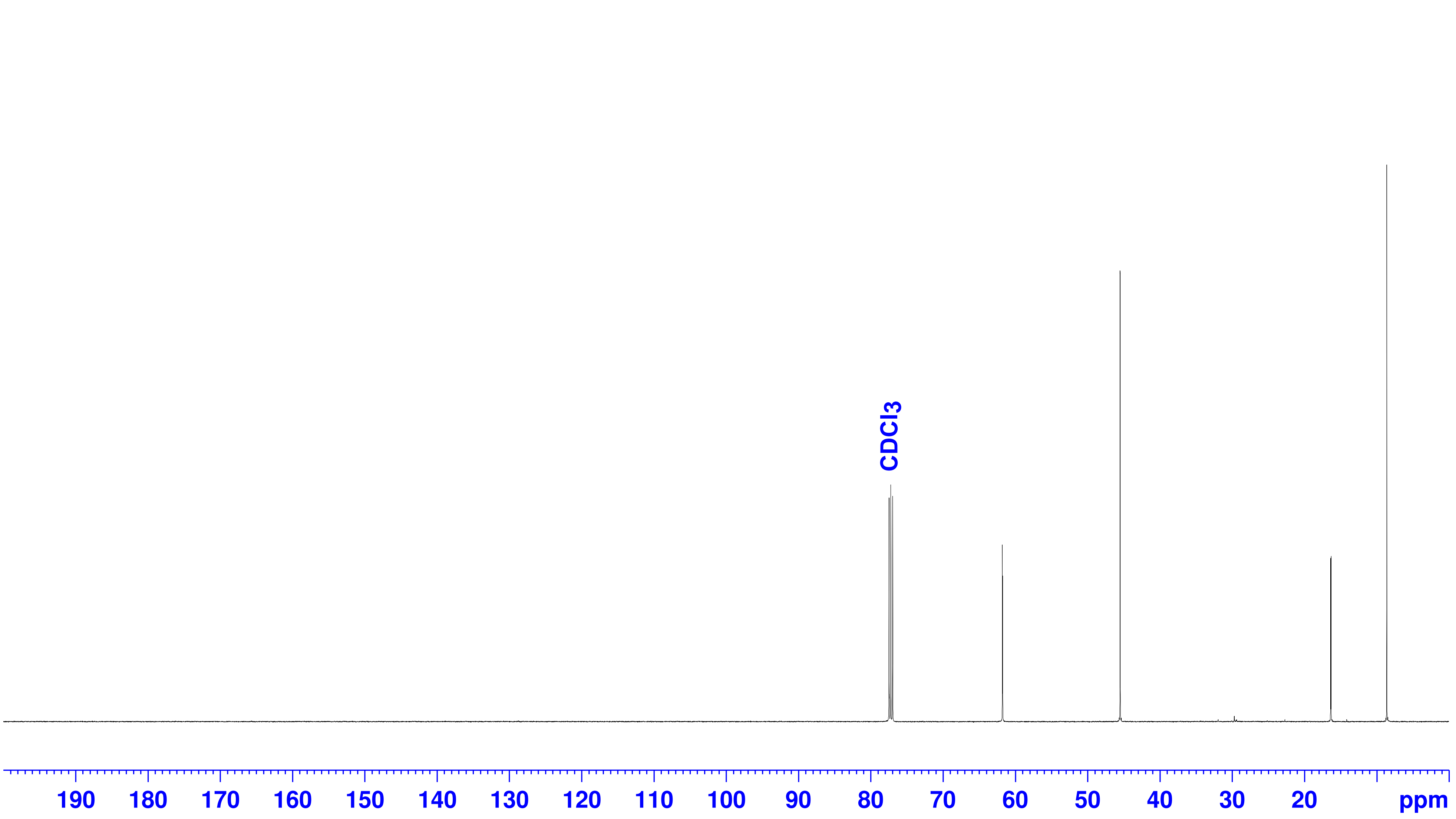

^{31}P NMR, 203 MHz, $CDCl_3$

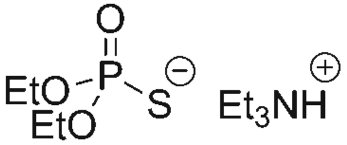

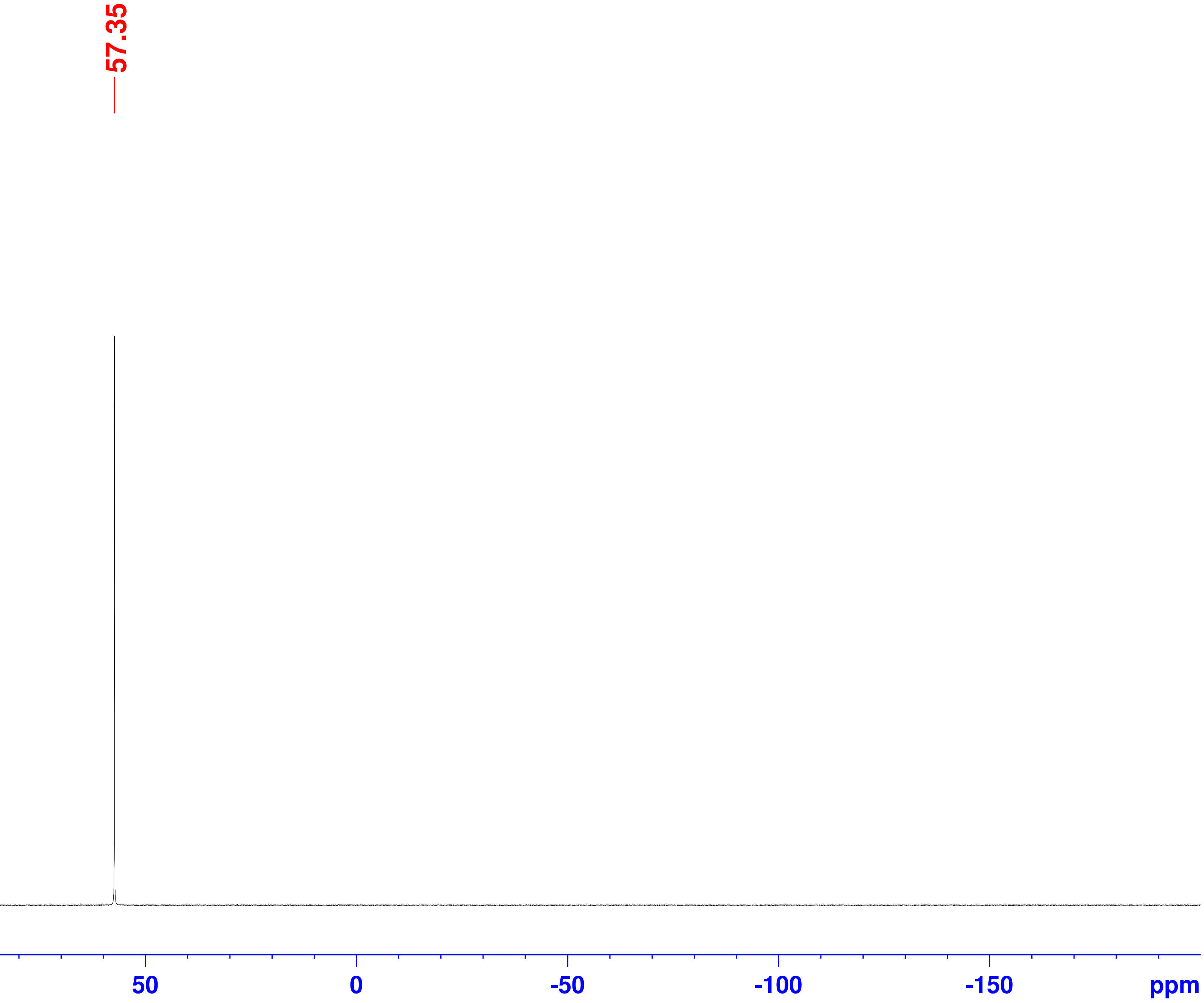

^{1}H NMR, 500 MHz, $CDCl_3$

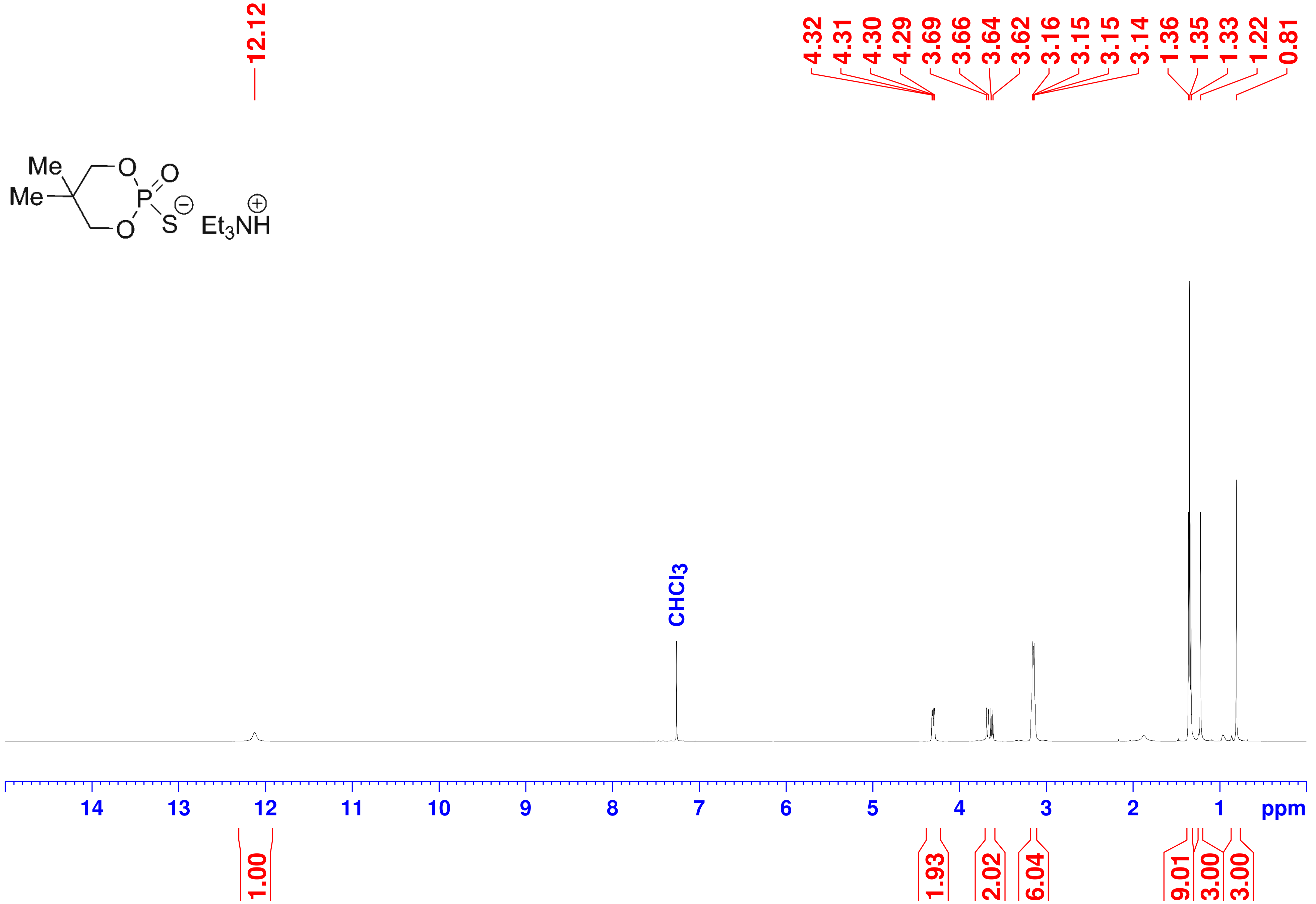

^{13}C NMR, 126 MHz, $CDCl_3$

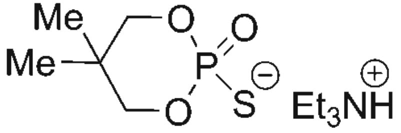

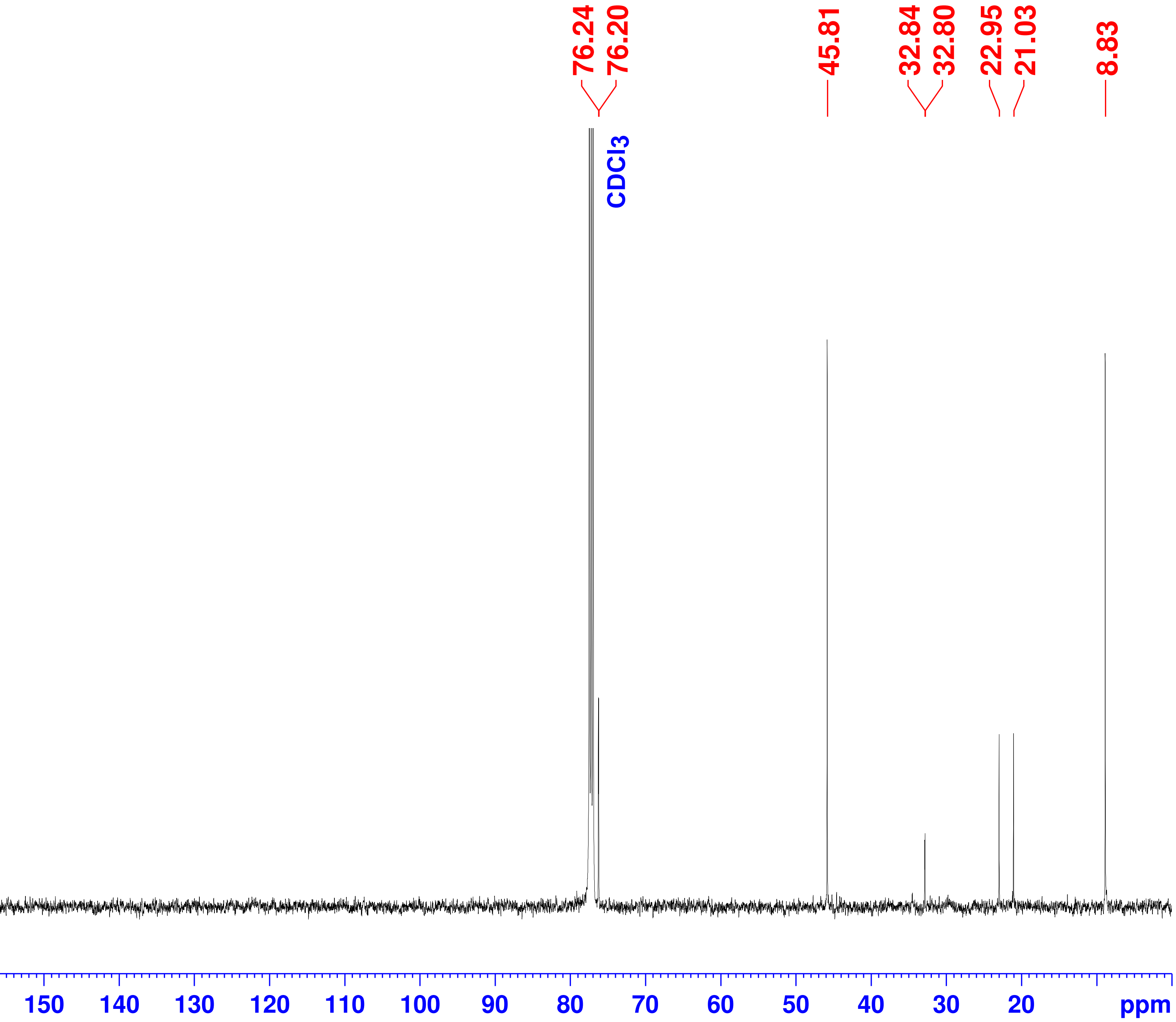

^{31}P NMR, 203 MHz, $CDCl_3$

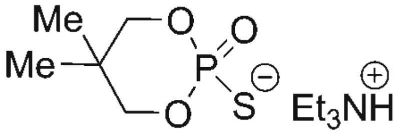

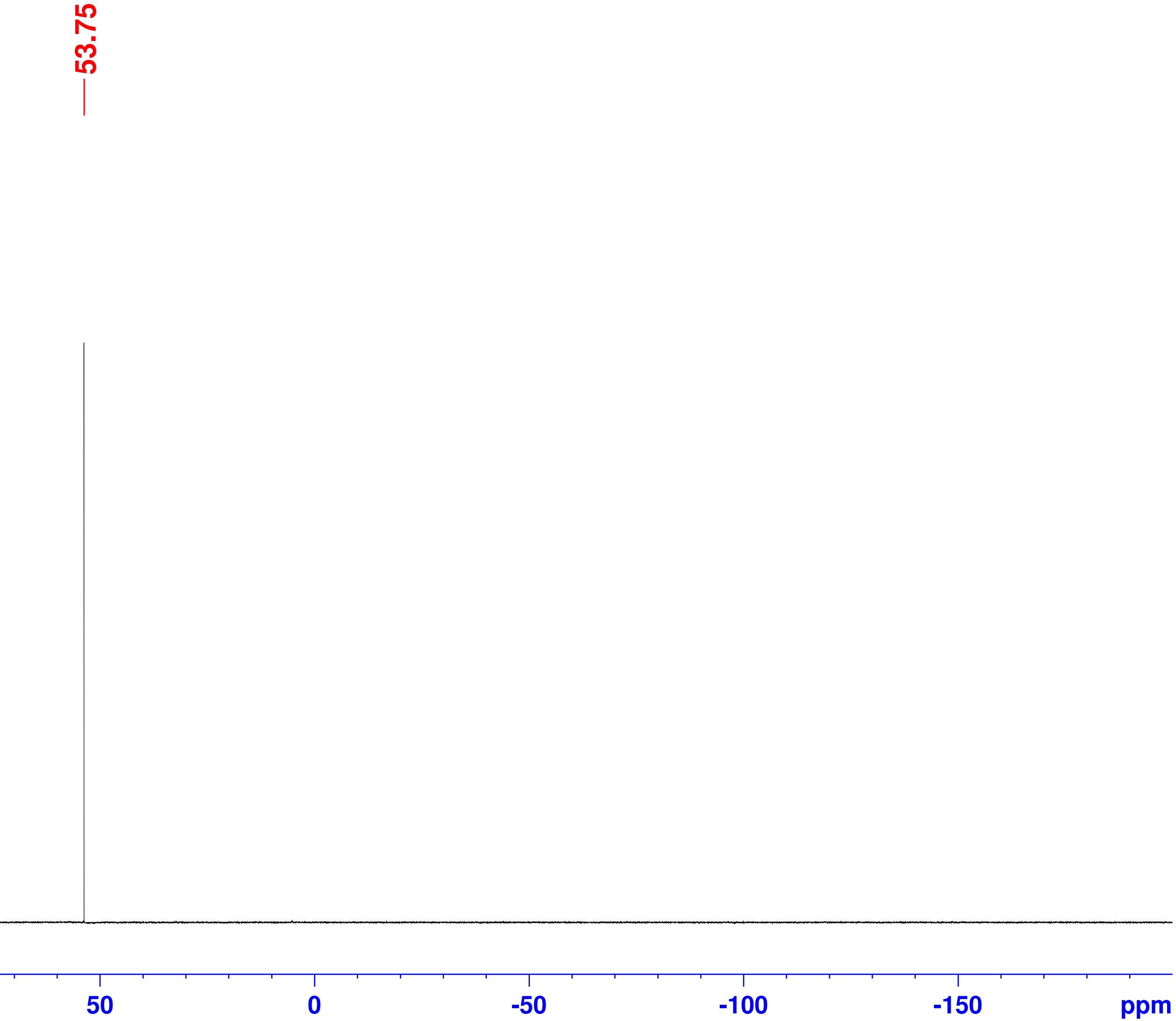

^{1}H NMR, 500 MHz, $CDCl_3$

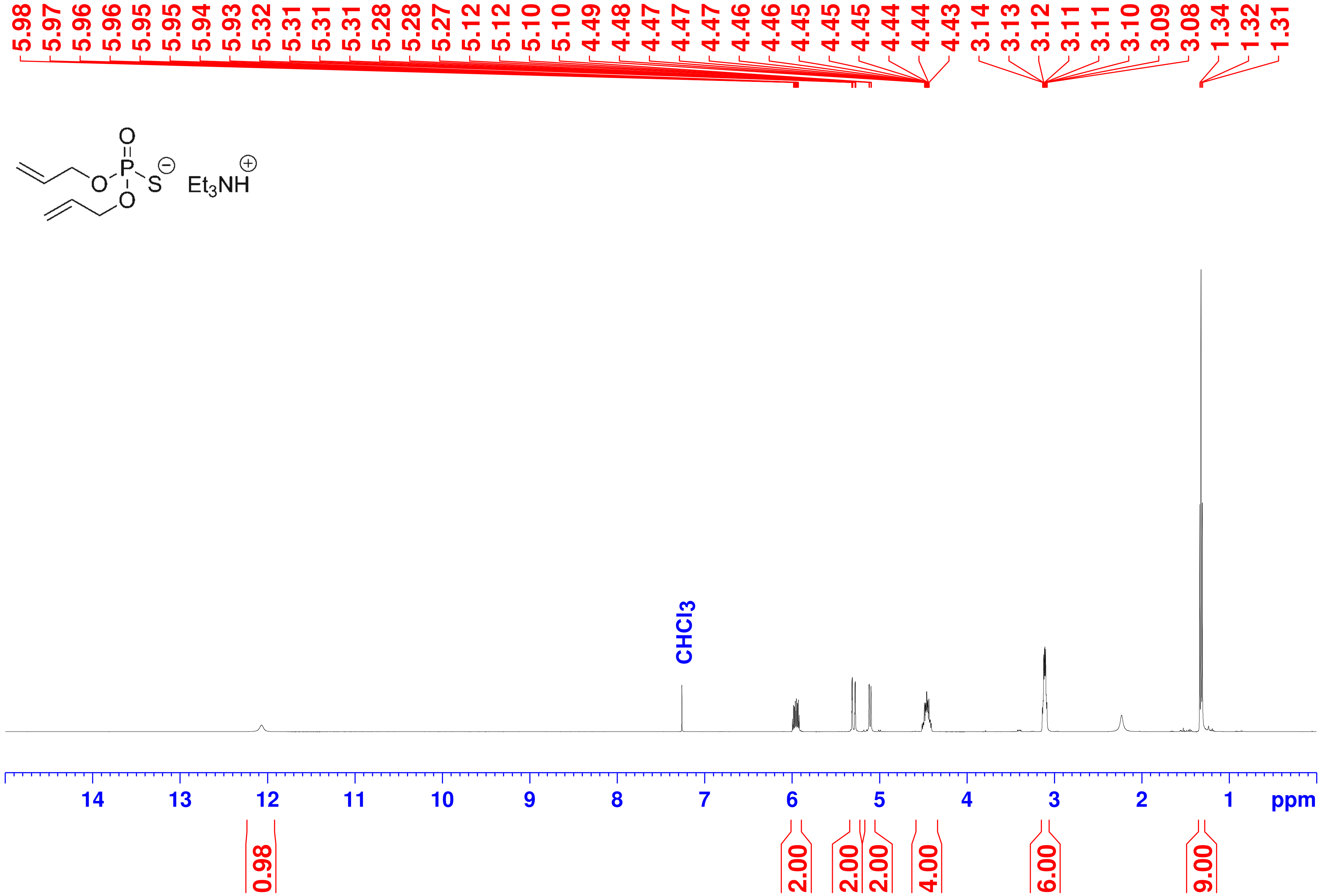

^{13}C NMR, 126 MHz, $CDCl_3$

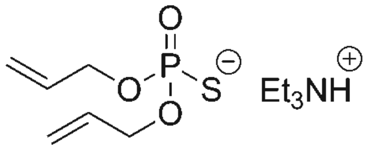

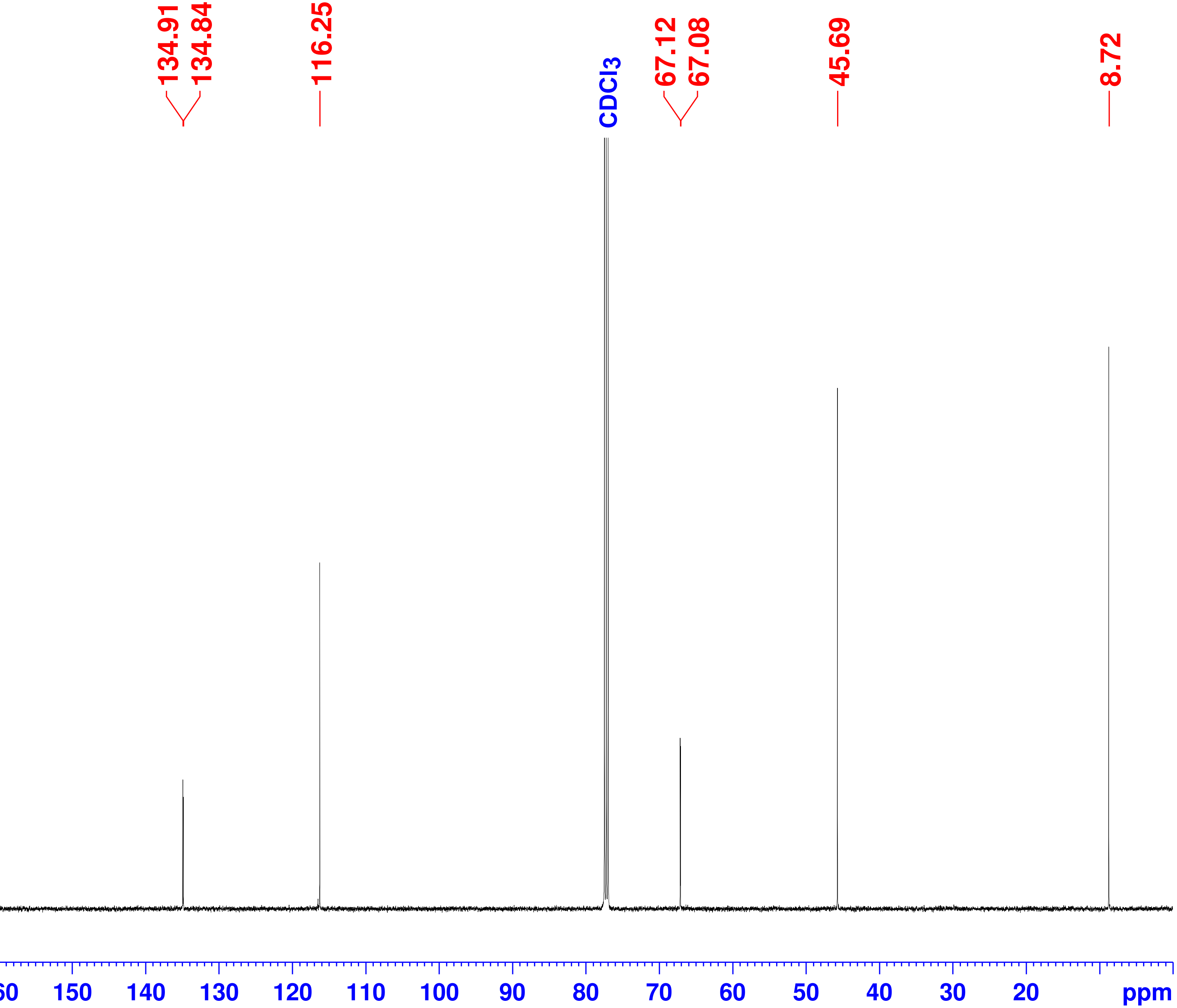

^{31}P NMR, 203 MHz, $CDCl_3$

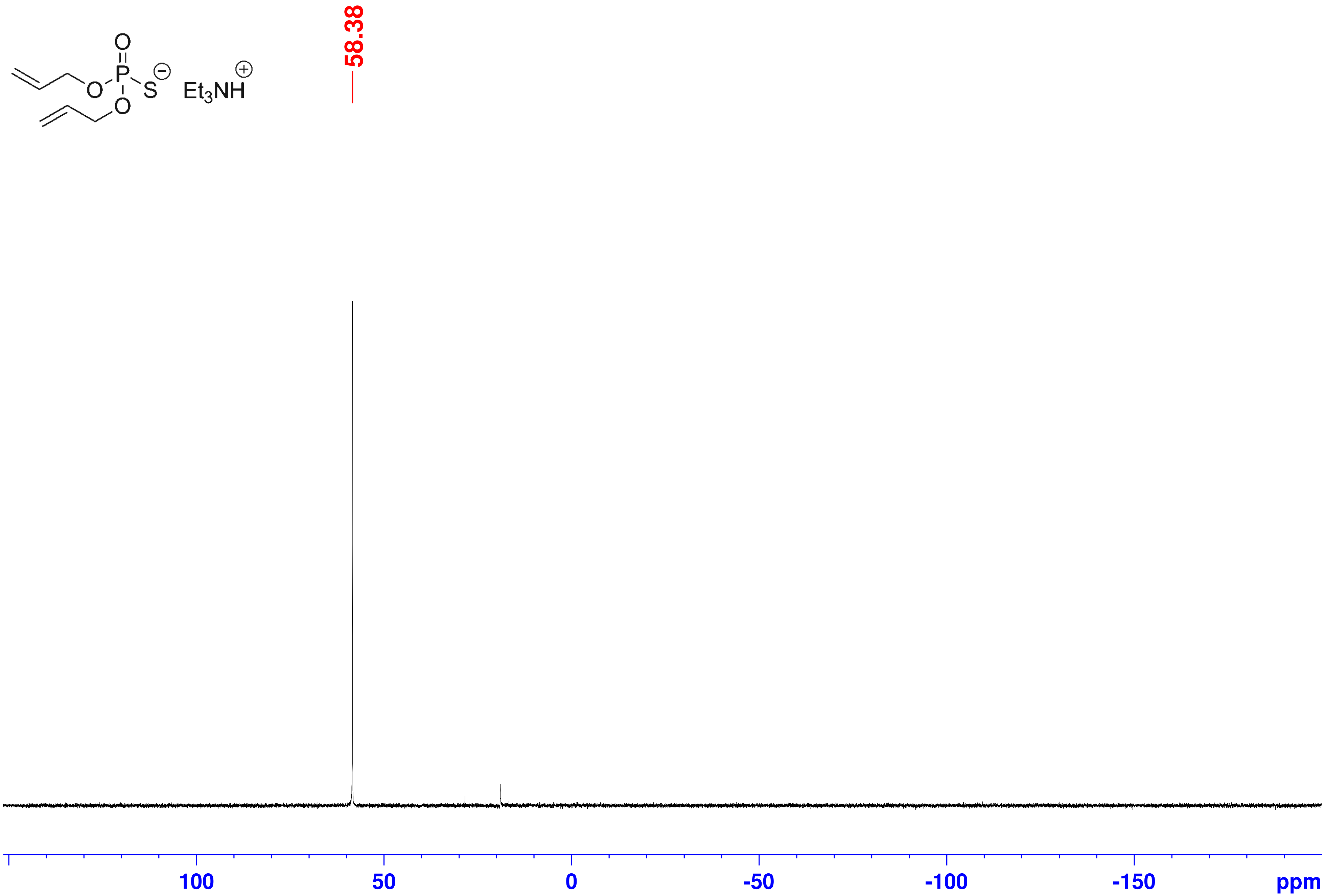

^{1}H NMR, 500 MHz, $CDCl_3$

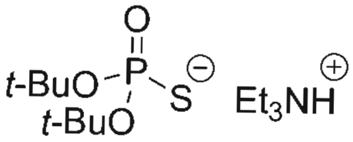

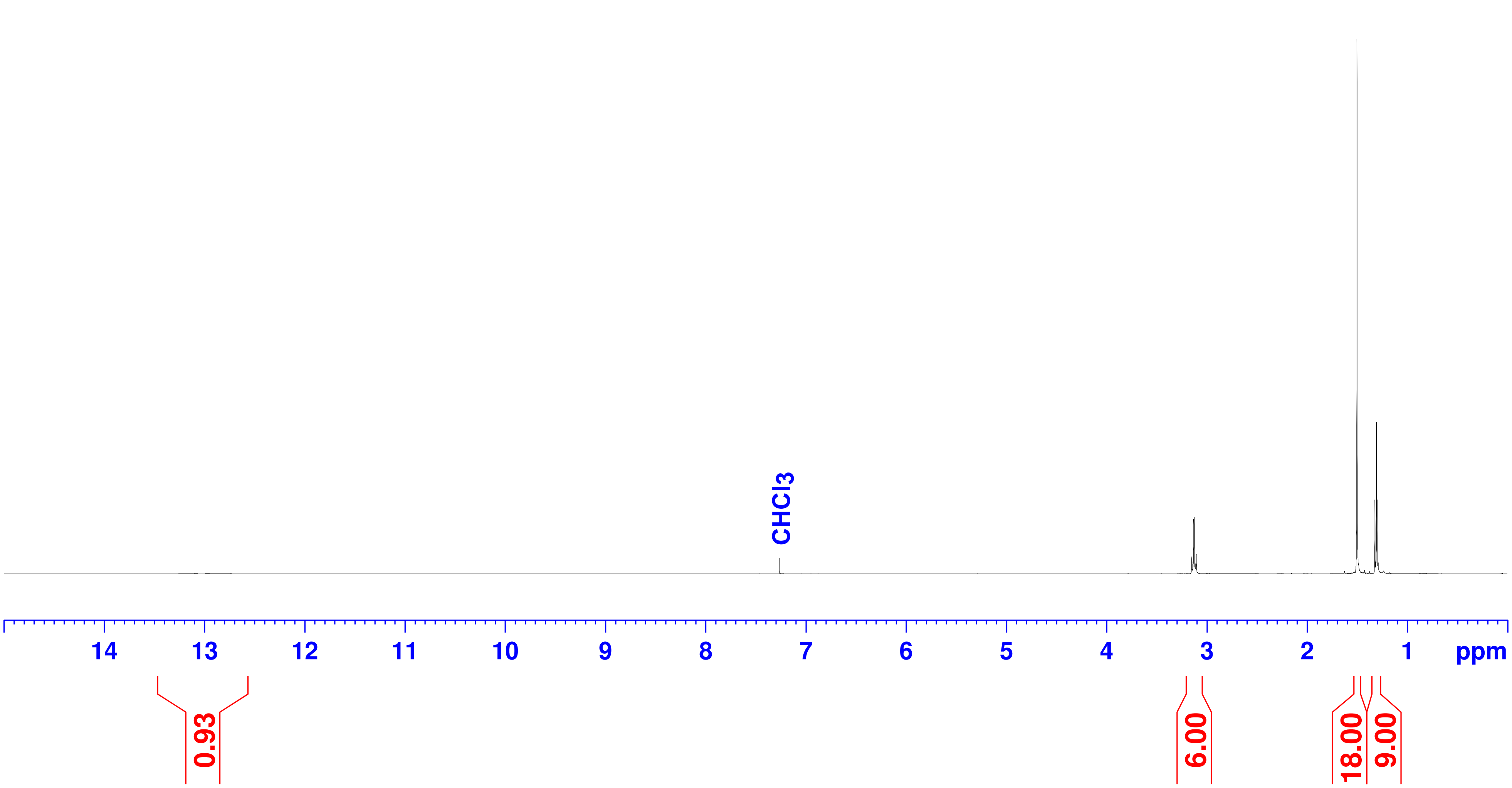

^{13}C NMR, 126 MHz, $CDCl_3$

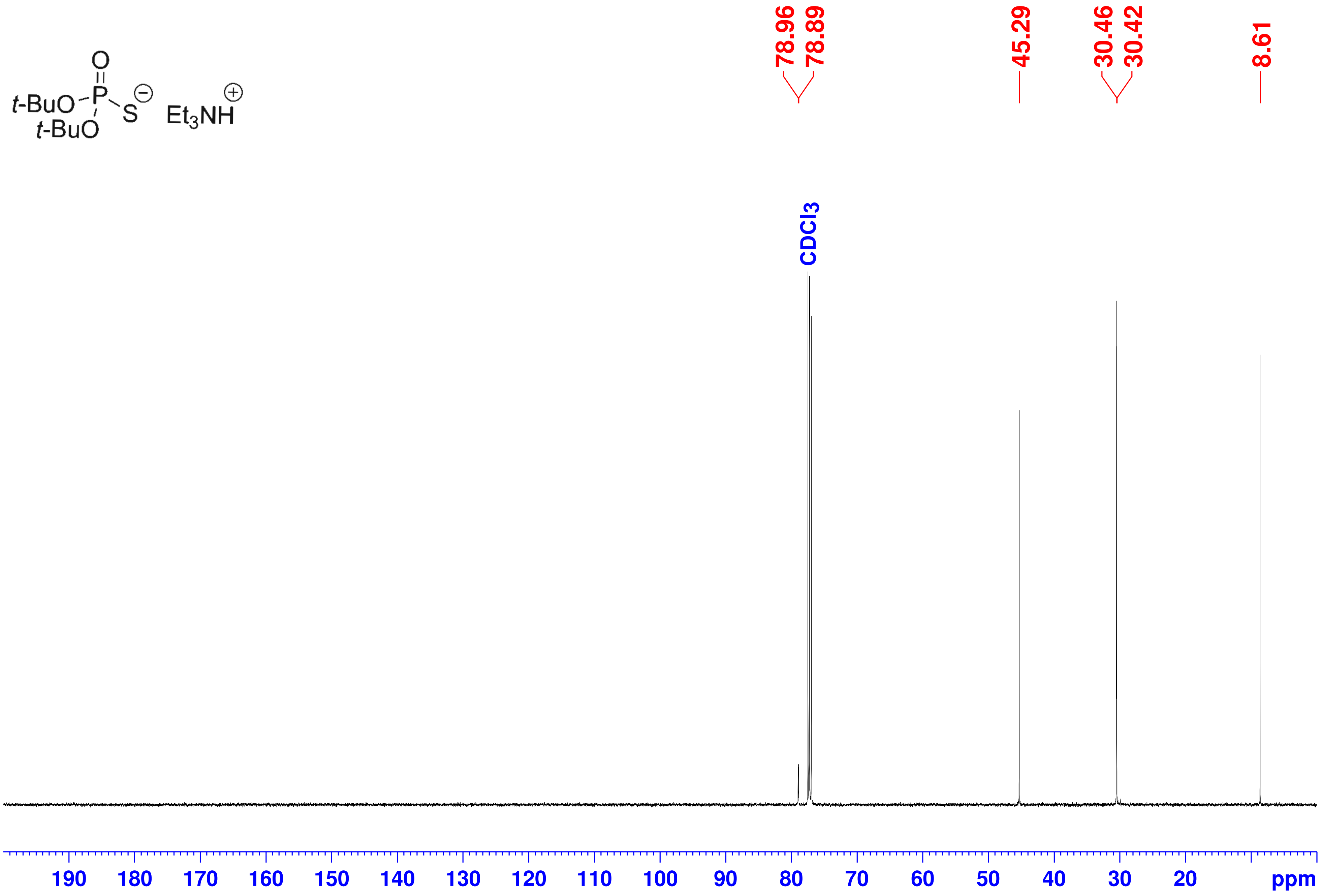

^{31}P NMR, 203 MHz, $CDCl_3$

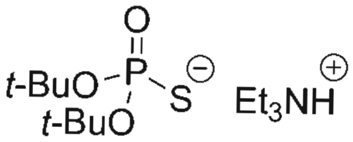

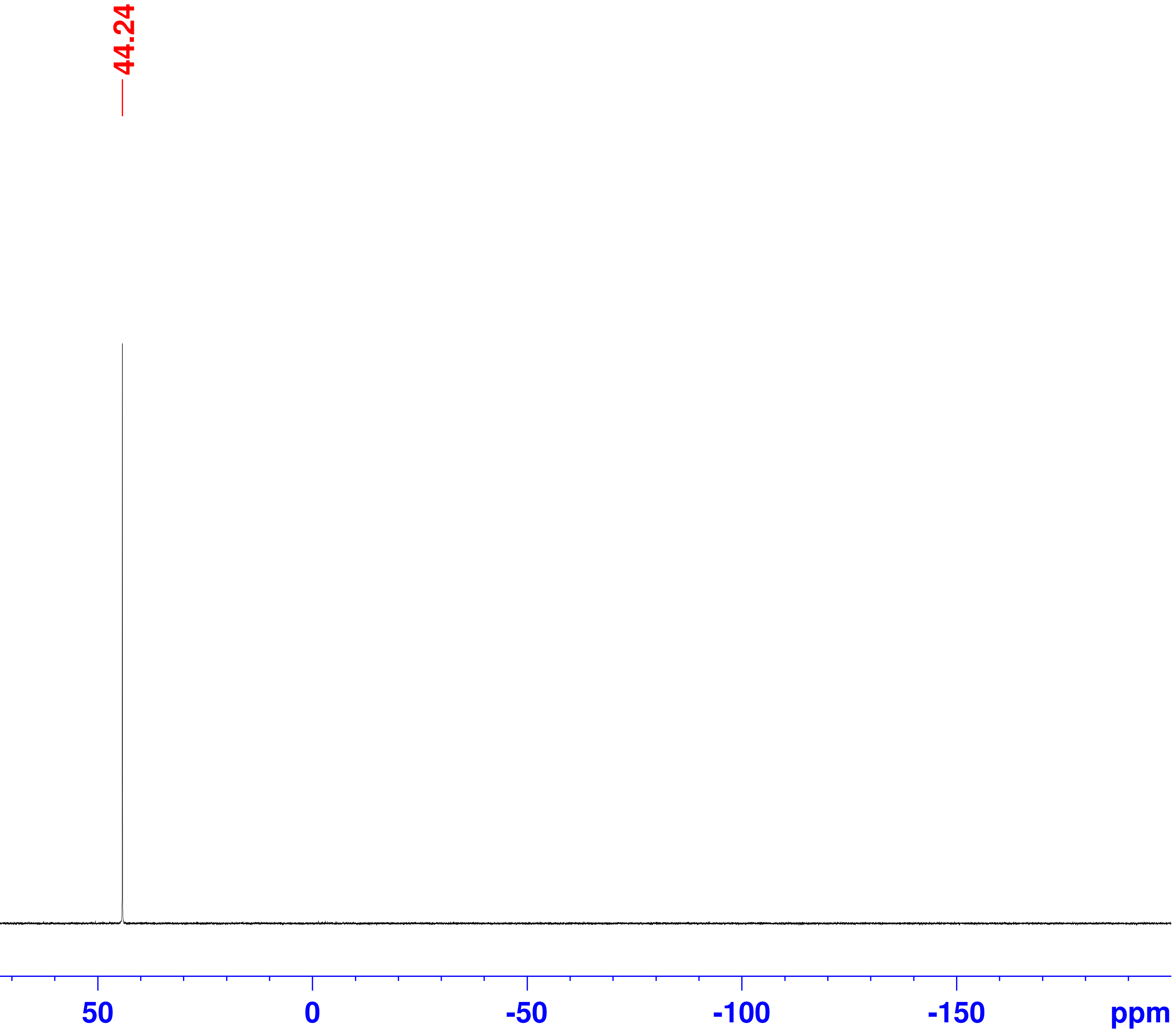

^{1}H NMR, 500 MHz, $CDCl_3$

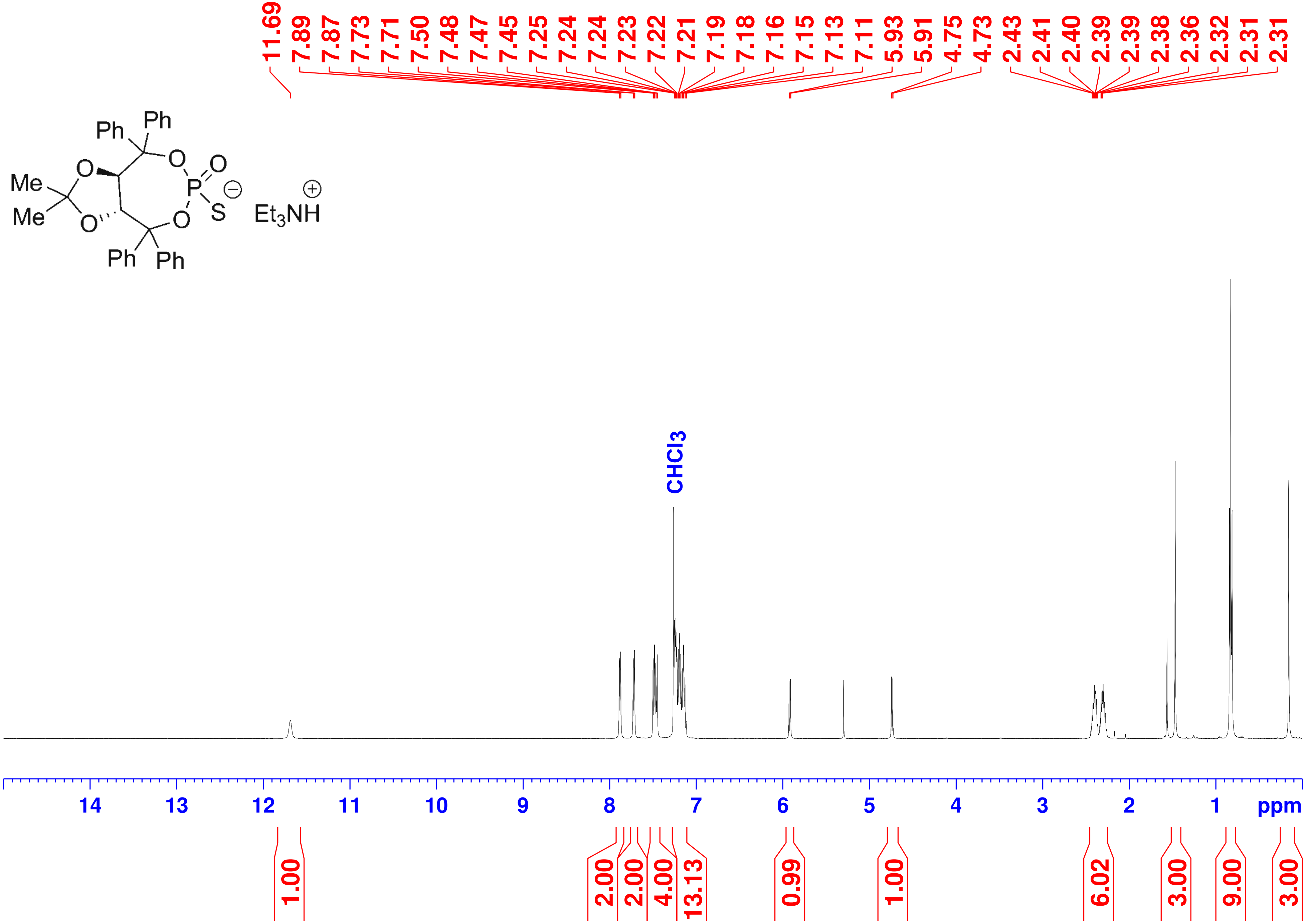

^{13}C NMR, 126 MHz, $CDCl_3$

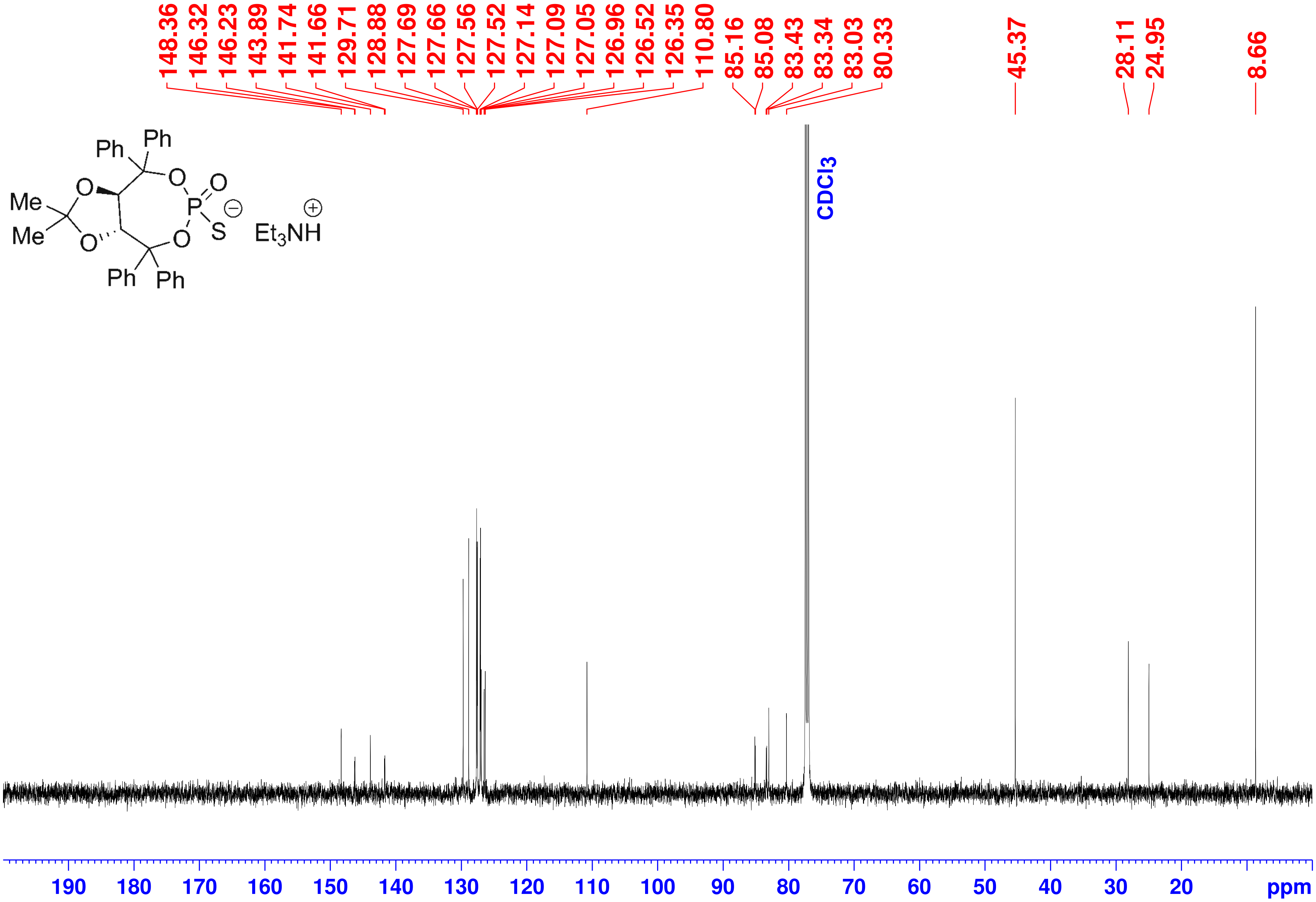

^{31}P NMR, 203 MHz, $CDCl_3$

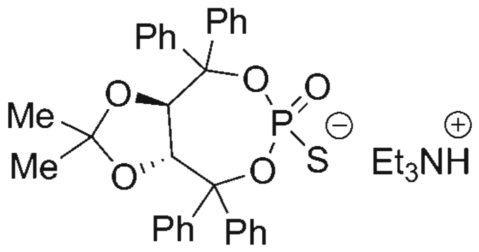

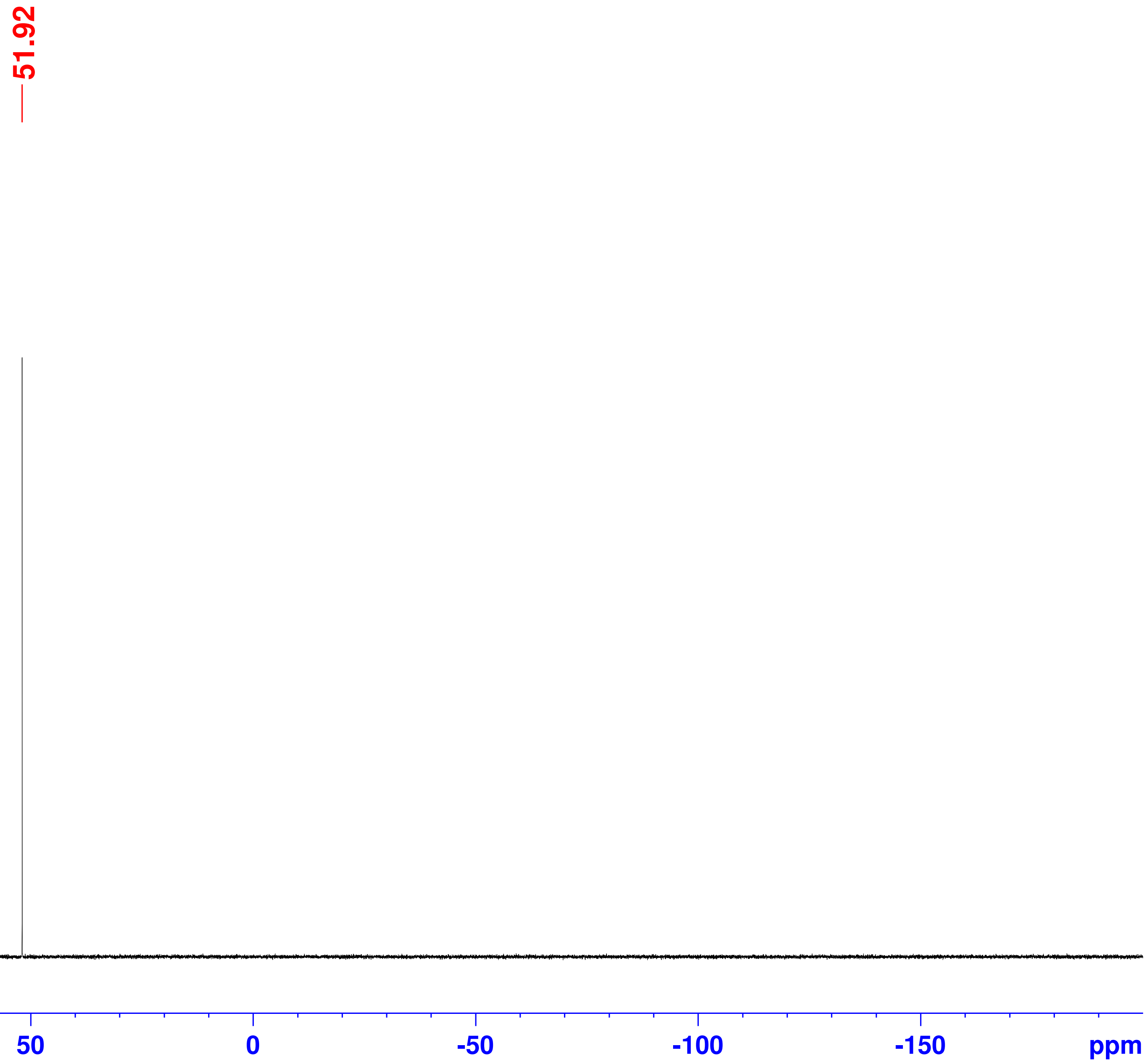

^{1}H NMR, 500 MHz, DMSO-d_6

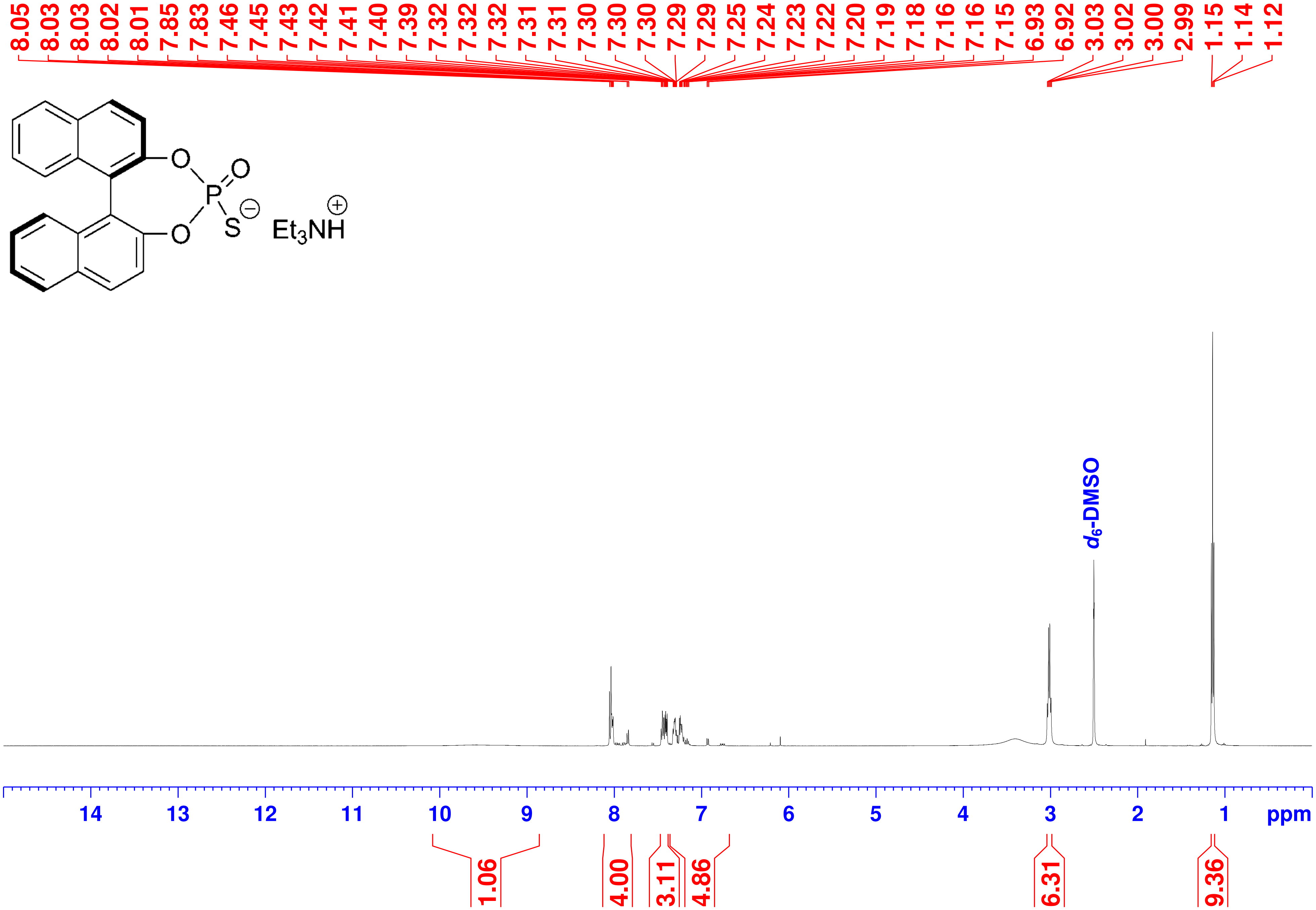

^{13}C NMR, 126 MHz, DMSO-d_6

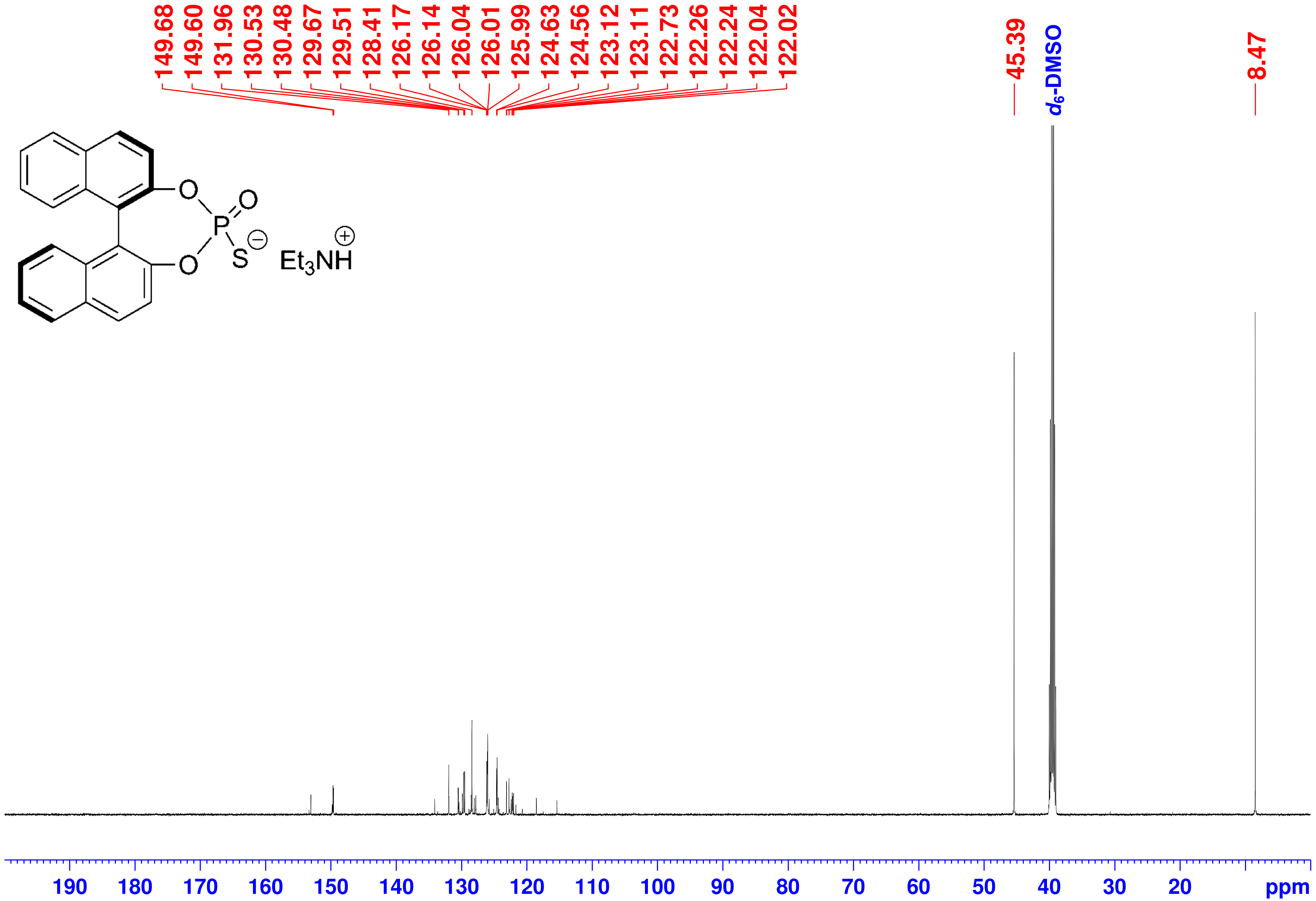

^{31}P NMR, 203 MHz, DMSO-d_6

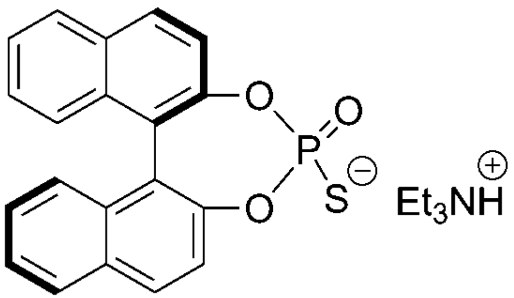

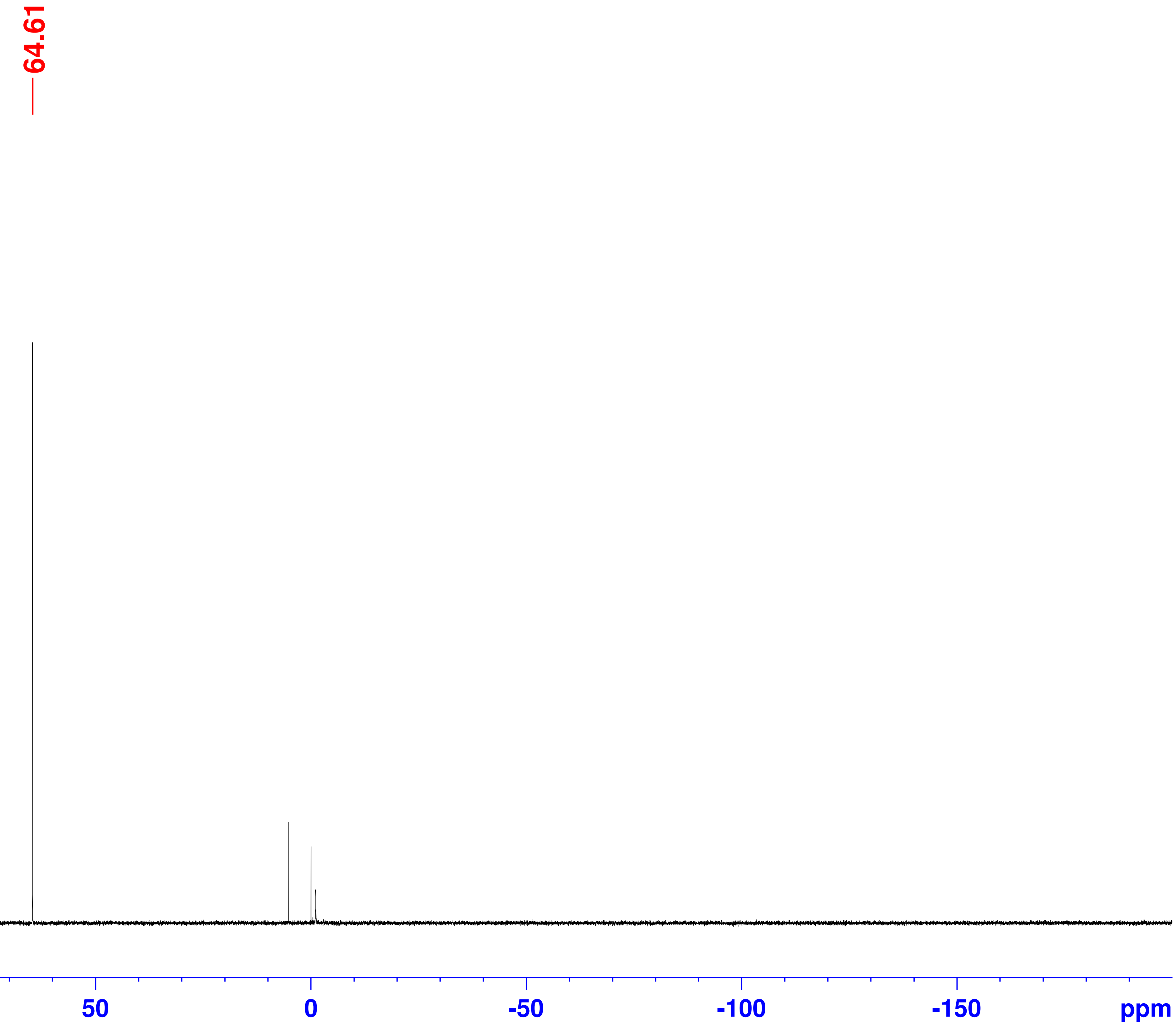

^{1}H NMR, 500 MHz, DMSO-d_6

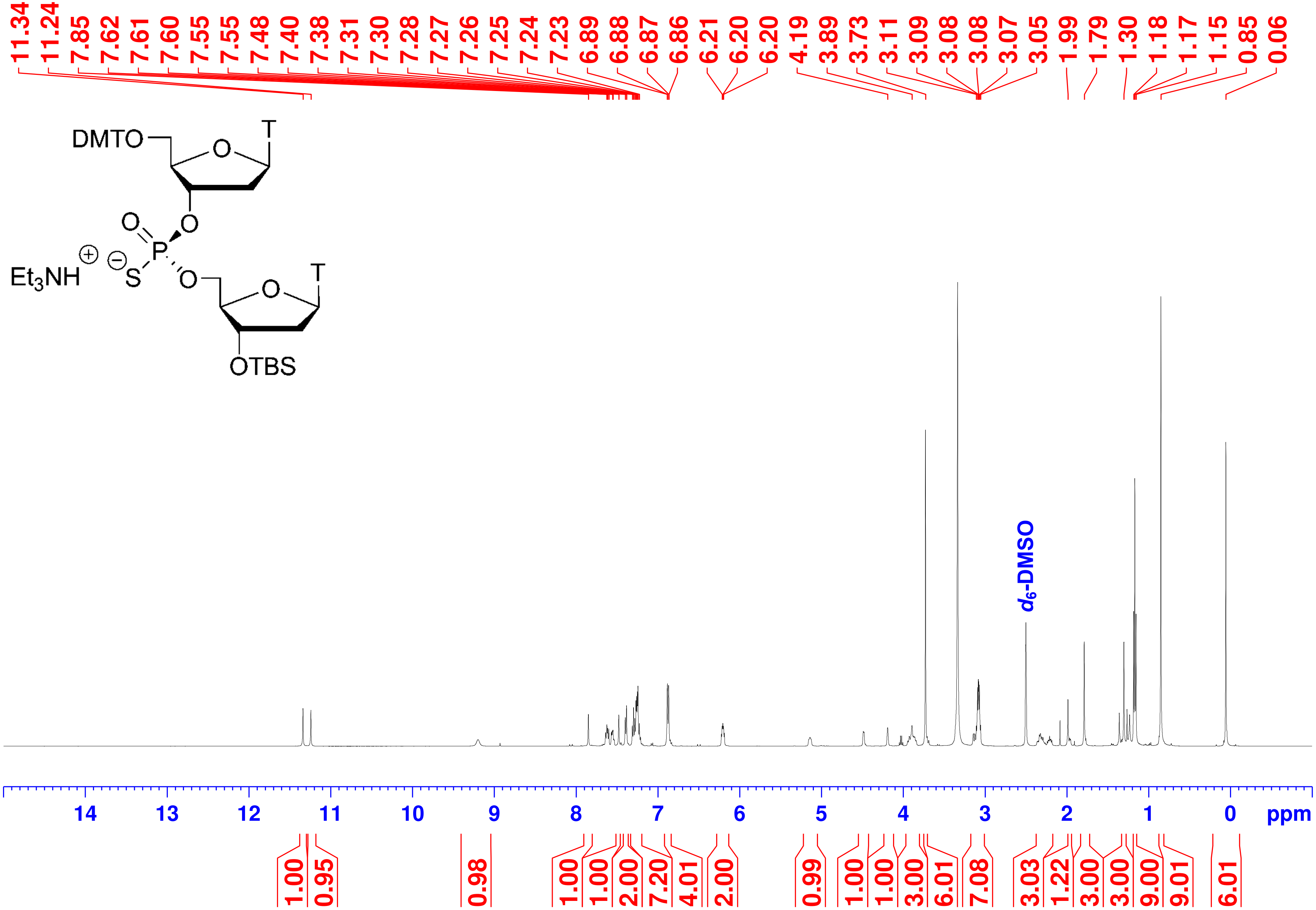

^{13}C NMR, 126 MHz, DMSO-d_6

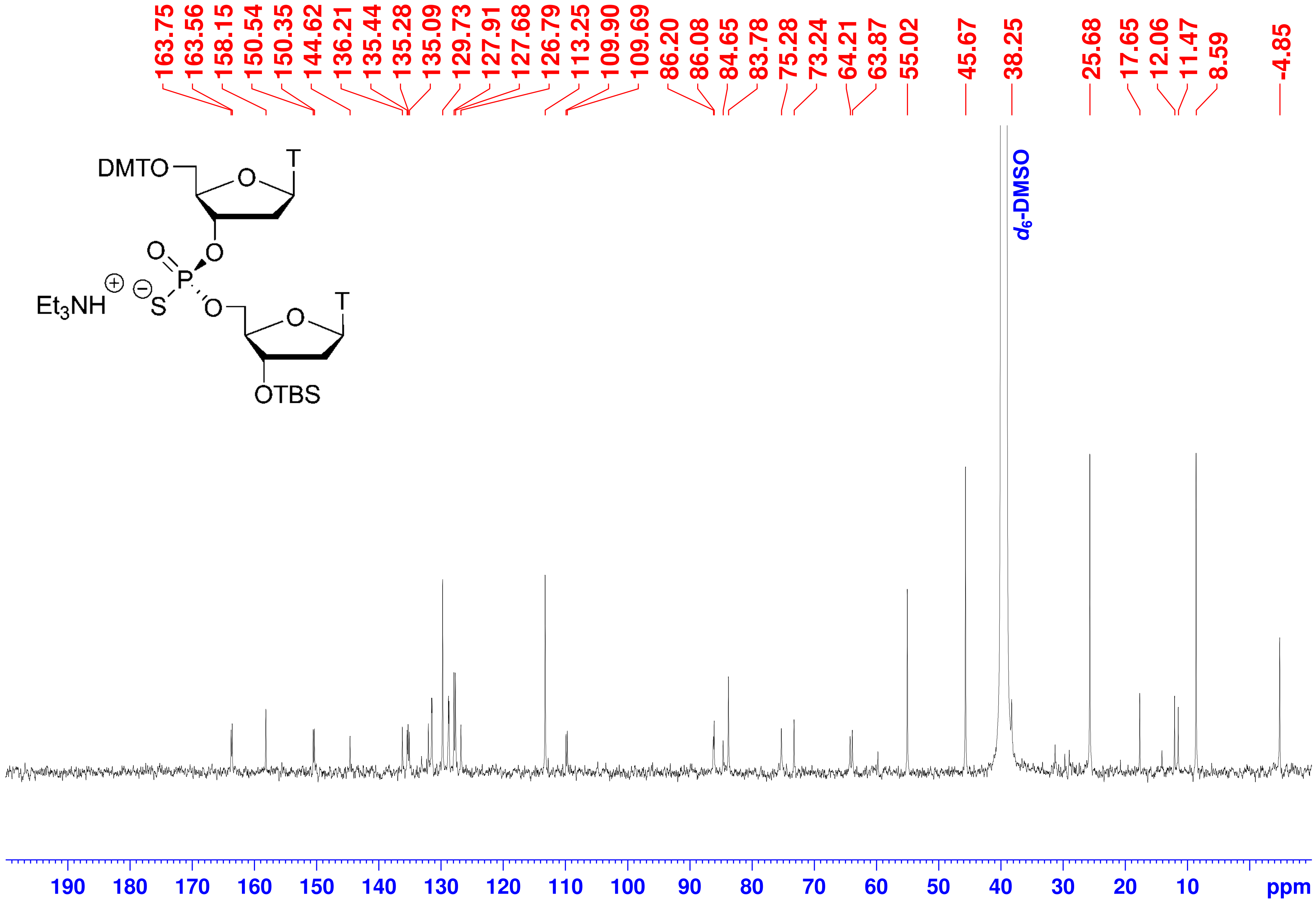

^{31}P NMR, 203 MHz, DMSO-d_6

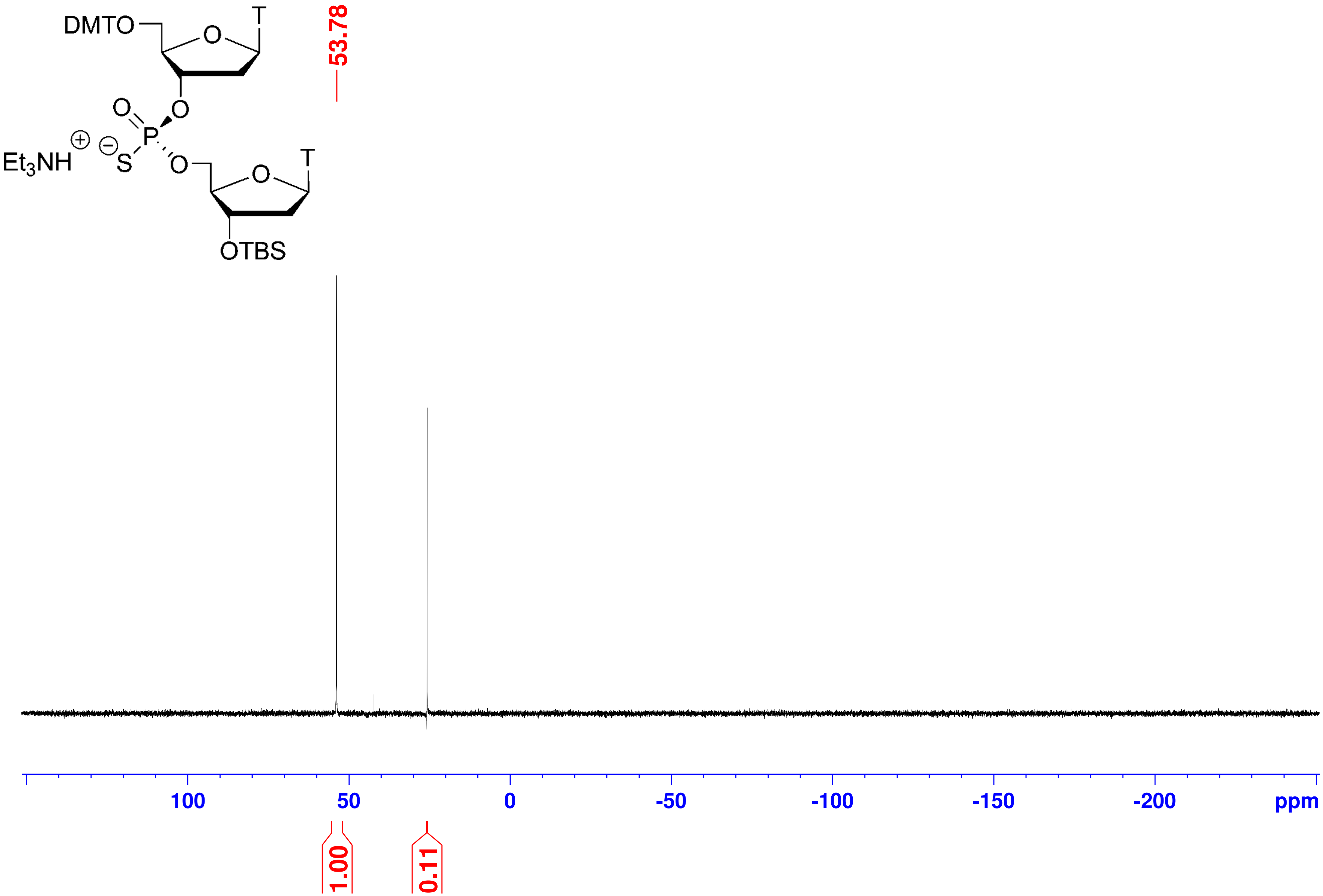

^{1}H NMR, 500 MHz, DMSO-d_6

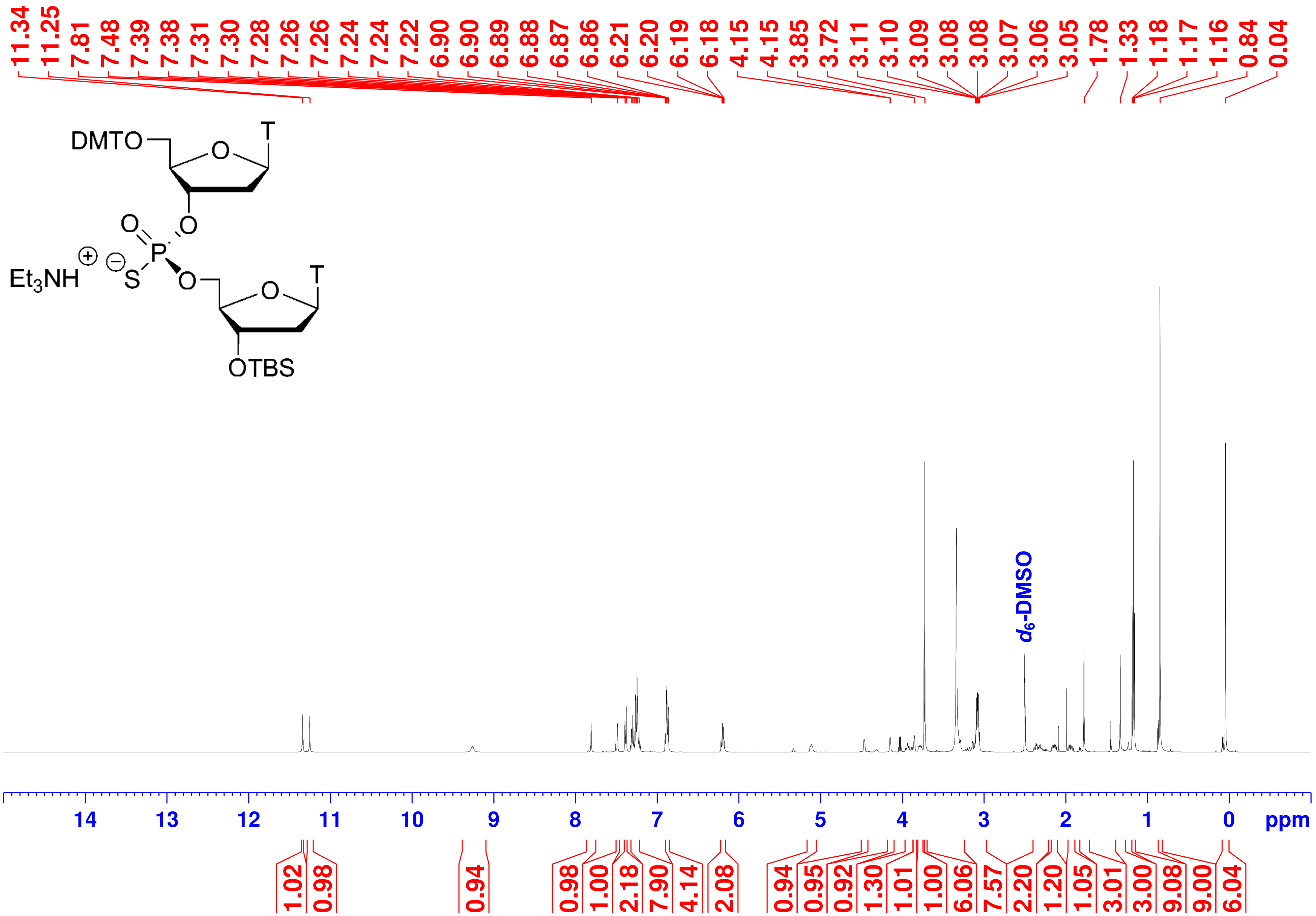

^{13}C NMR, 126 MHz, DMSO-d_6

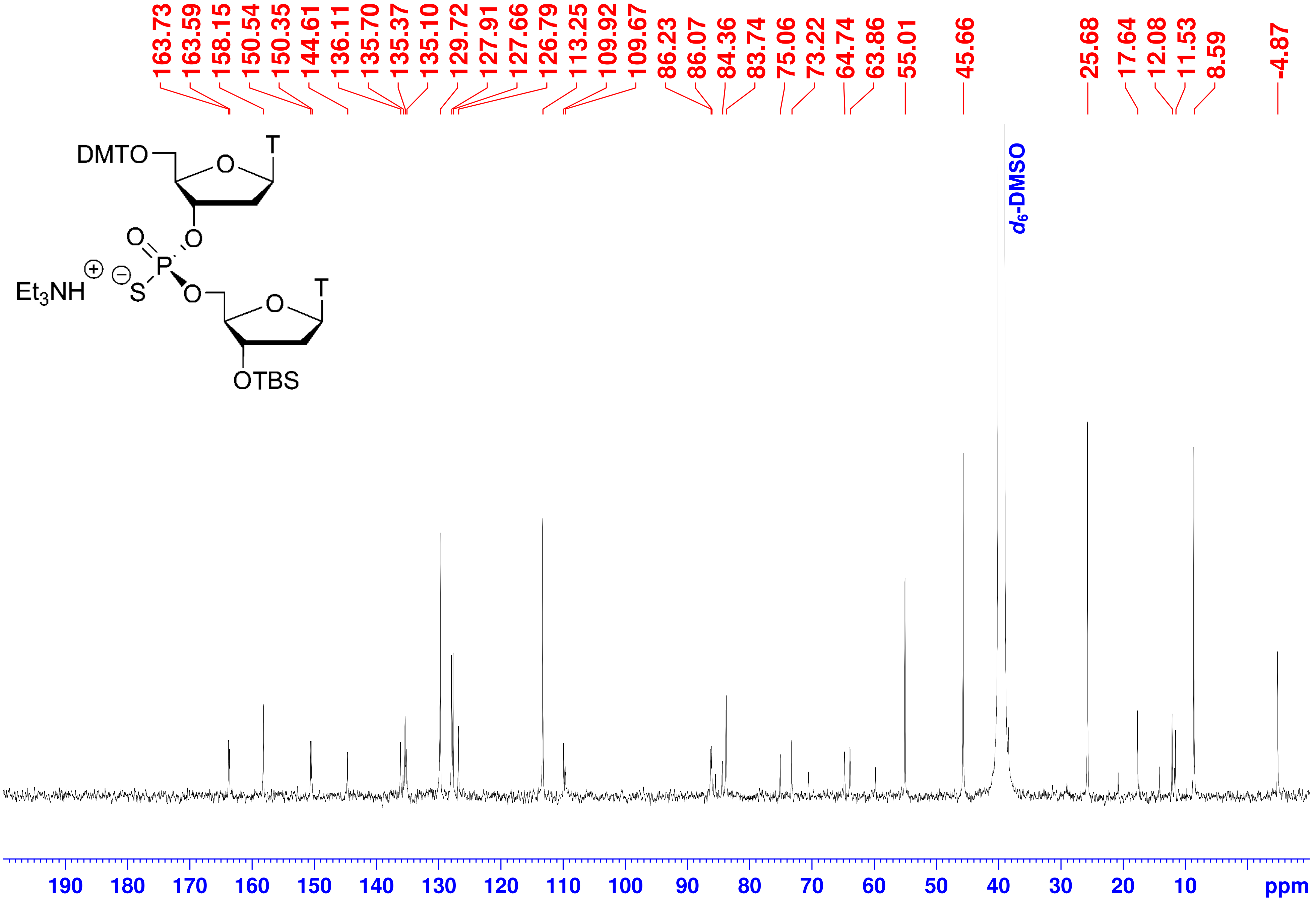

^{31}P NMR, 203 MHz, DMSO-d_6

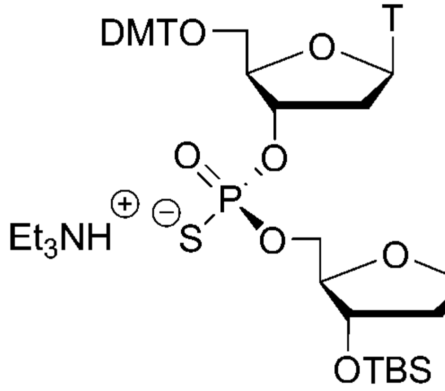

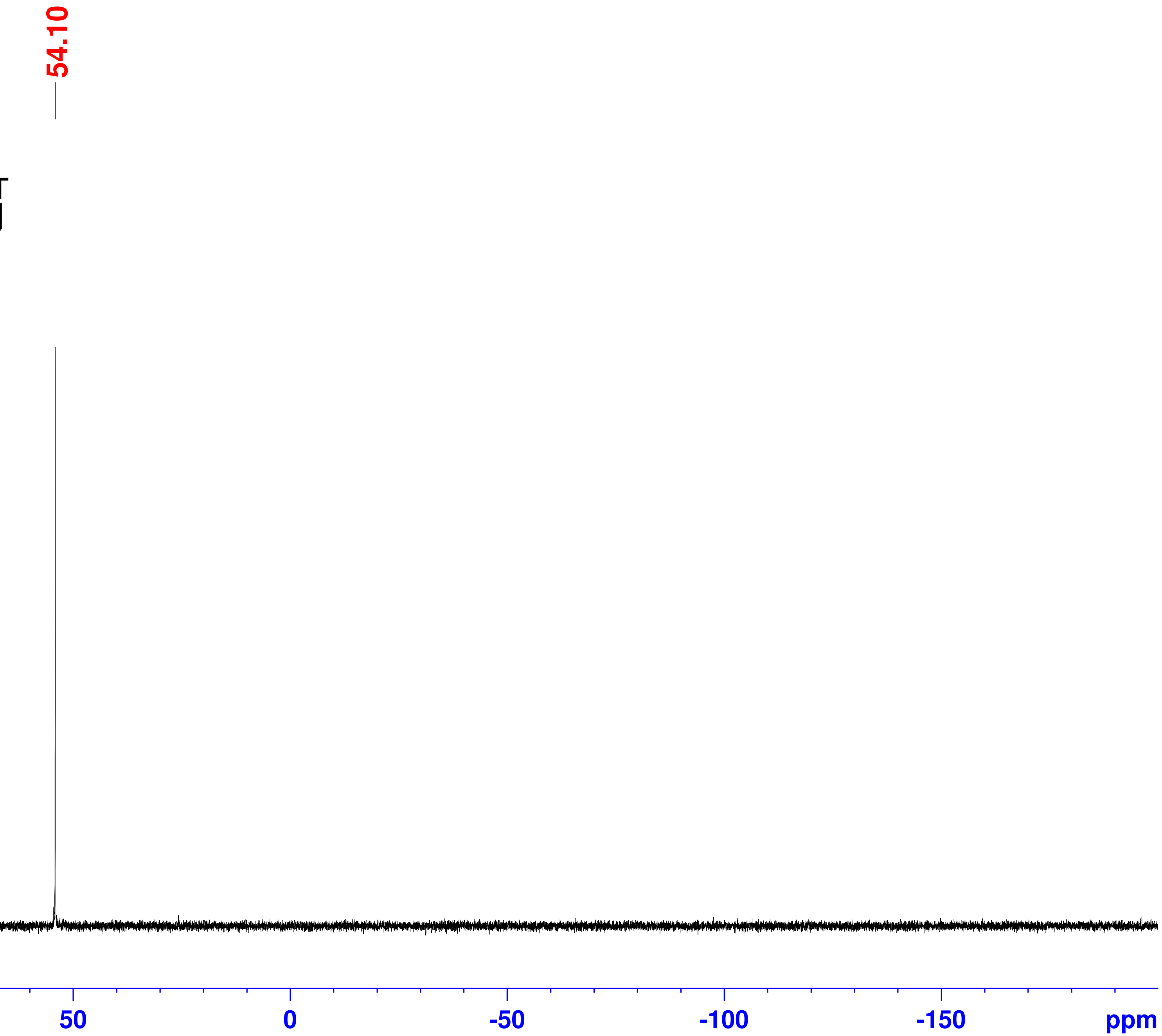

^{1}H NMR, 500 MHz, $CDCl_3$

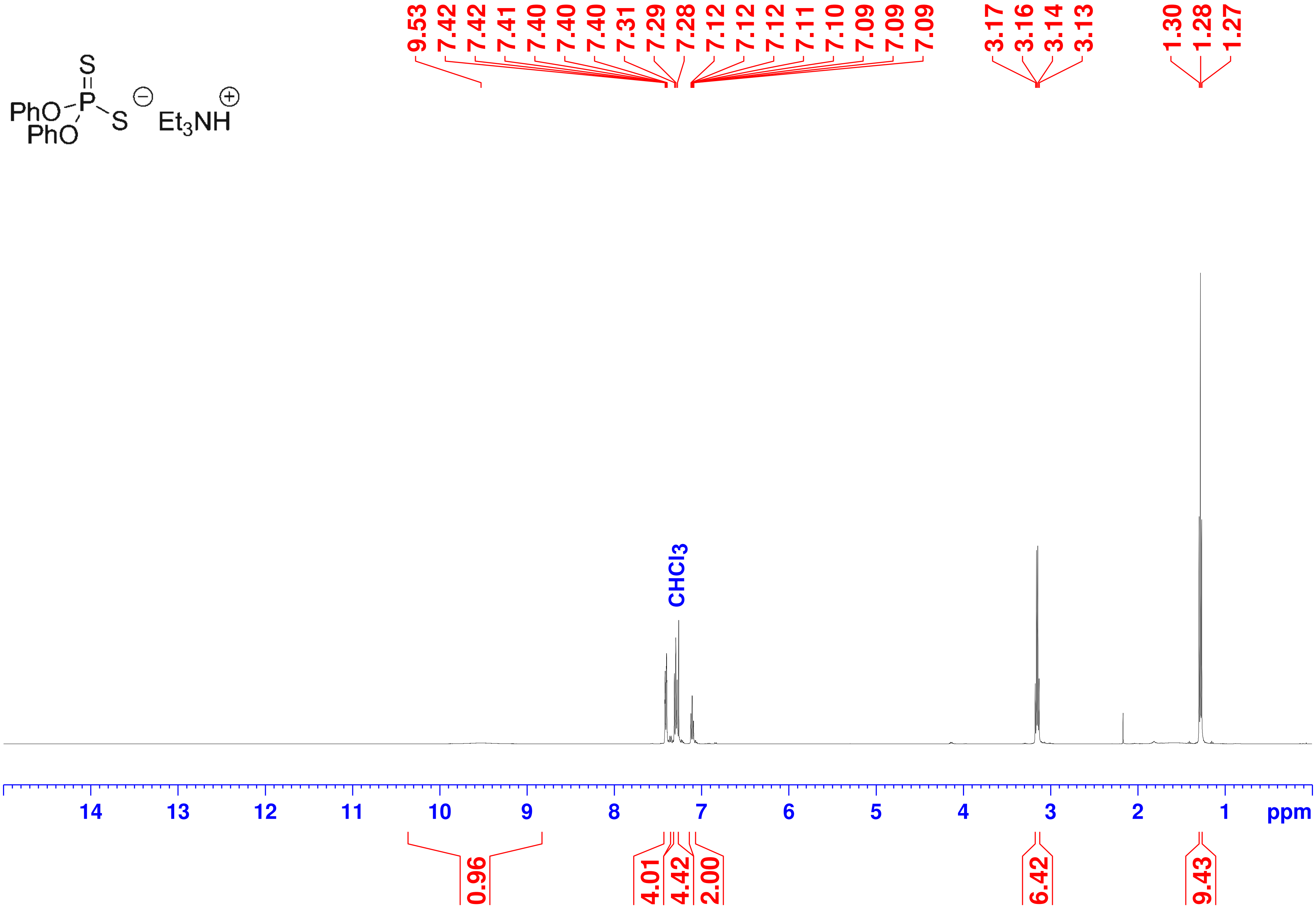

^{13}C NMR, 126 MHz, $CDCl_3$

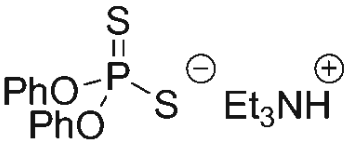

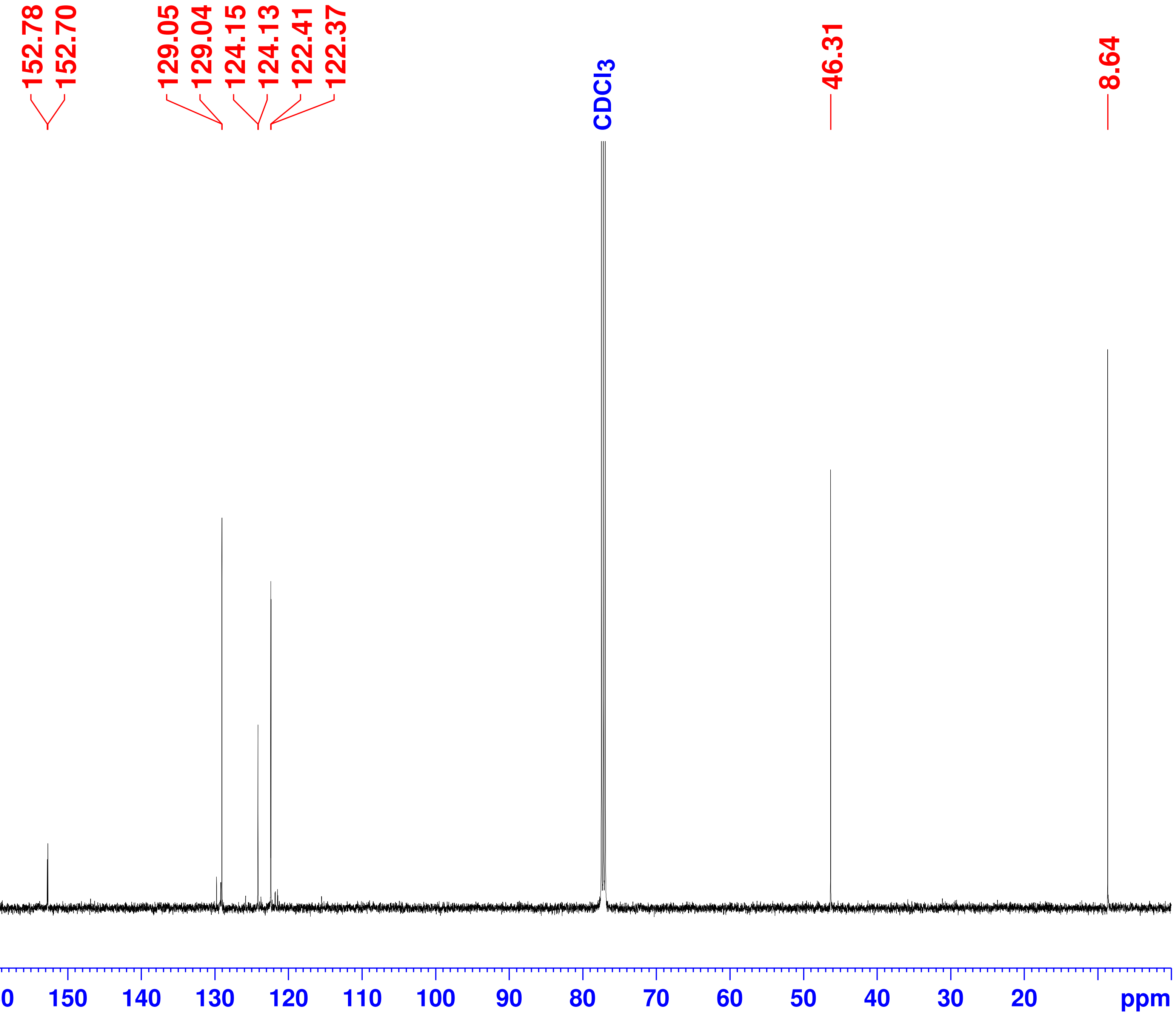

^{31}P NMR, 203 MHz, $CDCl_3$

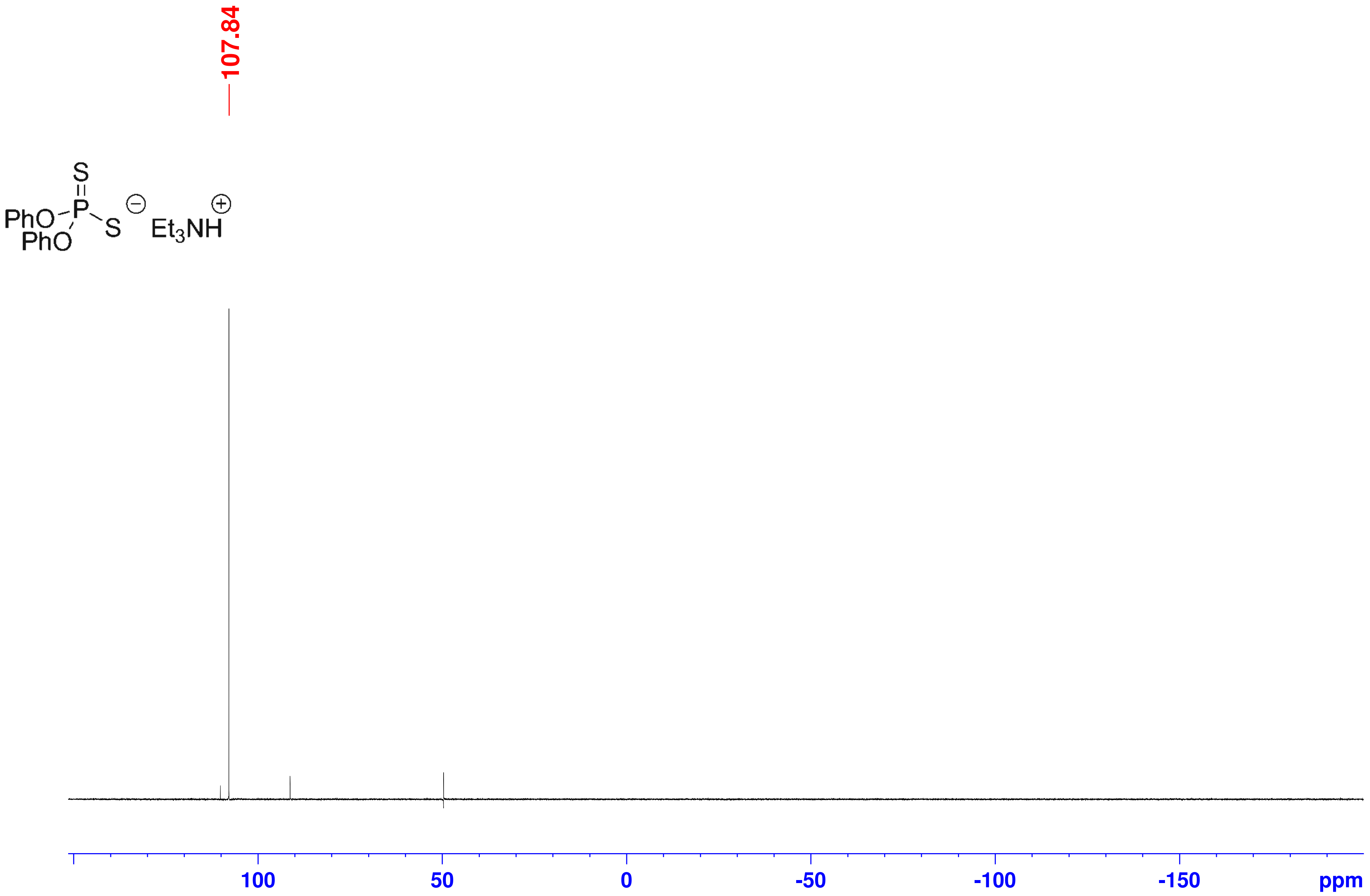

^{1}H NMR, 500 MHz, $CDCl_3$

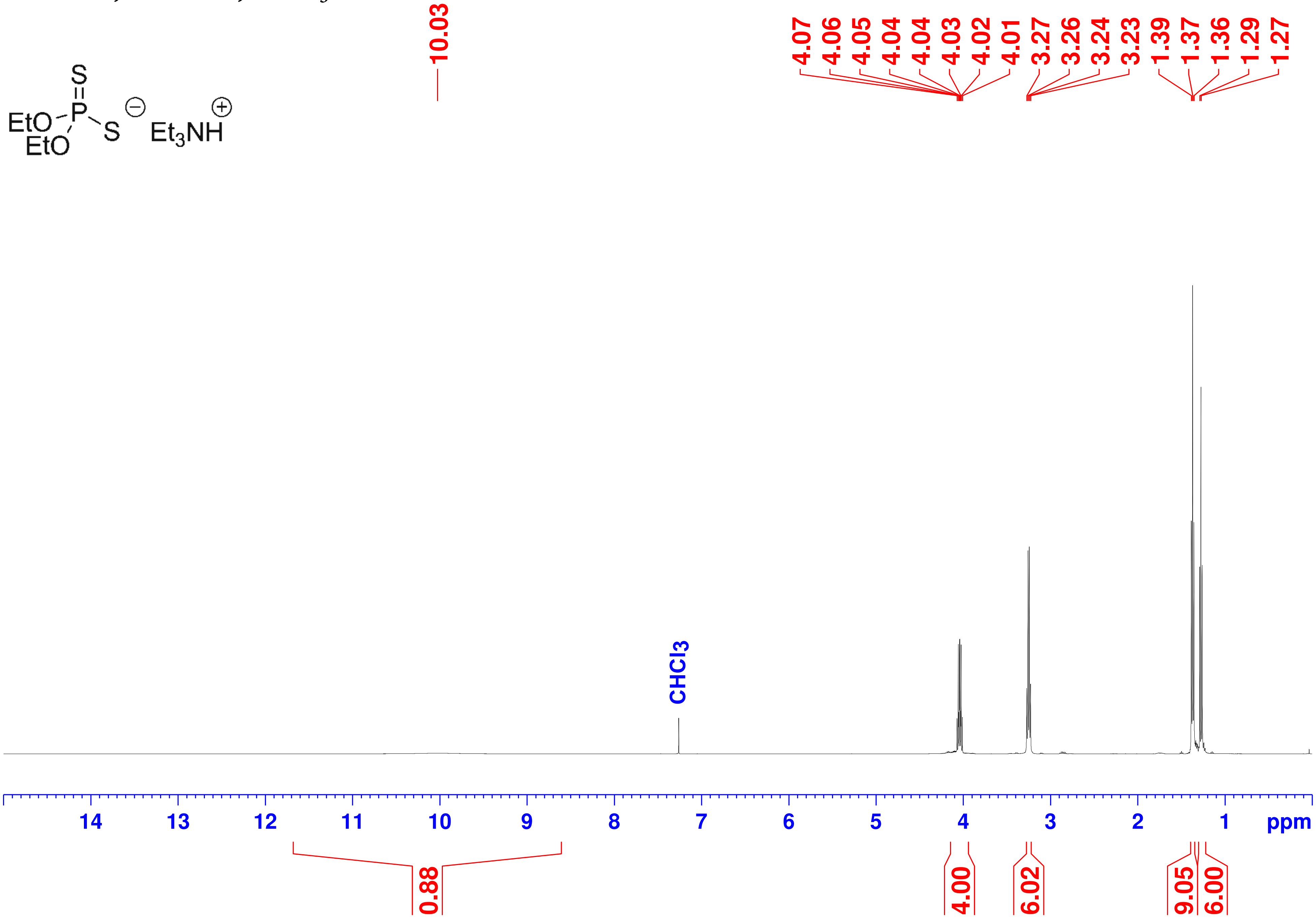

^{13}C NMR, 126 MHz, $CDCl_3$

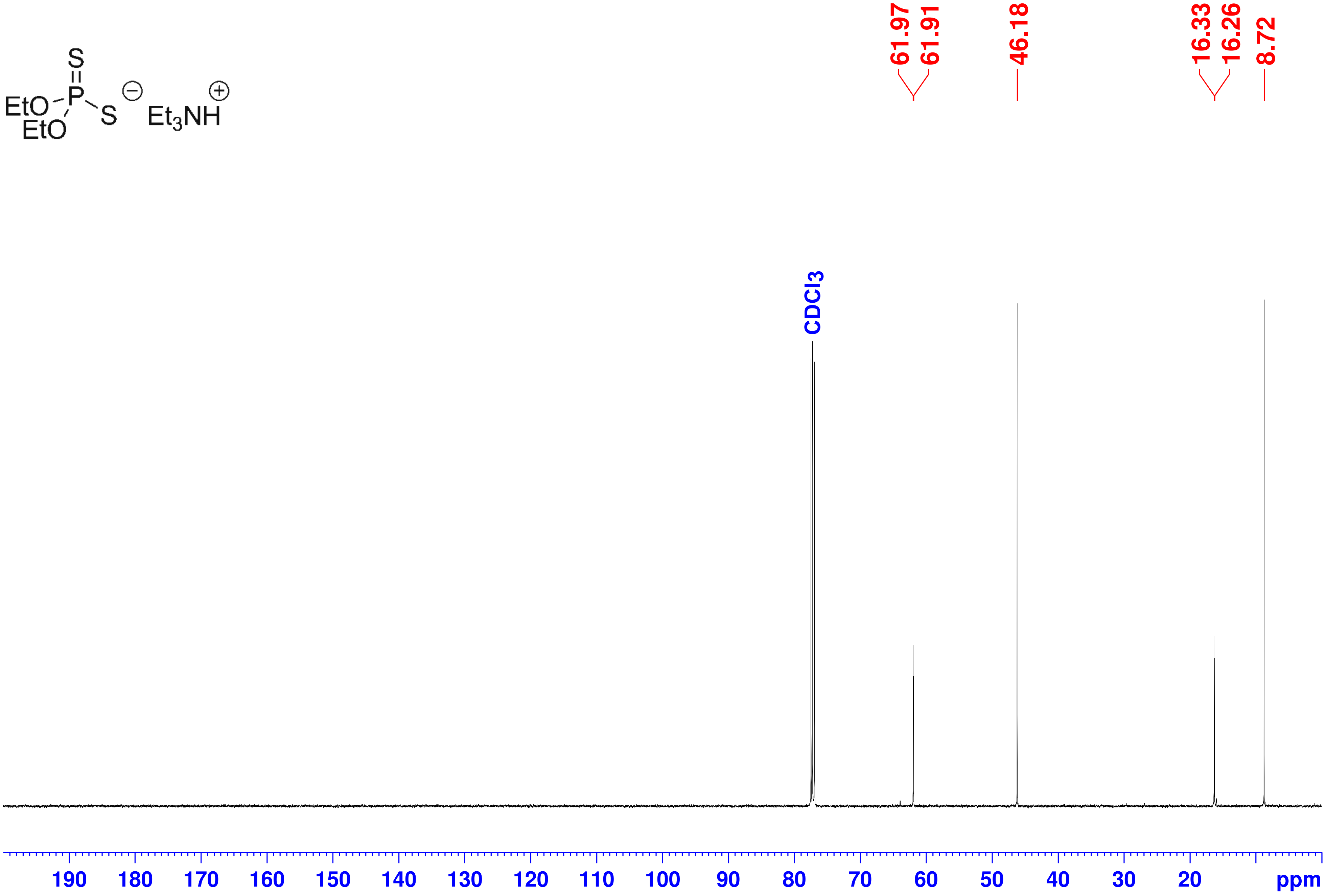

^{31}P NMR, 203 MHz, $CDCl_3$

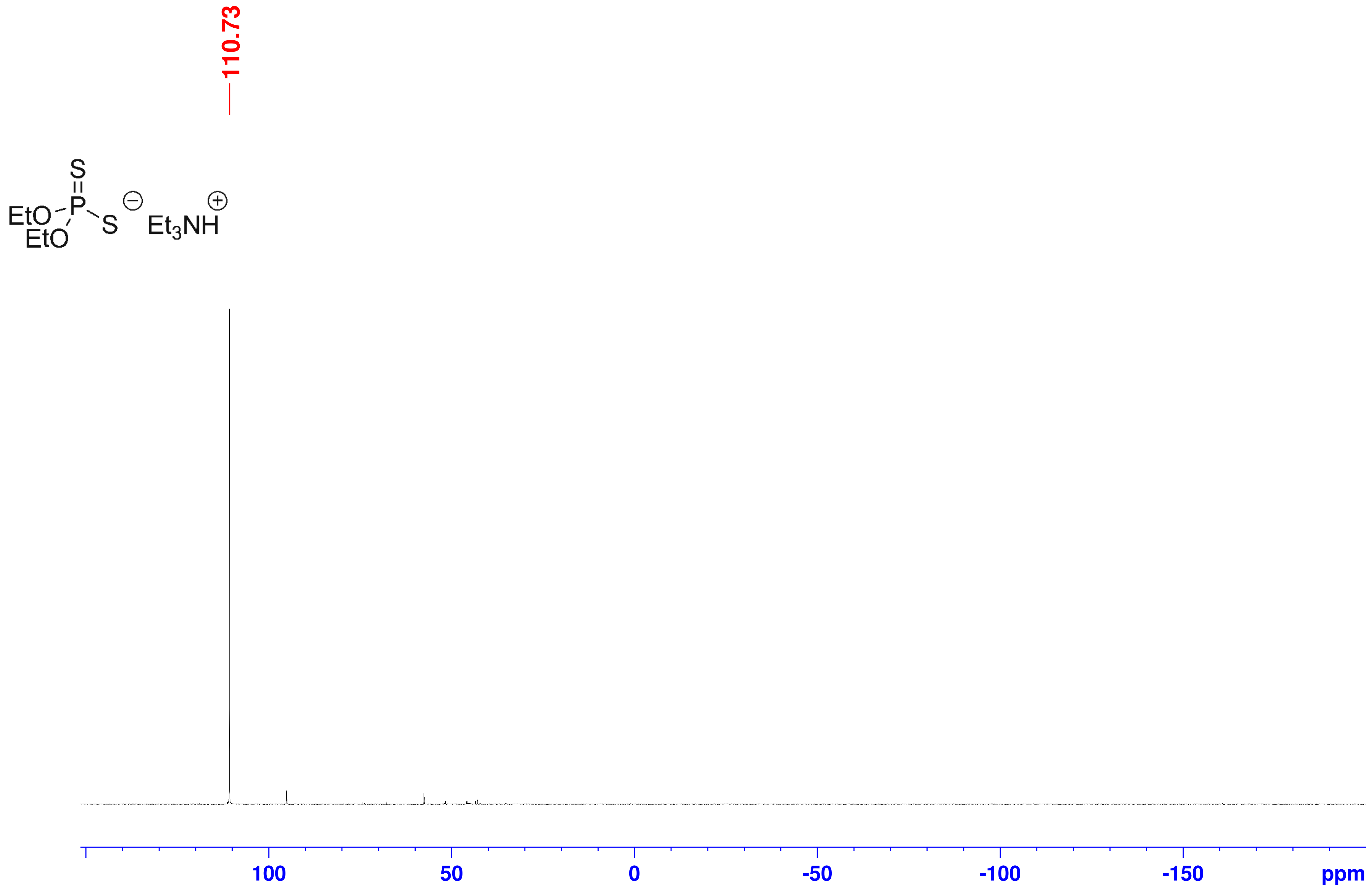

^{1}H NMR, 500 MHz, $CDCl_3$

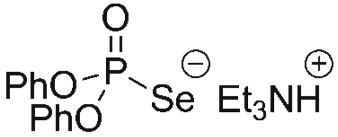

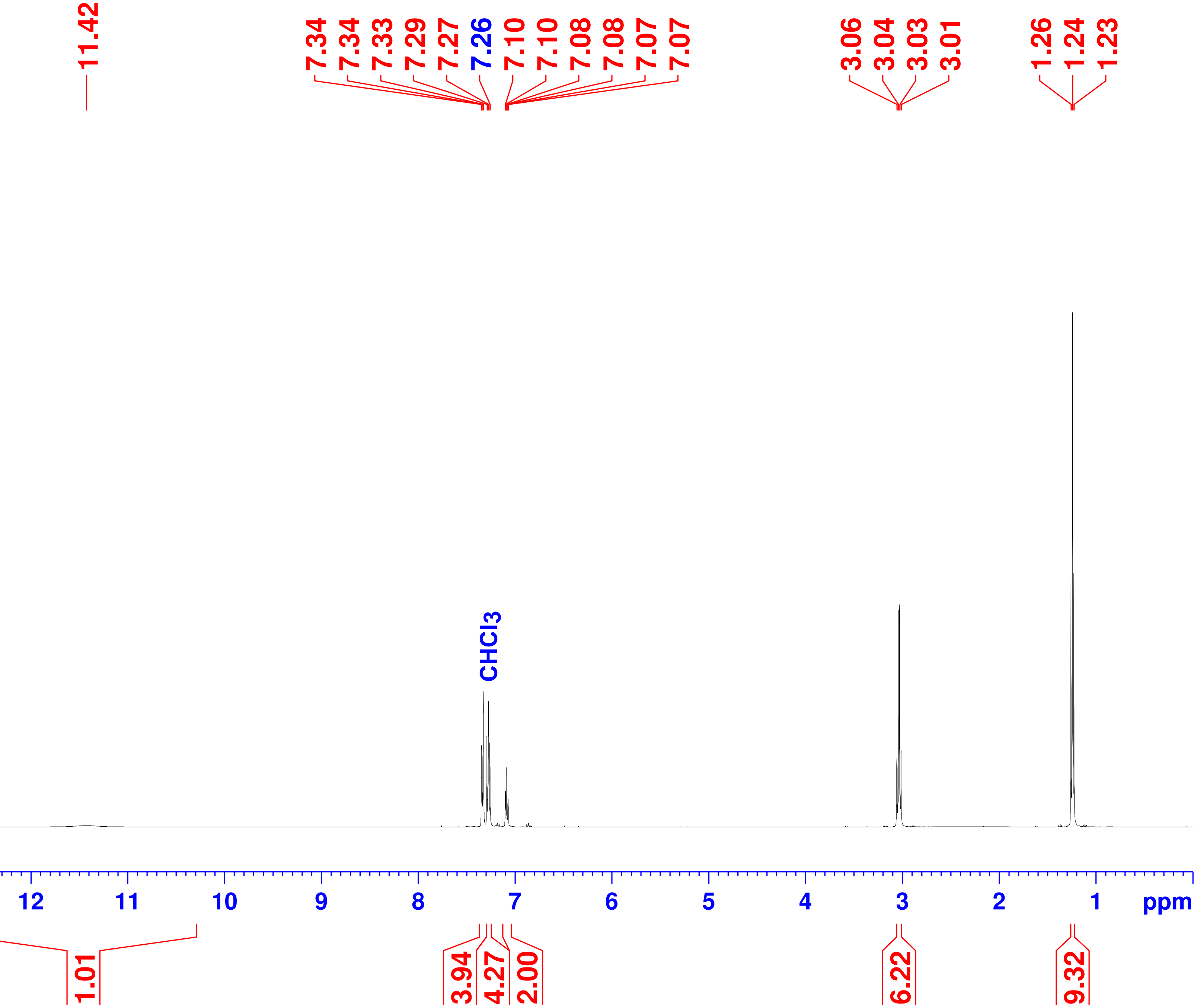

^{13}C NMR, 126 MHz, $CDCl_3$

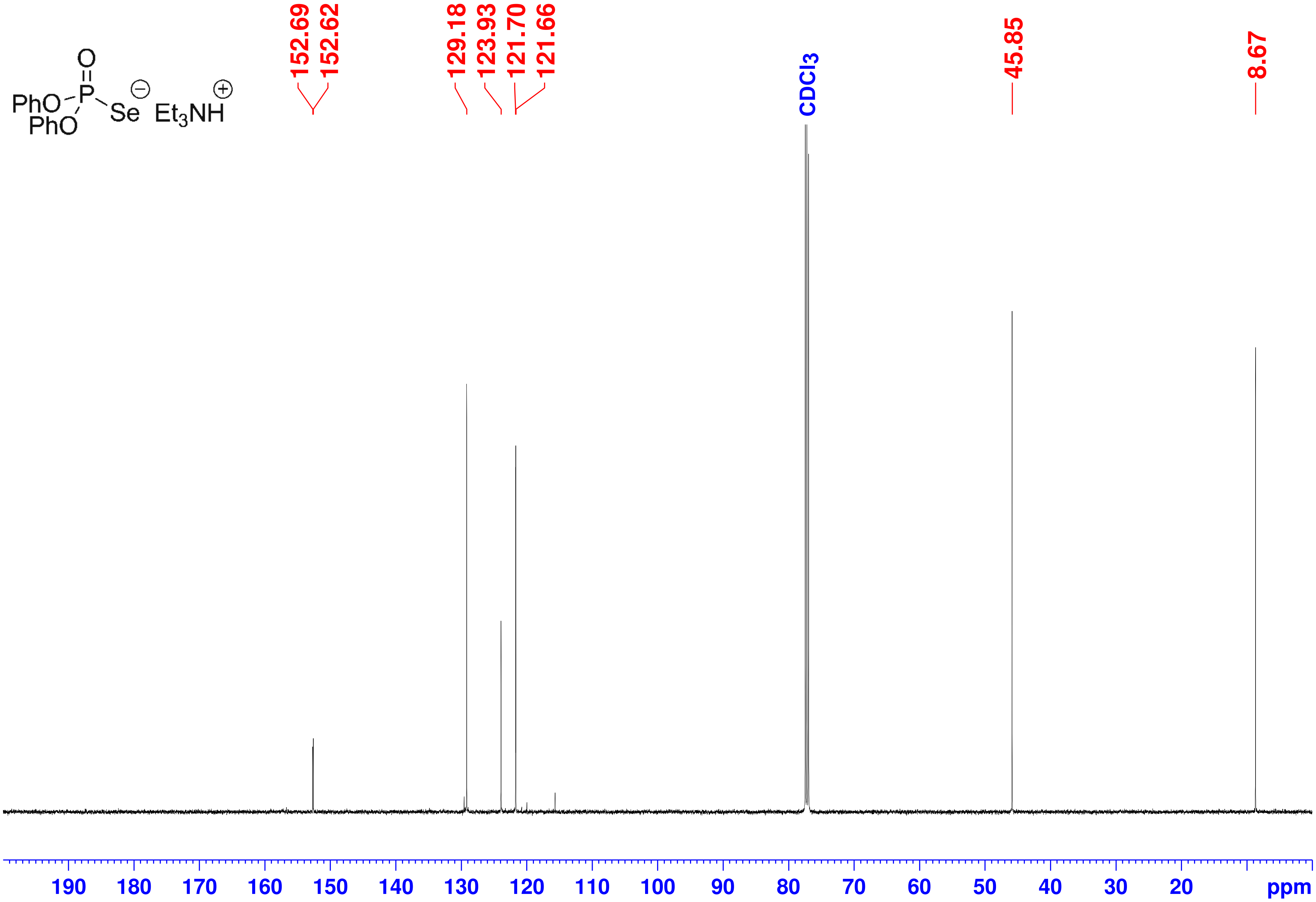

^{31}P NMR, 203 MHz, $CDCl_3$

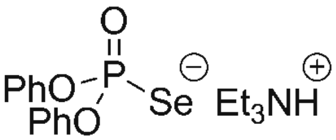

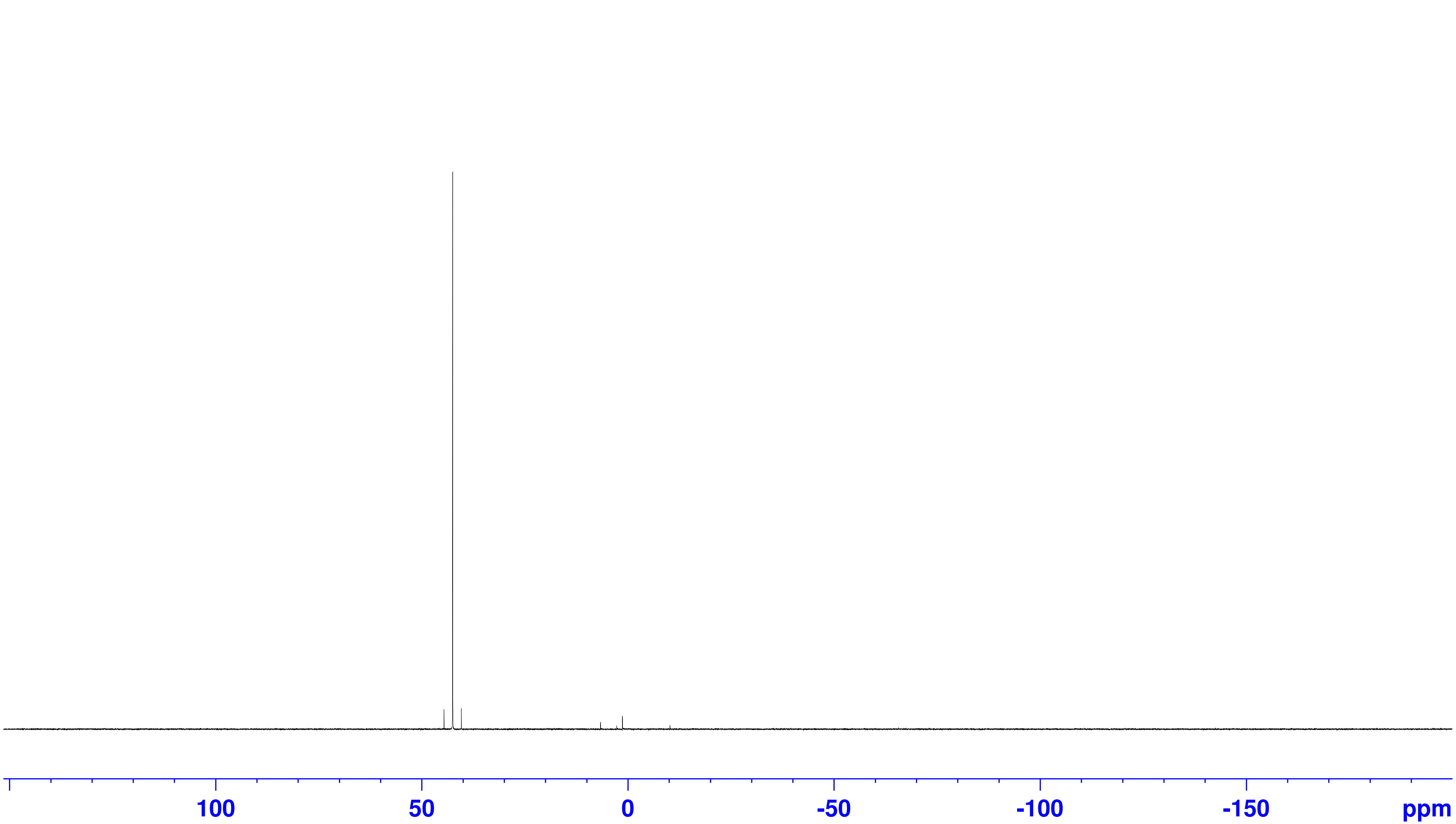

^1H NMR, 500 MHz, $CDCl_3$

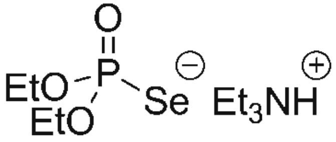

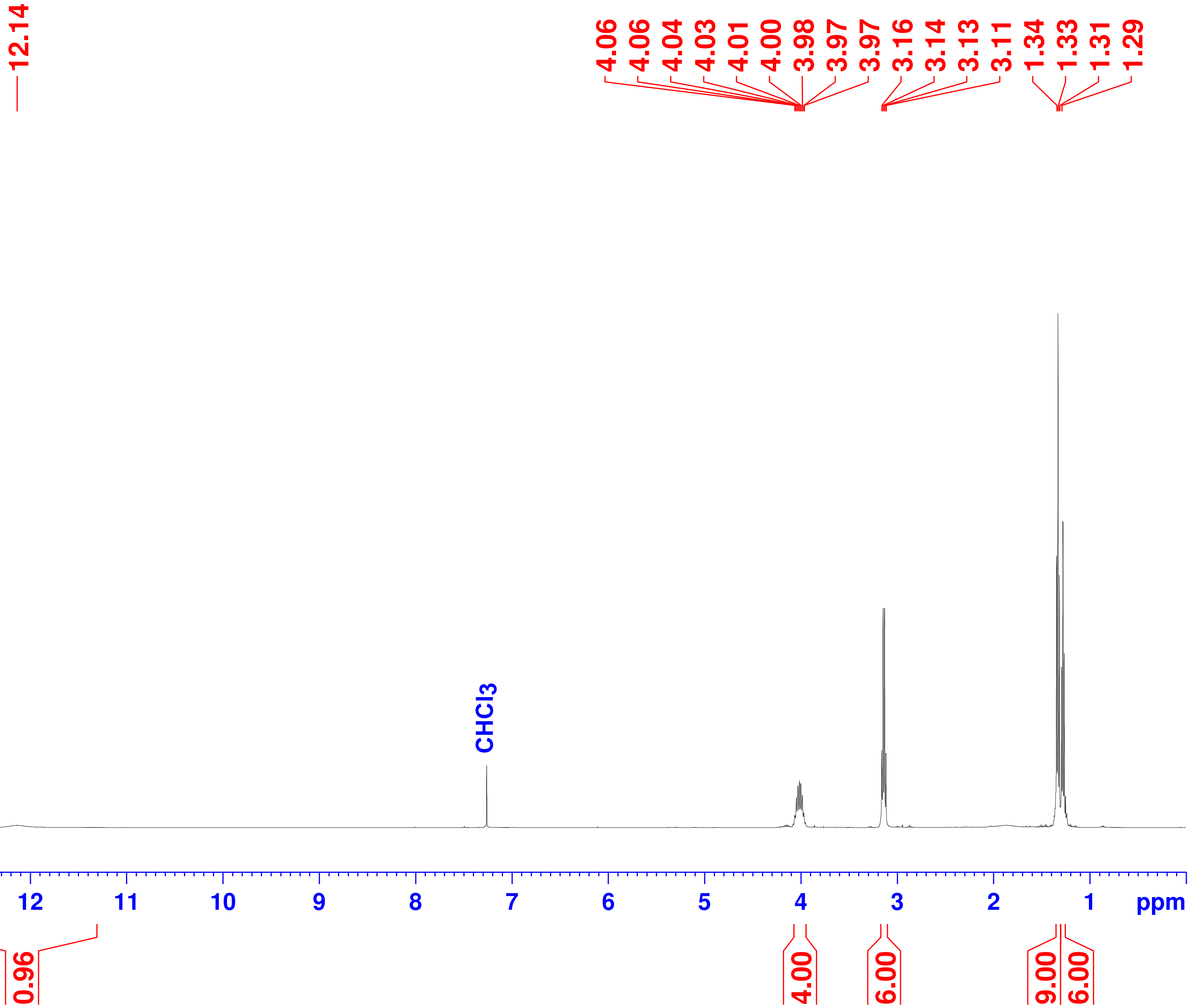

^{13}C NMR, 126 MHz, $CDCl_3$

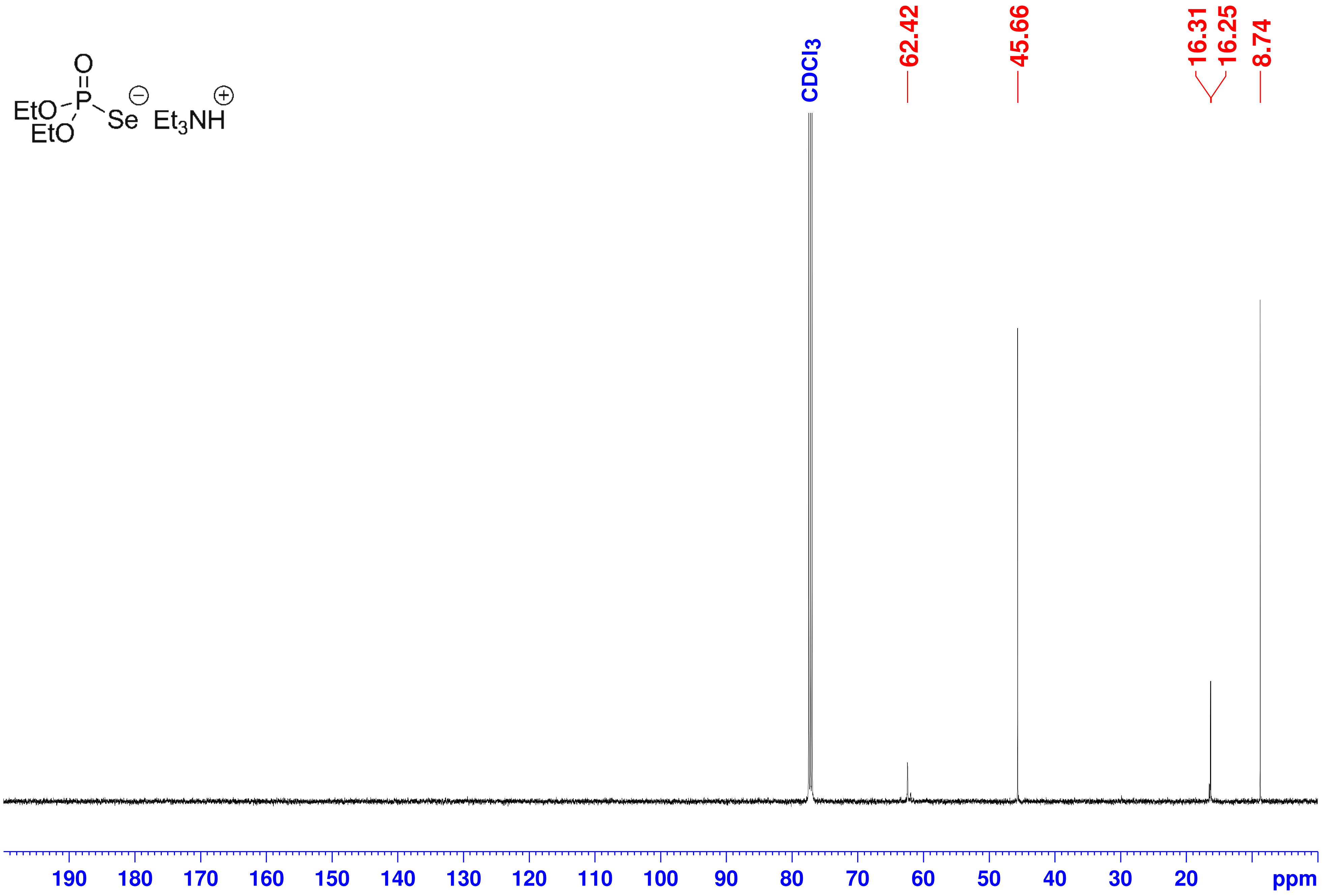

^{31}P NMR, 162 MHz, $CDCl_3$

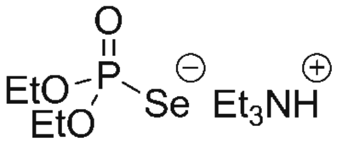

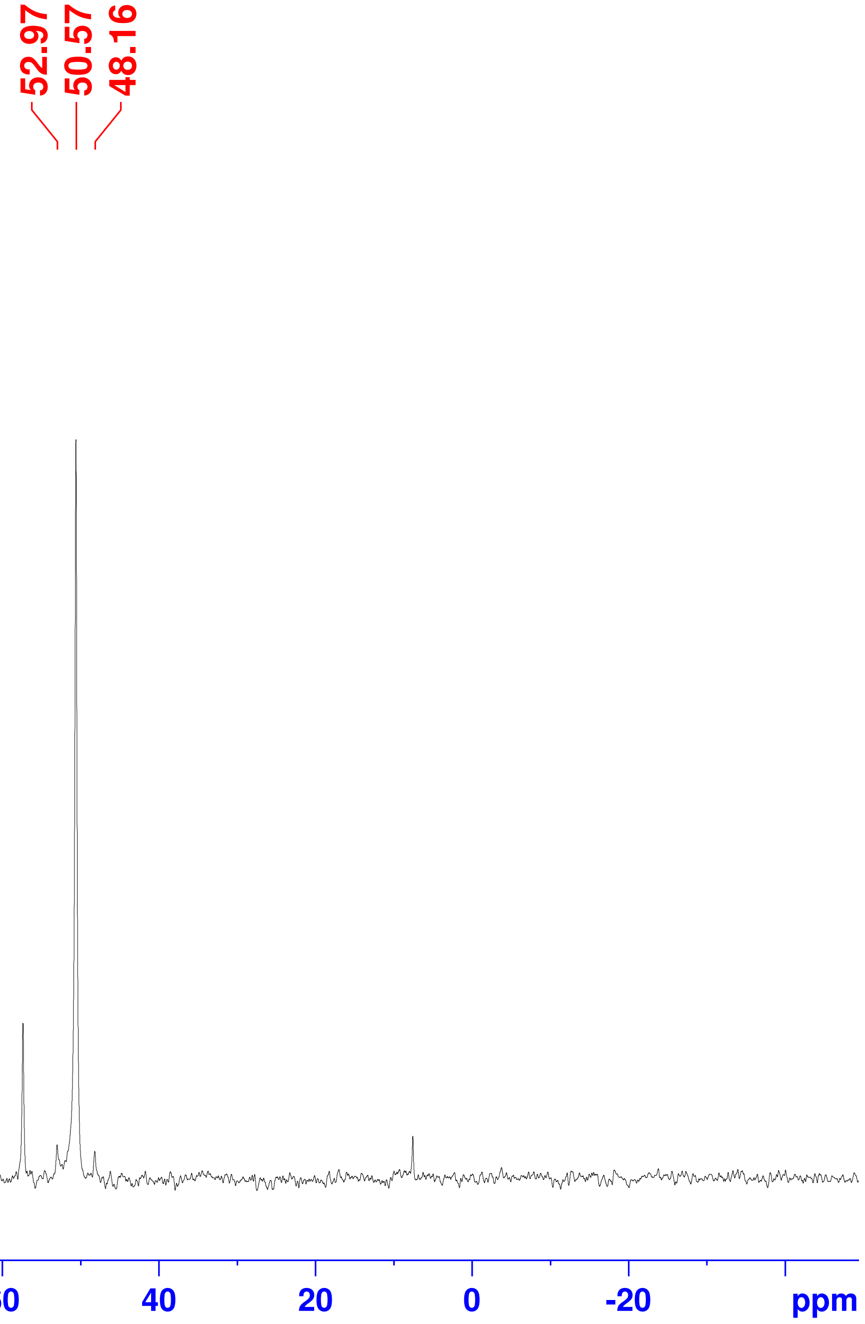

^{1}H NMR, 500 MHz, $CDCl_3$

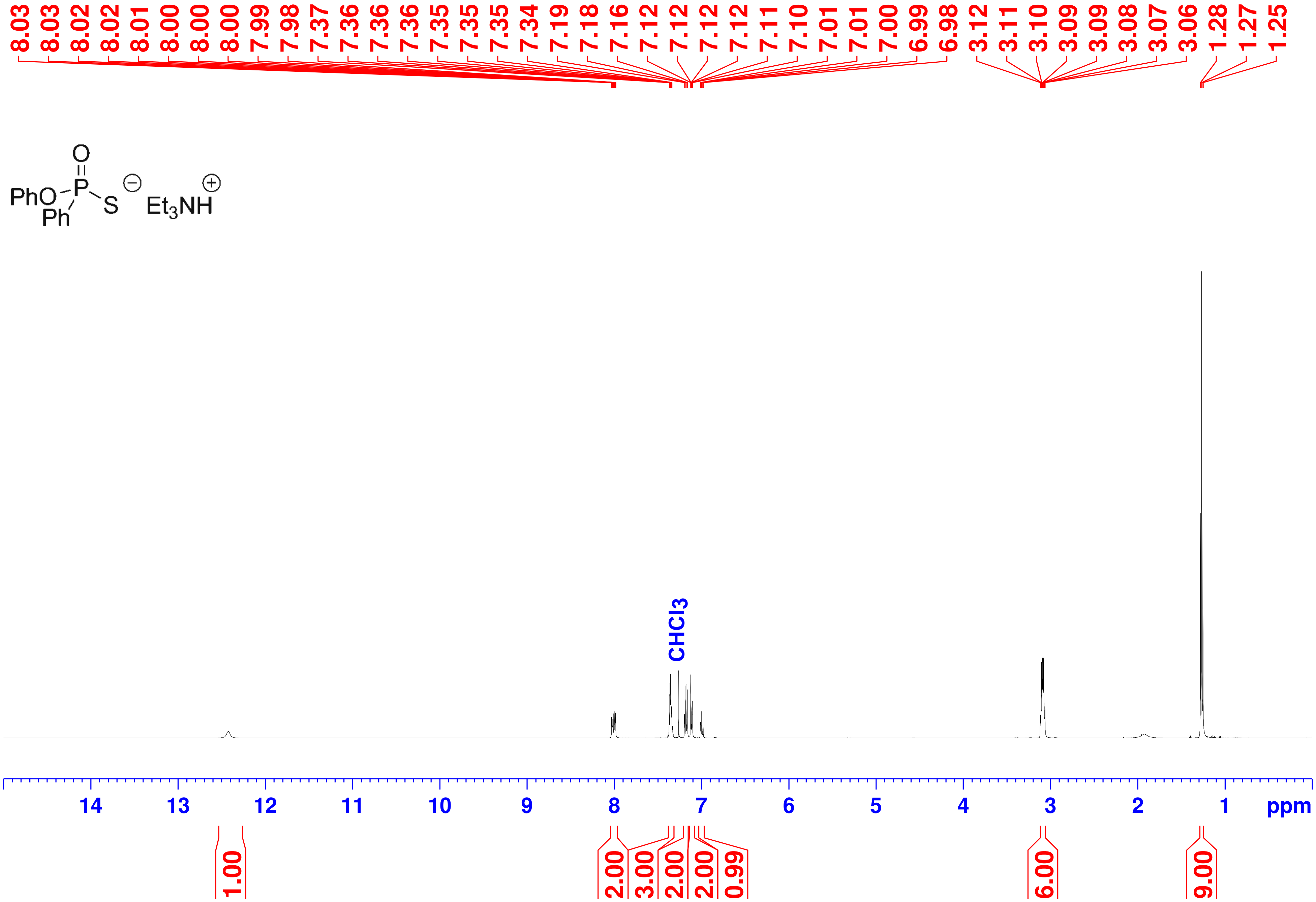

^{13}C NMR, 126 MHz, $CDCl_3$

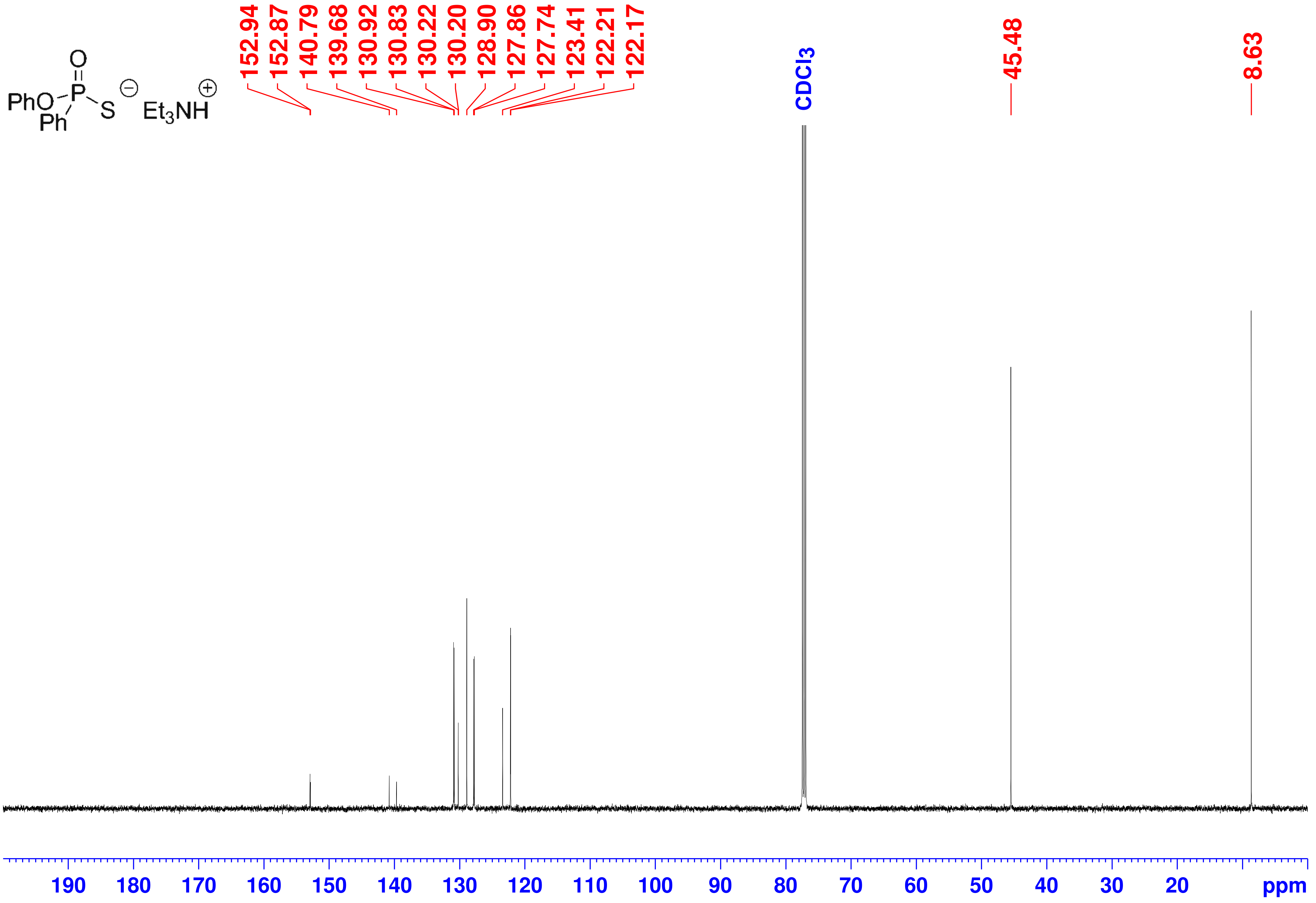

^{31}P NMR, 203 MHz, $CDCl_3$

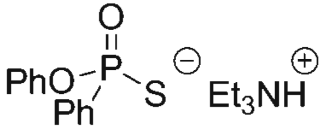

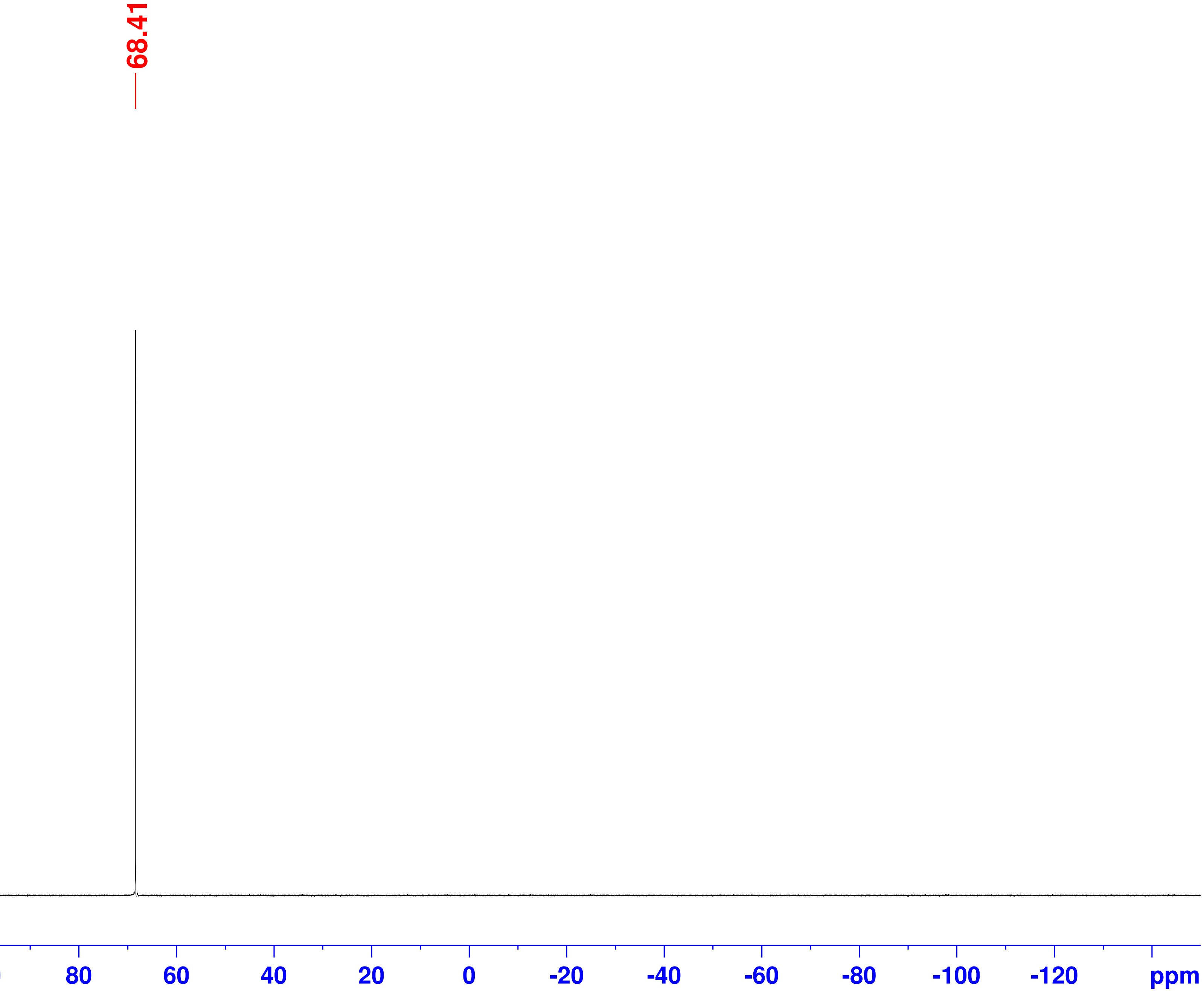

^{1}H NMR, 500 MHz, $CDCl_3$

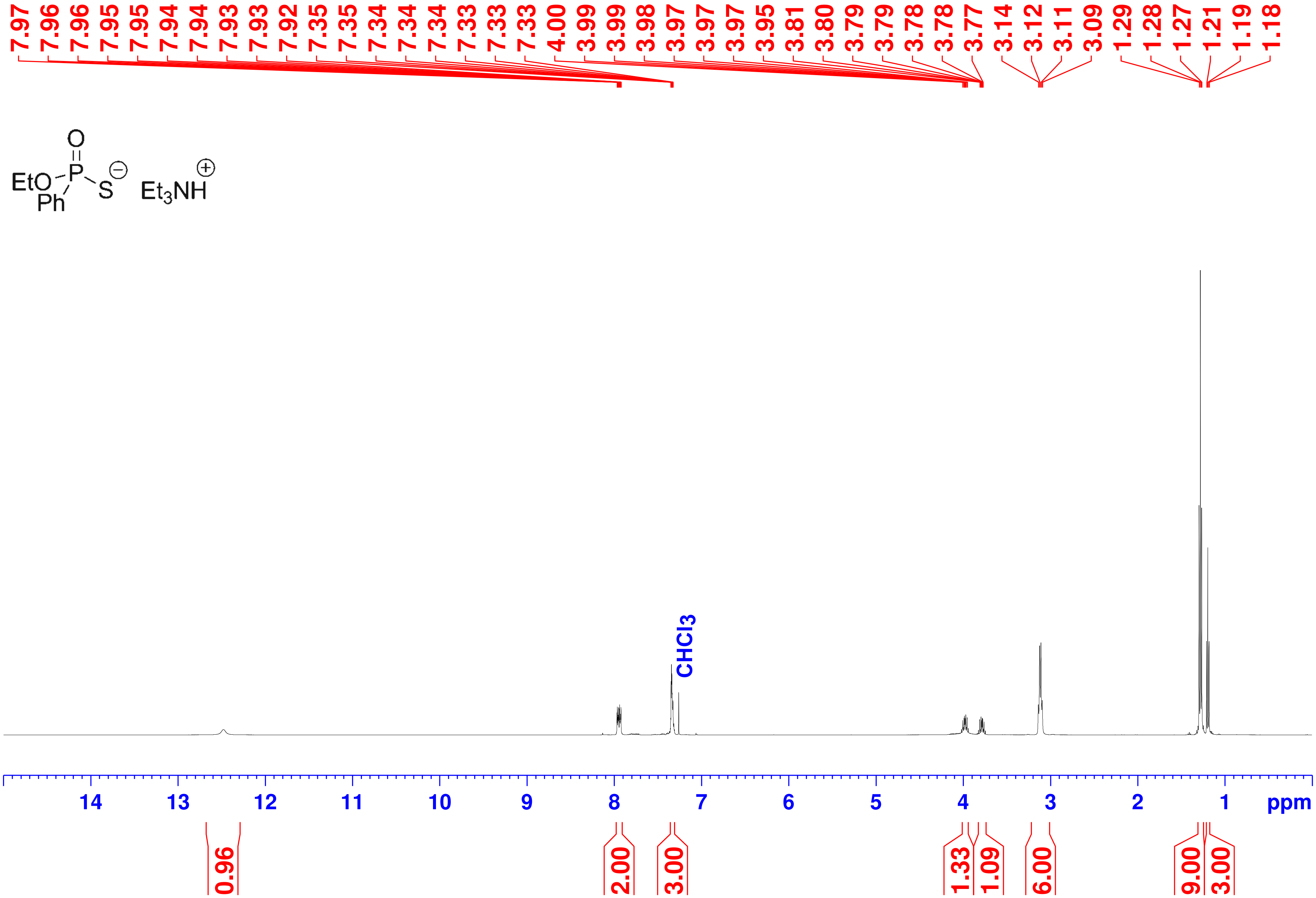

^{13}C NMR, 126 MHz, $CDCl_3$

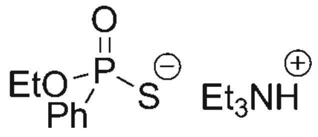

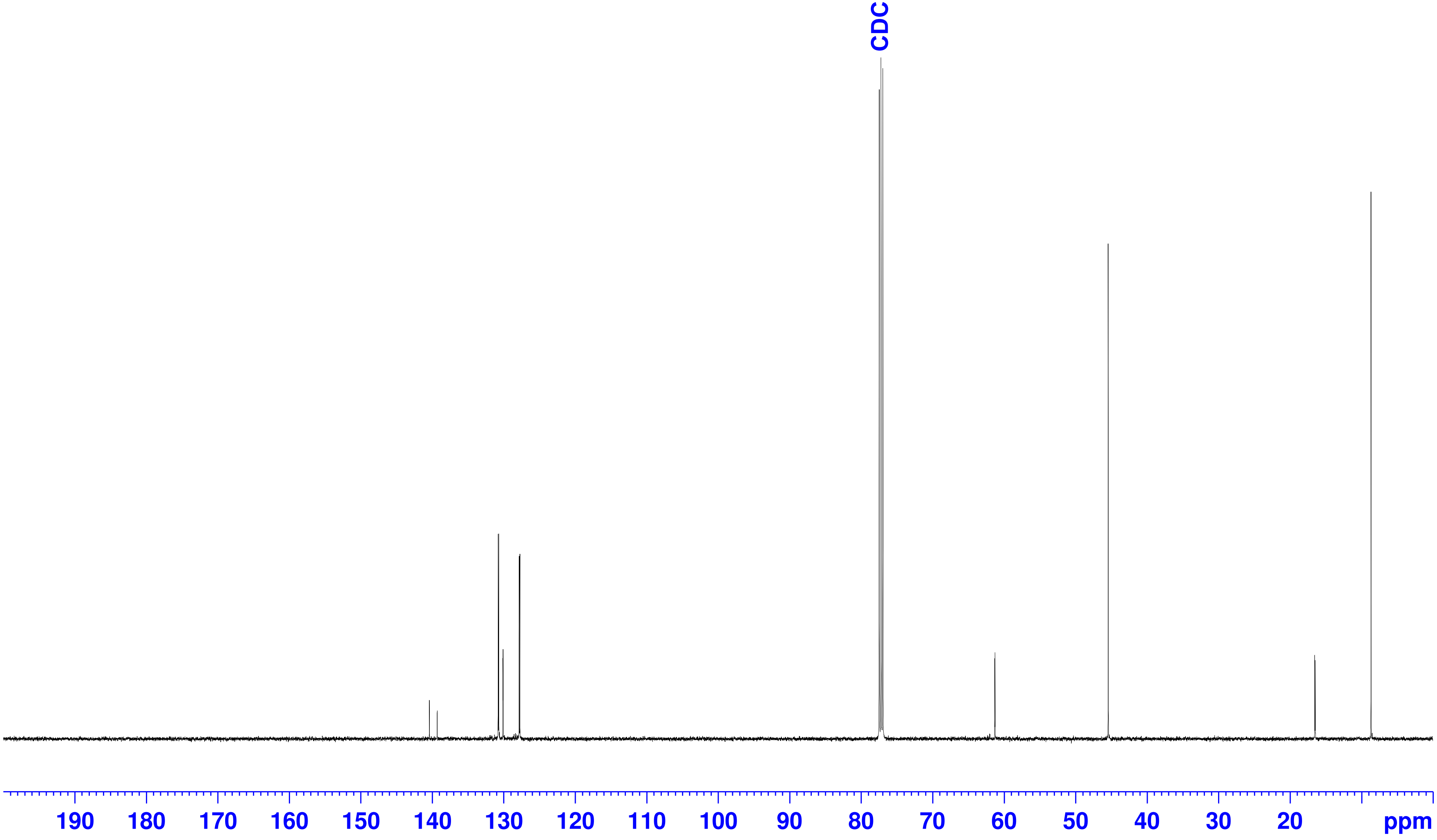

^{31}P NMR, 203 MHz, $CDCl_3$

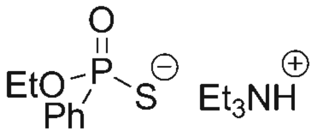

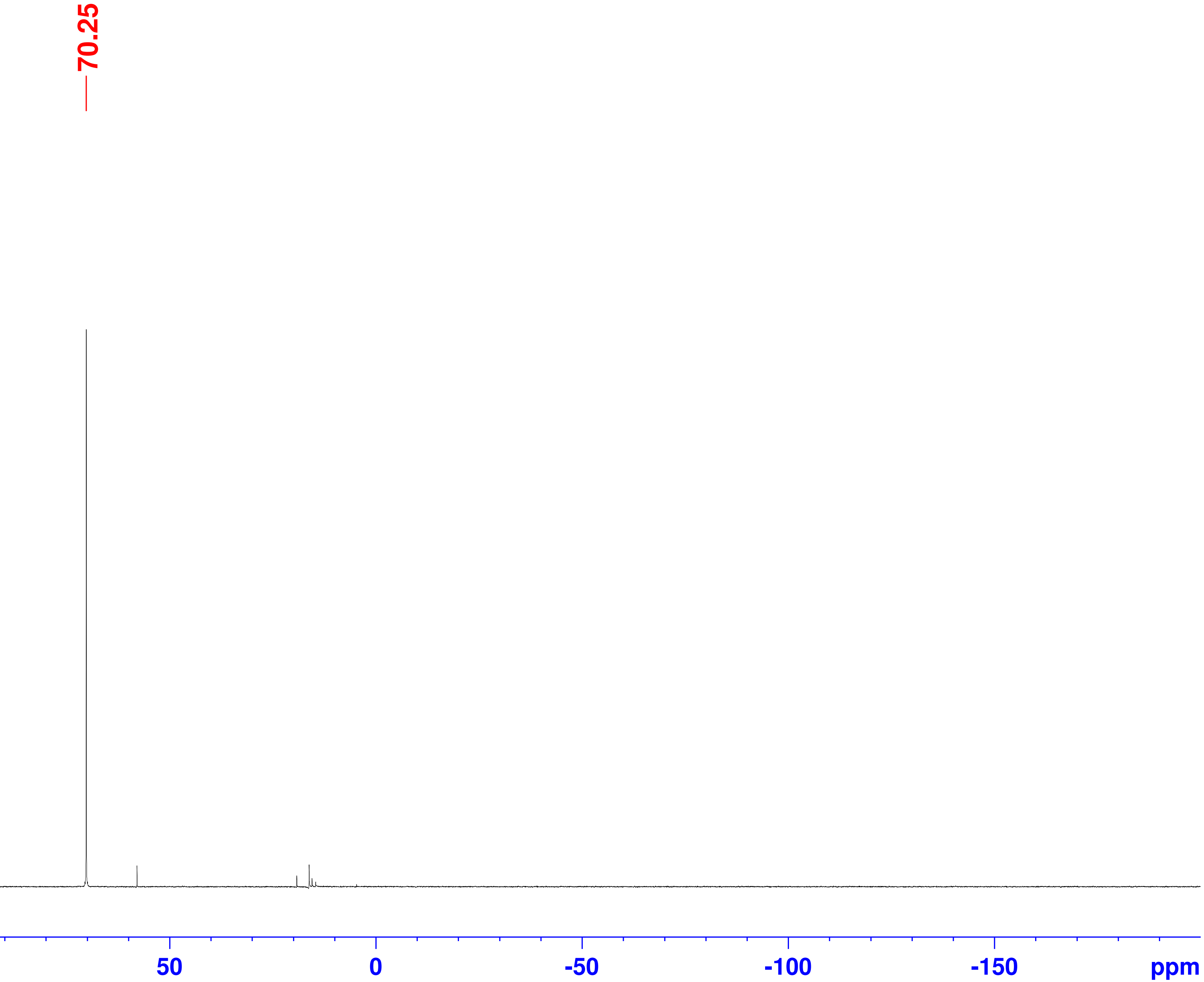

^{1}H NMR, 500 MHz, $CDCl_3$

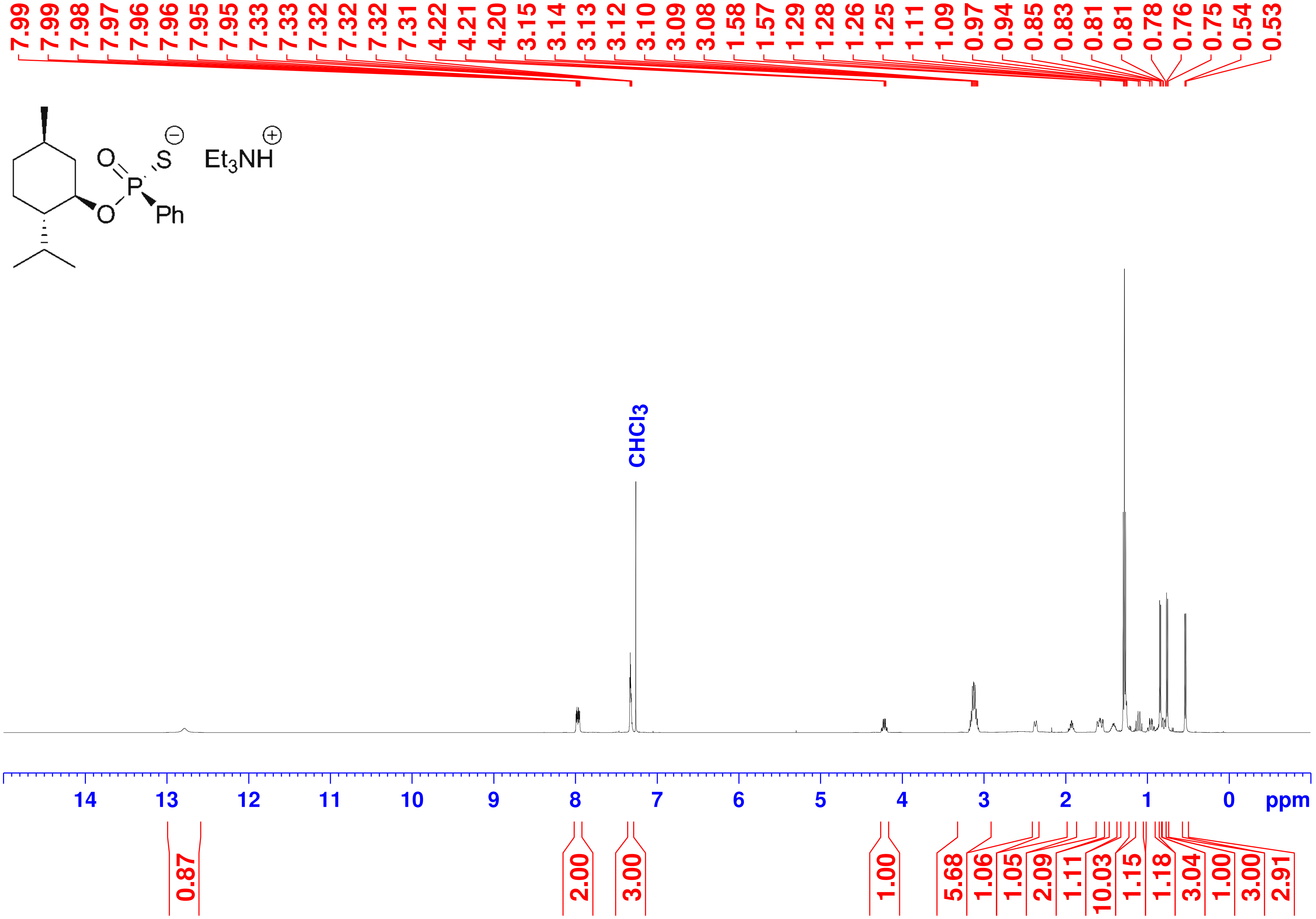

^{13}C NMR, 126 MHz, $CDCl_3$

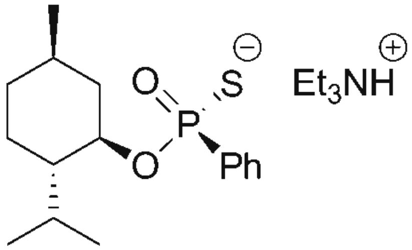

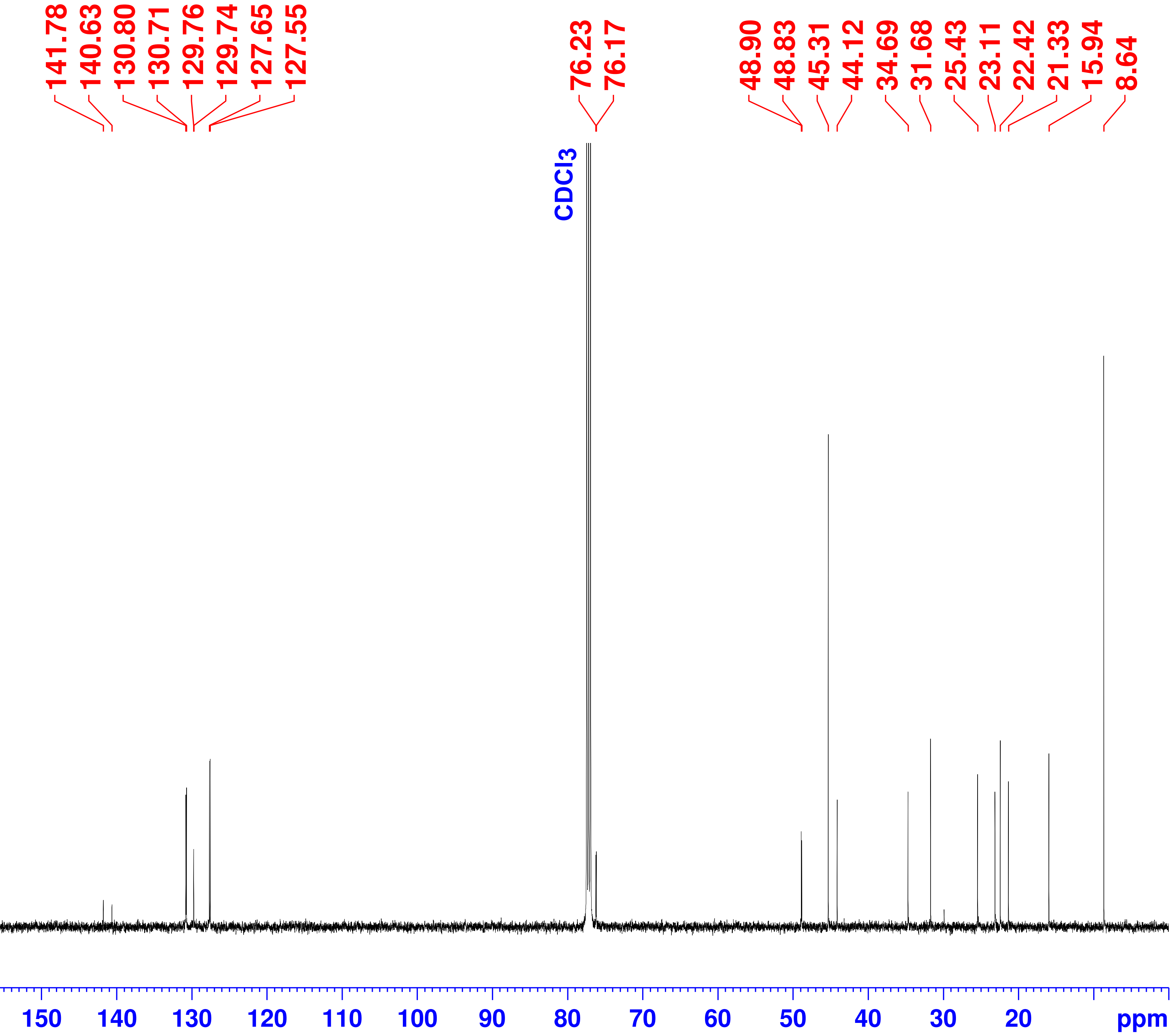

^{31}P NMR, 203 MHz, $CDCl_3$

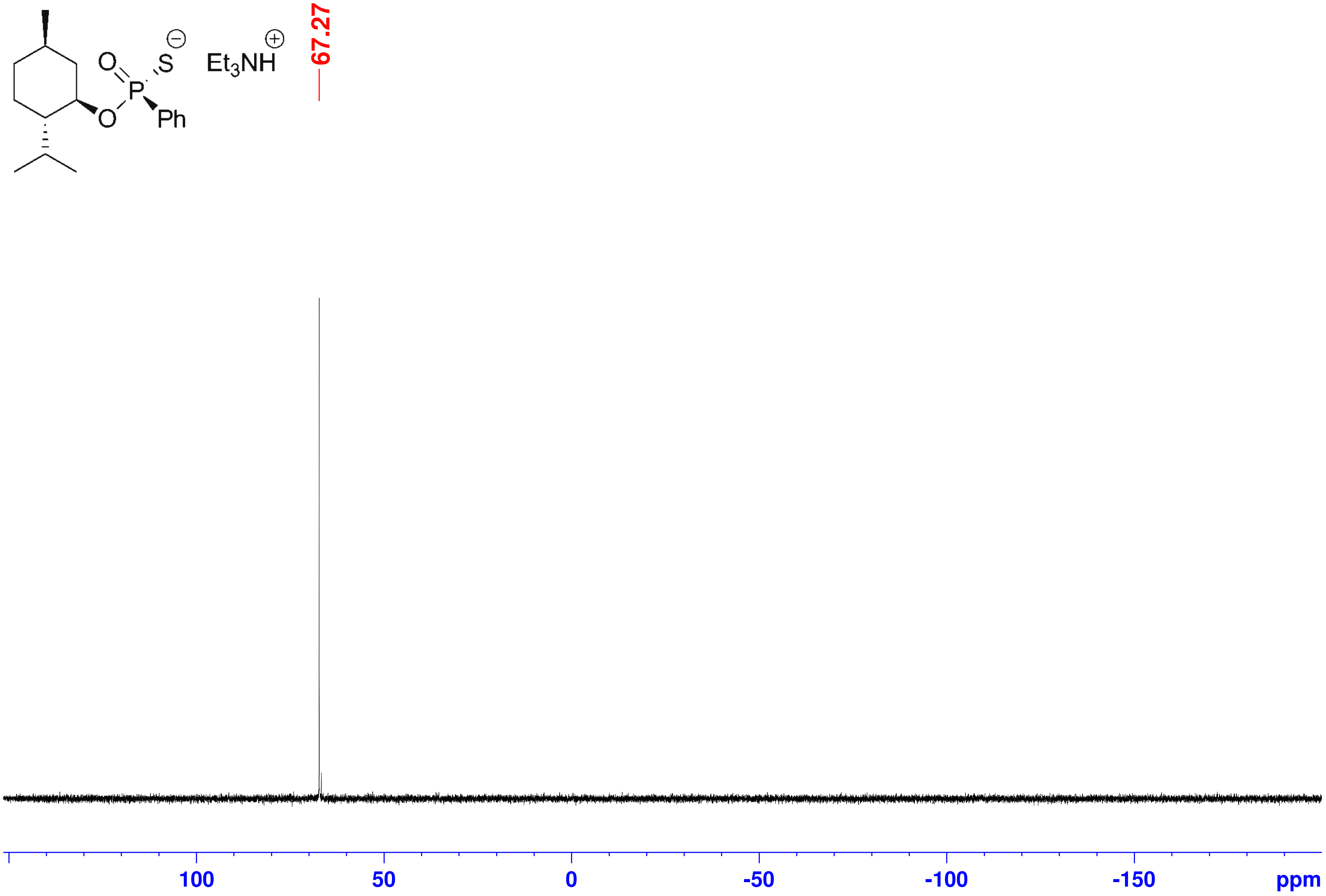

^{1}H NMR, 500 MHz, $CDCl_3$

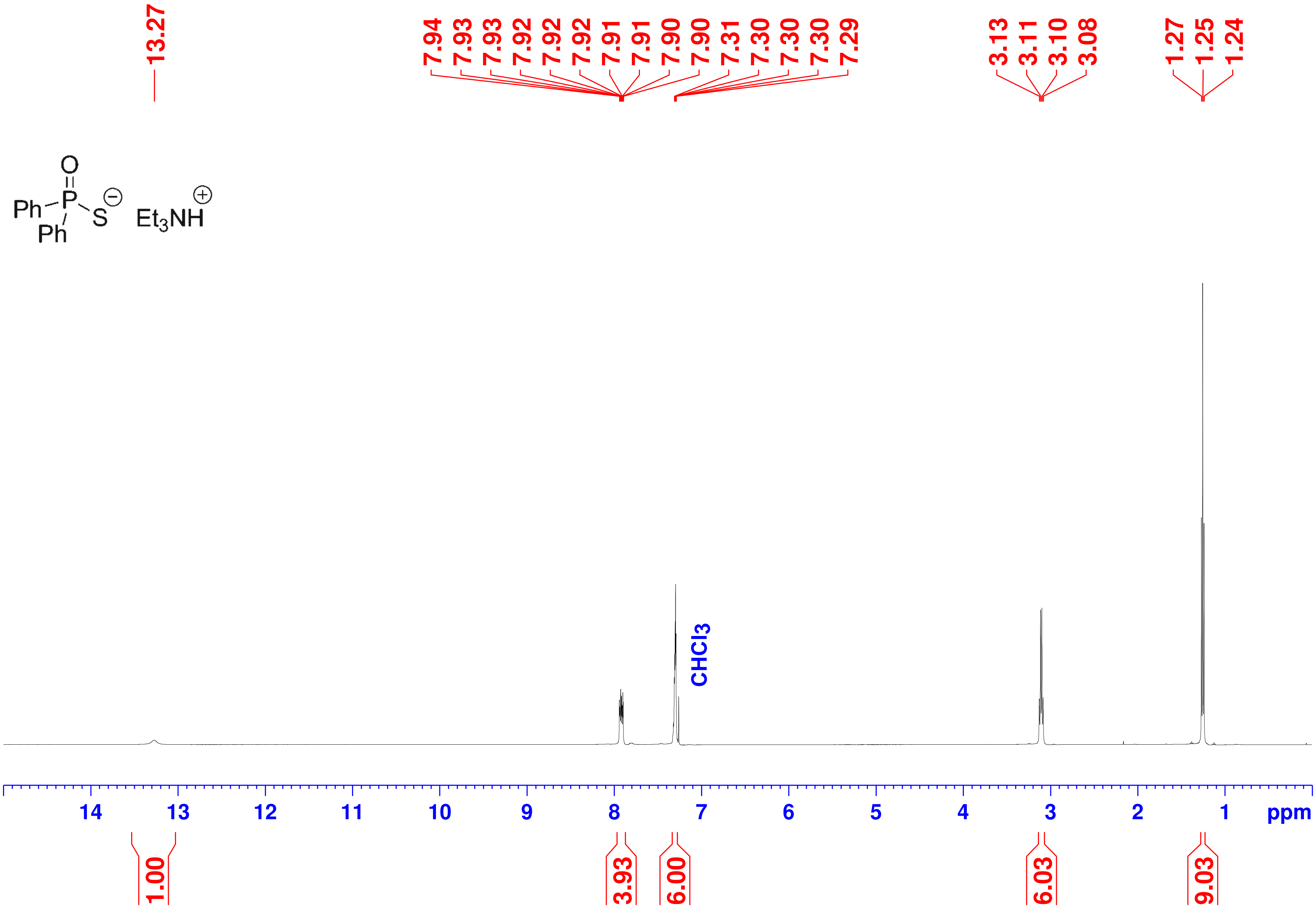

^{13}C NMR, 126 MHz, $CDCl_3$

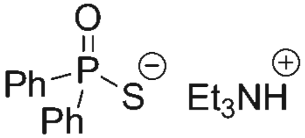

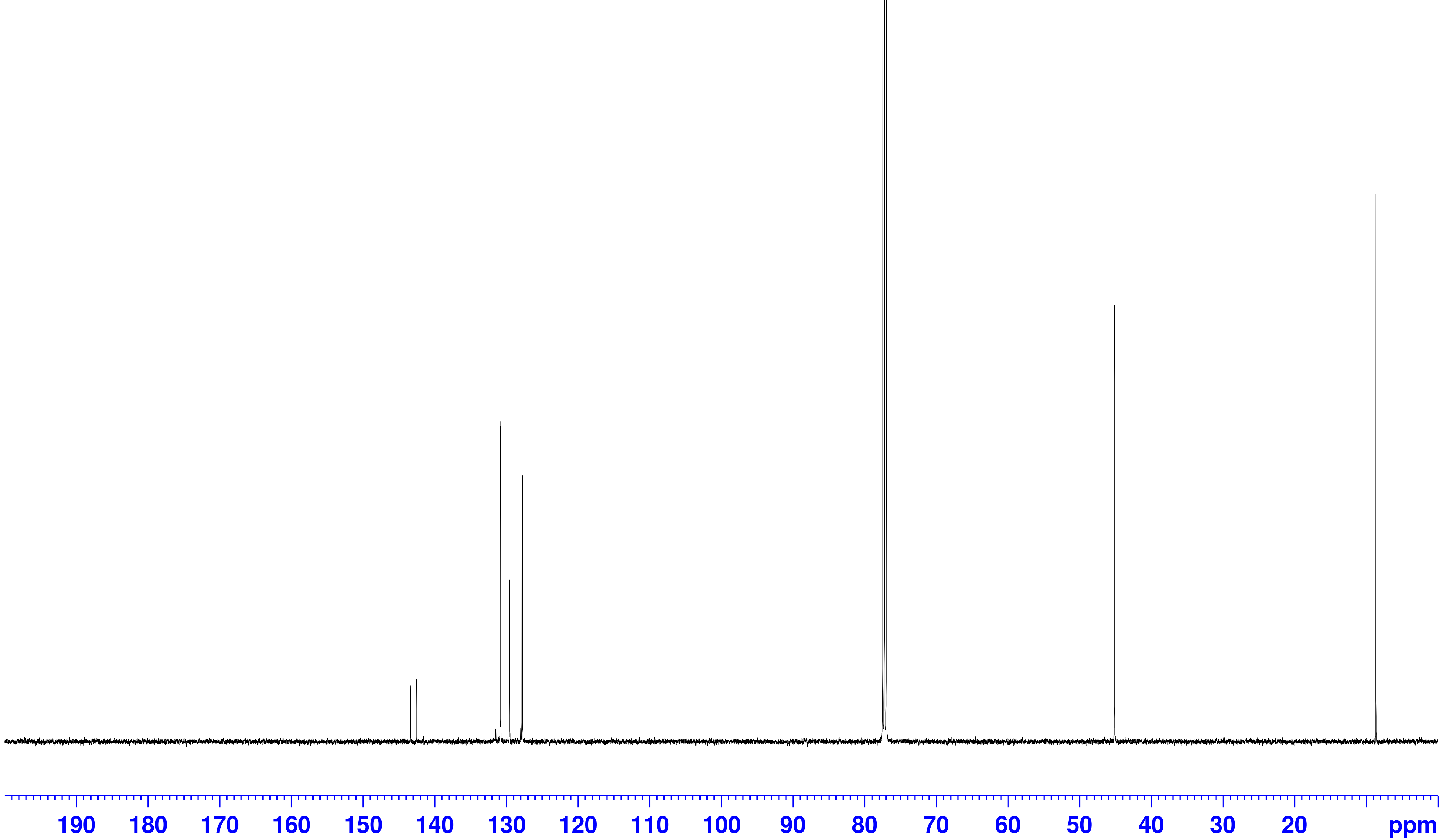

^{31}P NMR, 203 MHz, $CDCl_3$

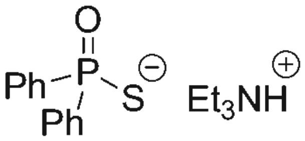

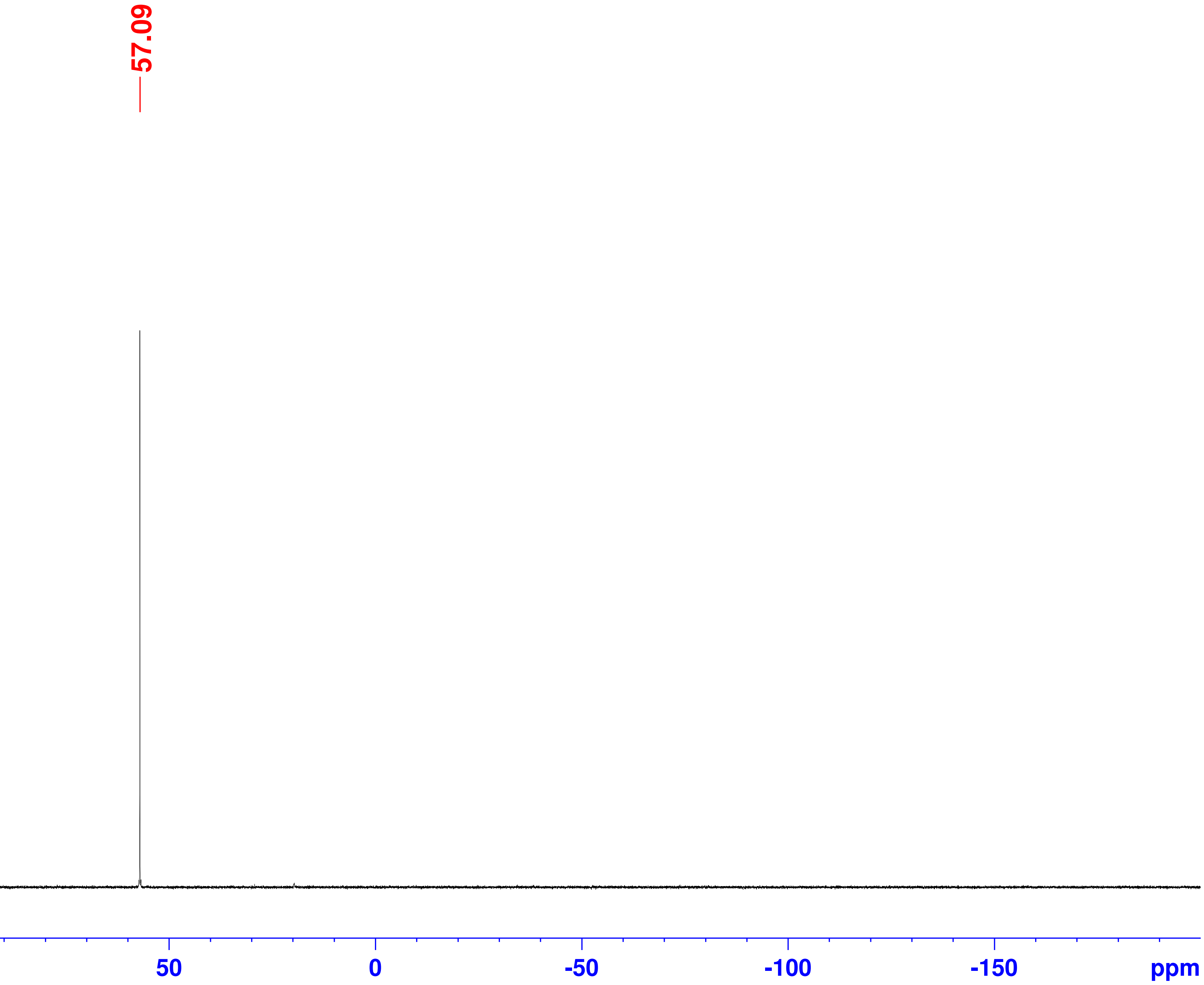

^{1}H NMR, 500 MHz, $CDCl_3$

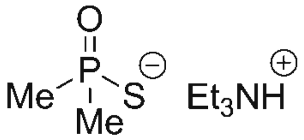

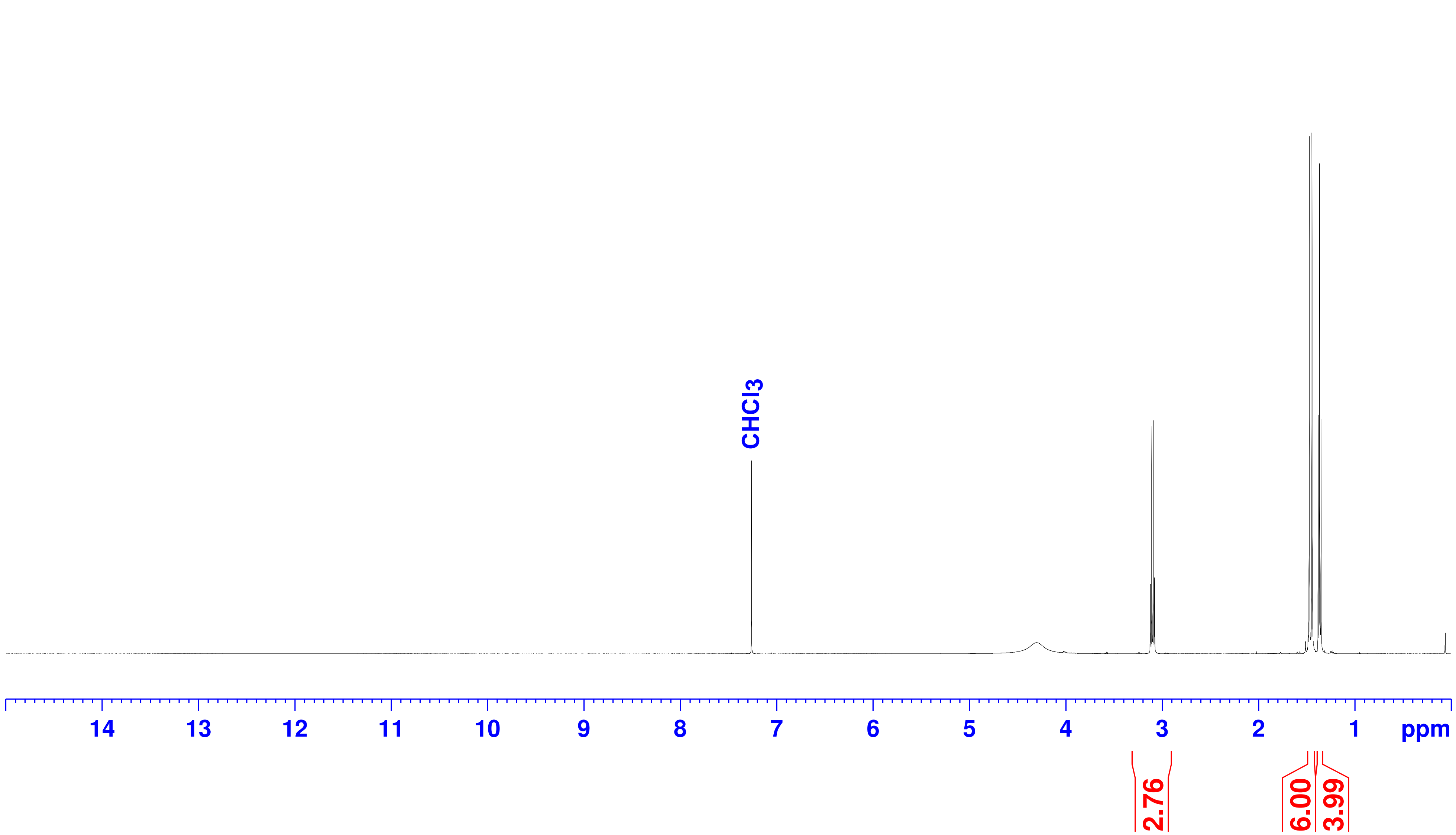

^{13}C NMR, 126 MHz, $CDCl_3$

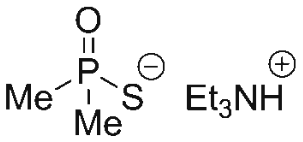

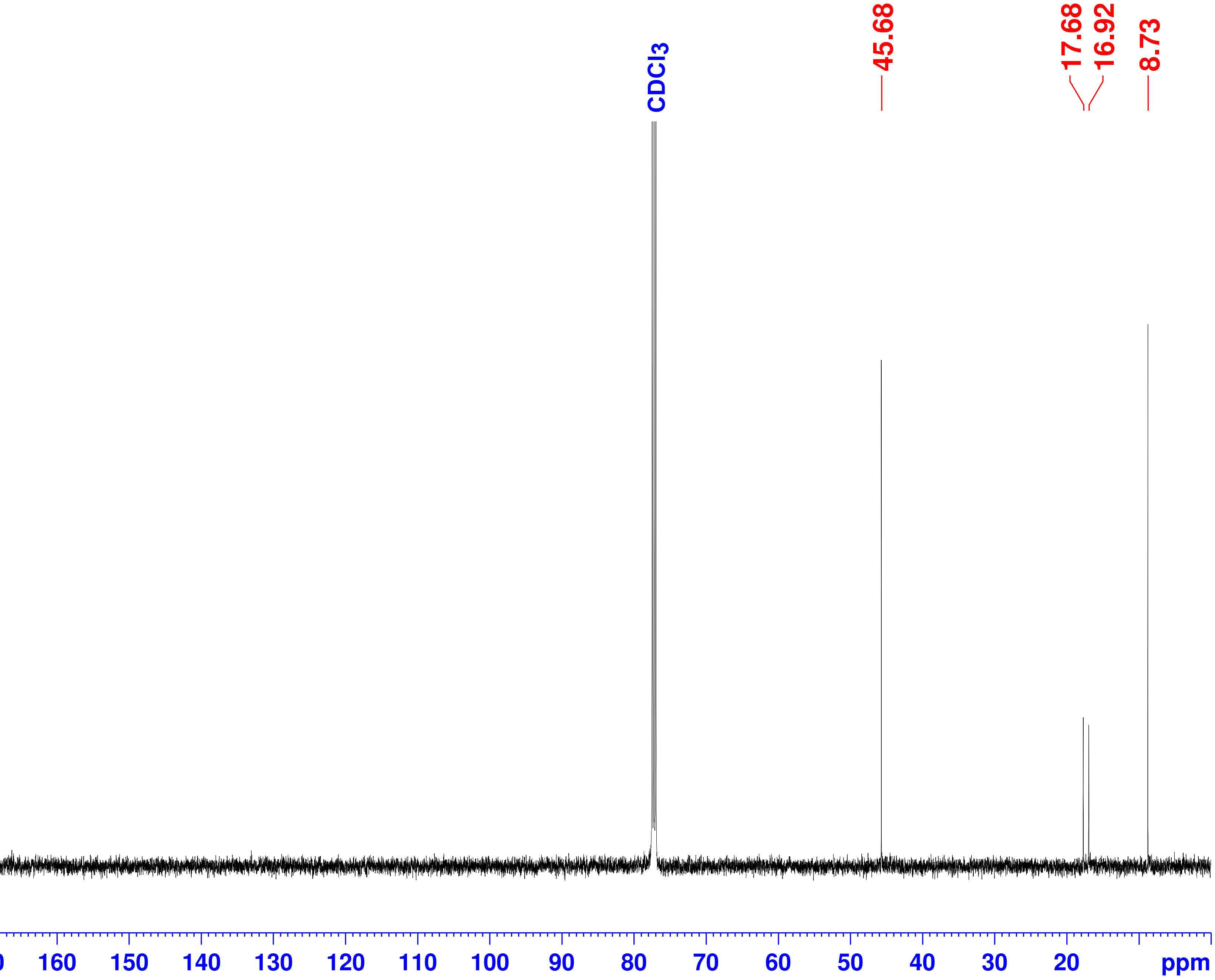

^{31}P NMR, 203 MHz, $CDCl_3$

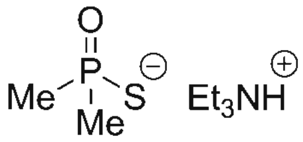

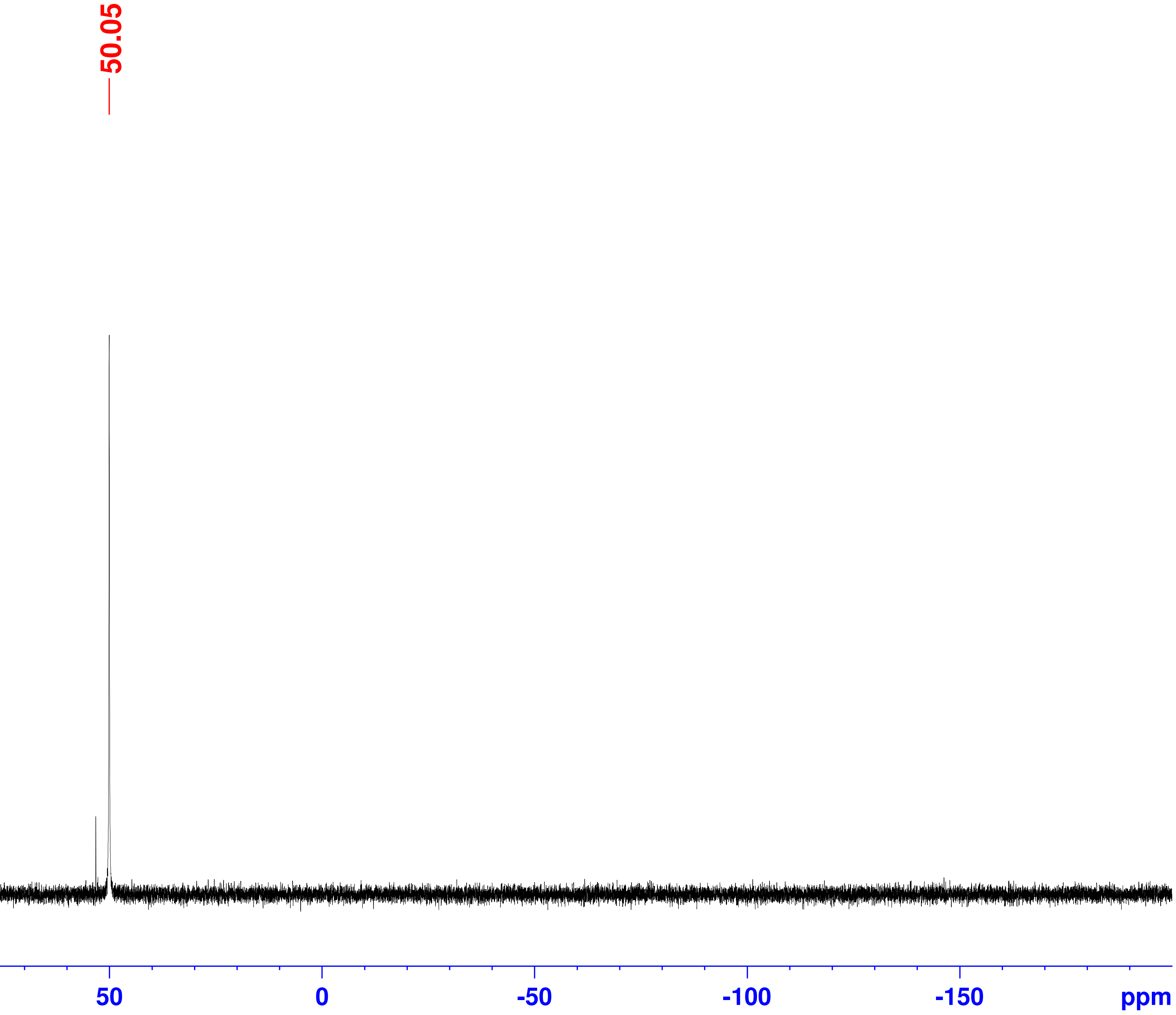

^{1}H NMR, 500 MHz, $CDCl_3$

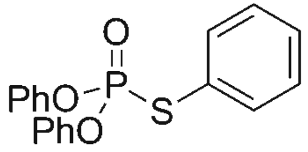

3a

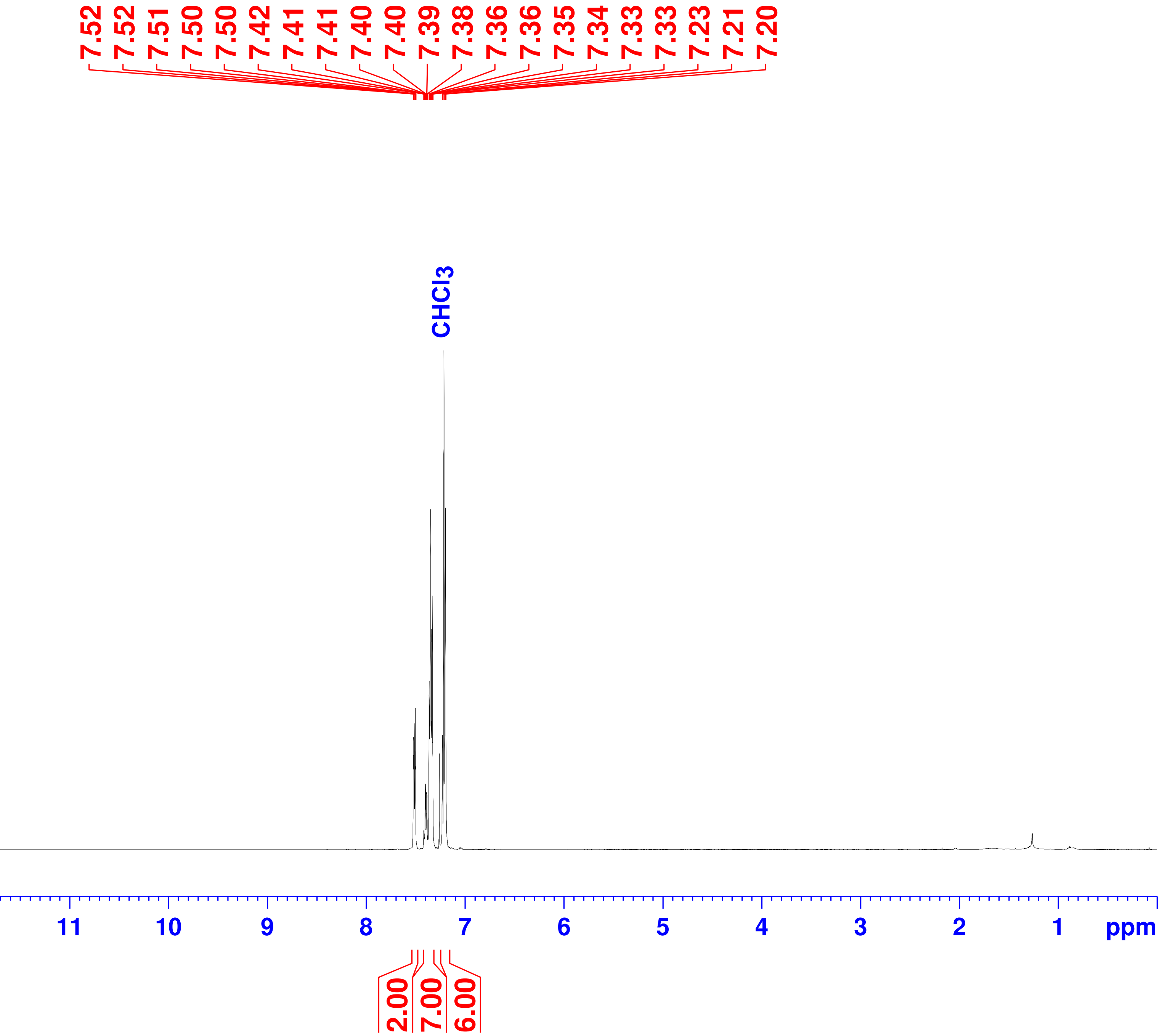

^{13}C NMR, 126 MHz, $CDCl_3$

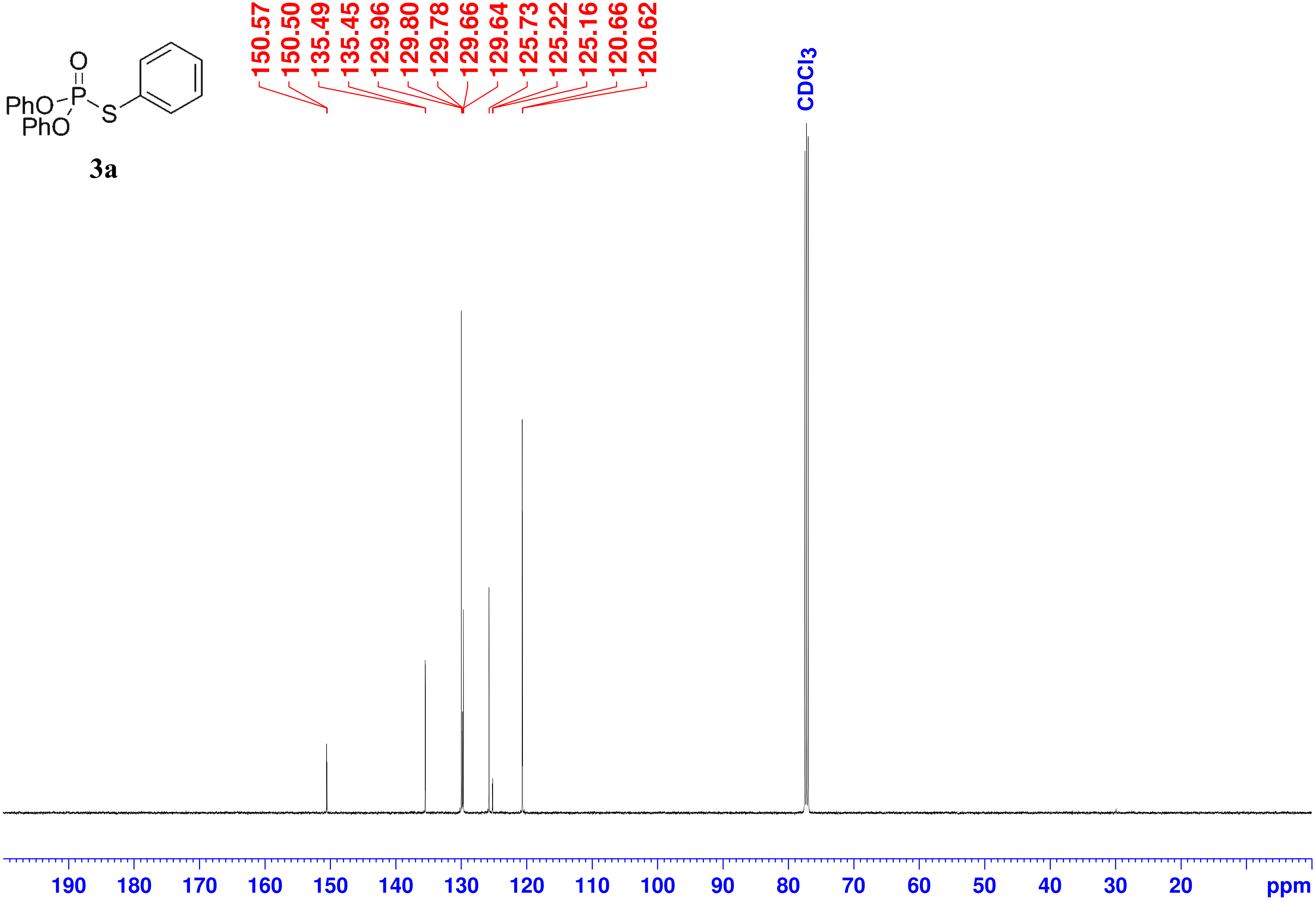

^{31}P NMR, 203 MHz, $CDCl_3$

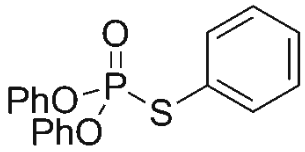

3a

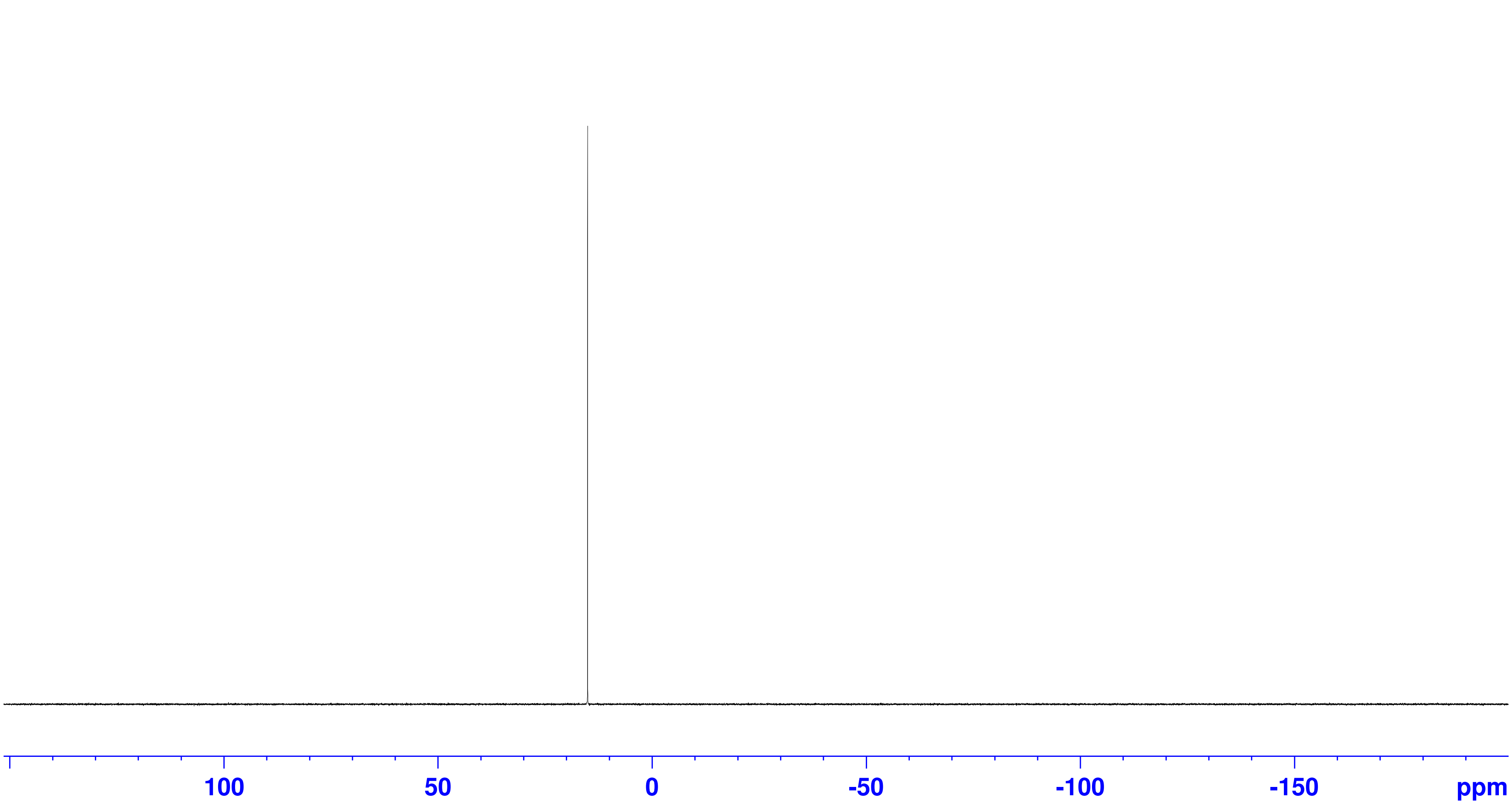

^{1}H NMR, 500 MHz, $CDCl_3$

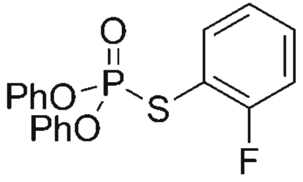

3b

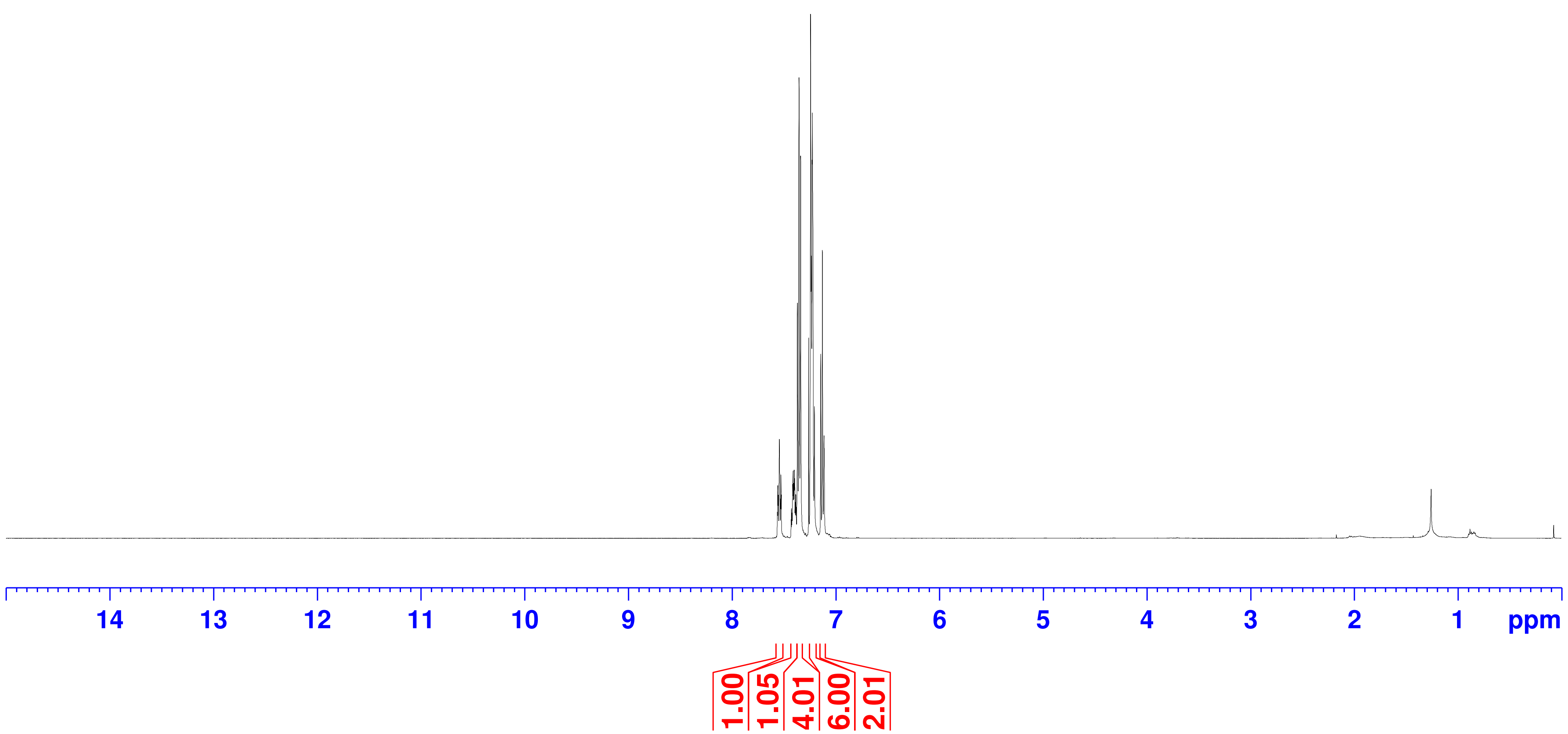

^{13}C NMR, 126 MHz, $CDCl_3$

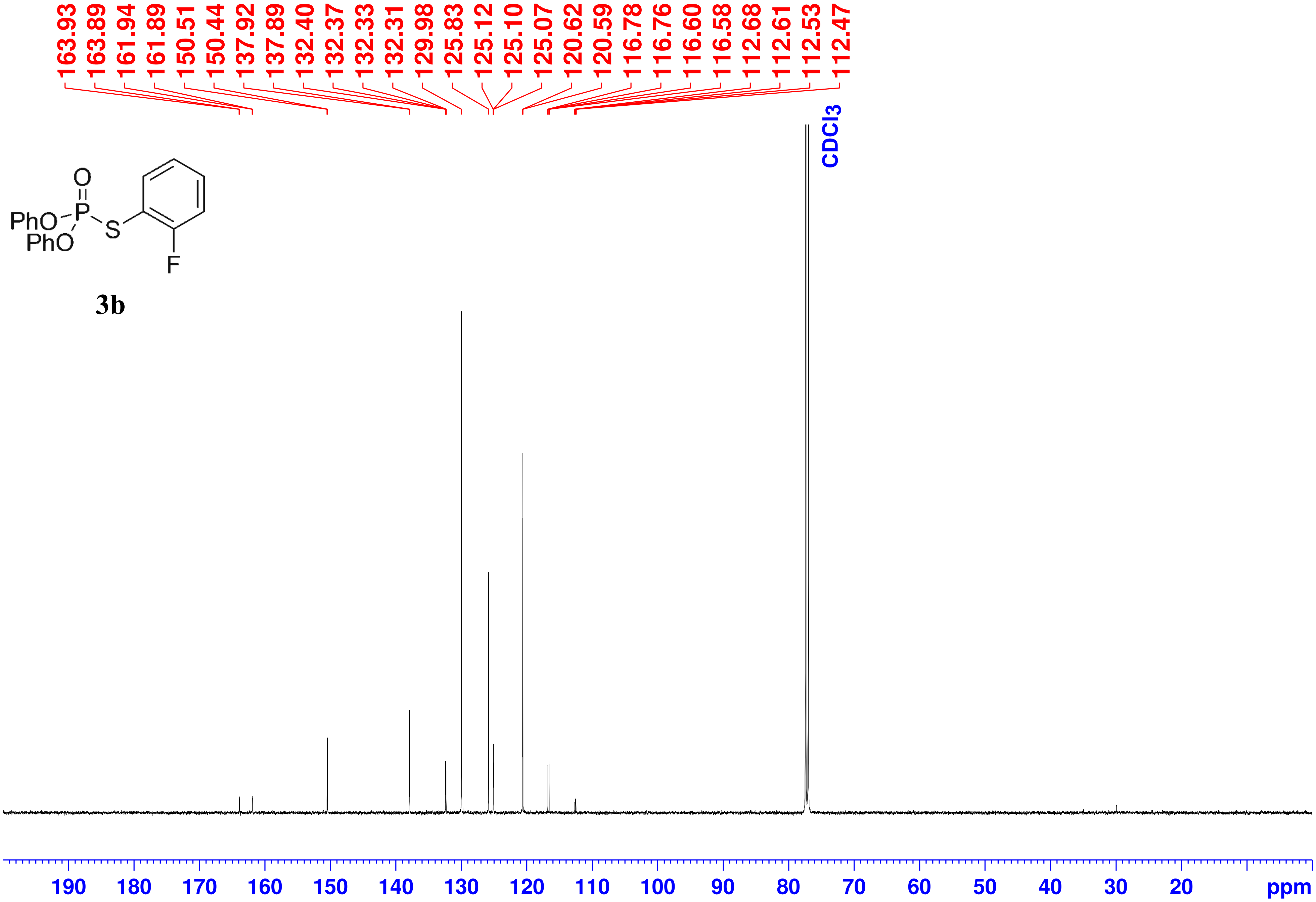

3b

^{31}P NMR, 203 MHz, $CDCl_3$

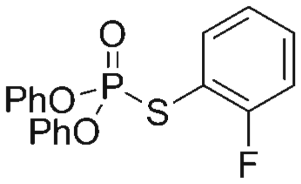

3b

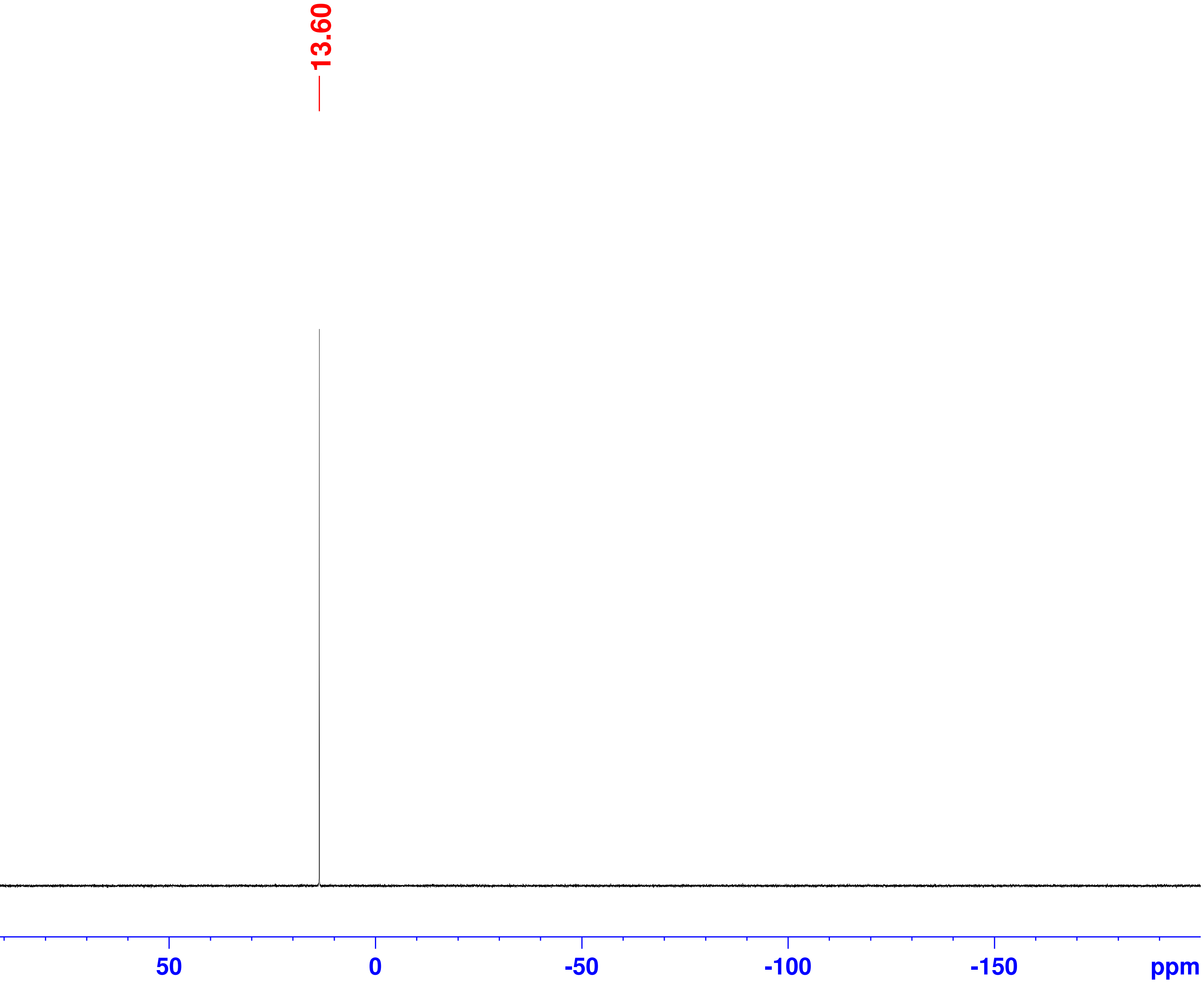

^{19}F NMR, 376 MHz, CDCl$_3$

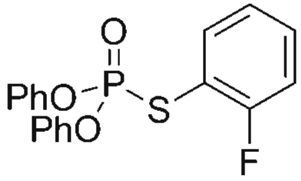

3b

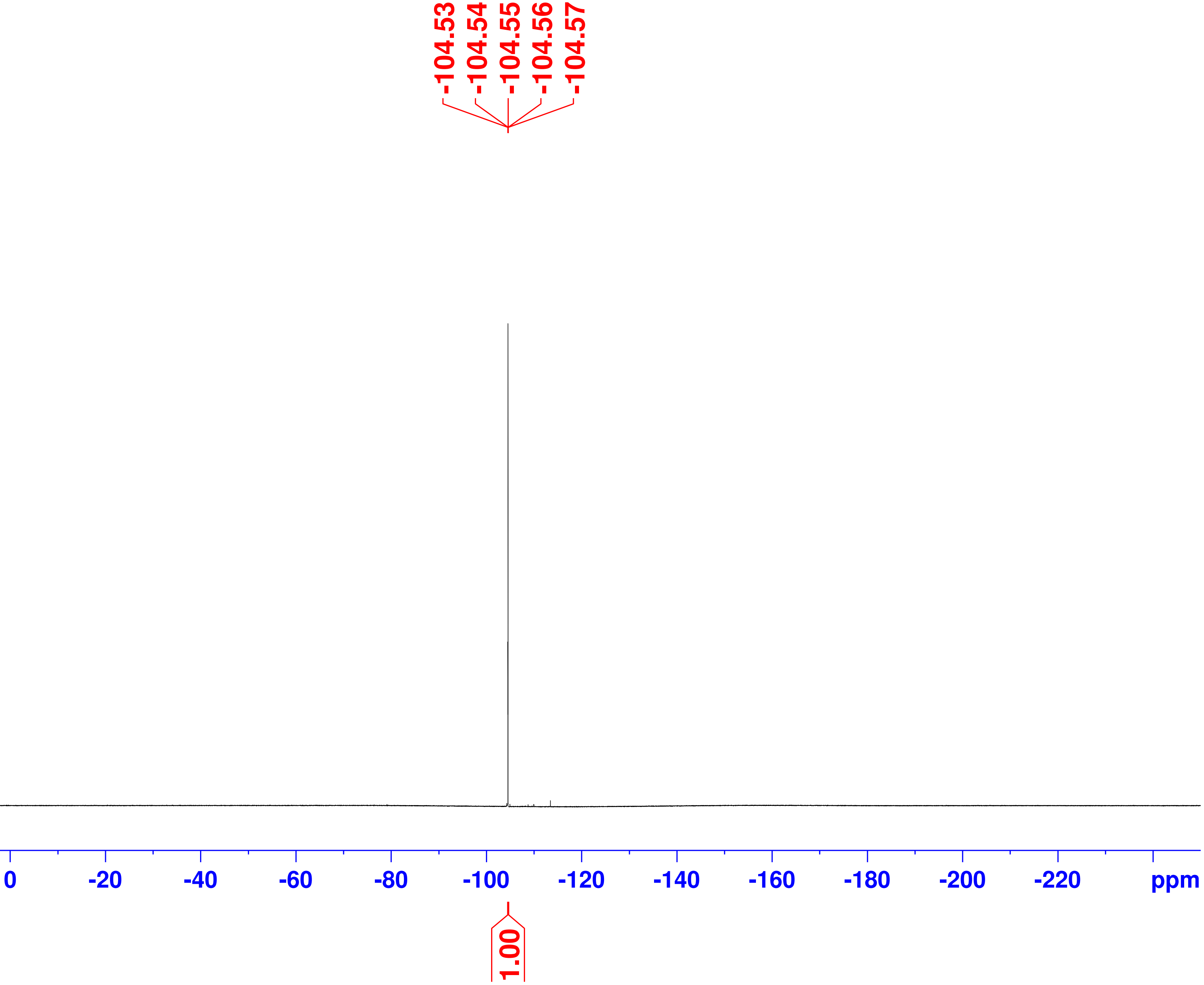

^1H NMR, 500 MHz, $CDCl_3$

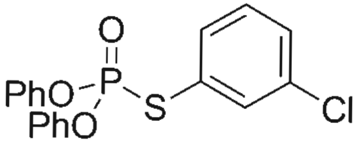

3c

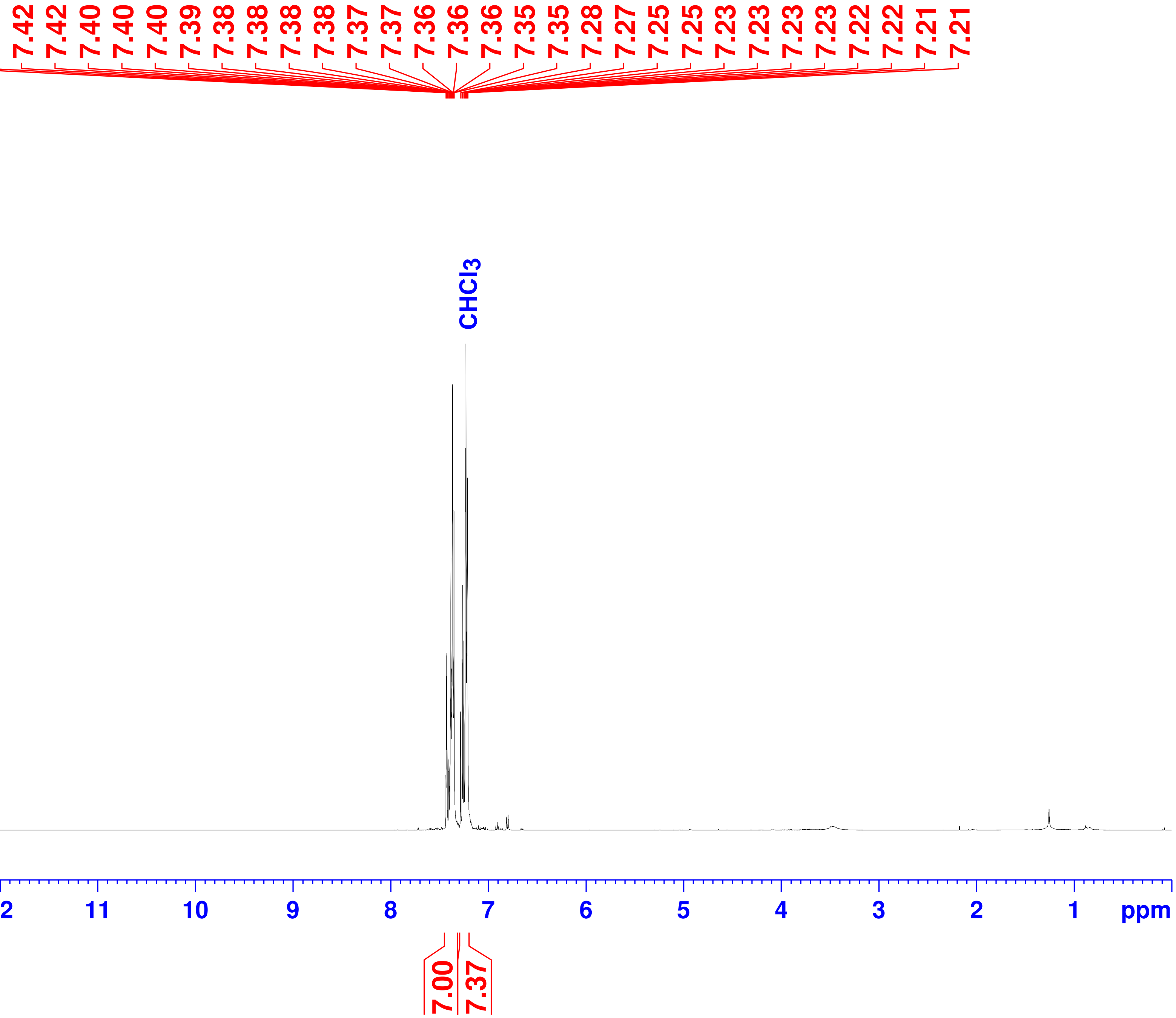

^{13}C NMR, 126 MHz, $CDCl_3$

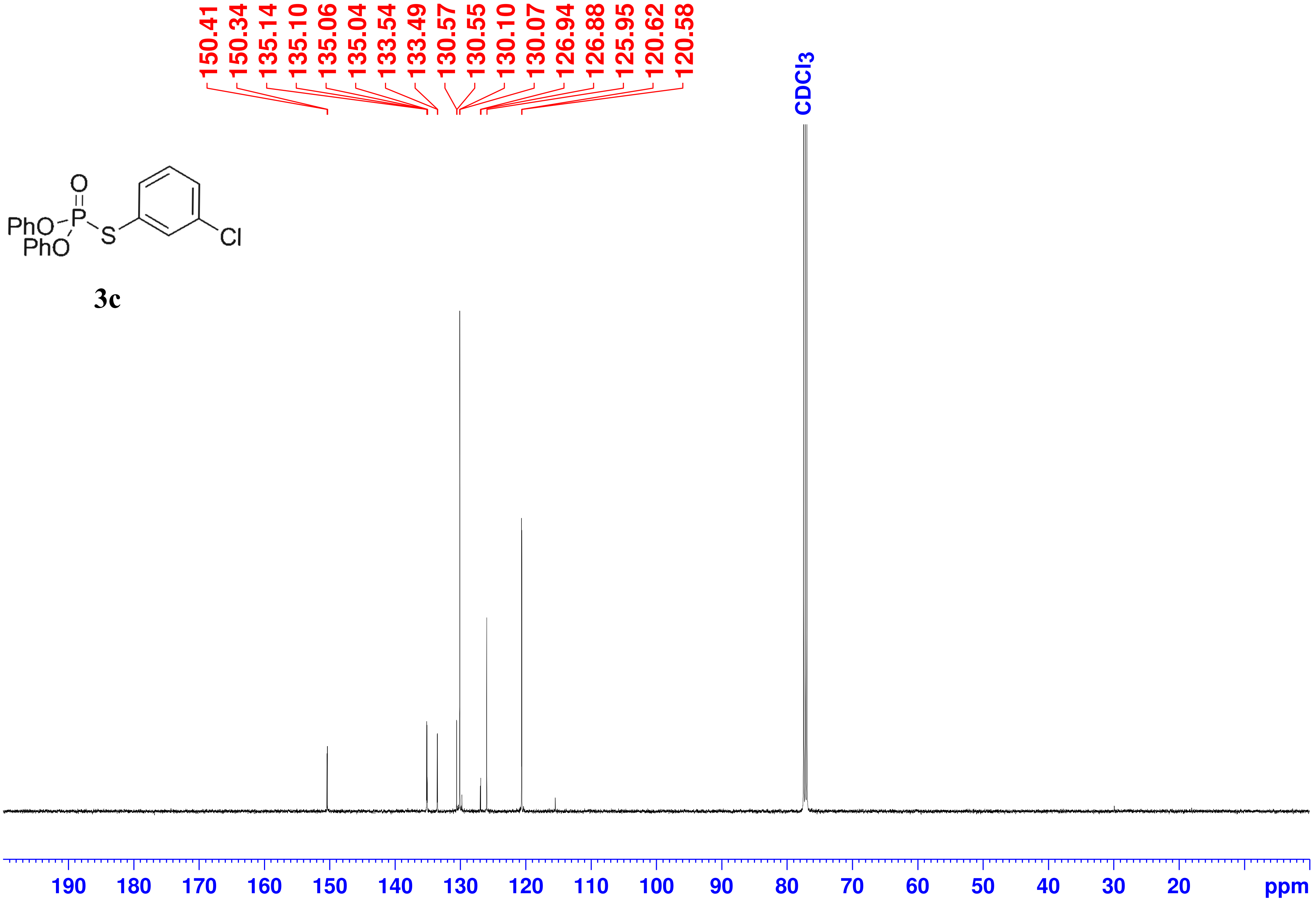

^{31}P NMR, 203 MHz, $CDCl_3$

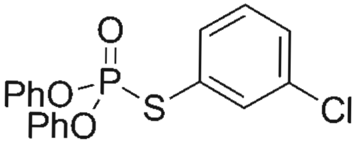

3c

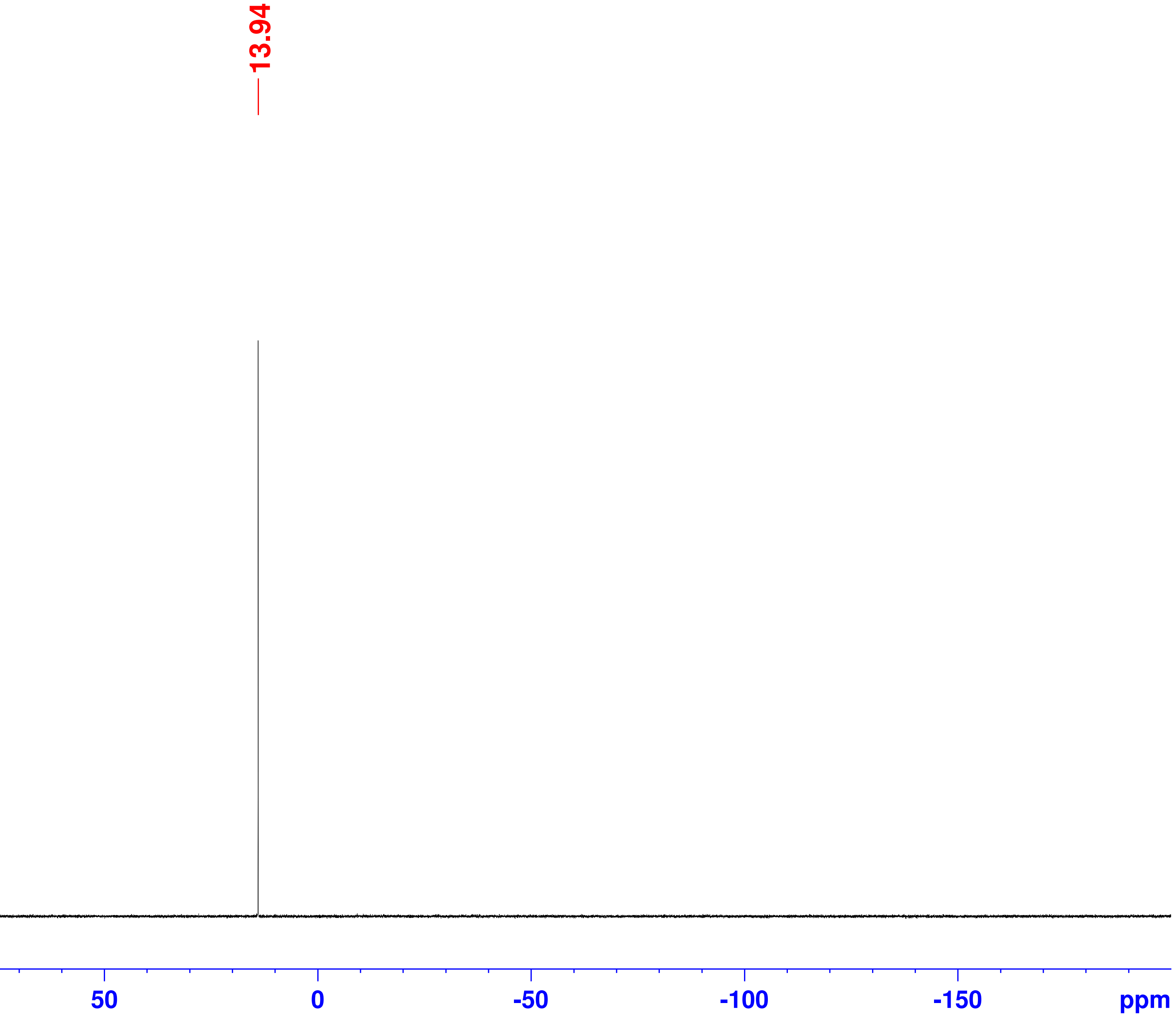

^{1}H NMR, 500 MHz, $CDCl_3$

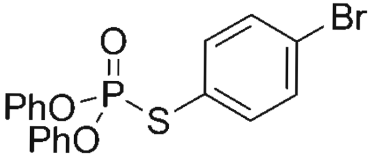

3d

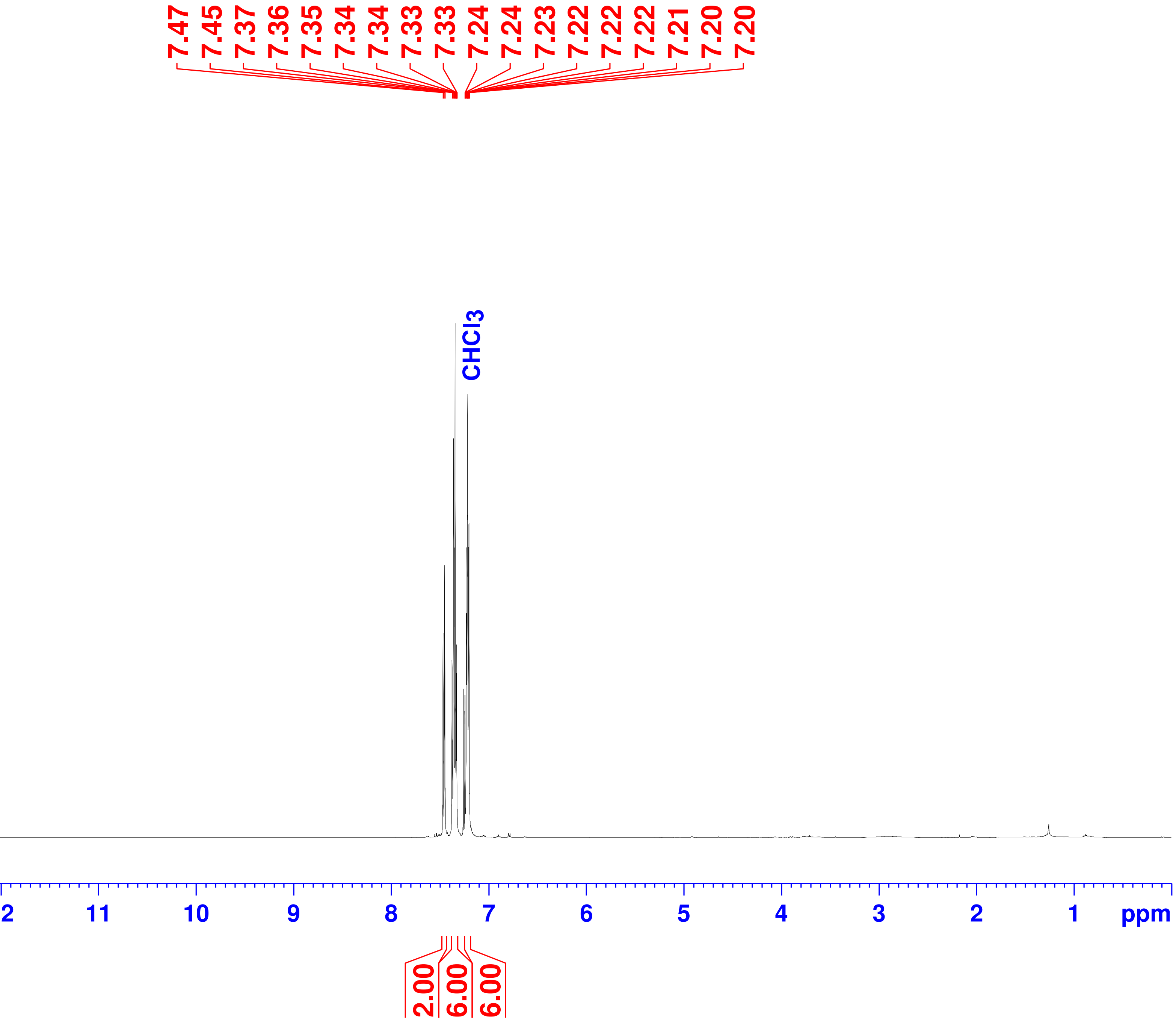

^{13}C NMR, 126 MHz, $CDCl_3$

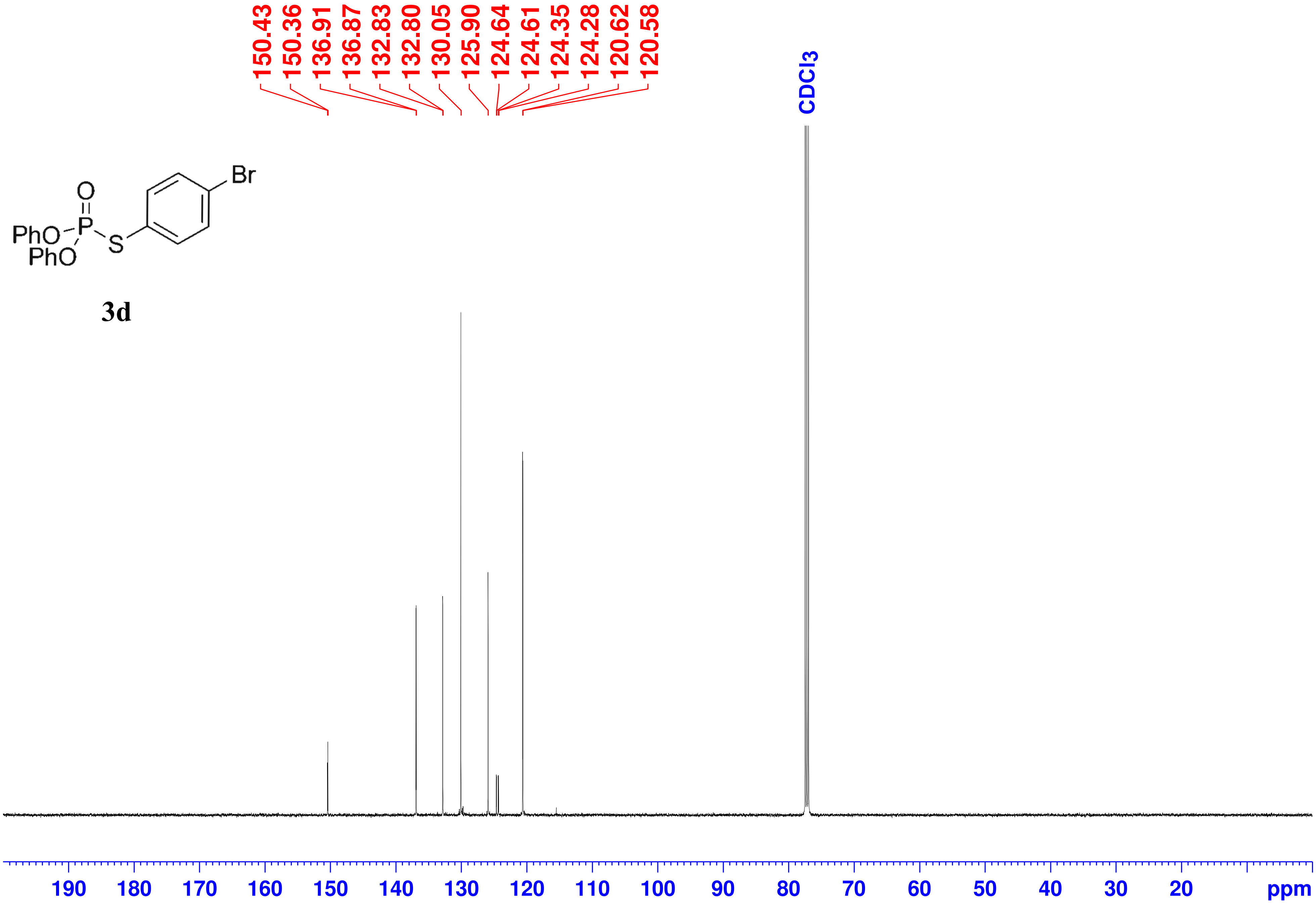

^{31}P NMR, 203 MHz, $CDCl_3$

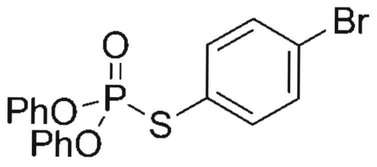

3d

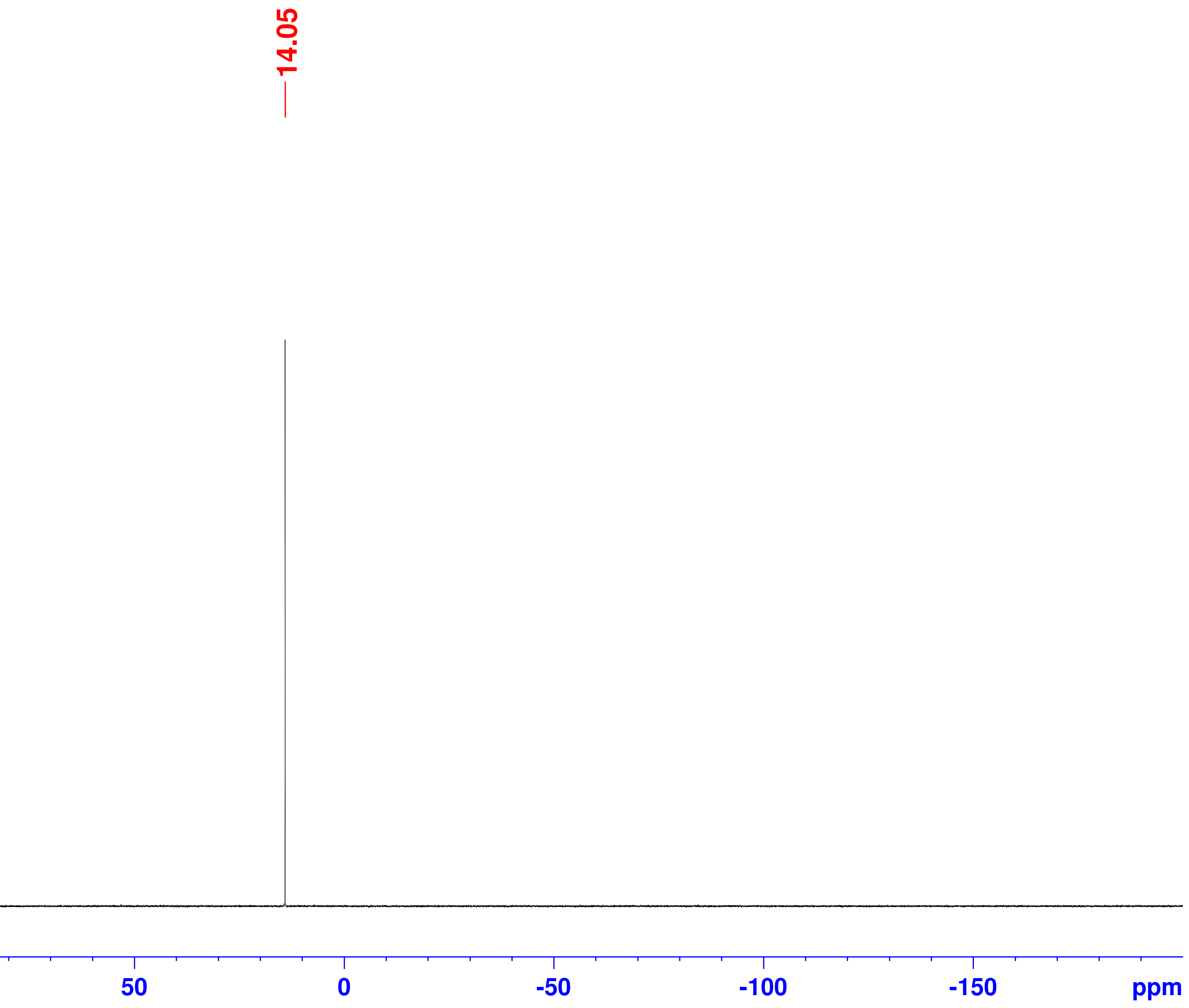

^{1}H NMR, 500 MHz, $CDCl_3$

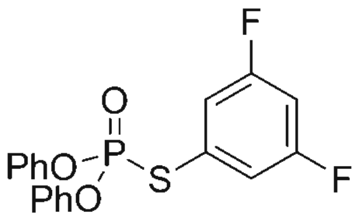

3e

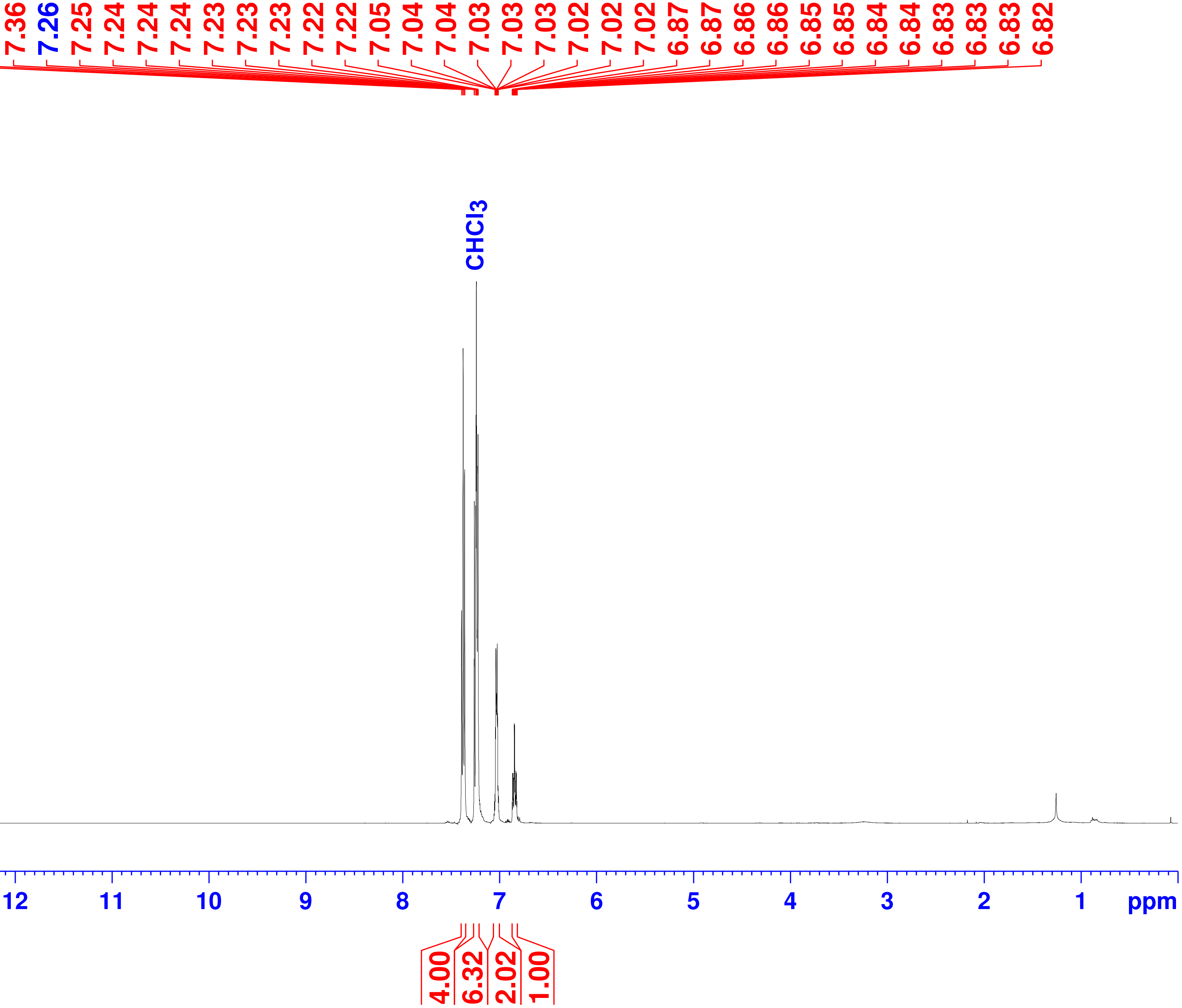

^{13}C NMR, 126 MHz, $CDCl_3$

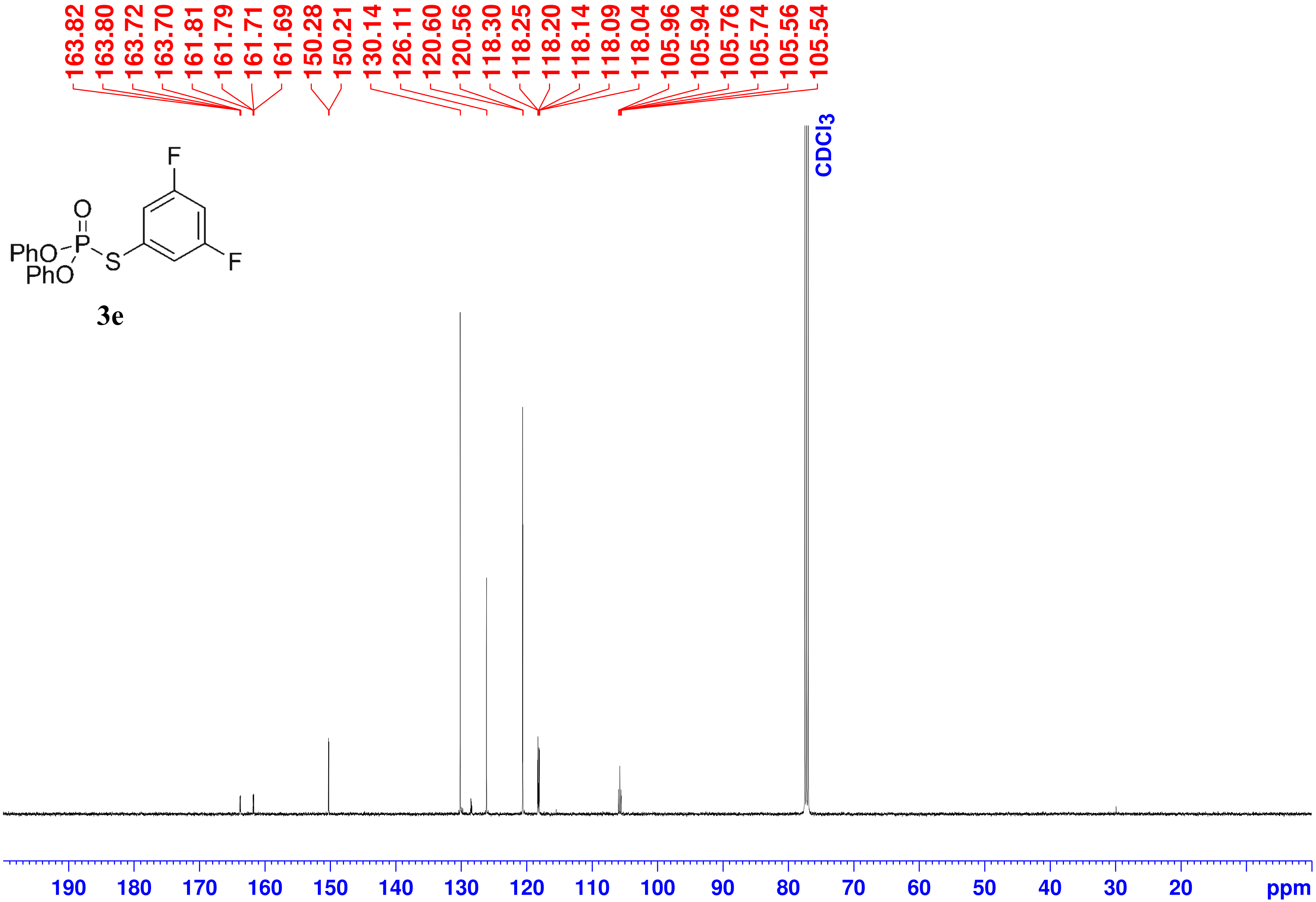

3e

^{31}P NMR, 203 MHz, $CDCl_3$

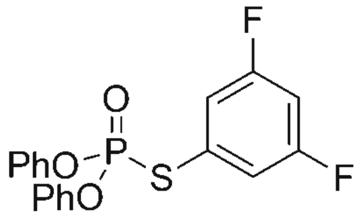

3e

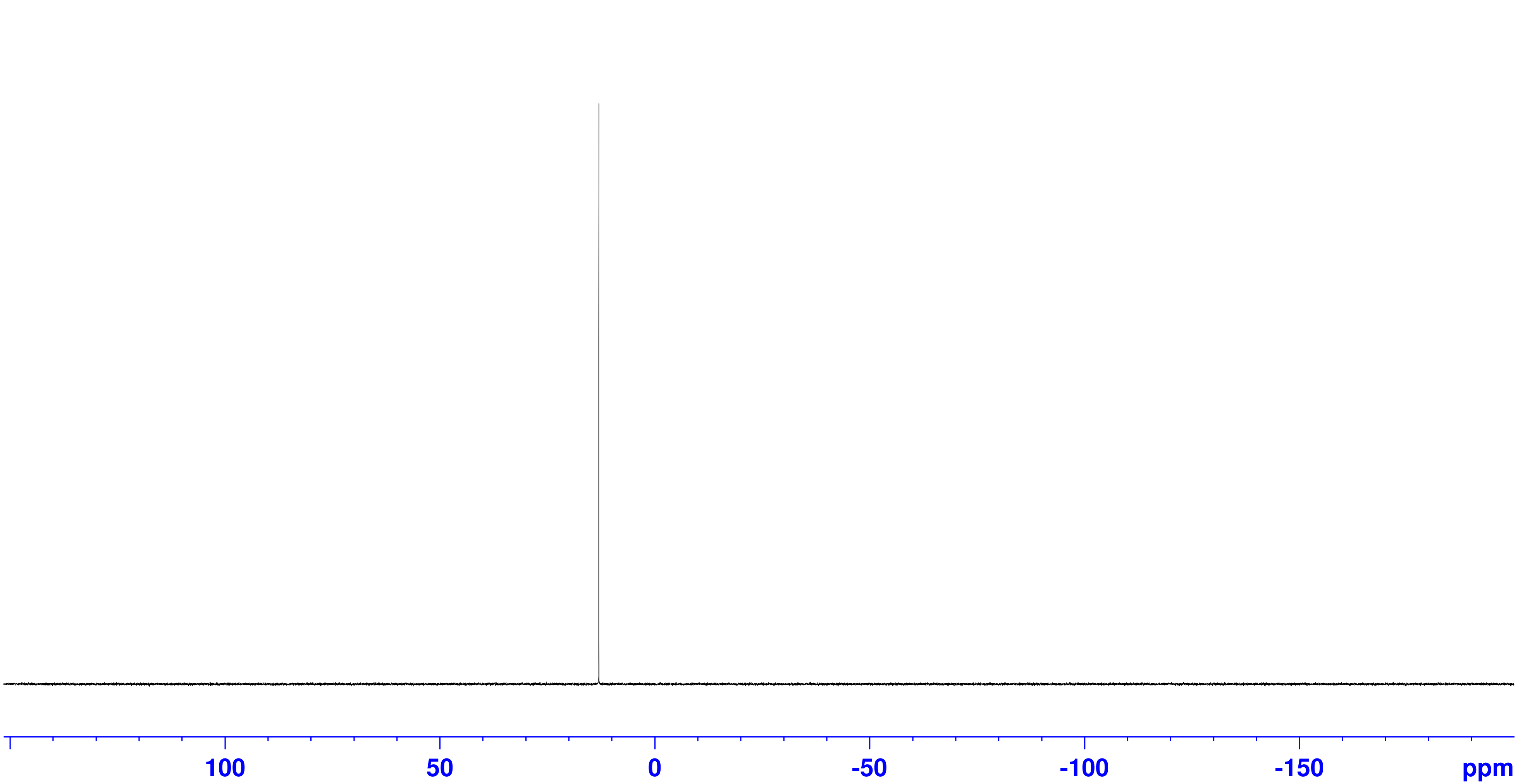

^{19}F NMR, 376 MHz, $CDCl_3$

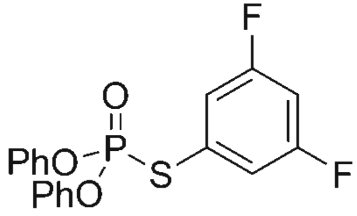

3e

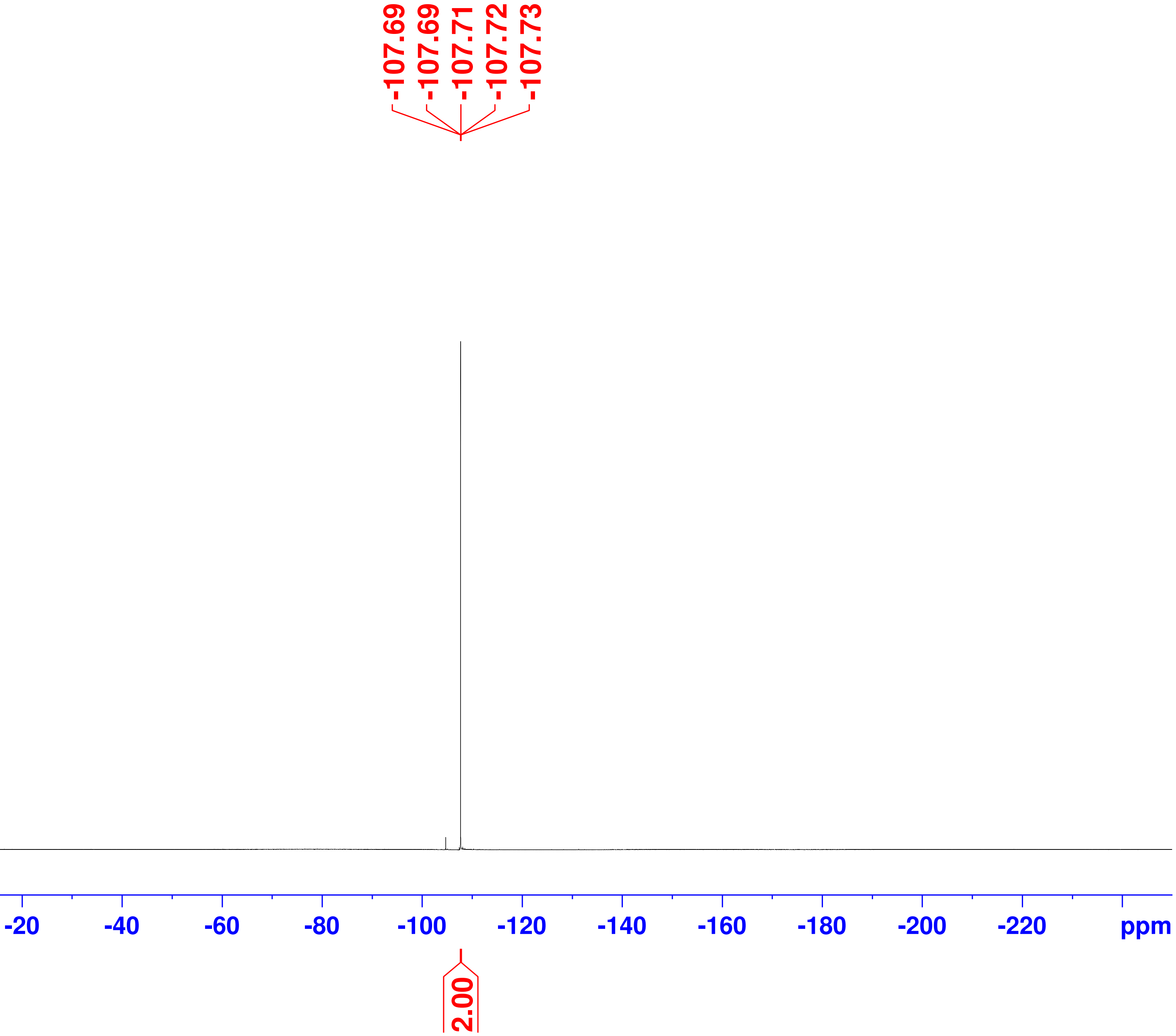

^{1}H NMR, 500 MHz, $CDCl_3$

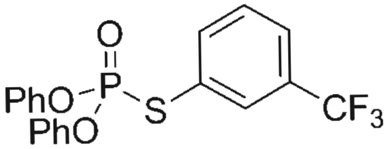

3f

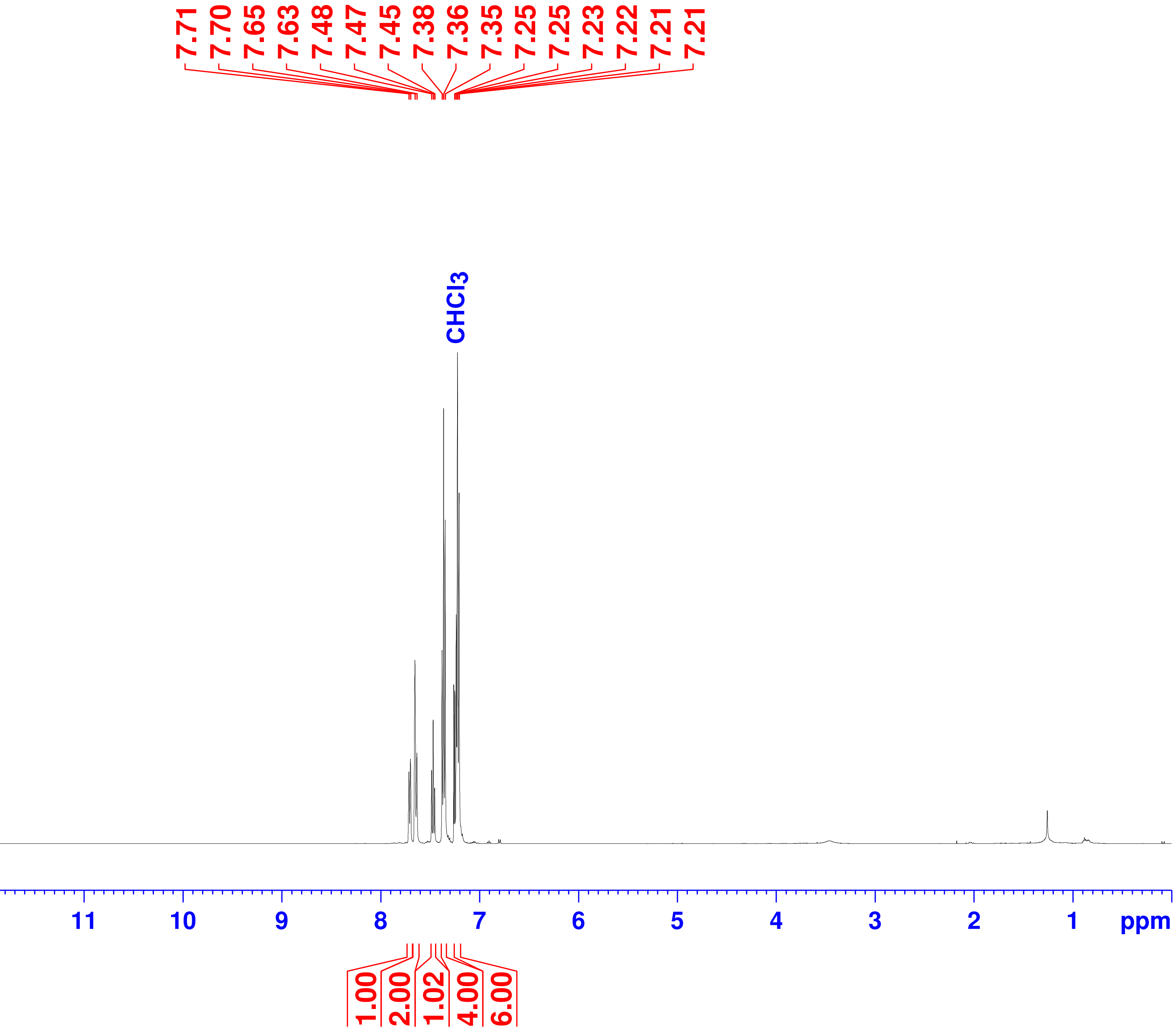

^{13}C NMR, 126 MHz, $CDCl_3$

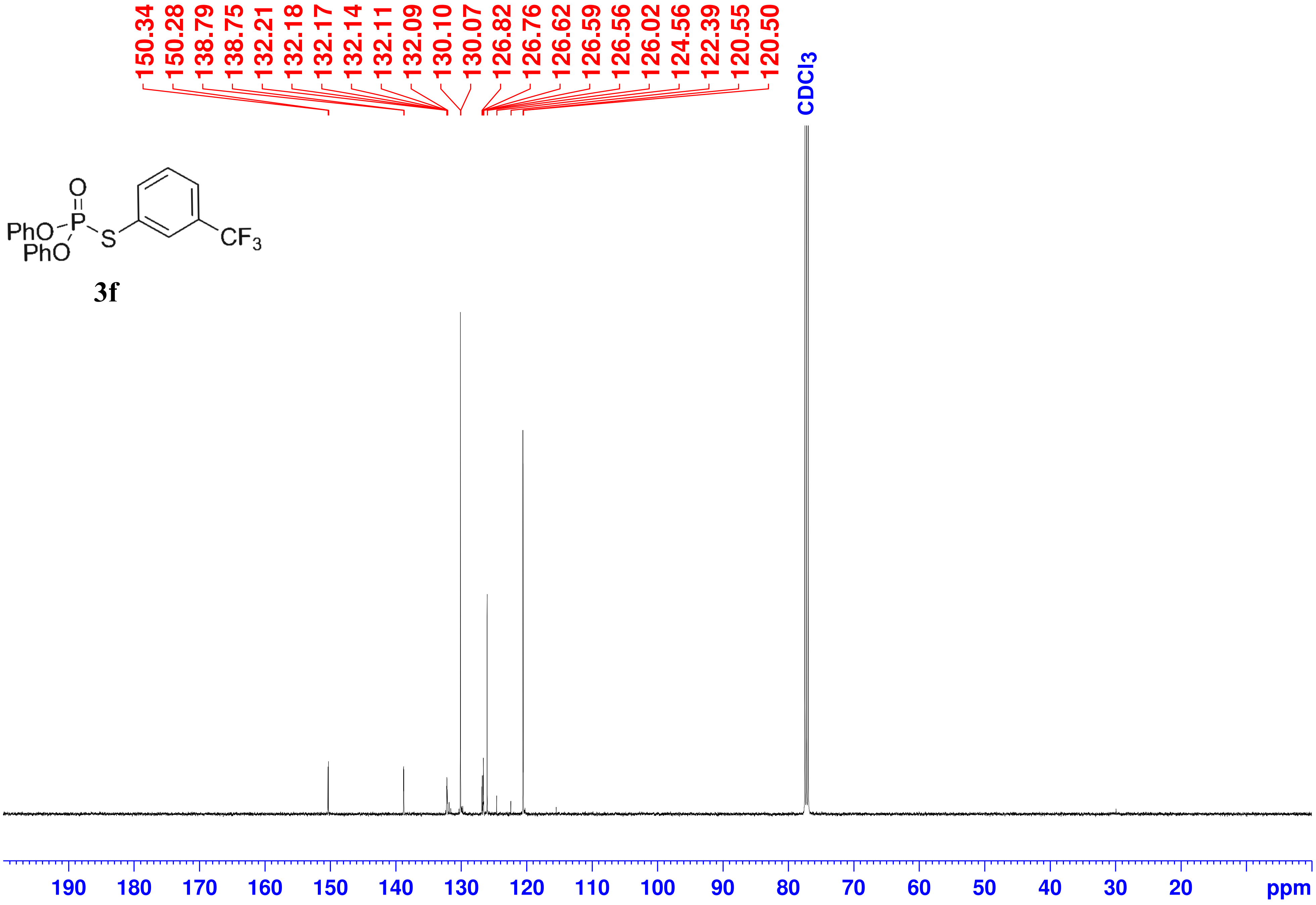

3f

^{31}P NMR, 203 MHz, $CDCl_3$

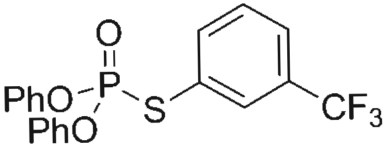

3f

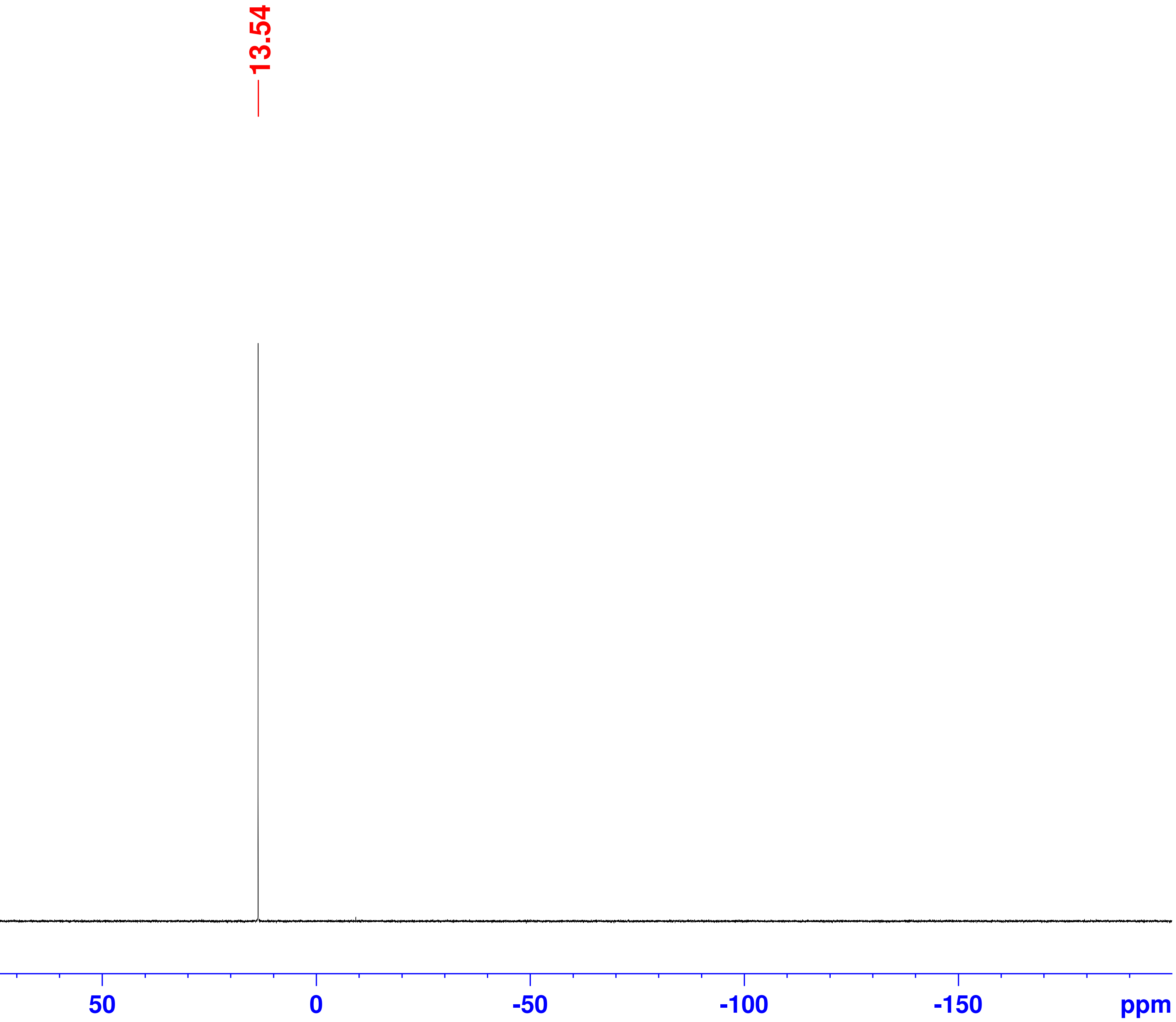

^{19}F NMR, 376 MHz, $CDCl_3$

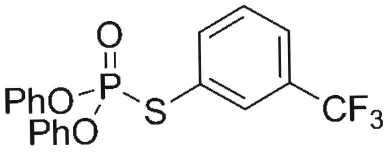

3f

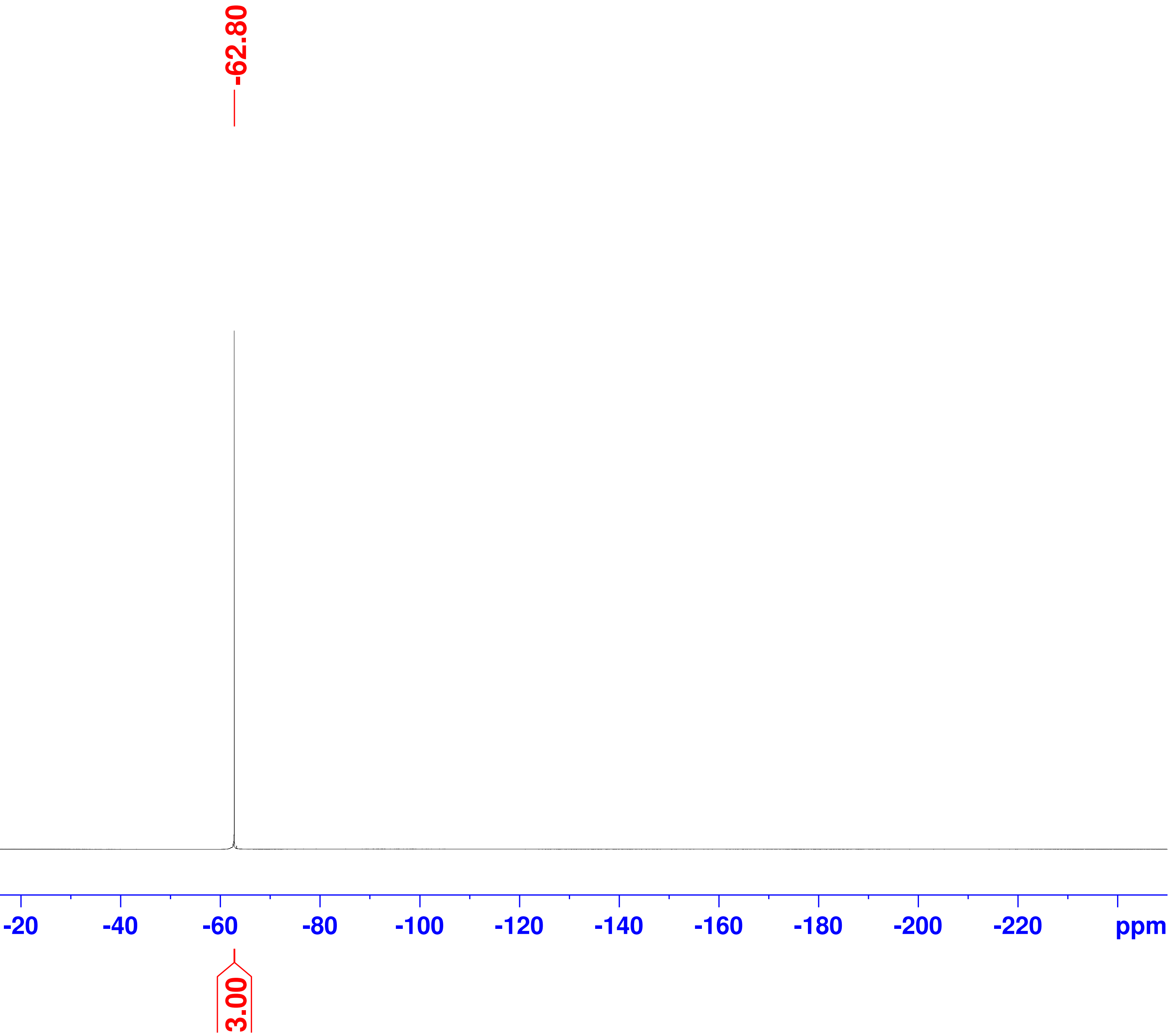

^{1}H NMR, 500 MHz, $CDCl_3$

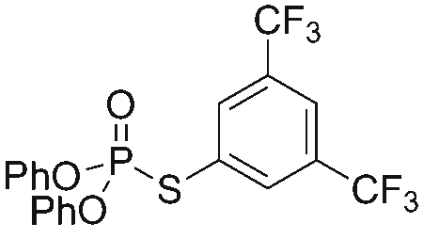

3g

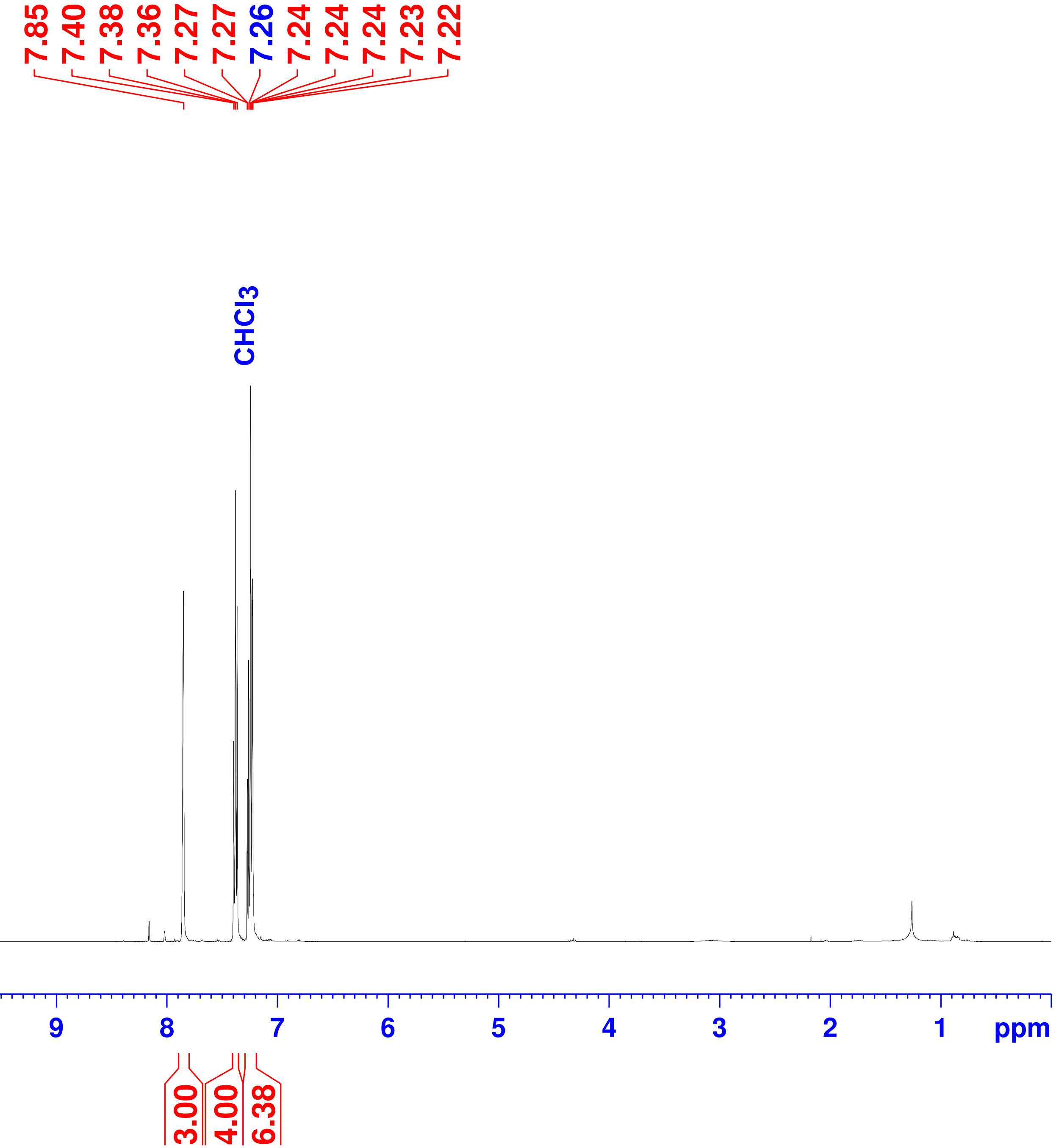

^{13}C NMR, 126 MHz, $CDCl_3$

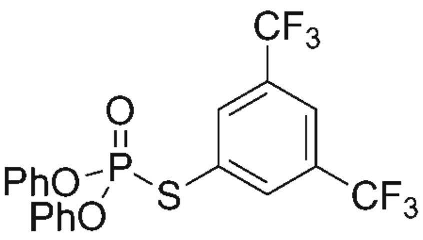

3g

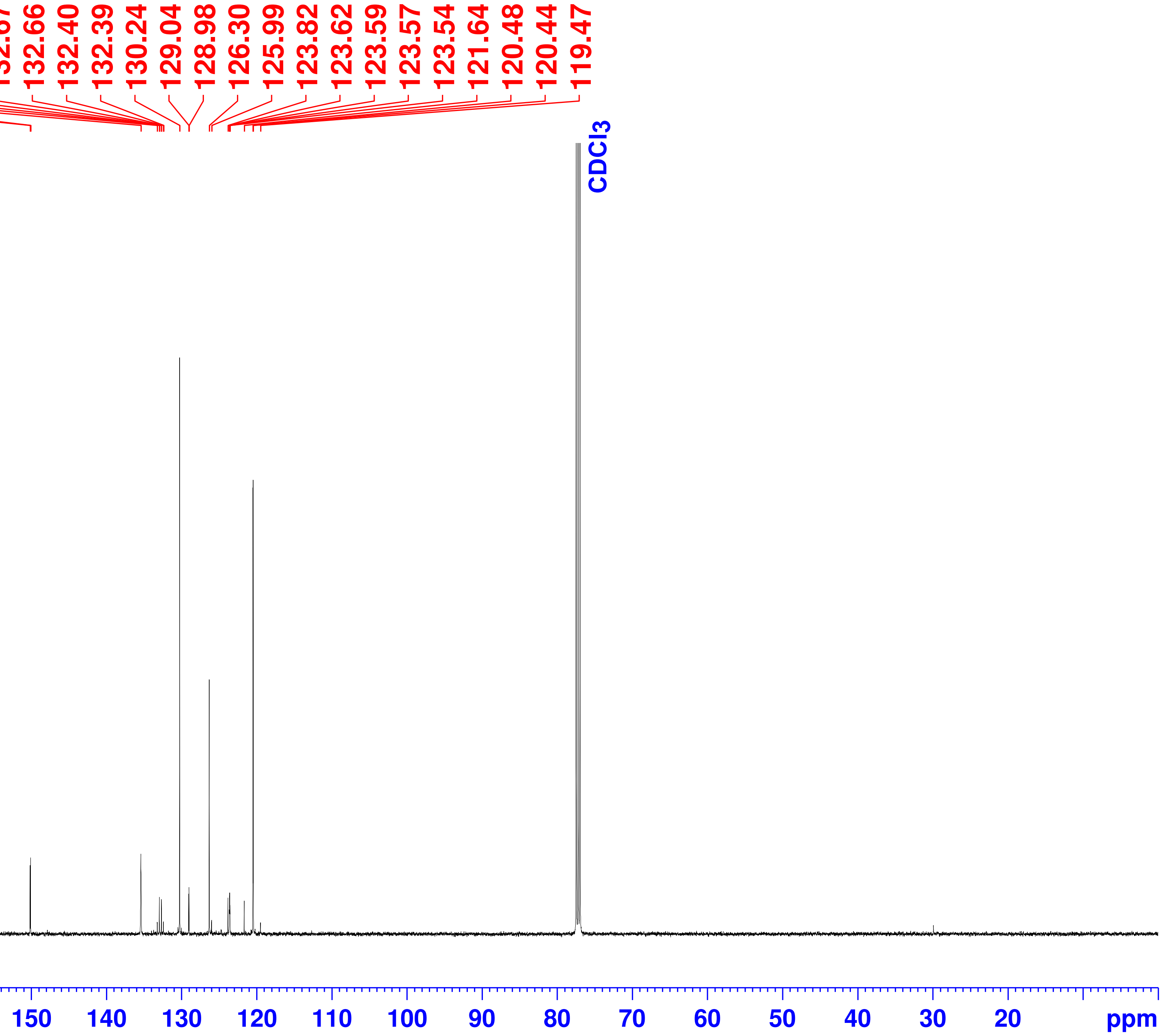

^{31}P NMR, 203 MHz, $CDCl_3$

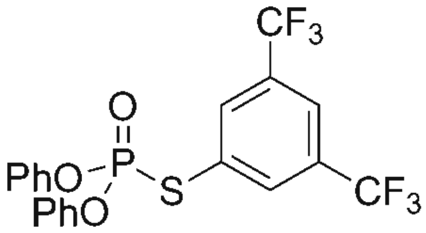

3g

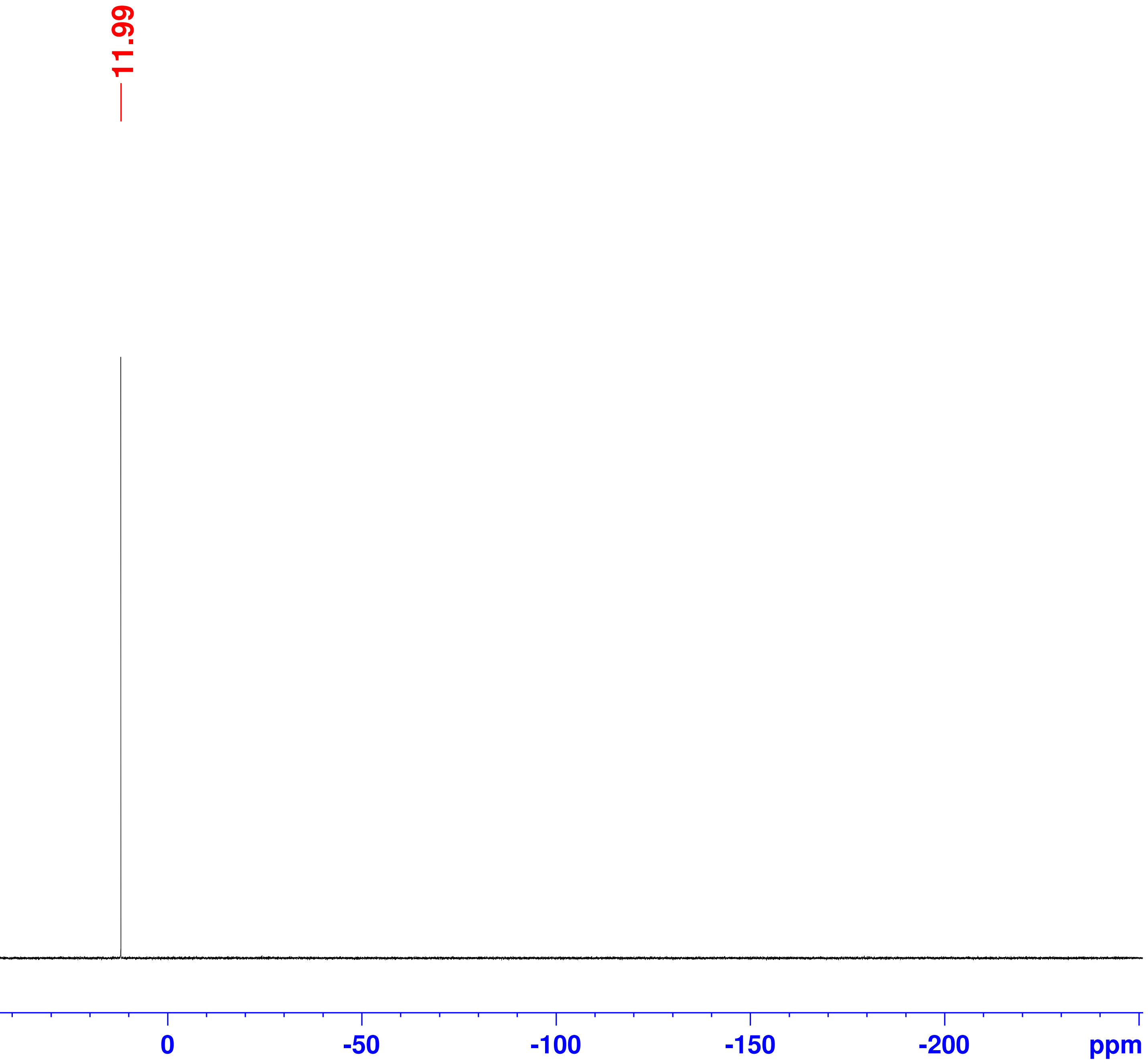

^{19}F NMR, 376 MHz, $CDCl_3$

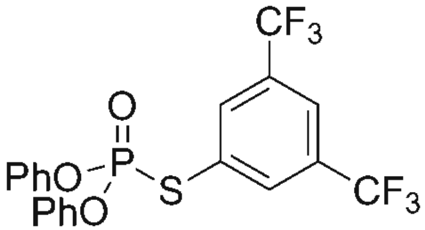

3g

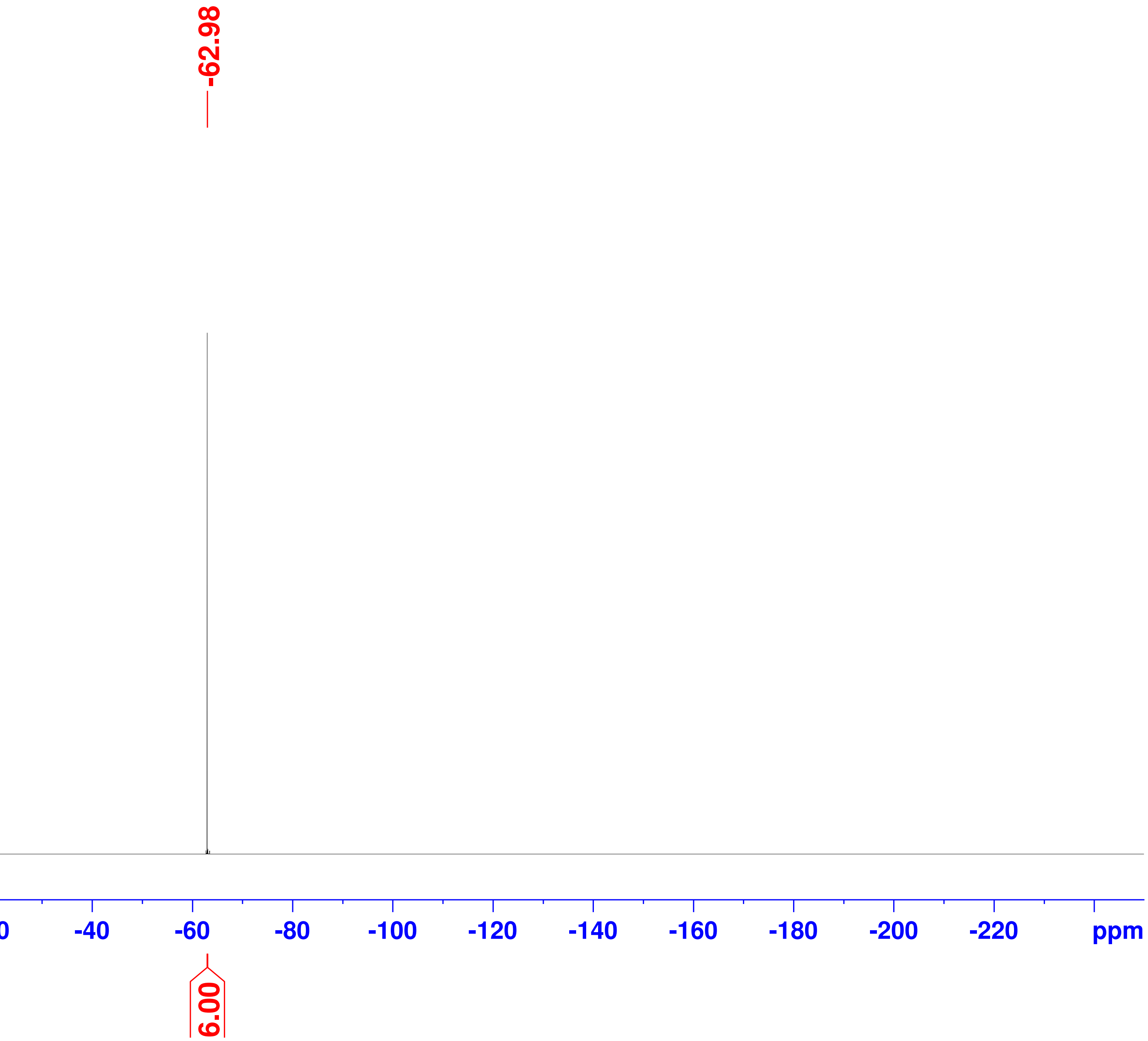

^{1}H NMR, 500 MHz, CDCl$_3$

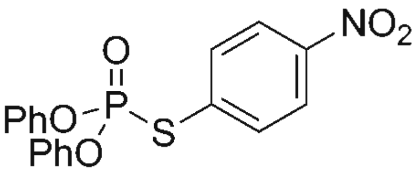

3h

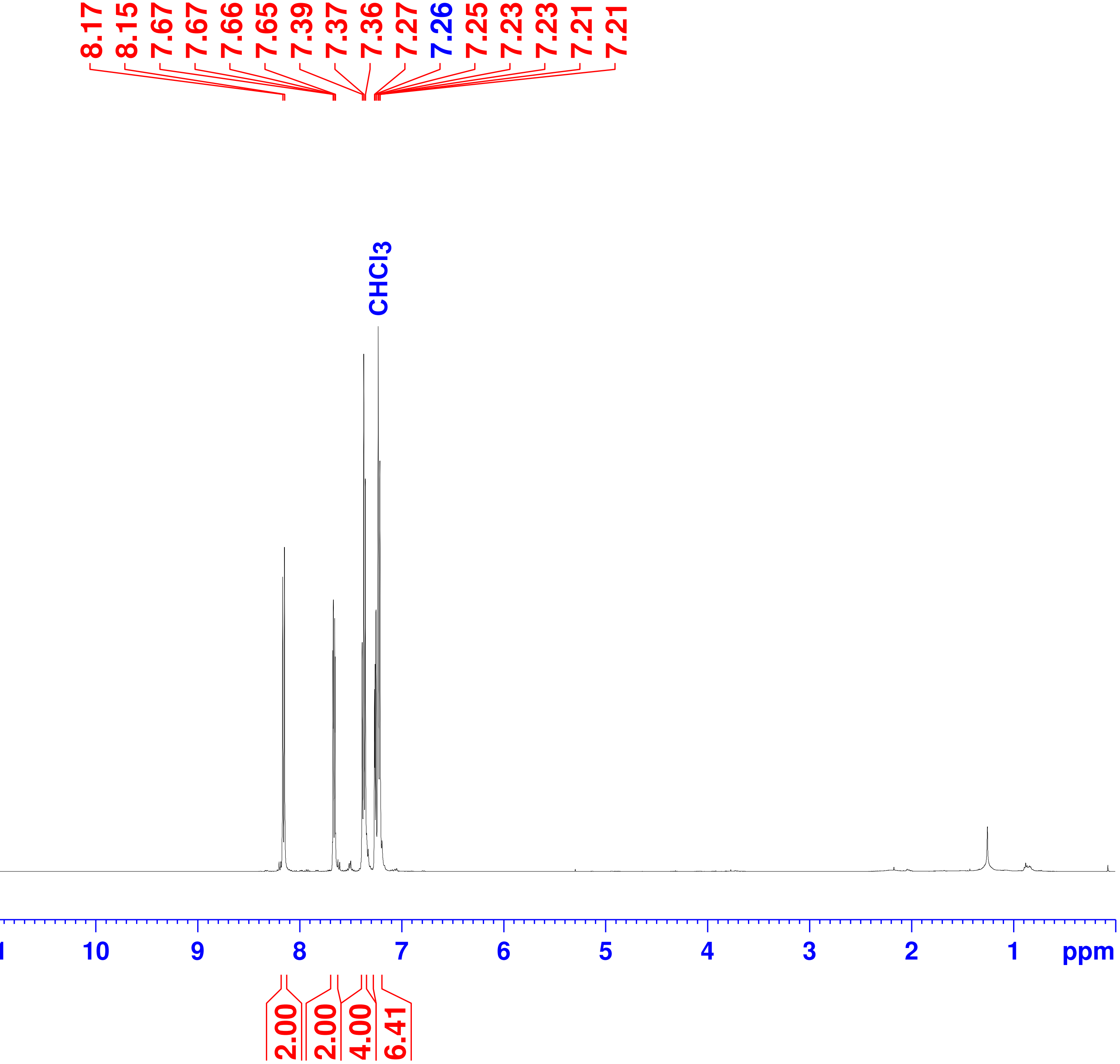

^{13}C NMR, 126 MHz, $CDCl_3$

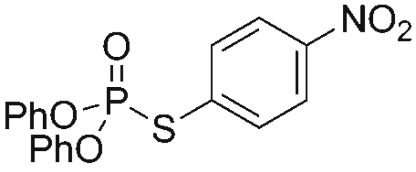

3h

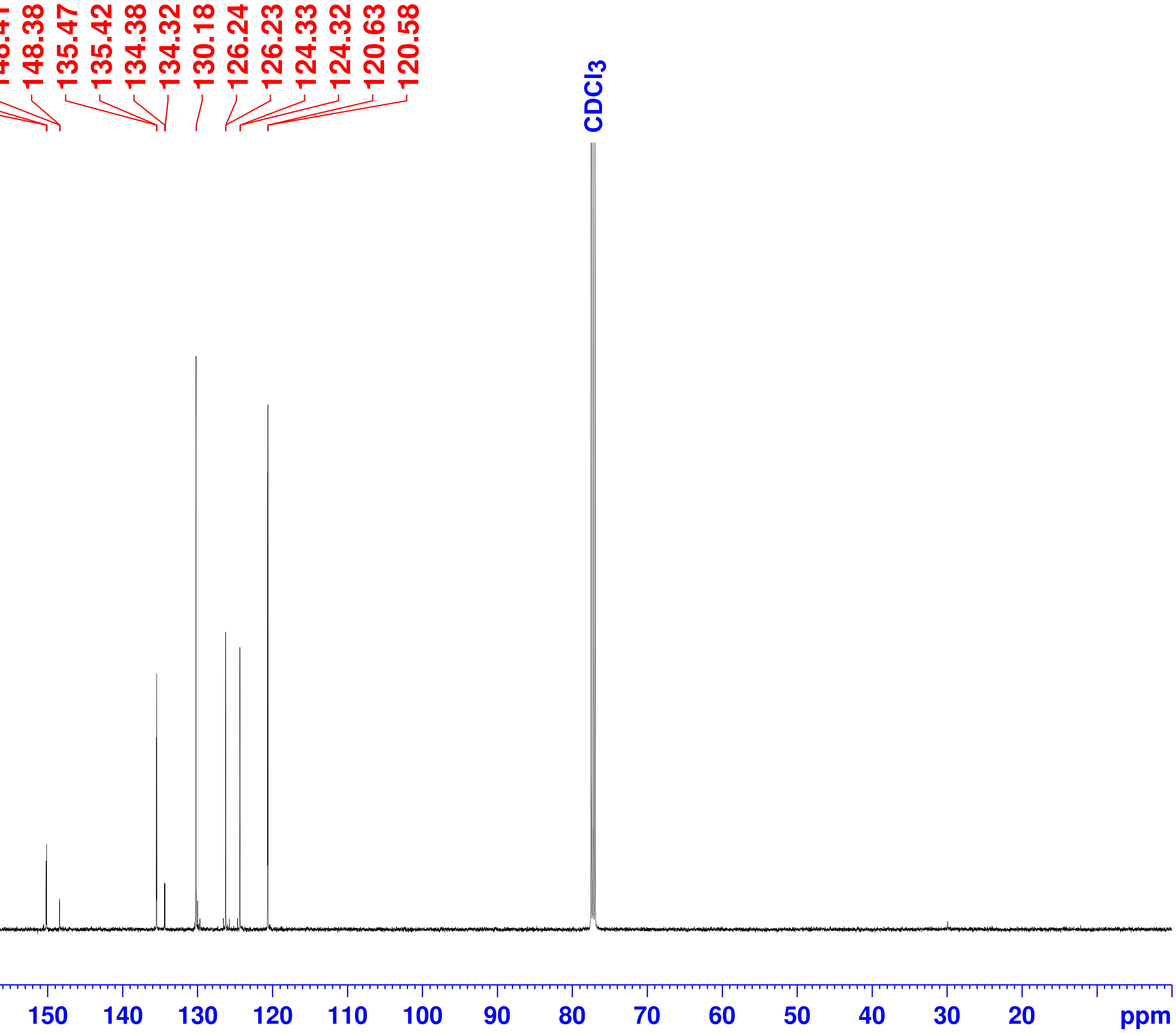

^{31}P NMR, 203 MHz, $CDCl_3$

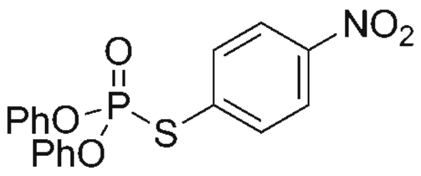

3h

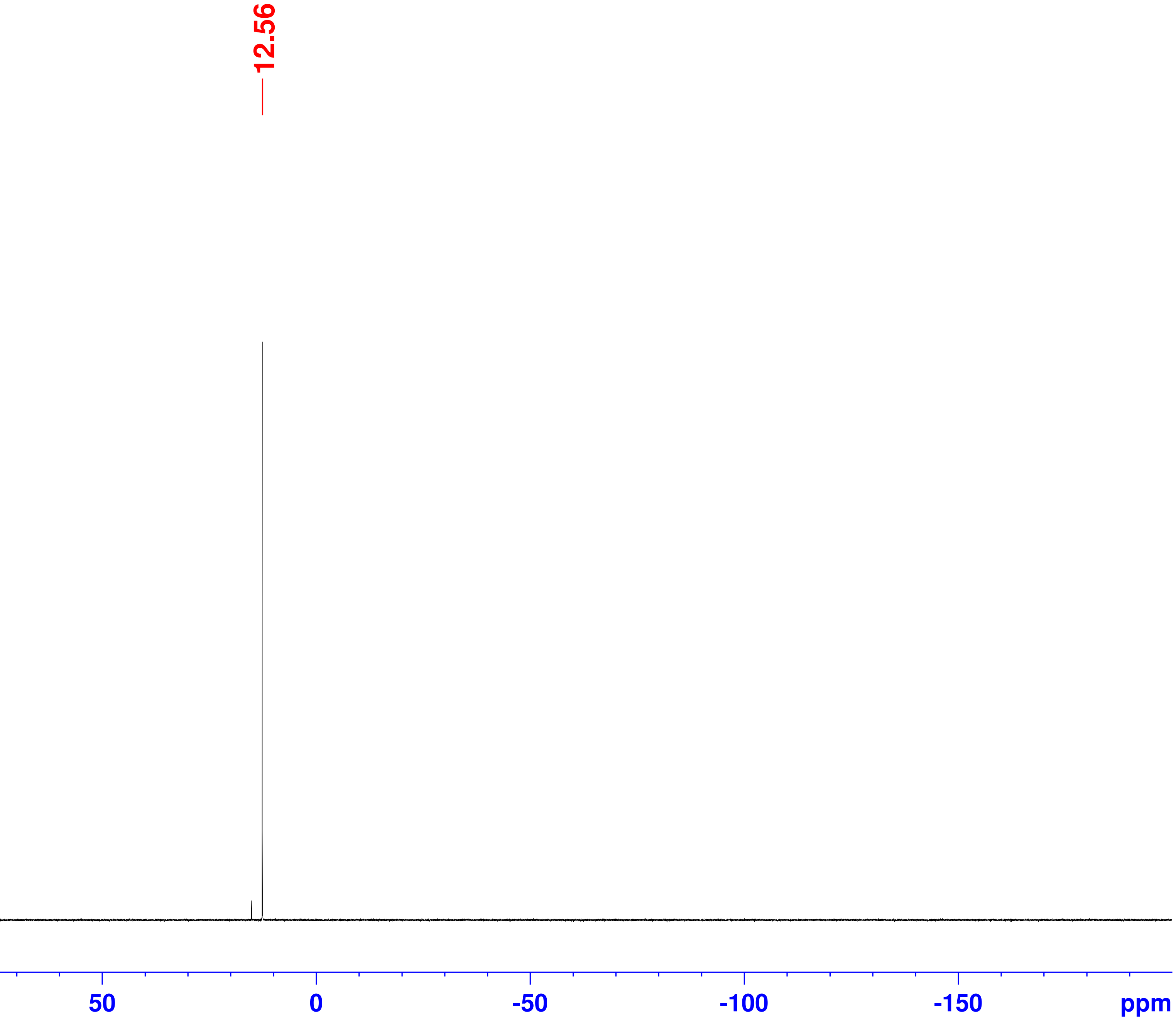

^{1}H NMR, 500 MHz, $CDCl_3$

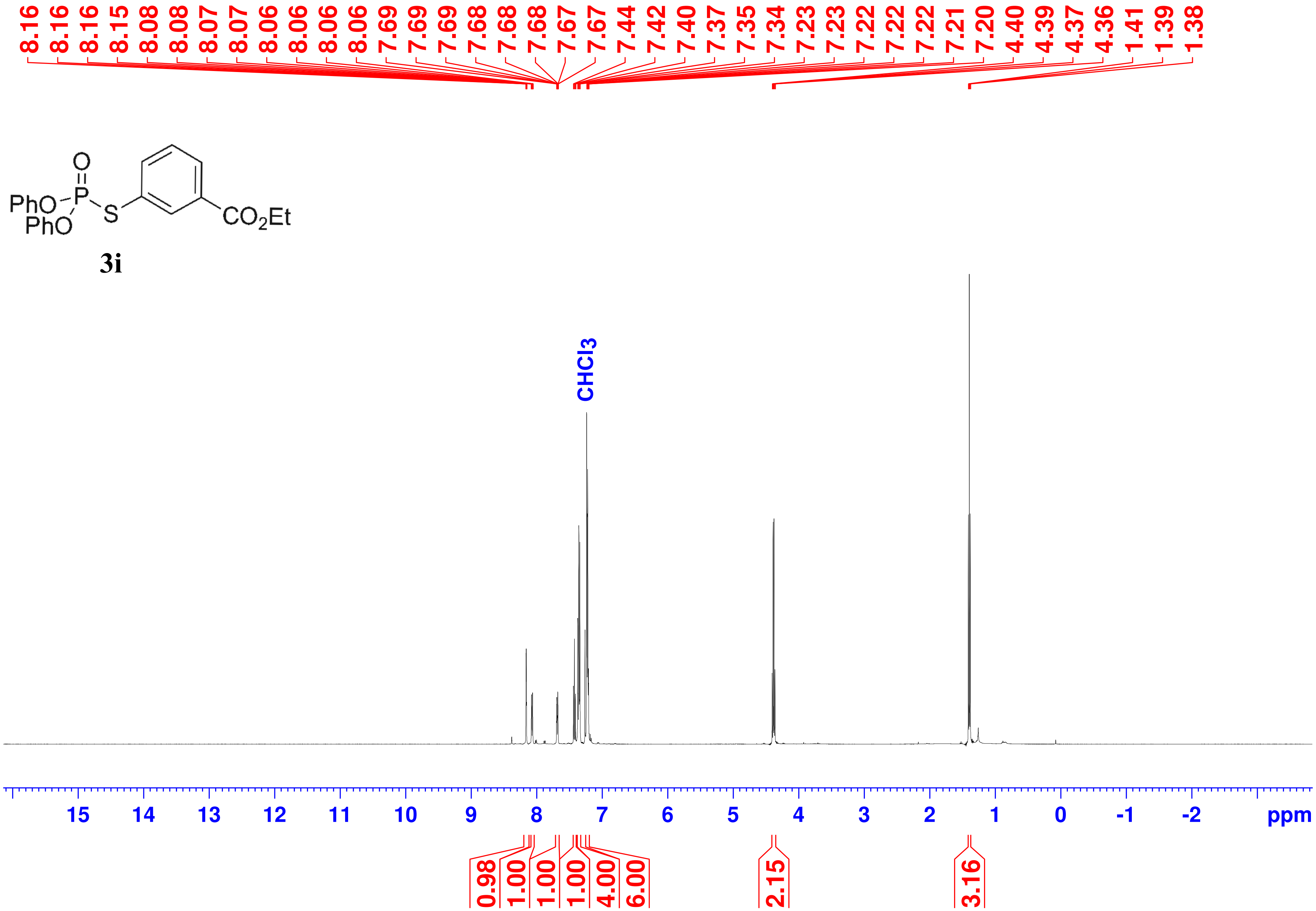

^{13}C NMR, 126 MHz, $CDCl_3$

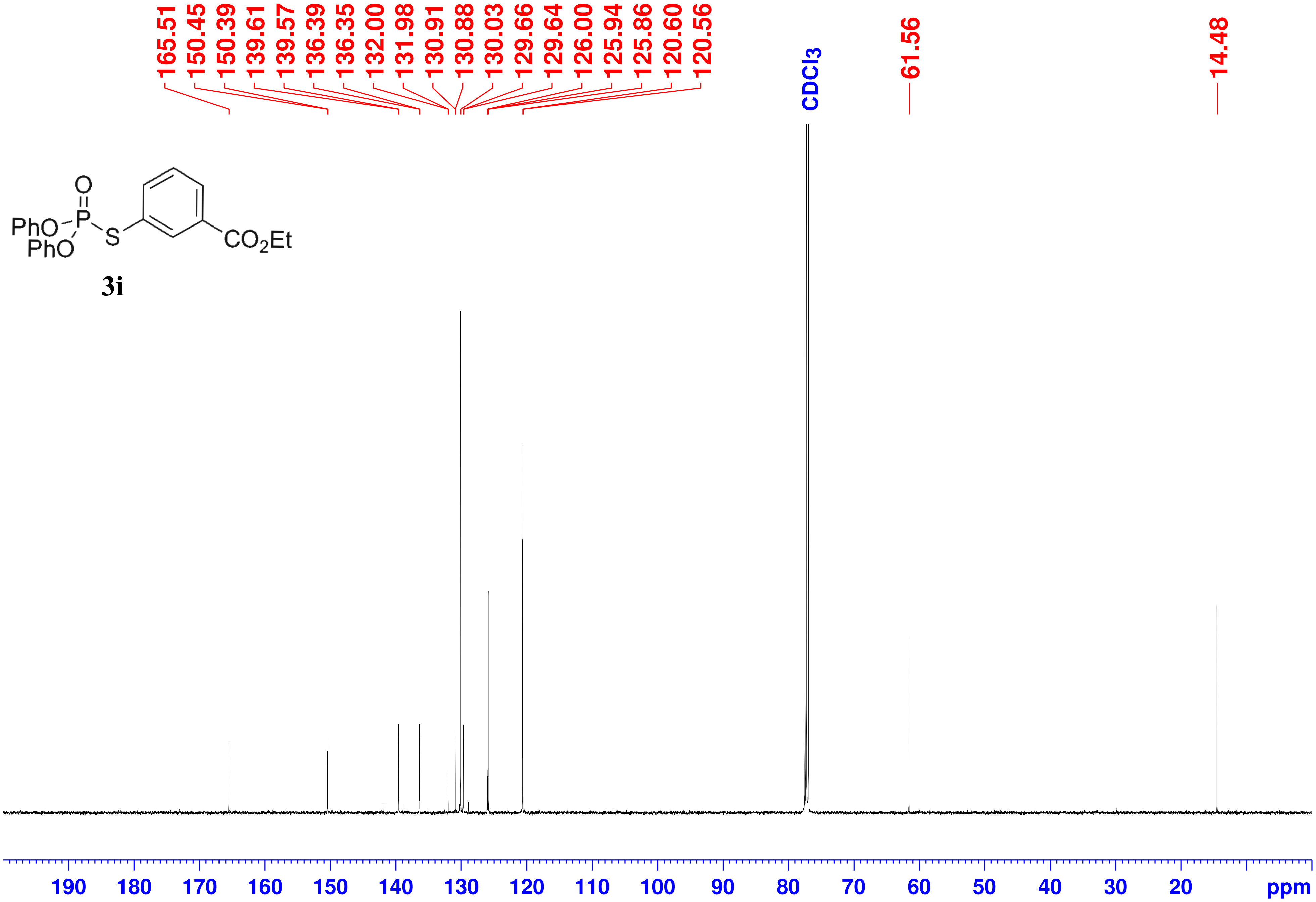

^{31}P NMR, 203 MHz, $CDCl_3$

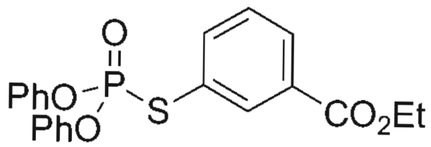

3i

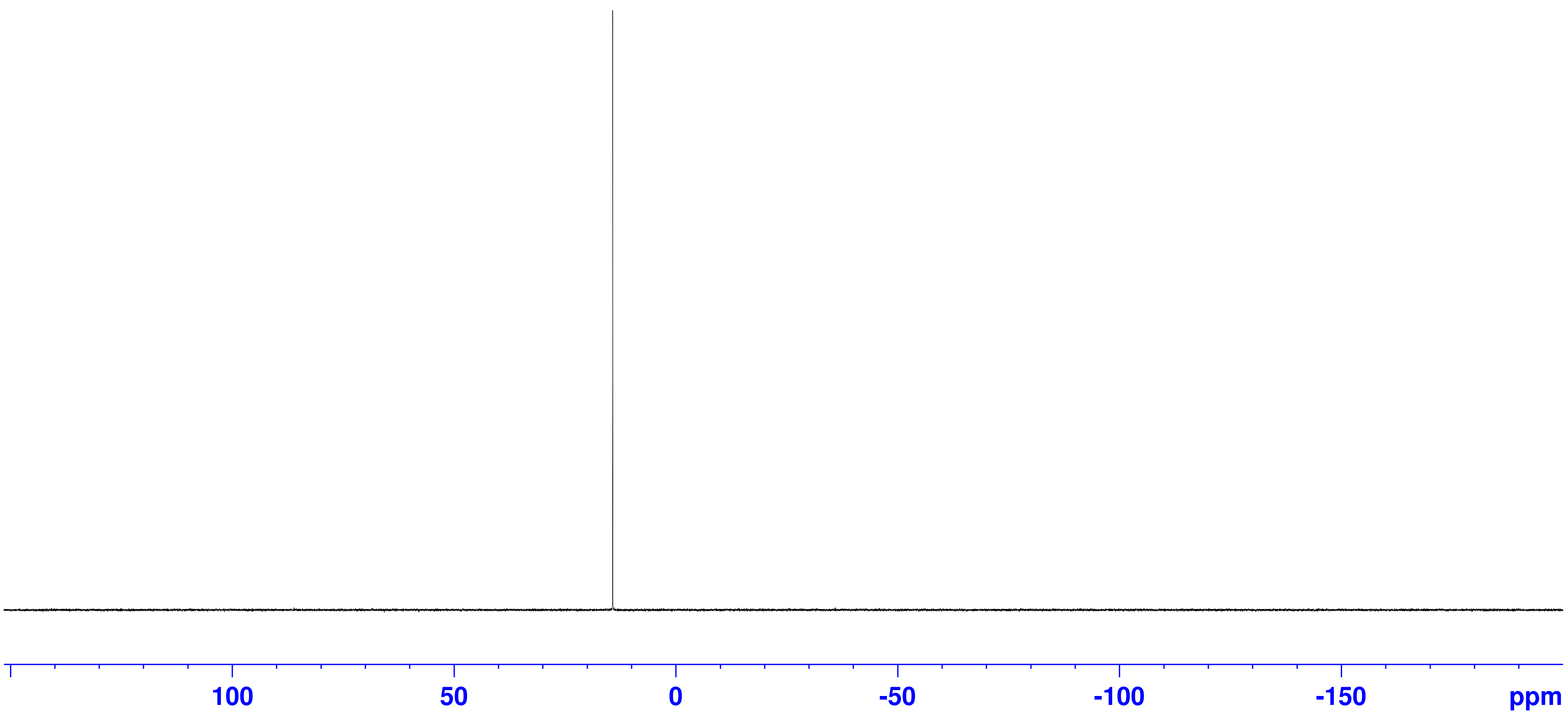

^{1}H NMR, 500 MHz, $CDCl_3$

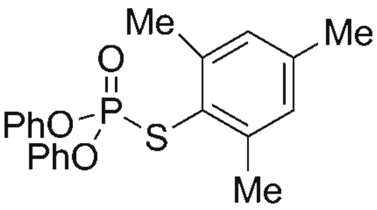

3j

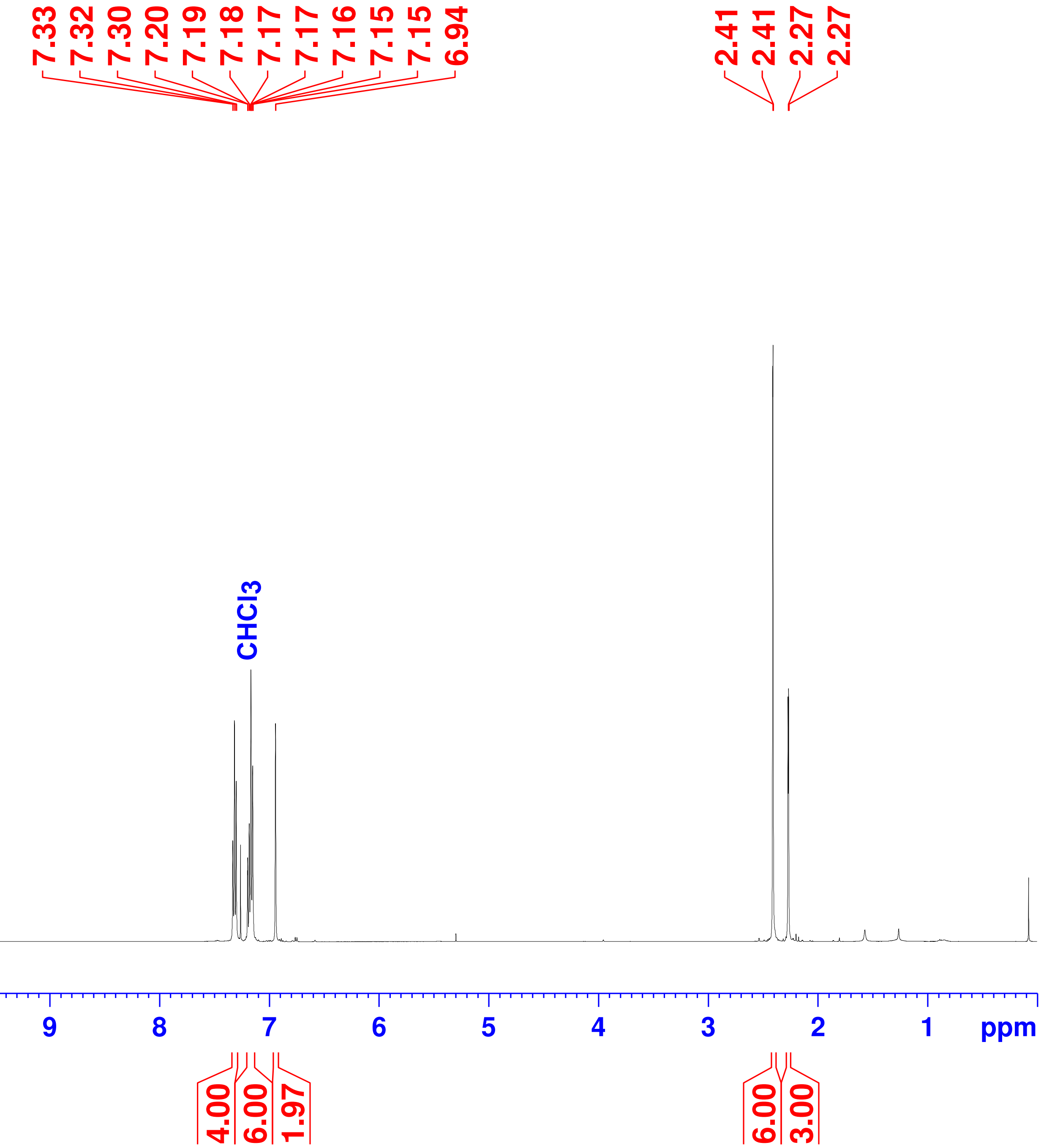

^{13}C NMR, 126 MHz, $CDCl_3$

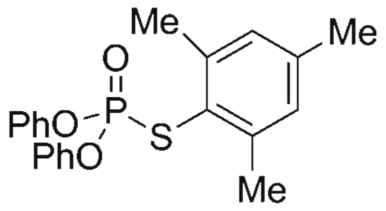

3j

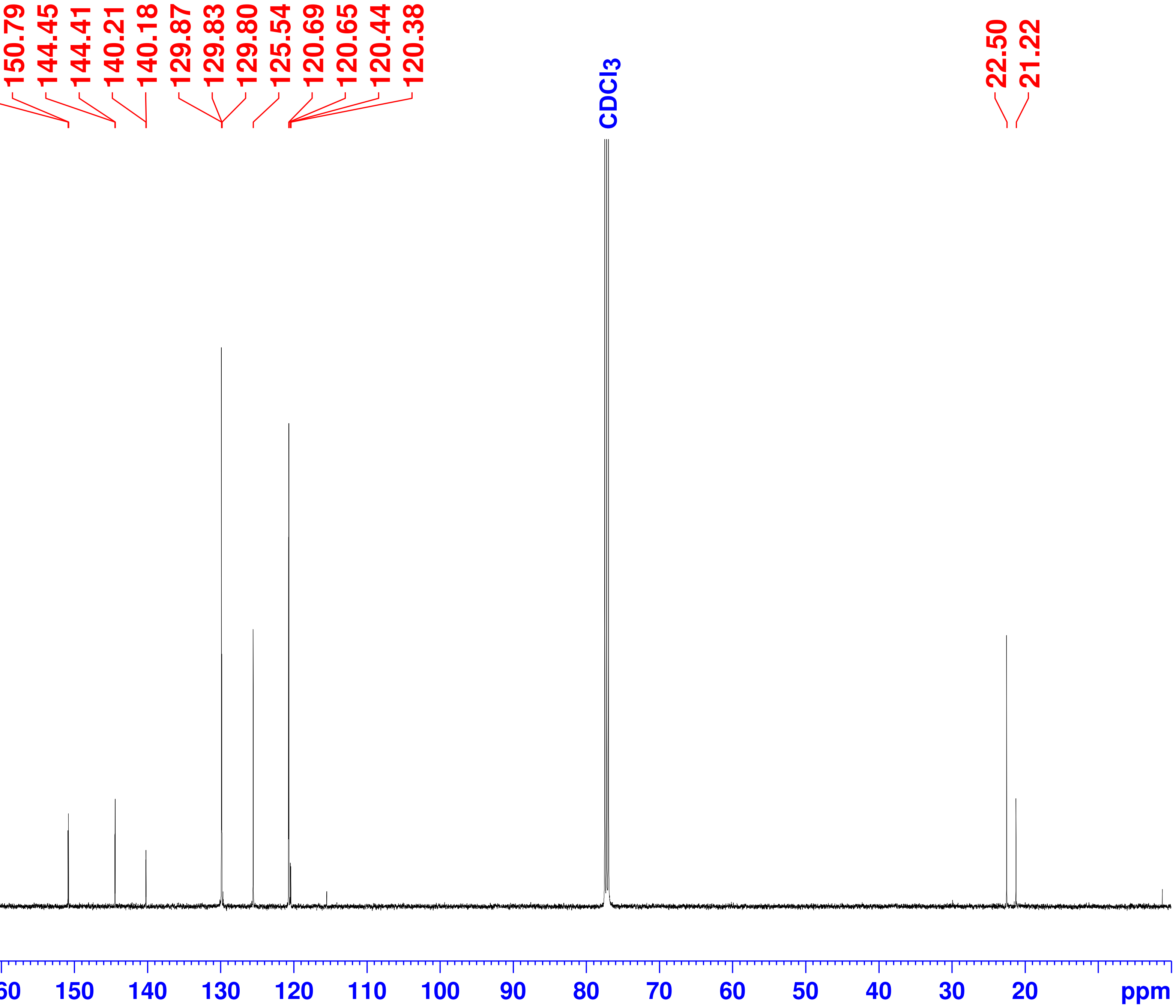

^{31}P NMR, 203 MHz, $CDCl_3$

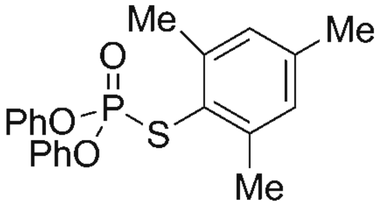

3j

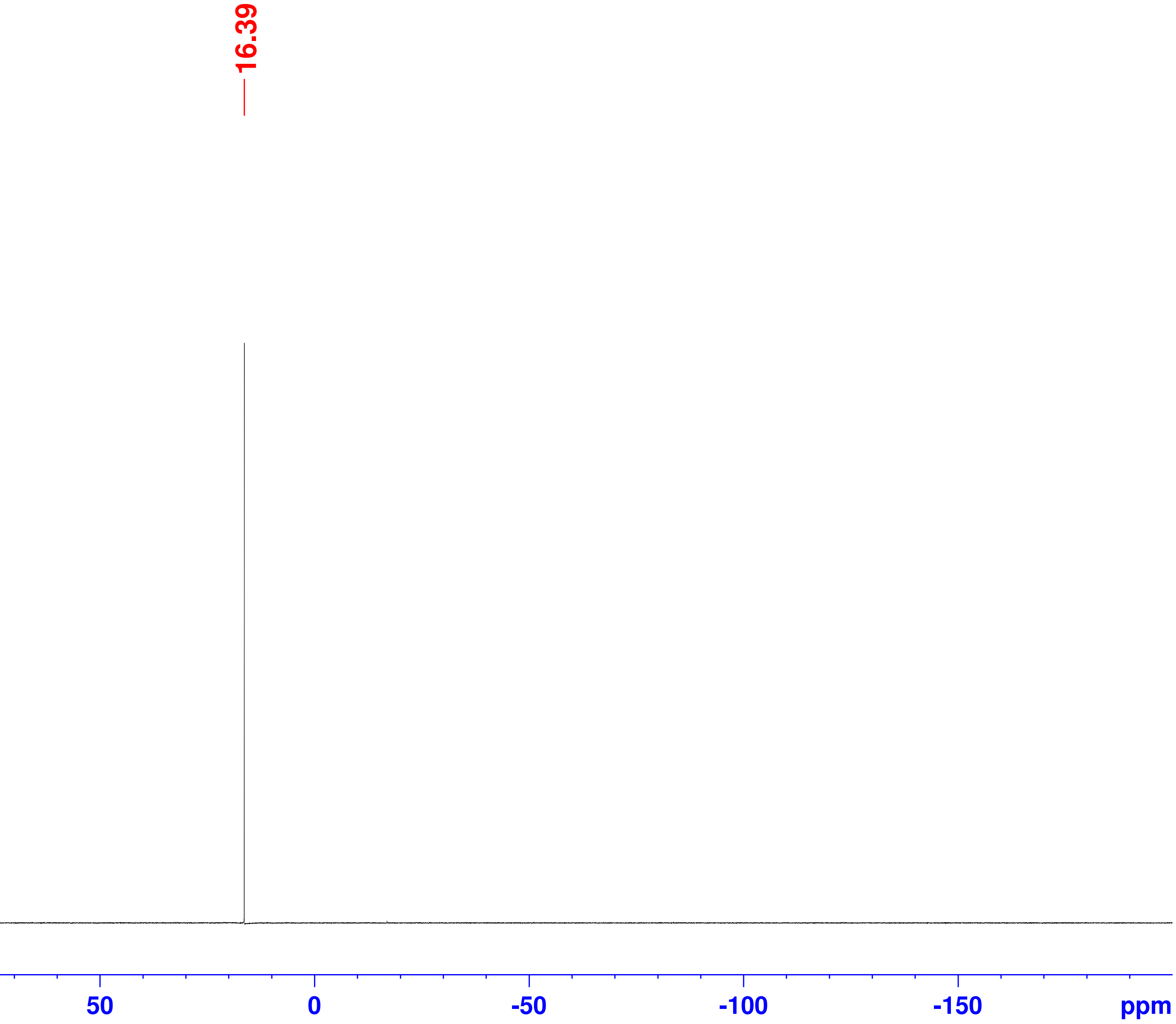

^{1}H NMR, 500 MHz, $CDCl_3$

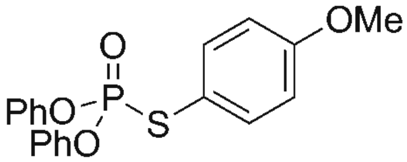

3k

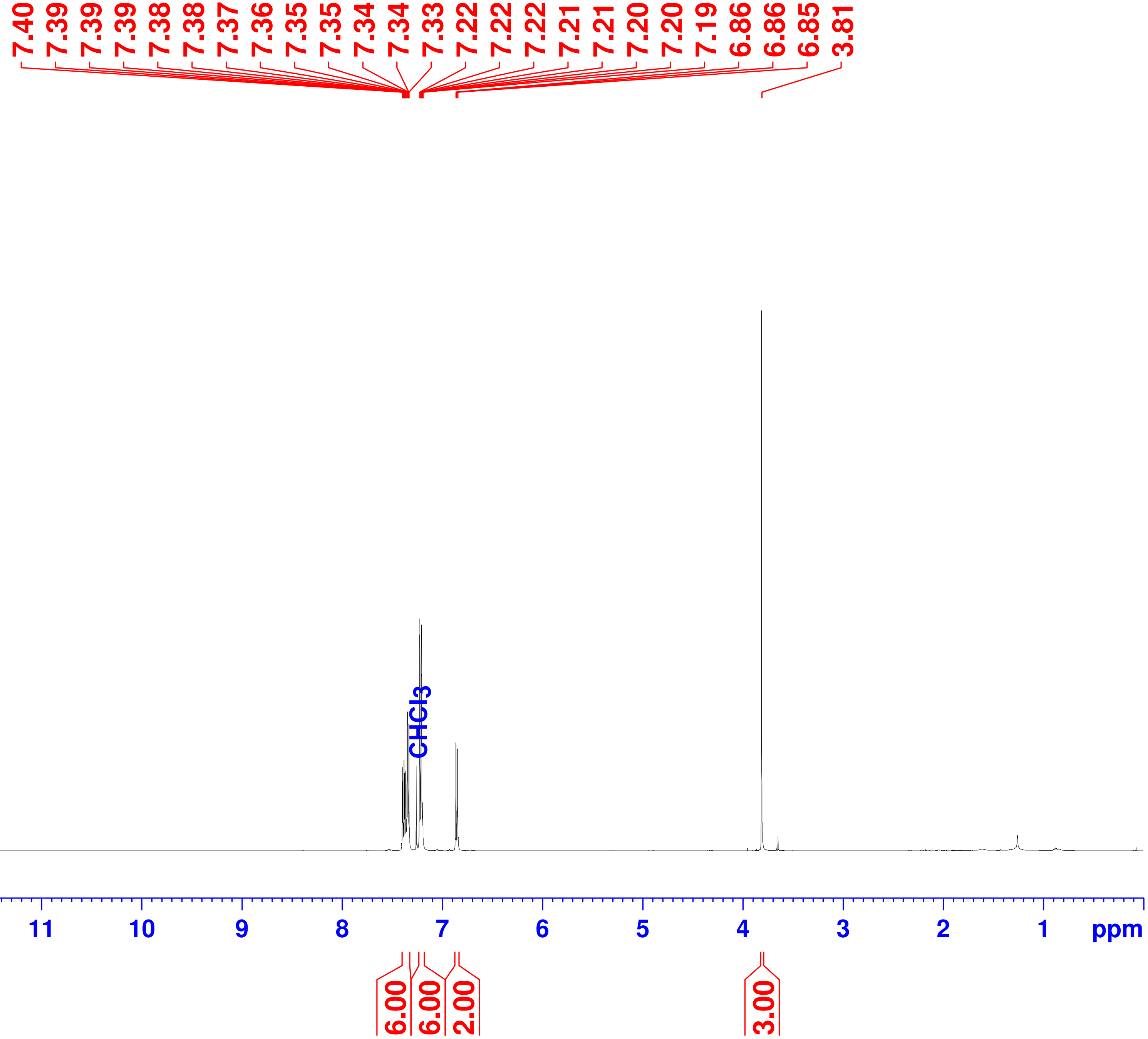

^{13}C NMR, 126 MHz, $CDCl_3$

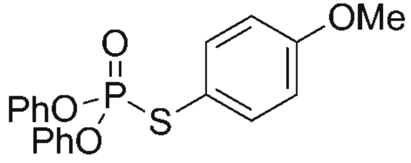

3k

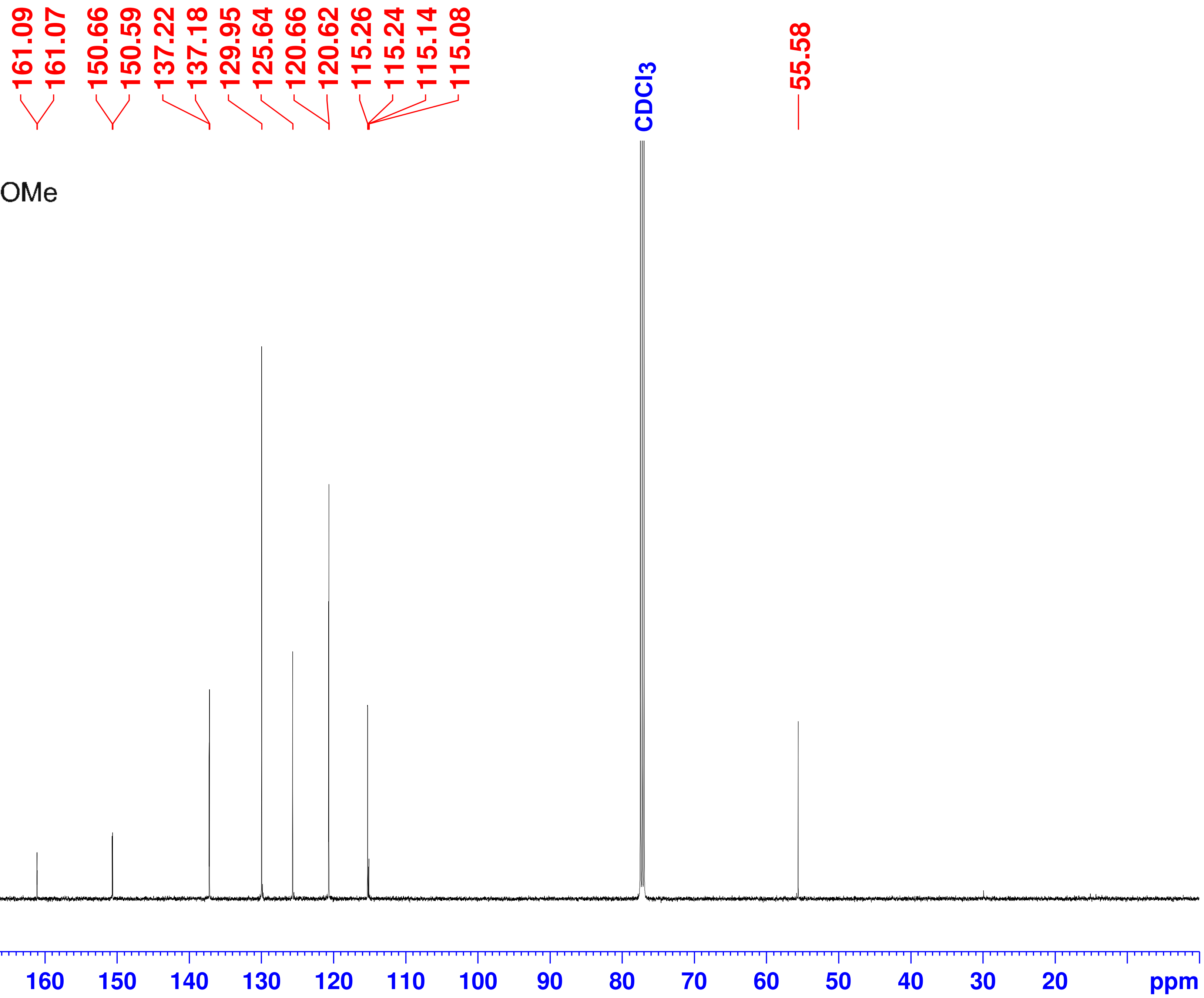

^{31}P NMR, 203 MHz, CDCl$_3$

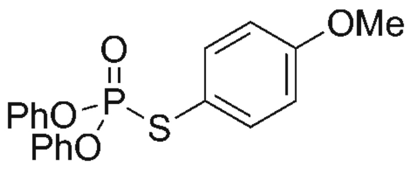

3k

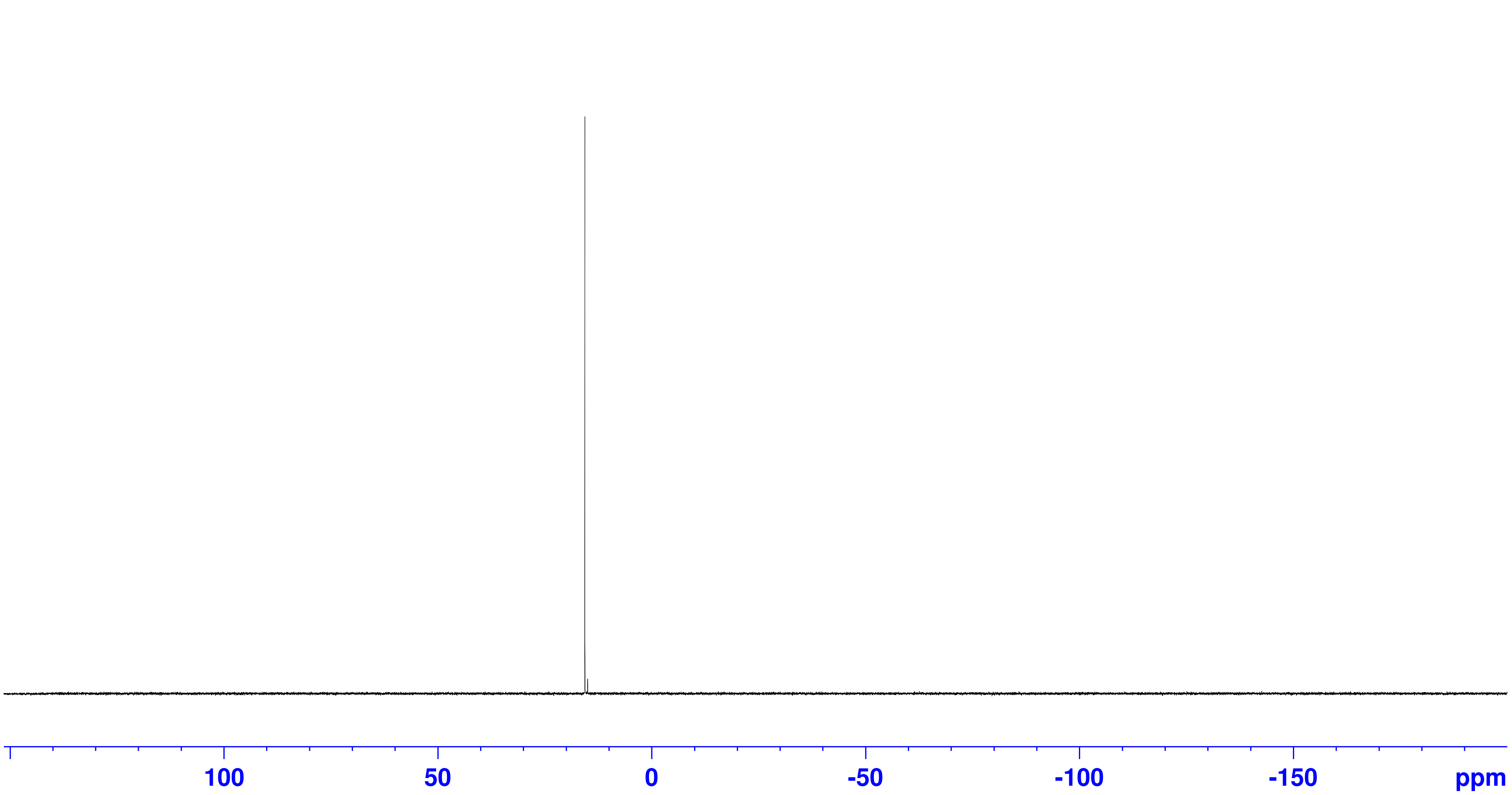

^{1}H NMR, 500 MHz, $CDCl_3$

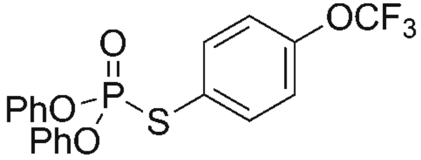

3l

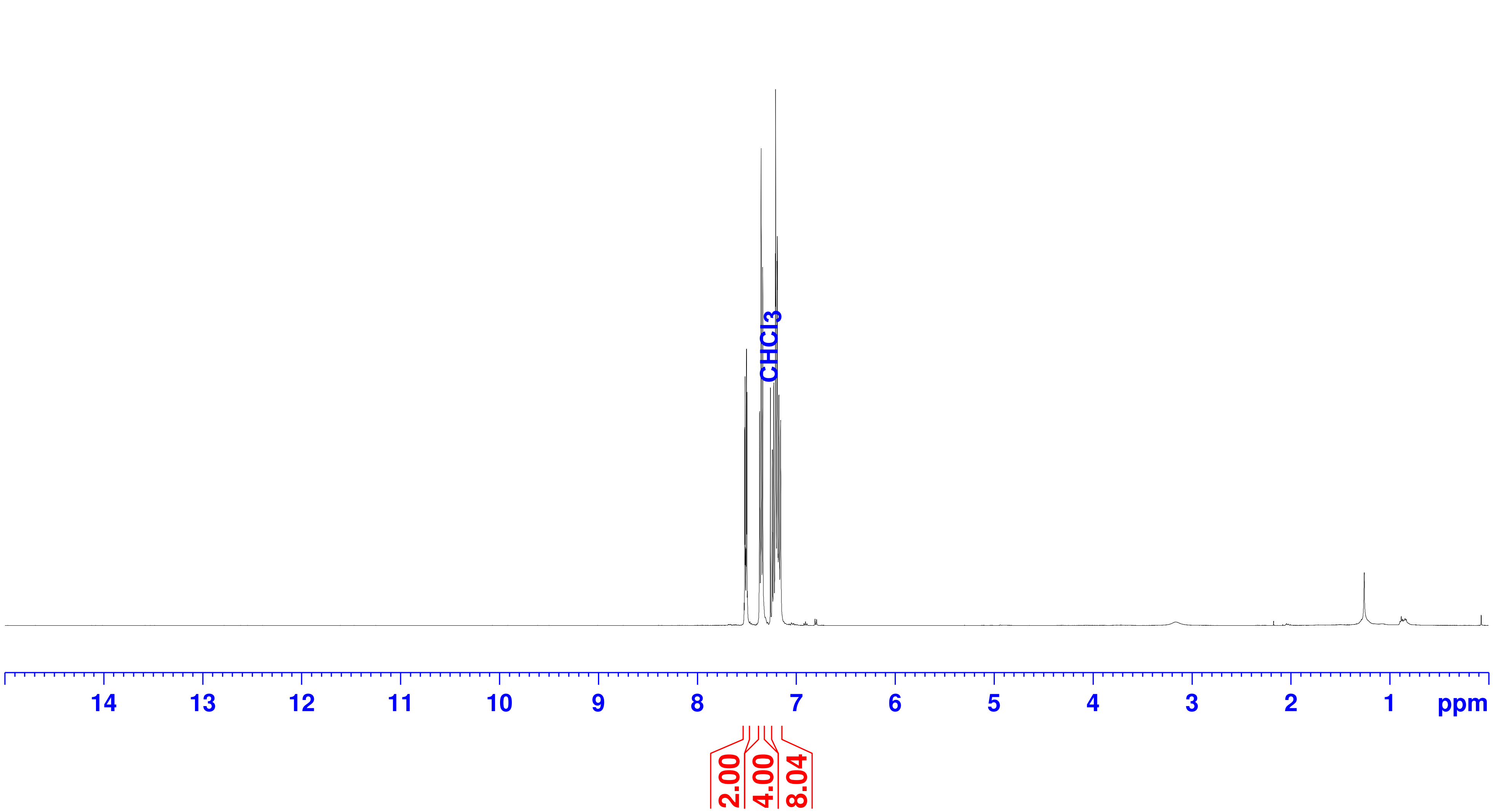

^{13}C NMR, 126 MHz, $CDCl_3$

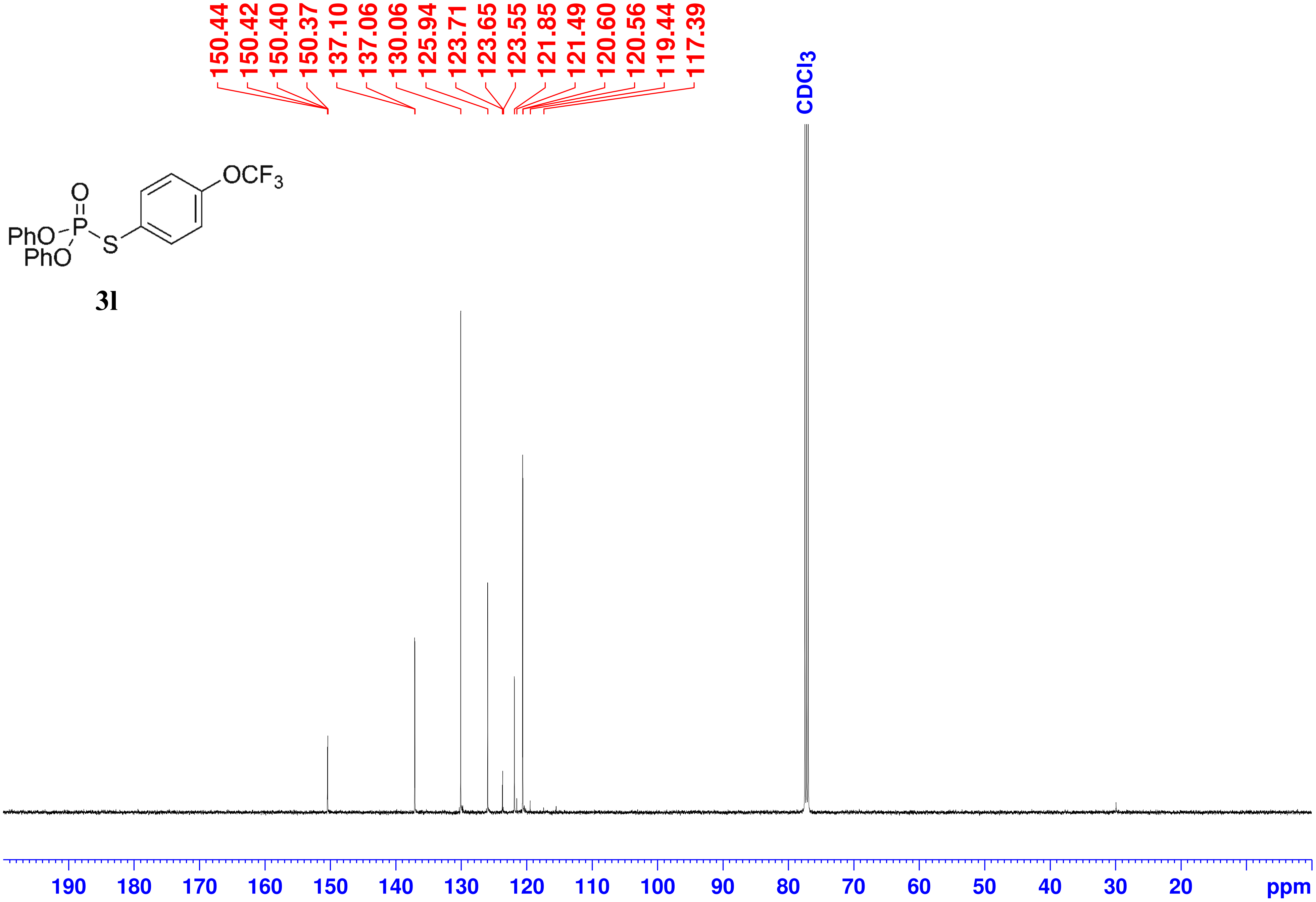

^{31}P NMR, 203 MHz, CDCl$_3$

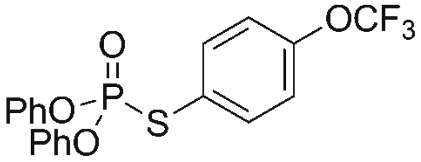

3l

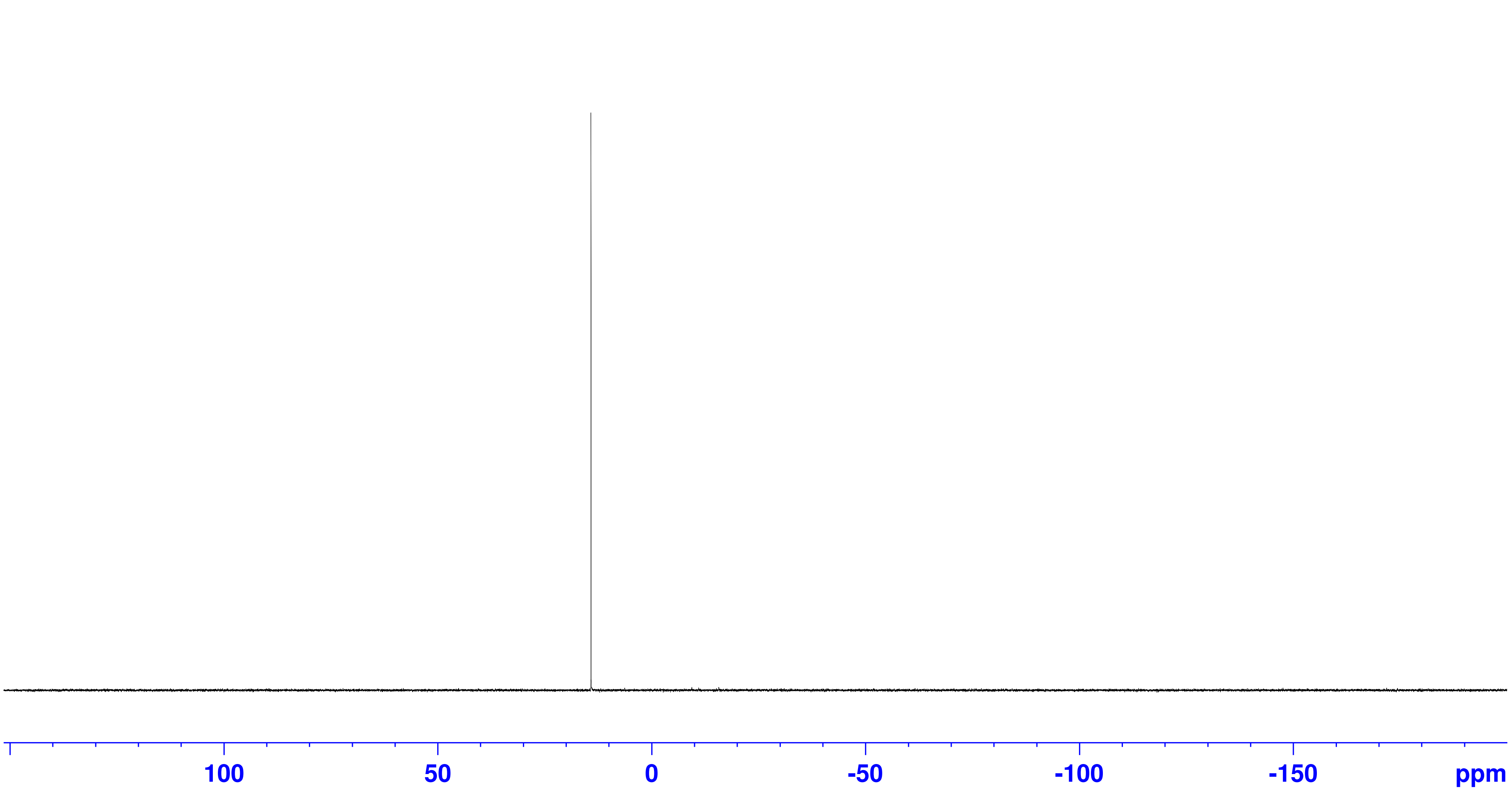

^{19}F NMR, 376 MHz, $CDCl_3$

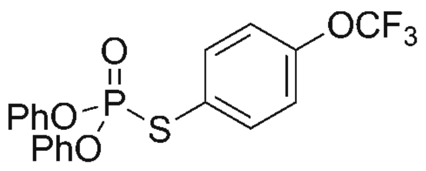

3l

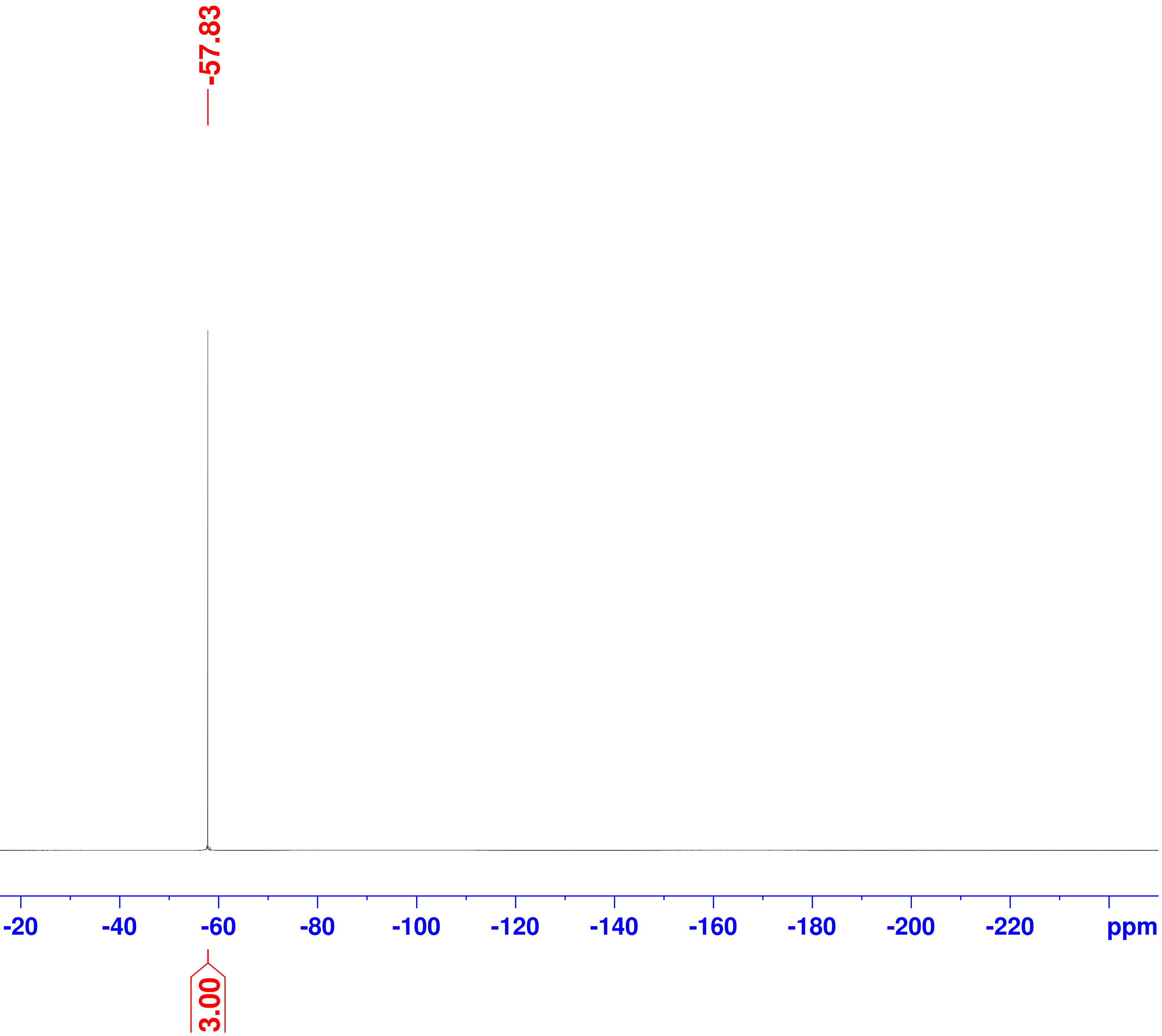

^{1}H NMR, 500 MHz, $CDCl_3$

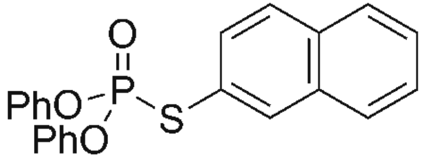

3m

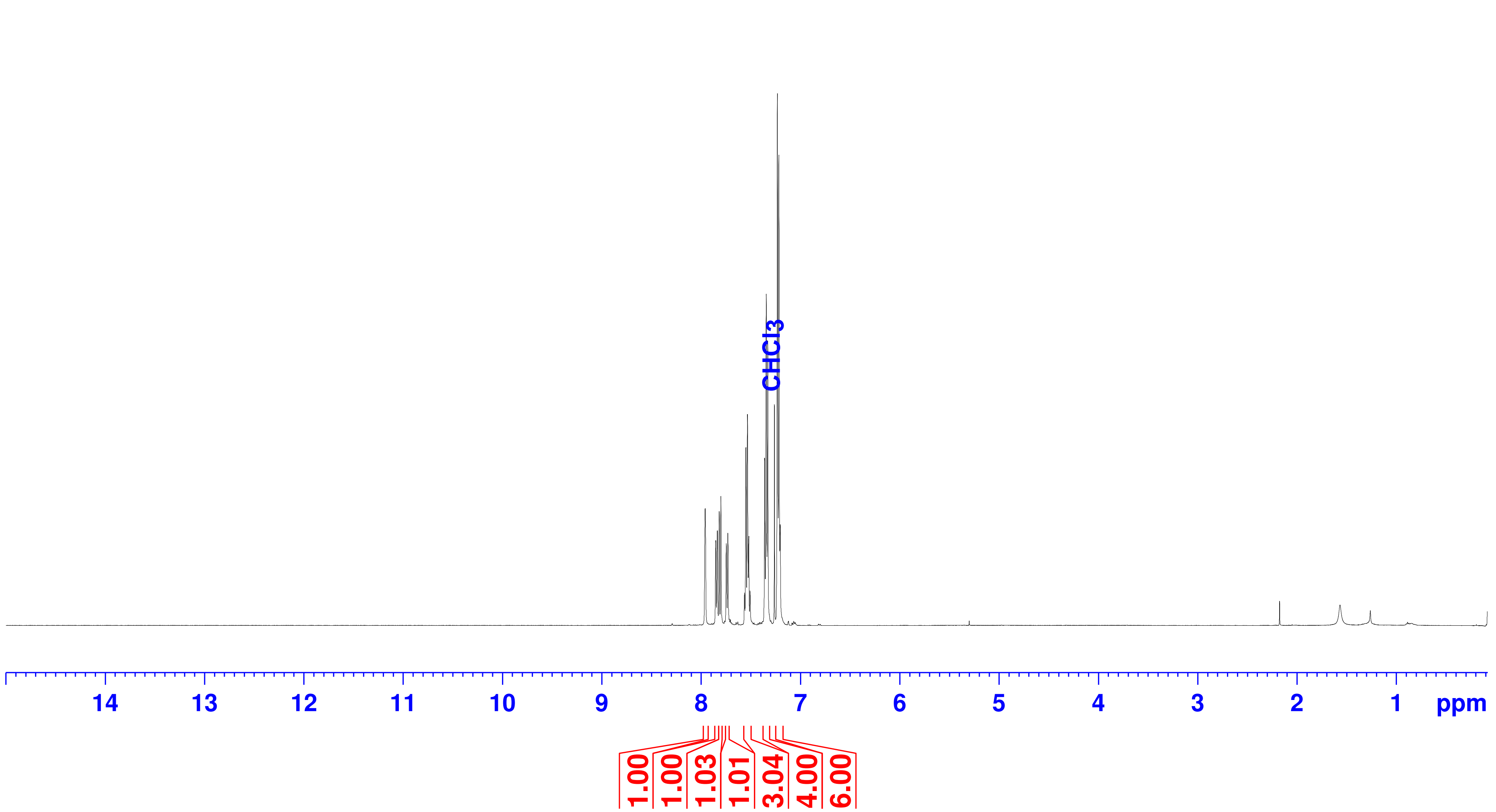

^{13}C NMR, 126 MHz, $CDCl_3$

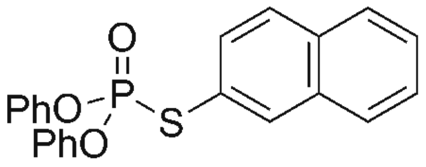

3m

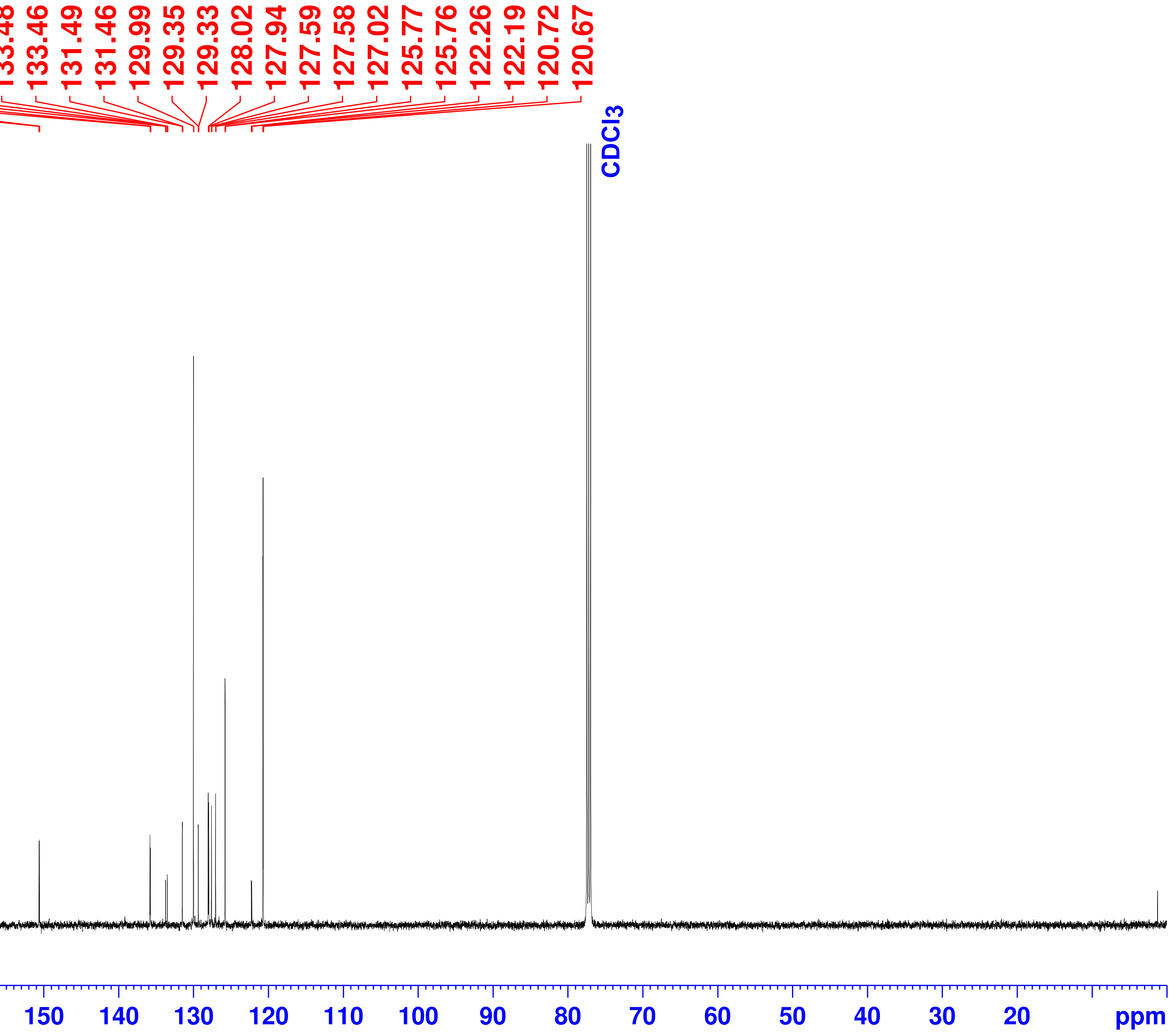

^{31}P NMR, 203 MHz, $CDCl_3$

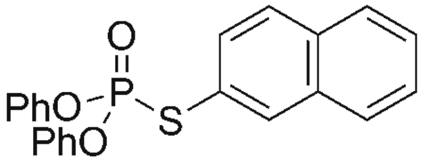

3m

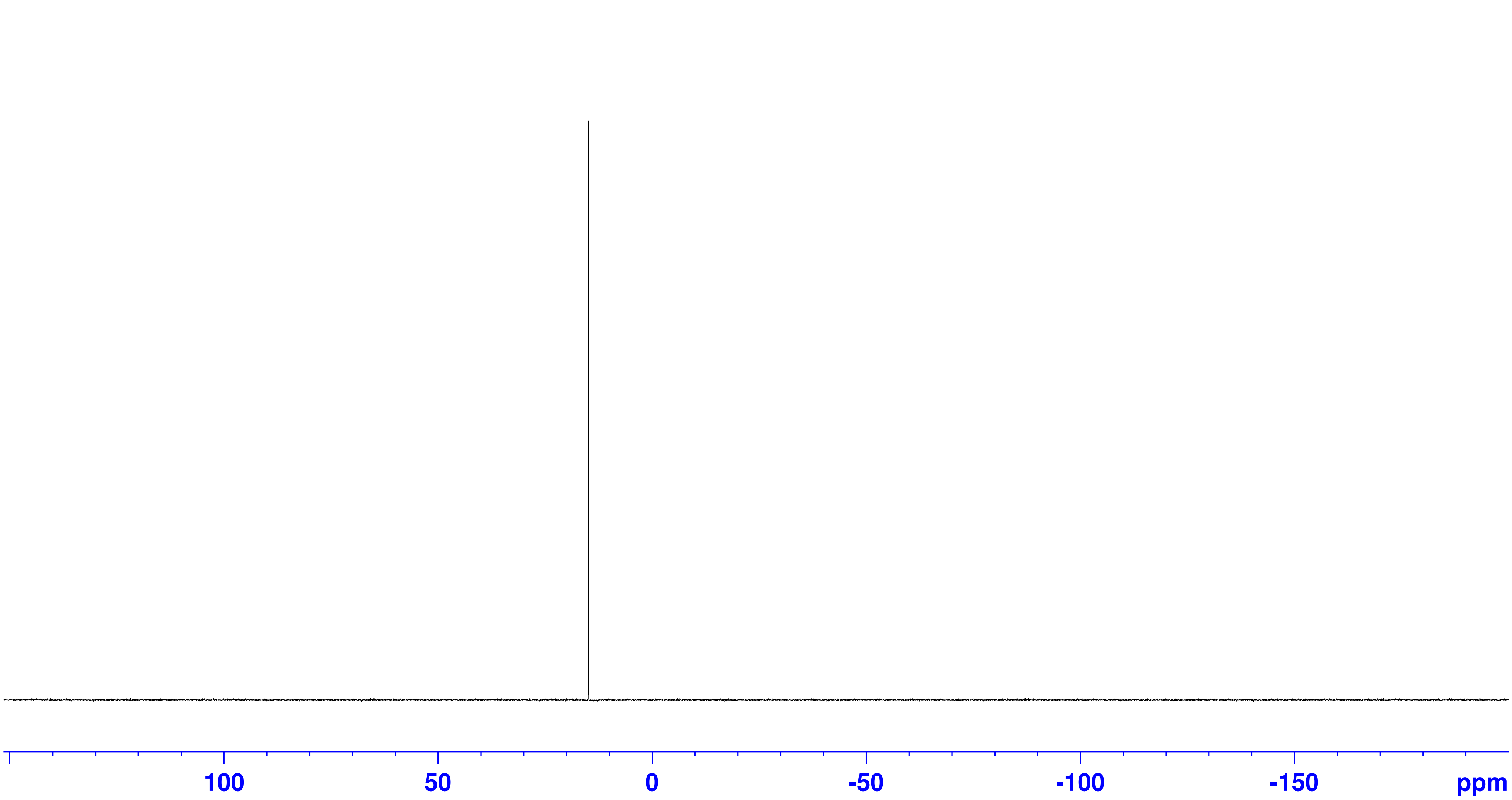

^{1}H NMR, 500 MHz, $CDCl_3$

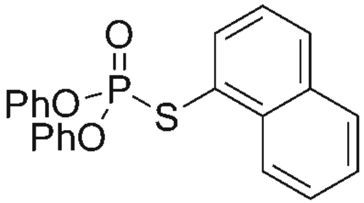

3n

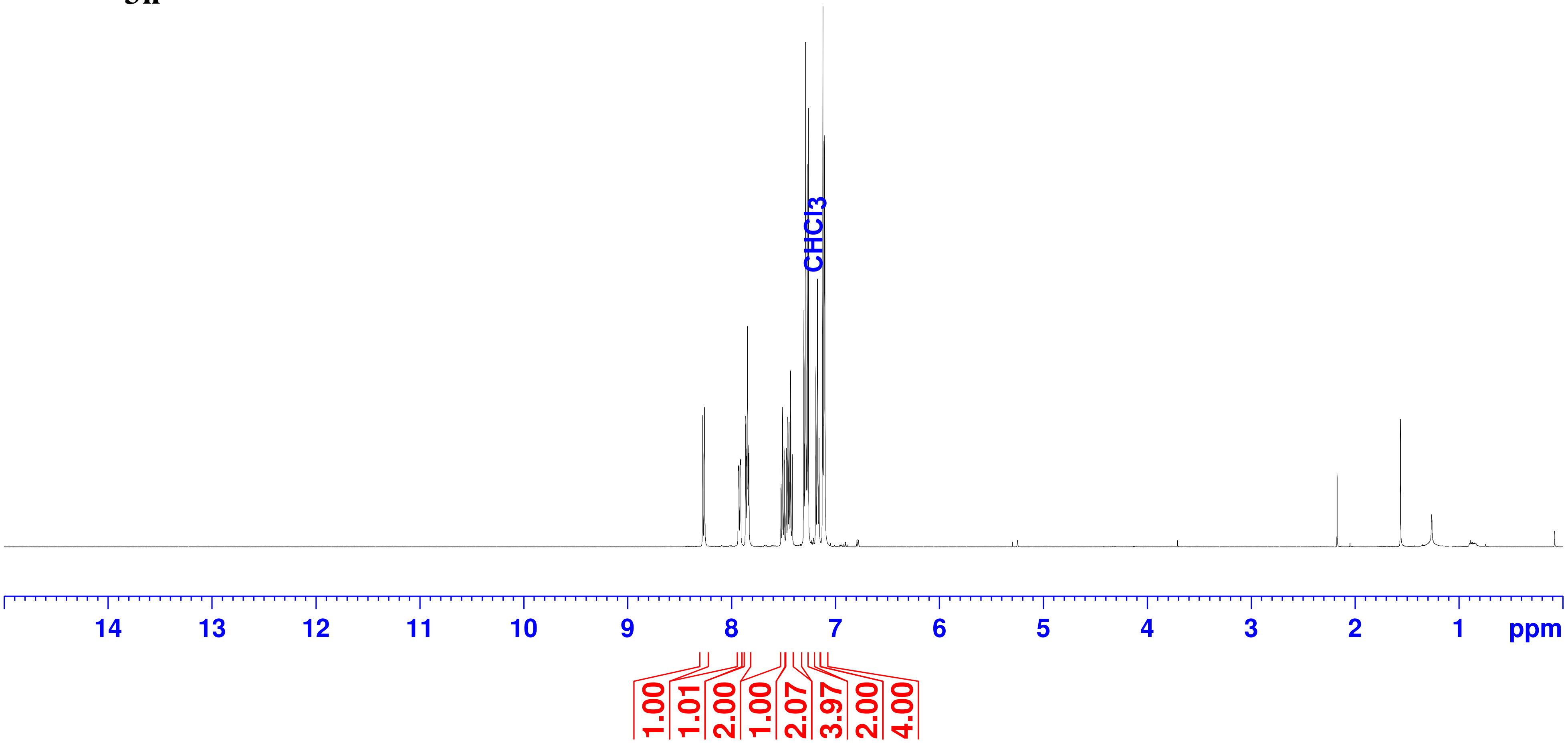

^{13}C NMR, 126 MHz, $CDCl_3$

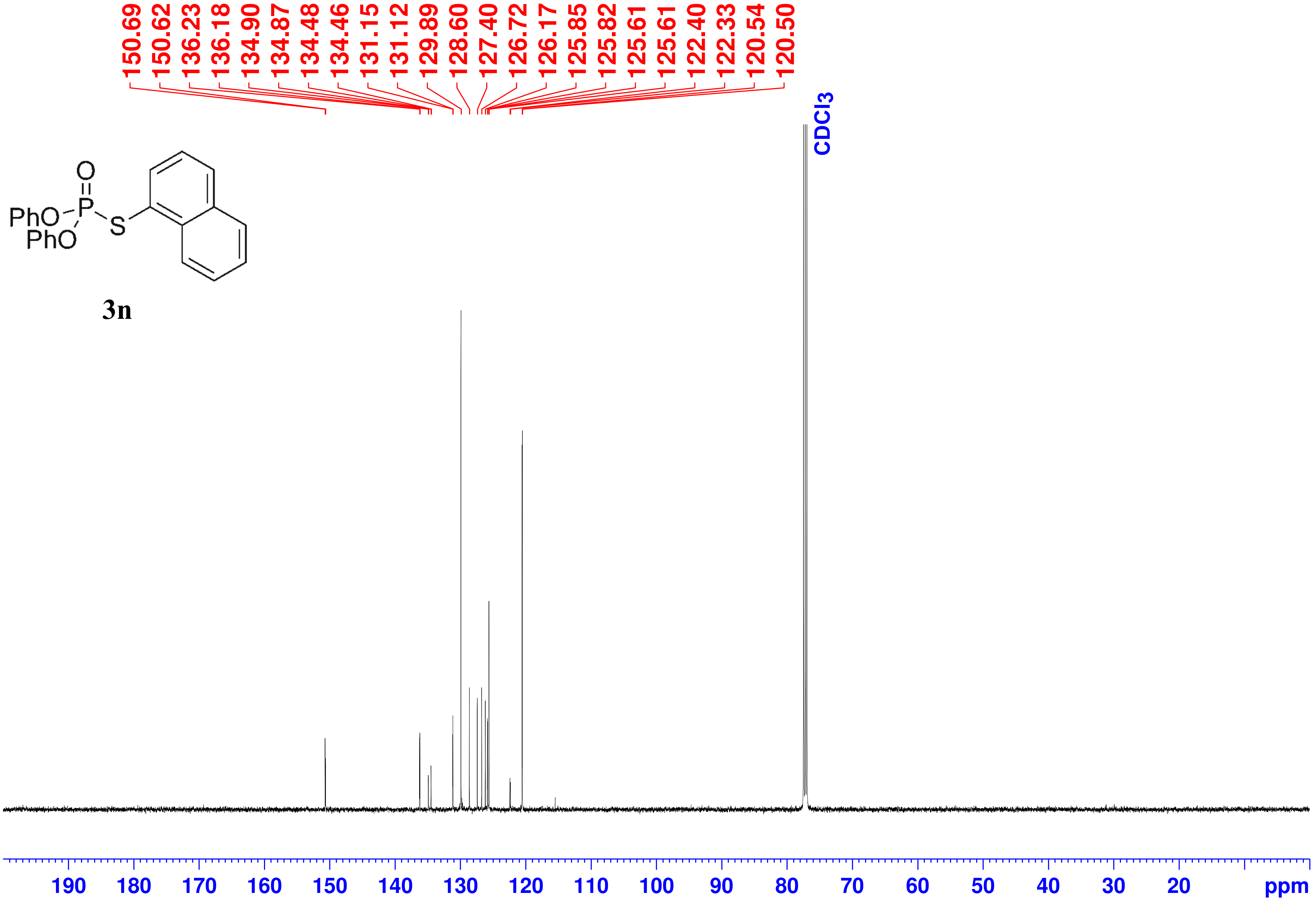

3n

^{31}P NMR, 203 MHz, $CDCl_3$

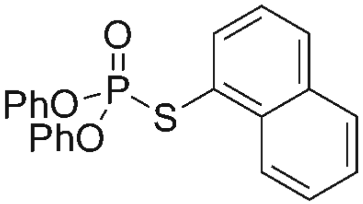

3n

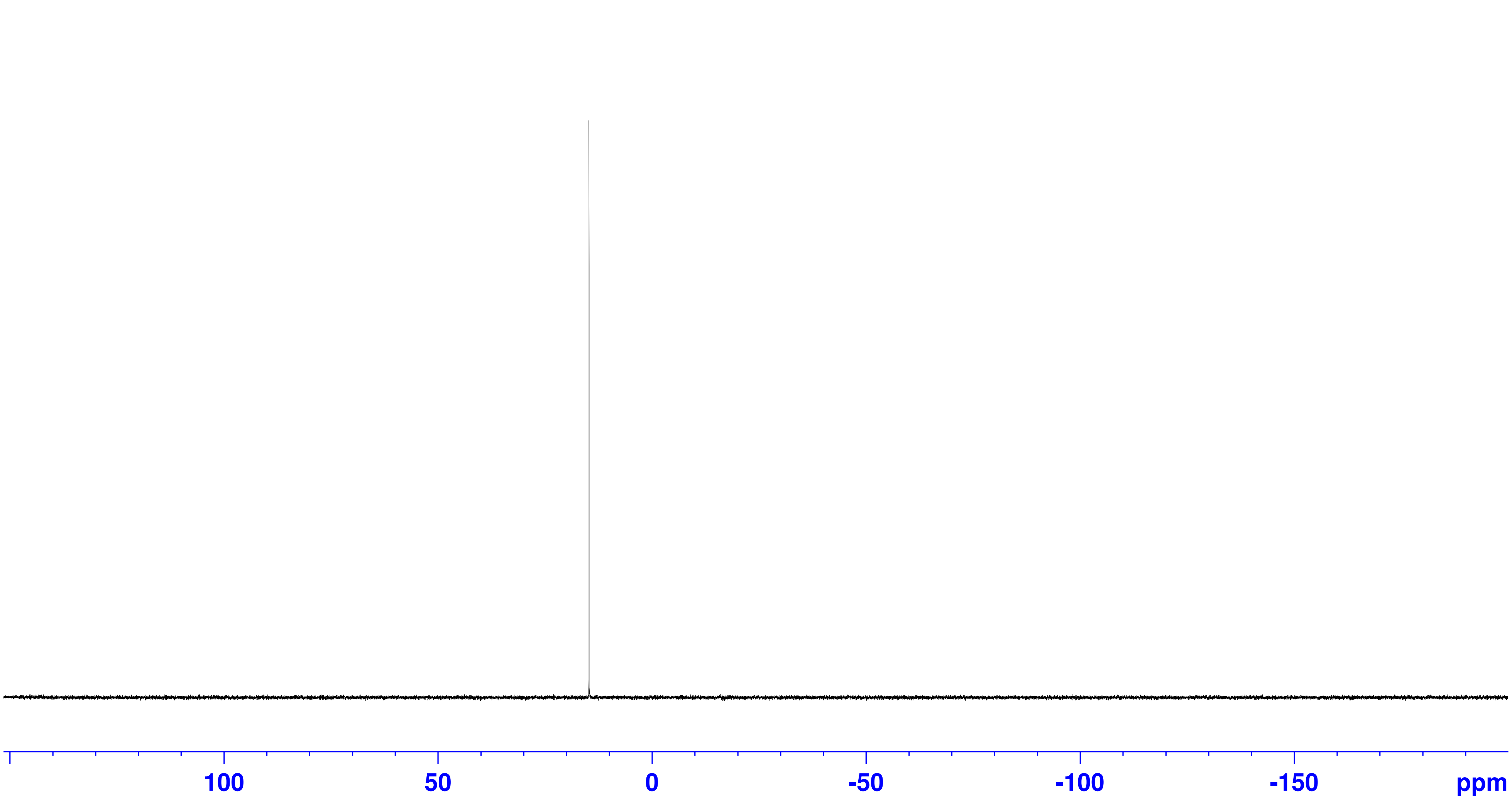

^{1}H NMR, 500 MHz, $CDCl_3$

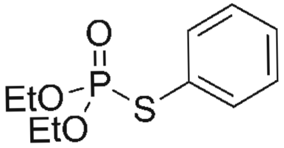

3o

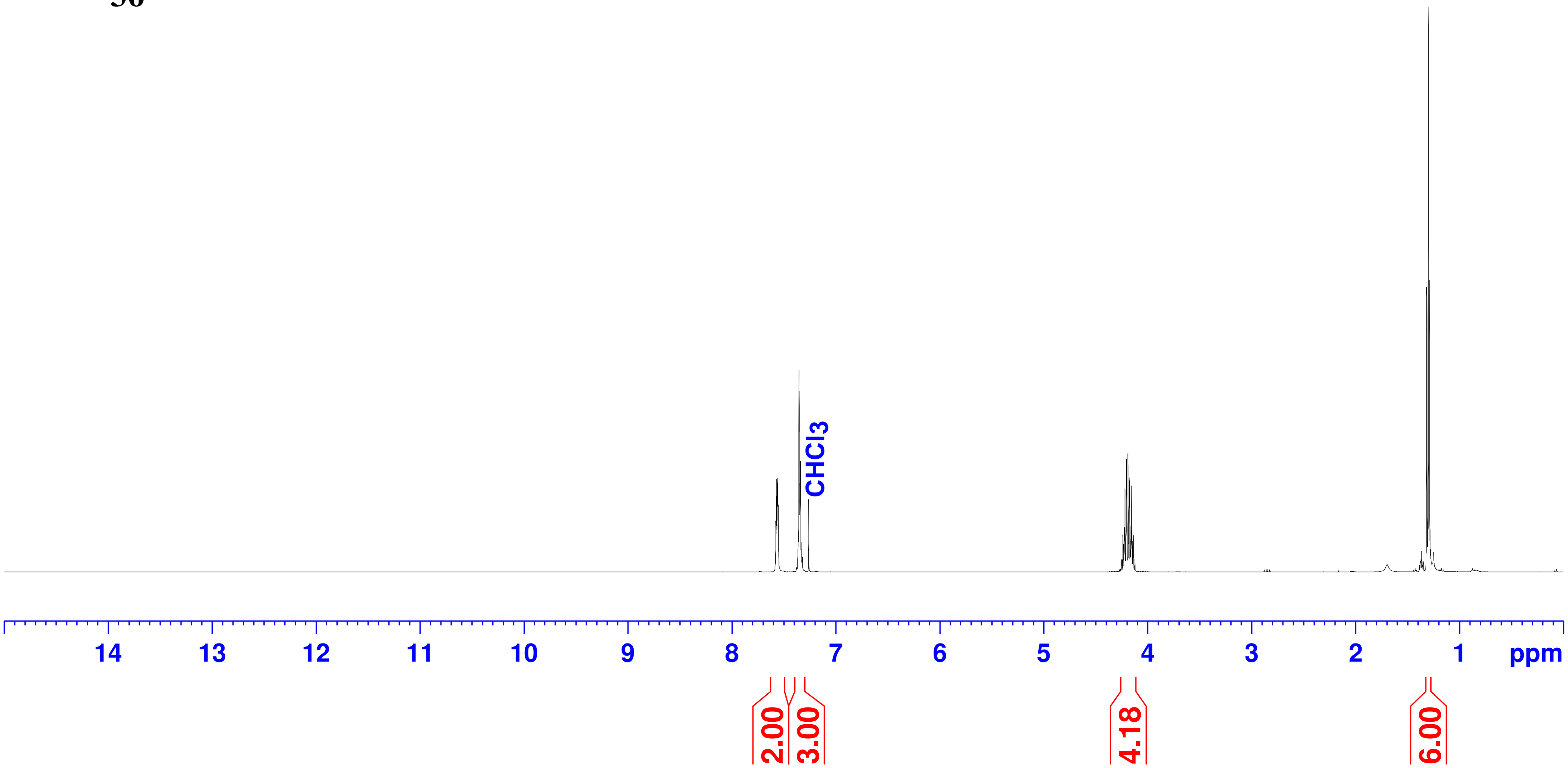

^{13}C NMR, 126 MHz, $CDCl_3$

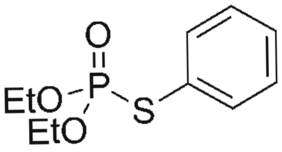

3o

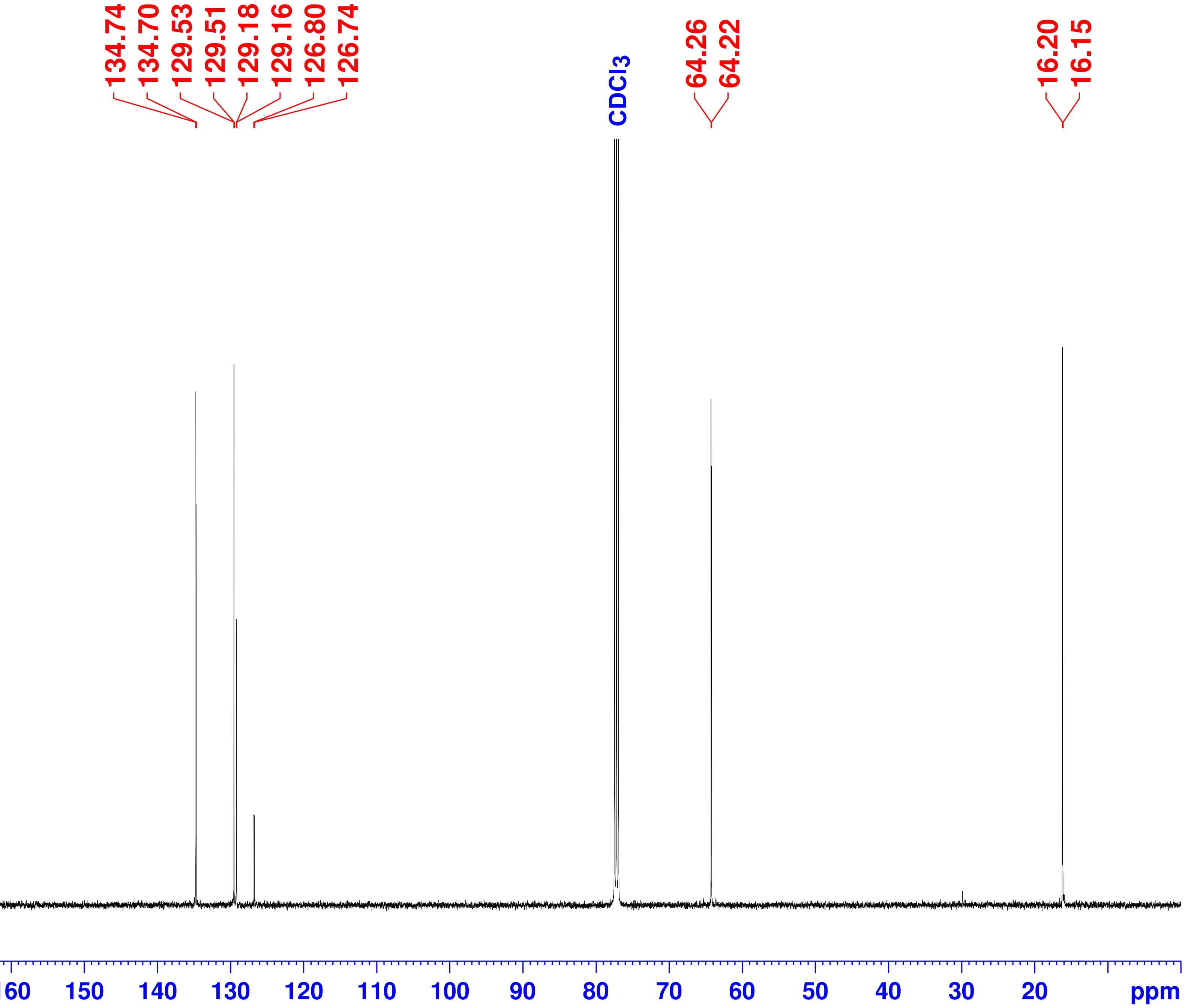

^{31}P NMR, 203 MHz, $CDCl_3$

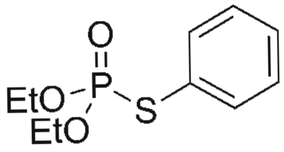

3o

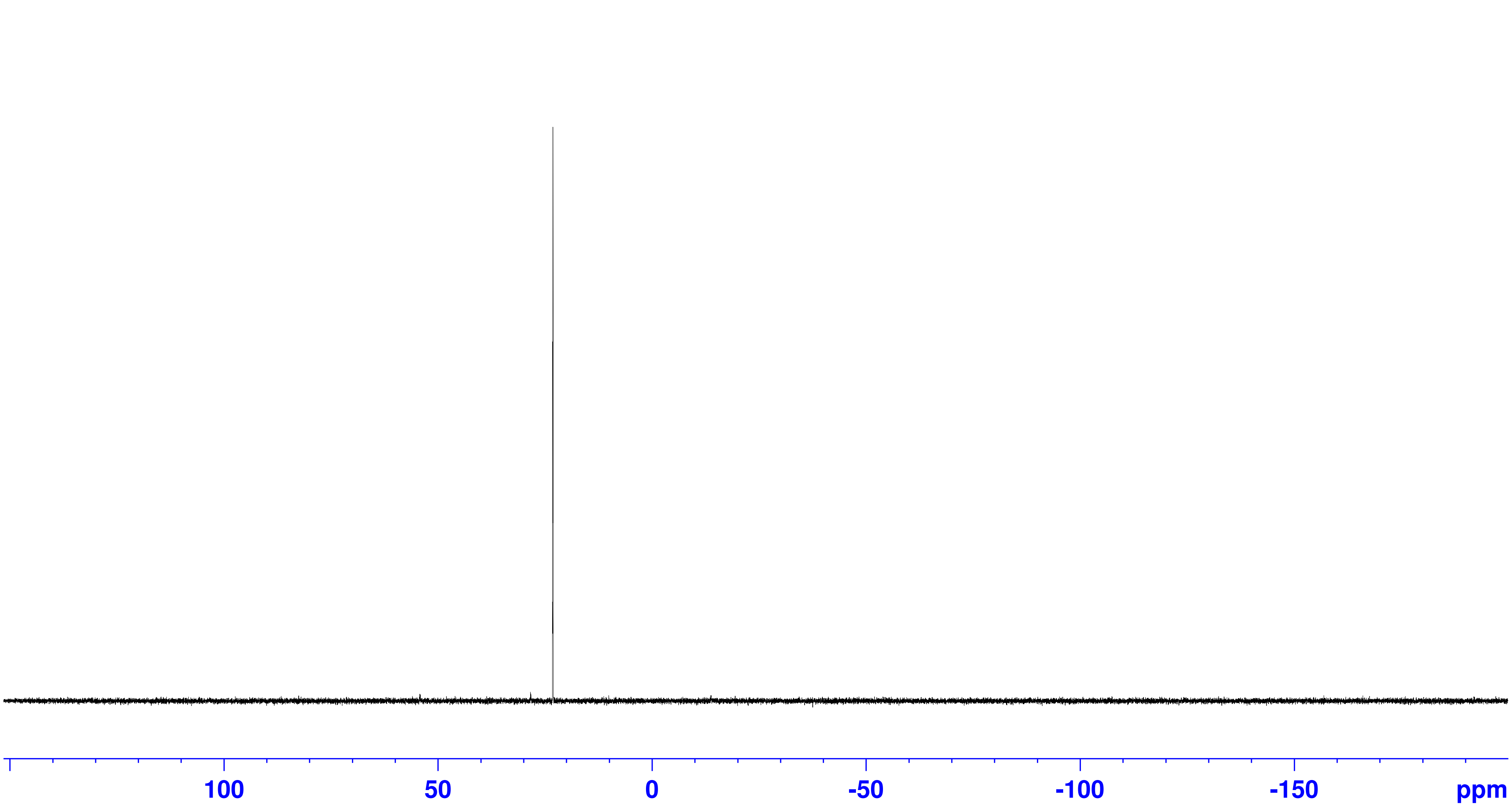

^{1}H NMR, 500 MHz, $CDCl_3$

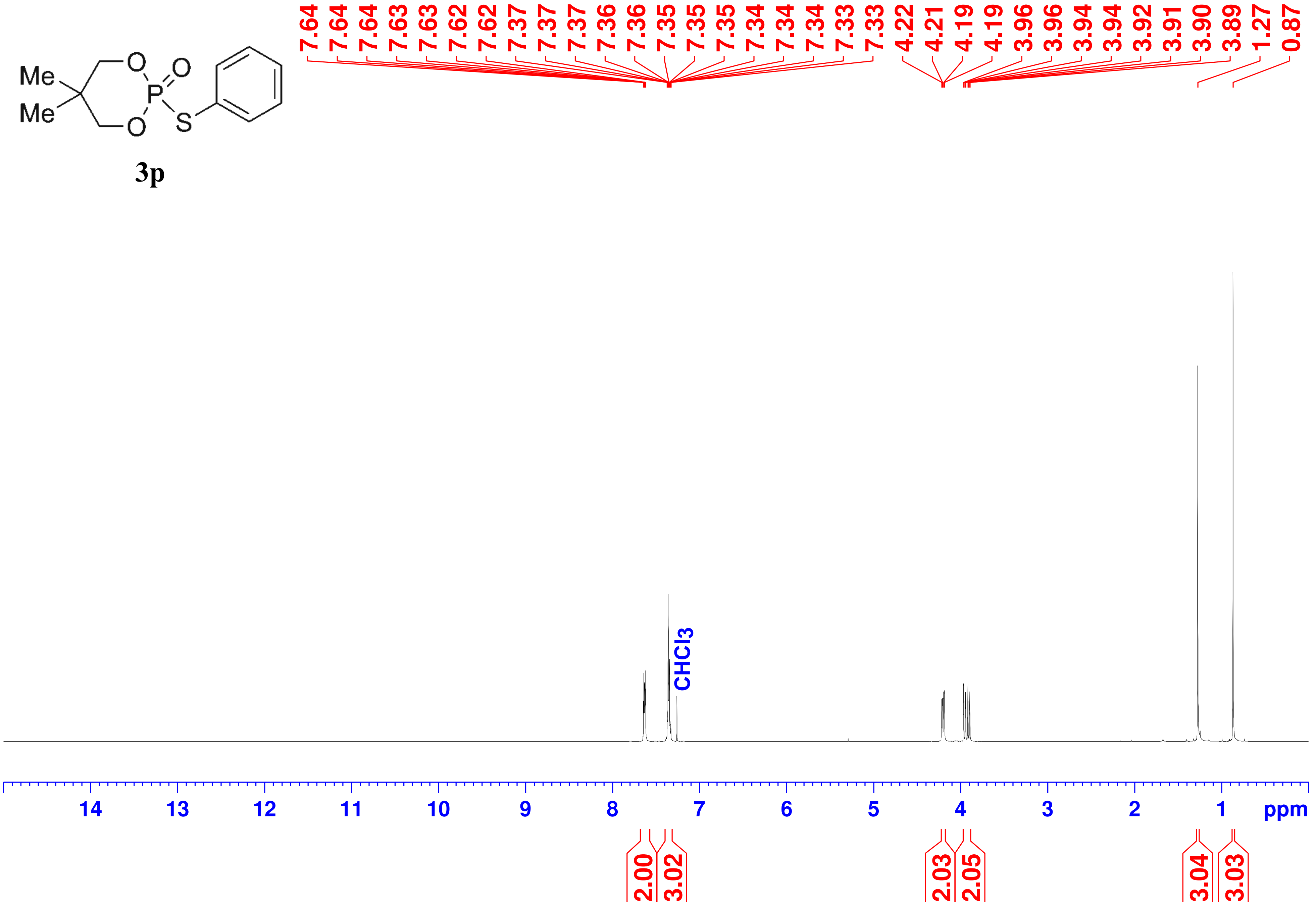

3p

^{13}C NMR, 126 MHz, $CDCl_3$

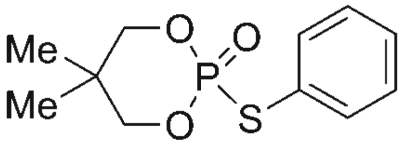

3p

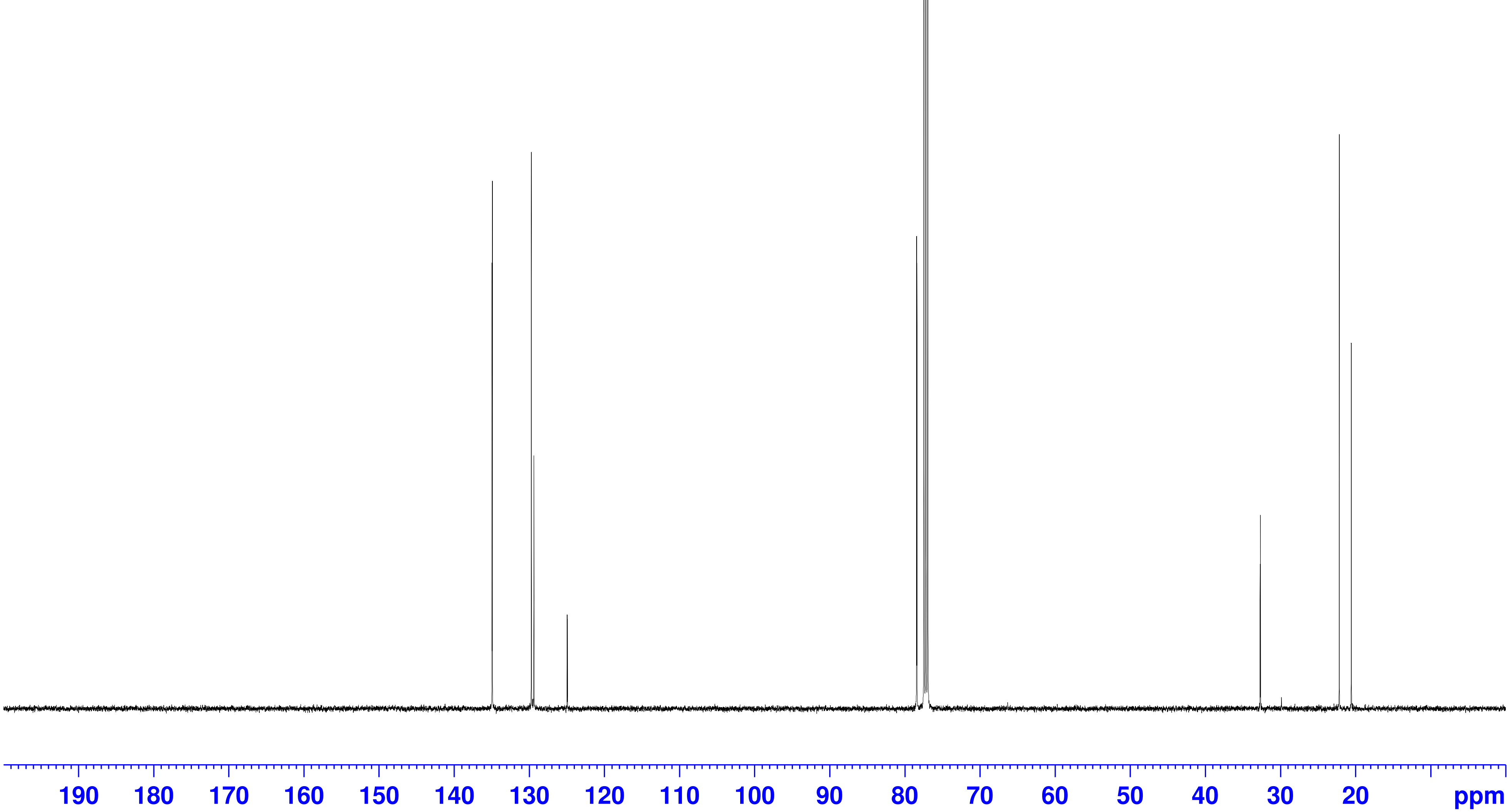

^{31}P NMR, 203 MHz, $CDCl_3$

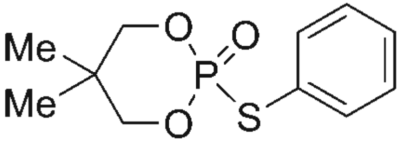

3p

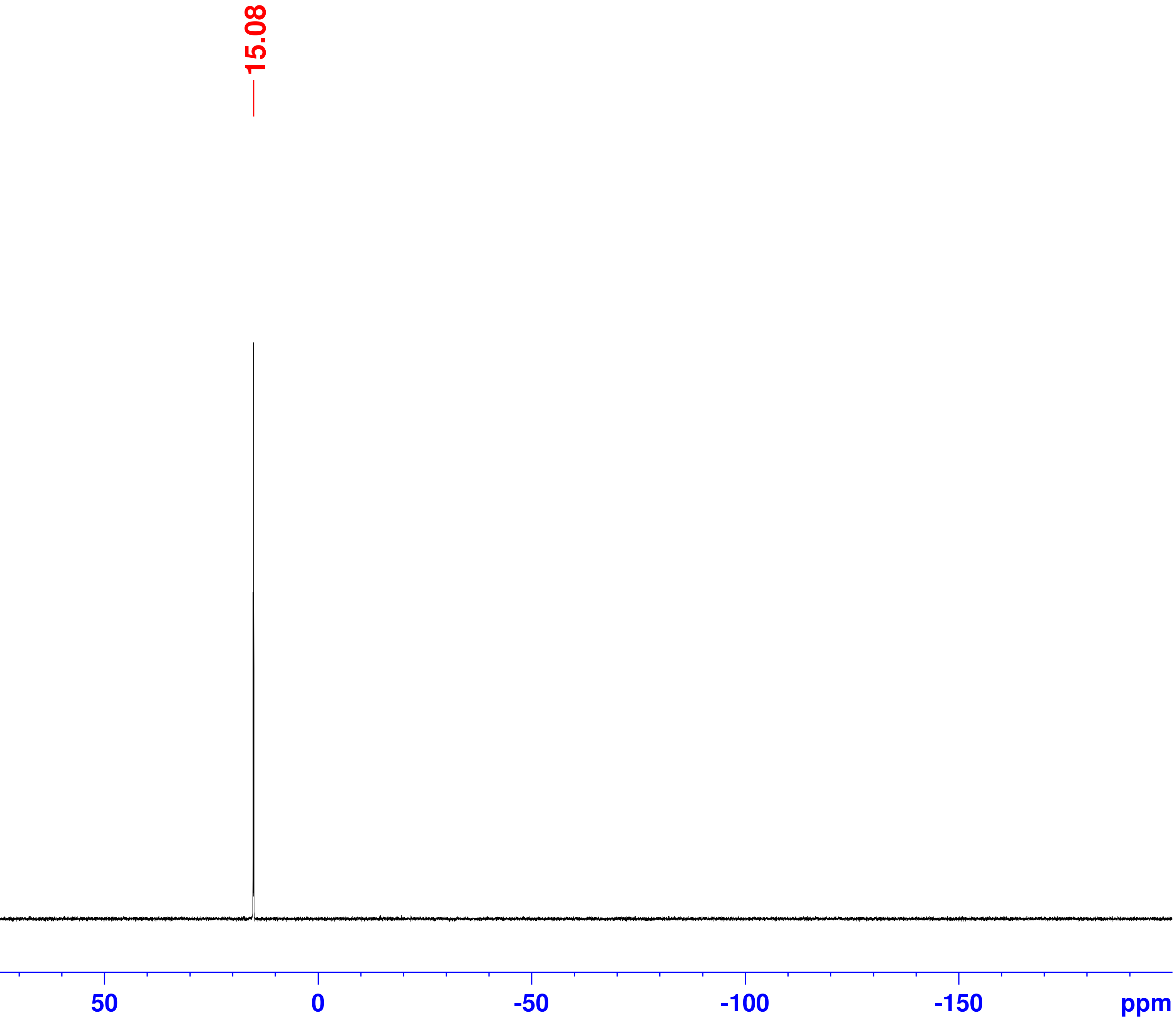

^{1}H NMR, 500 MHz, $CDCl_3$

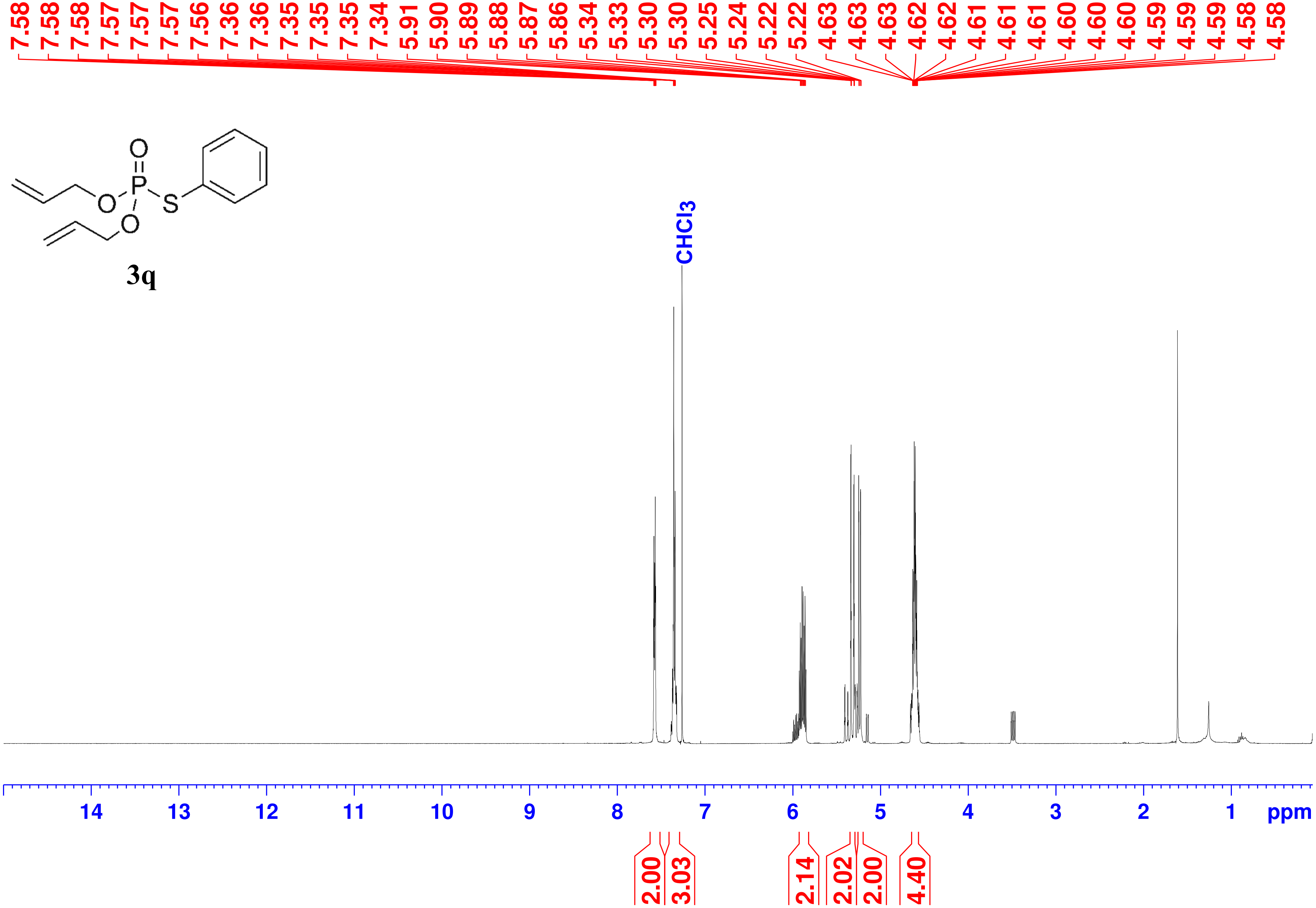

3q

^{13}C NMR, 126 MHz, $CDCl_3$

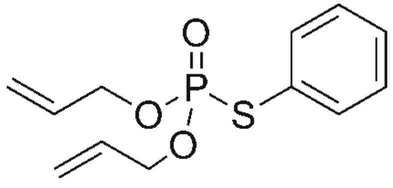

3q

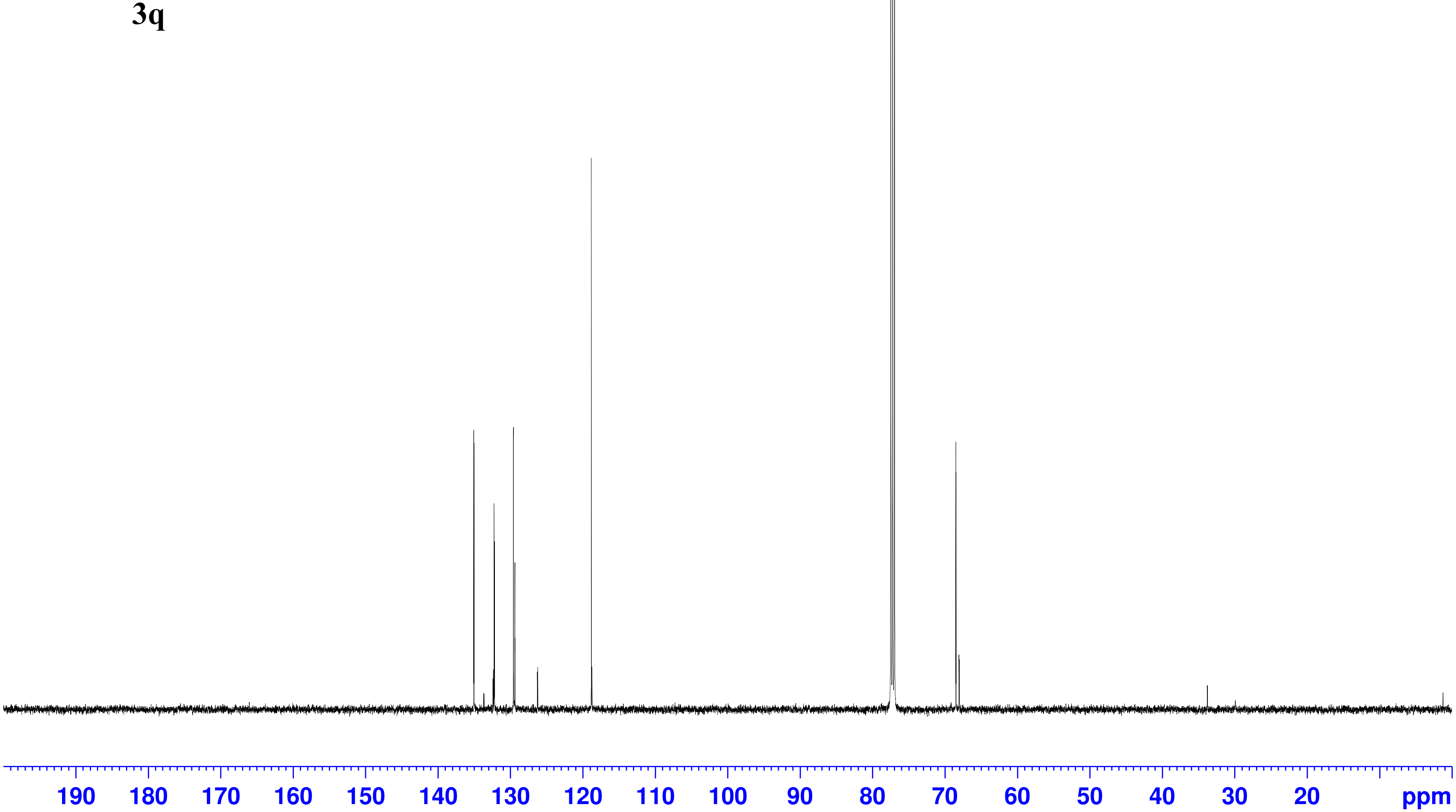

^{31}P NMR, 203 MHz, $CDCl_3$

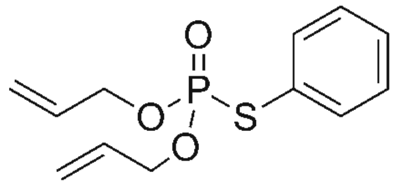

3q

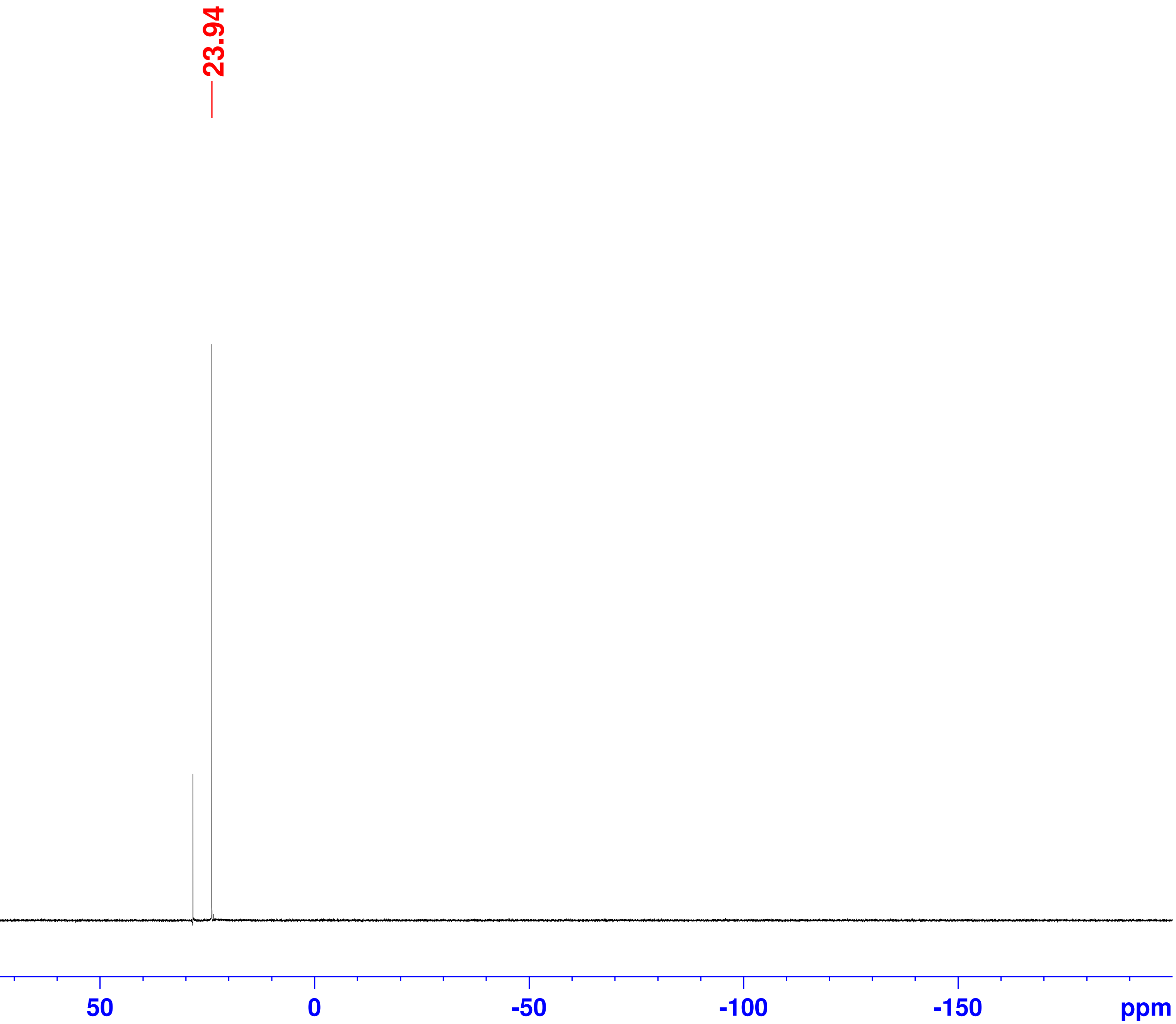

¹H NMR, 500 MHz, $CDCl_3$

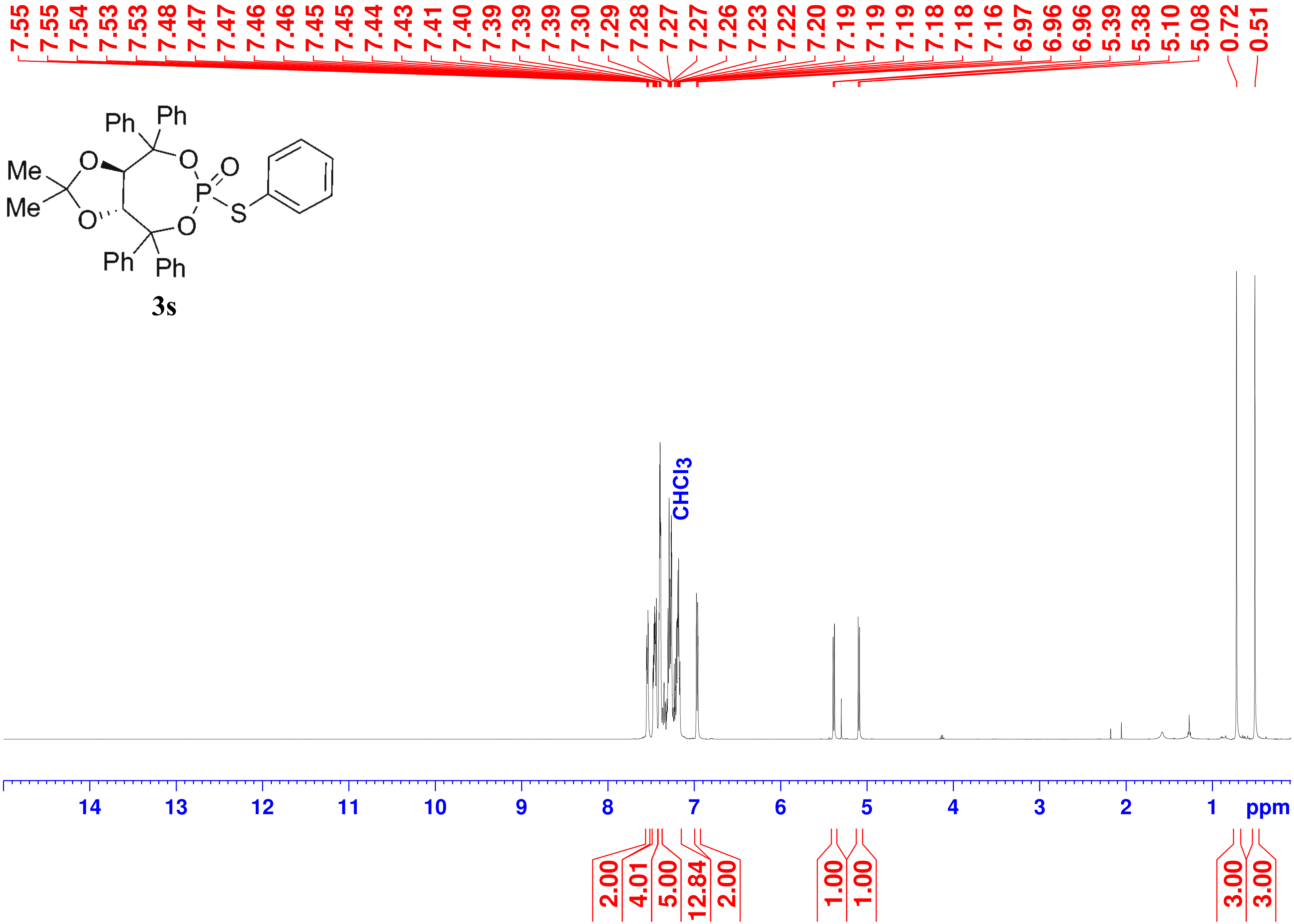

^{13}C NMR, 126 MHz, $CDCl_3$

3s

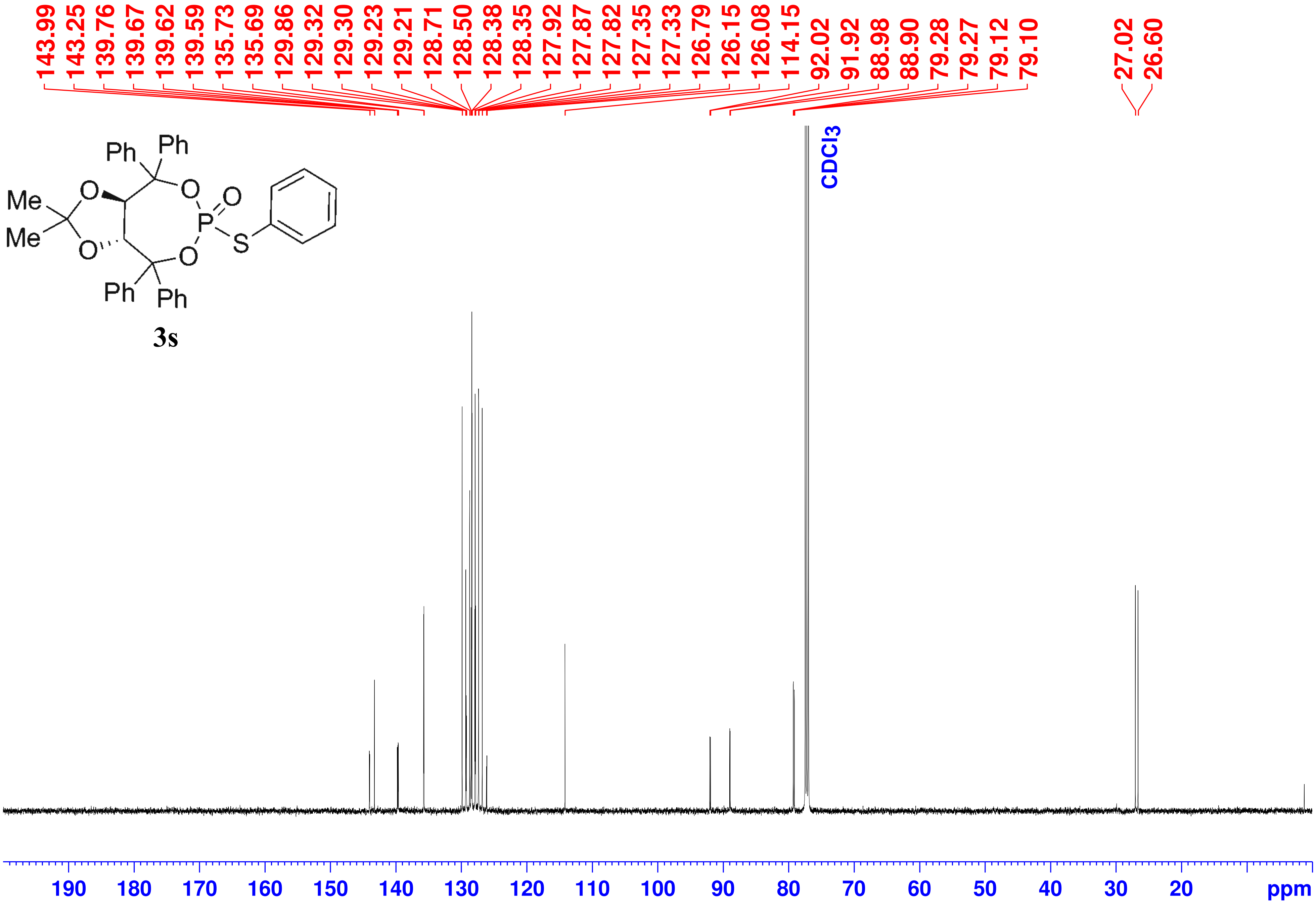

^{31}P NMR, 203 MHz, $CDCl_3$

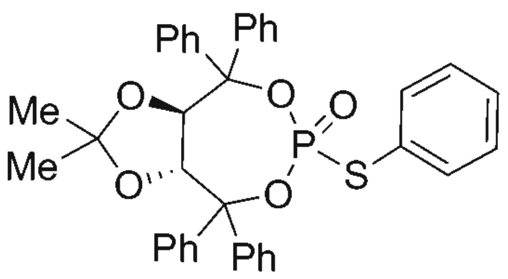

3s

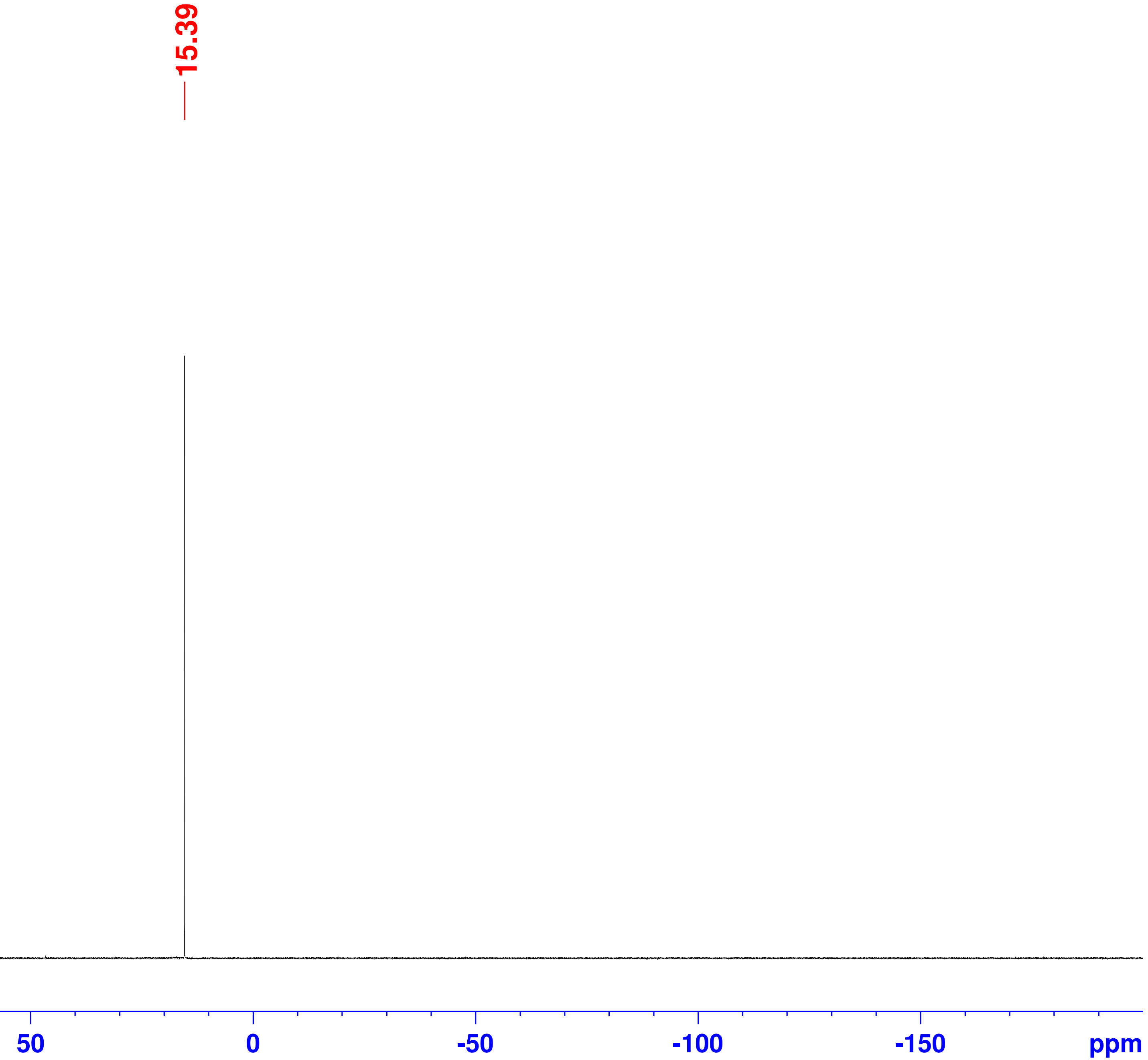

^{1}H NMR, 500 MHz, DMSO-d_6

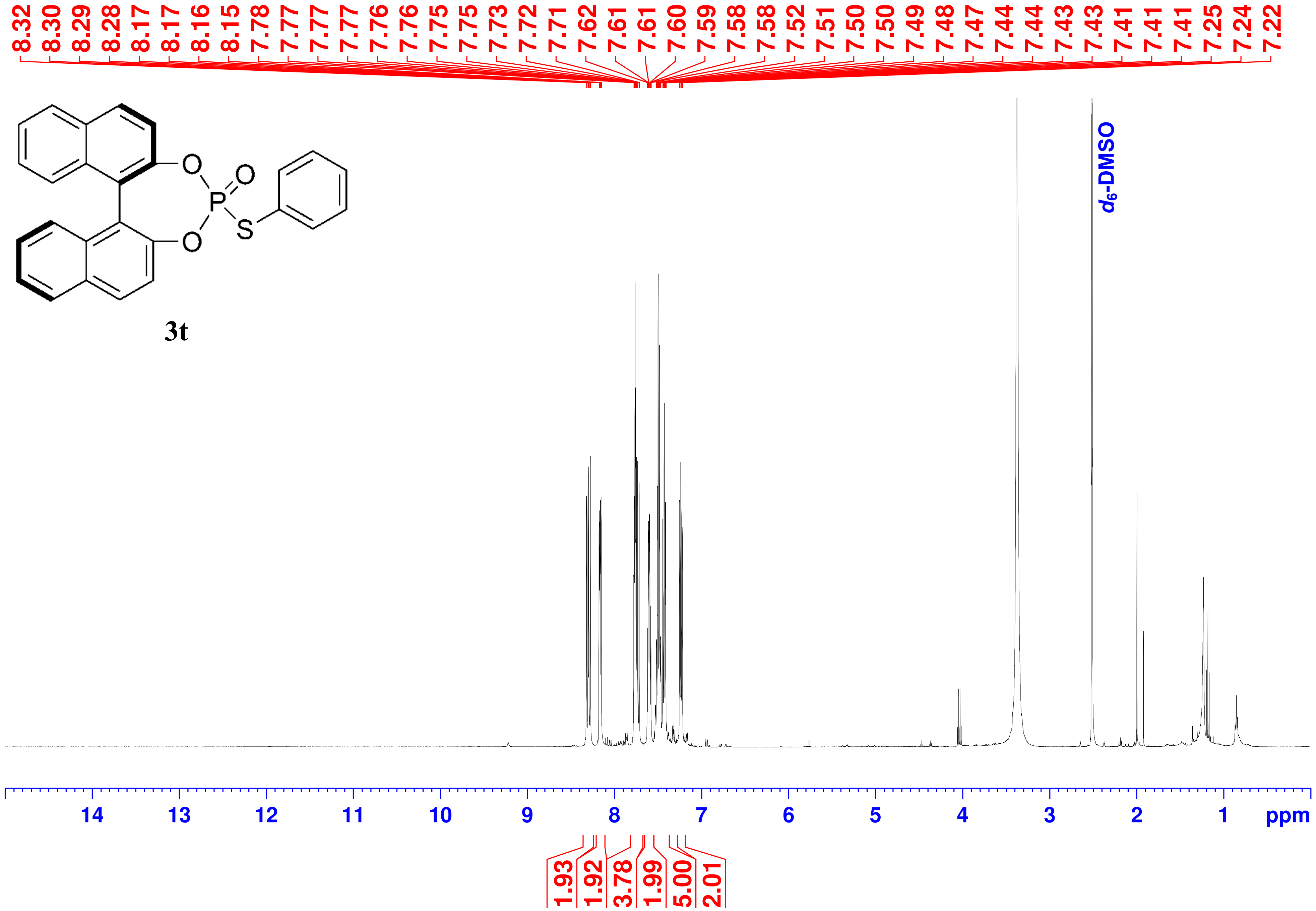

^{13}C NMR, 126 MHz, DMSO-d_6

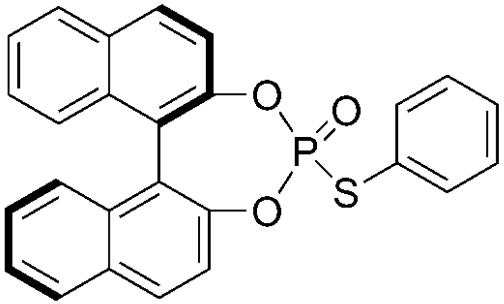

3t

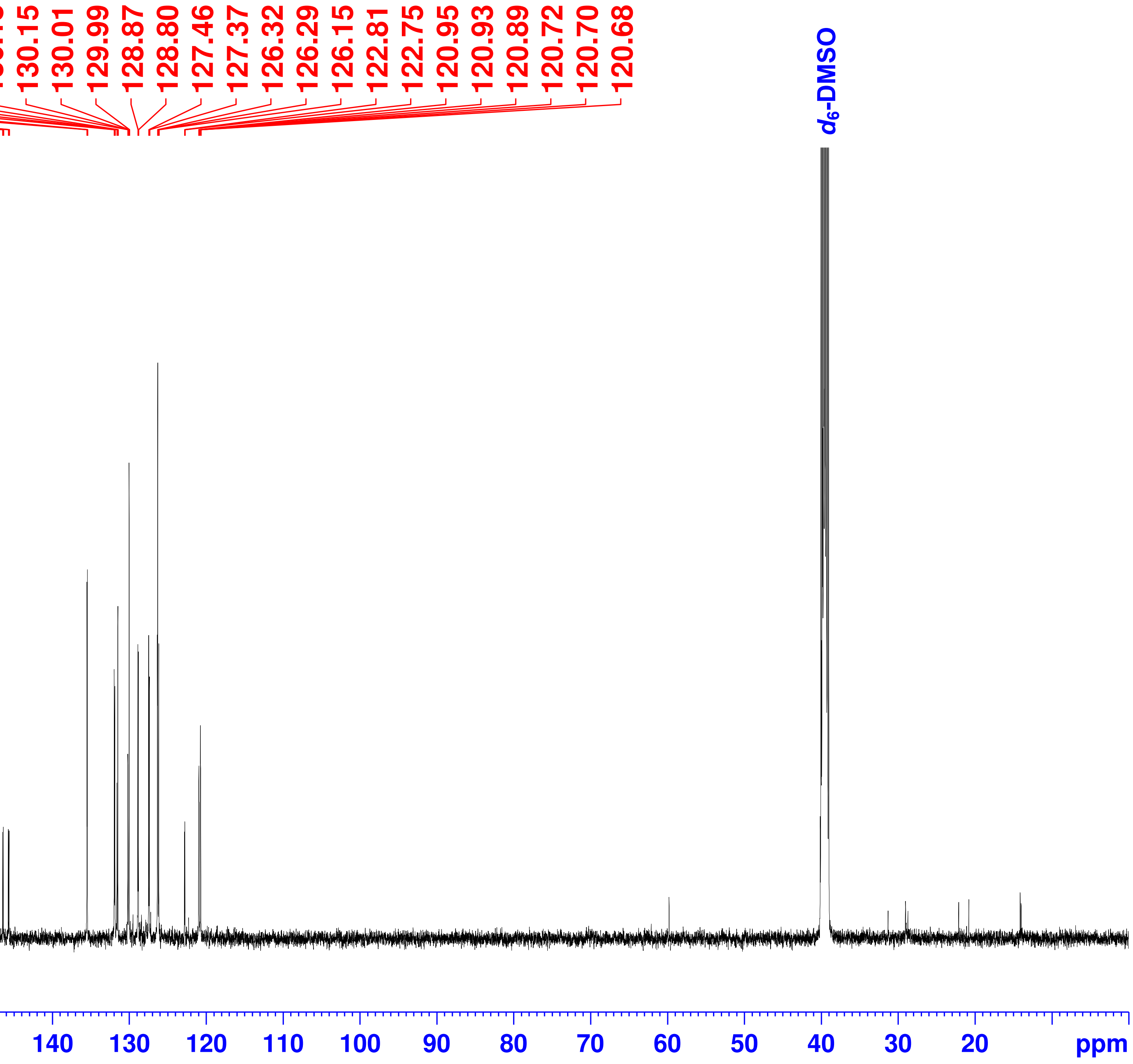

^{31}P NMR, 203 MHz, DMSO-d_6

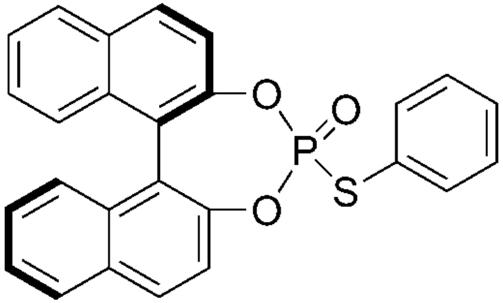

3t

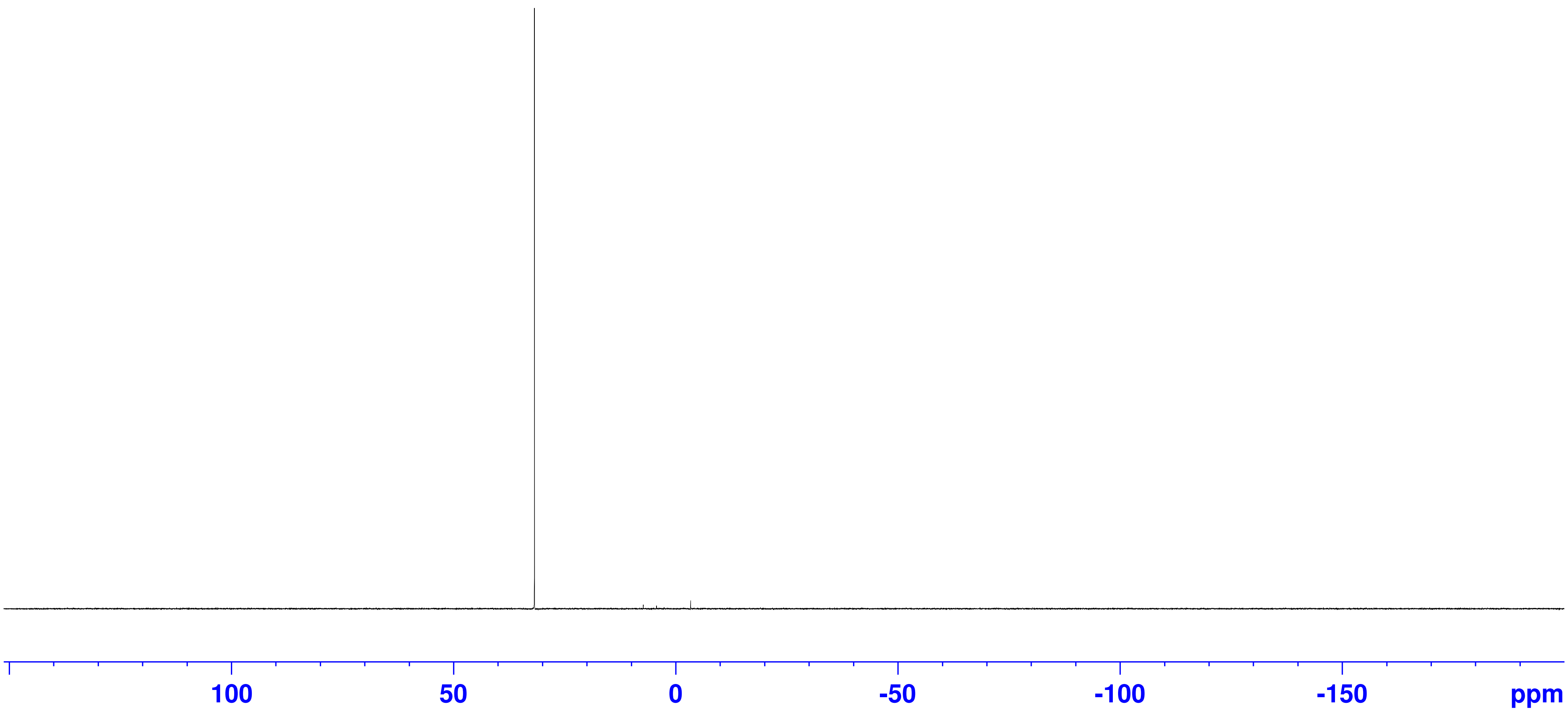

^{1}H NMR, 500 MHz, DMSO-d_6

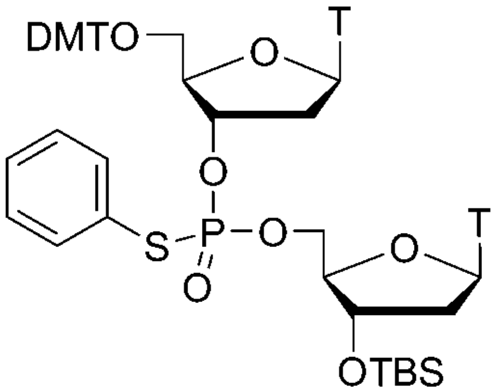

*R*p-3u

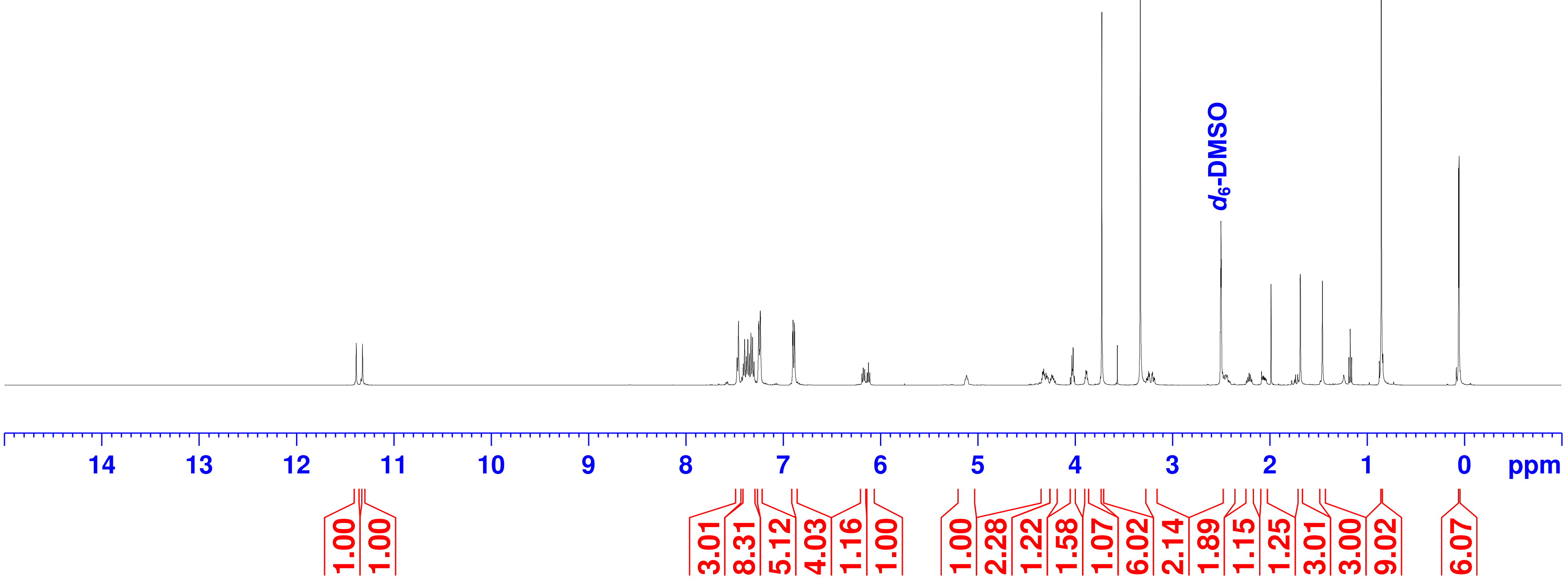

^{13}C NMR, 126 MHz, DMSO-d_6

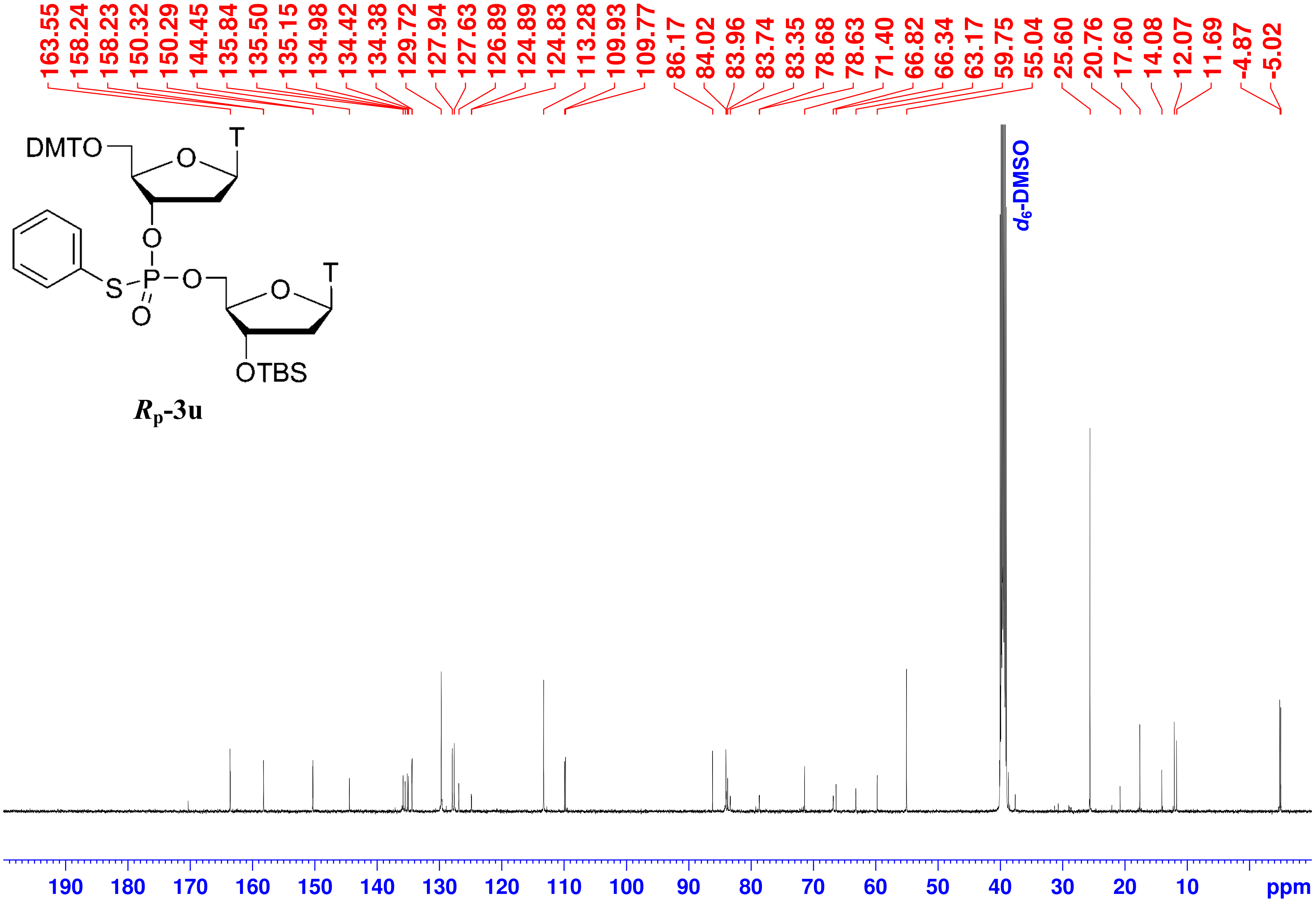

R_p-3u

^{31}P NMR, 203 MHz, DMSO-d_6

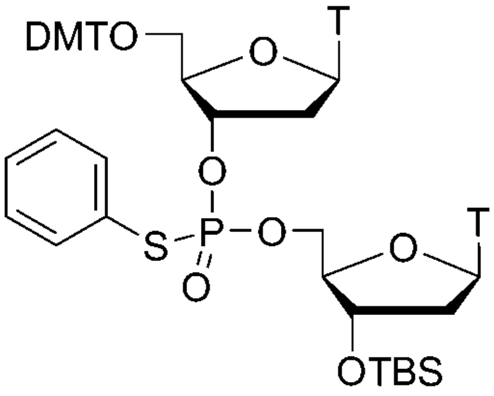

R$_p$-3u

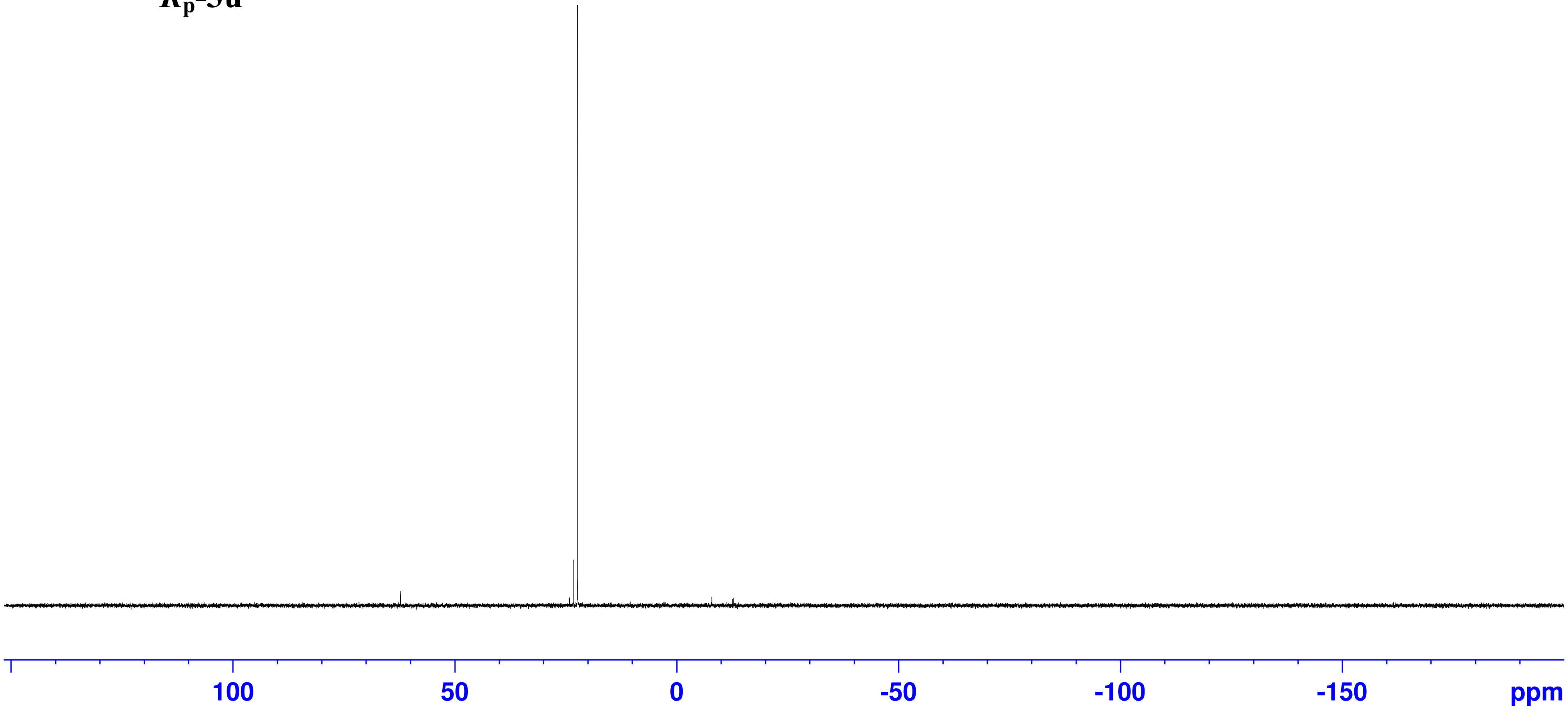

^{1}H NMR, 500 MHz, DMSO-d_6

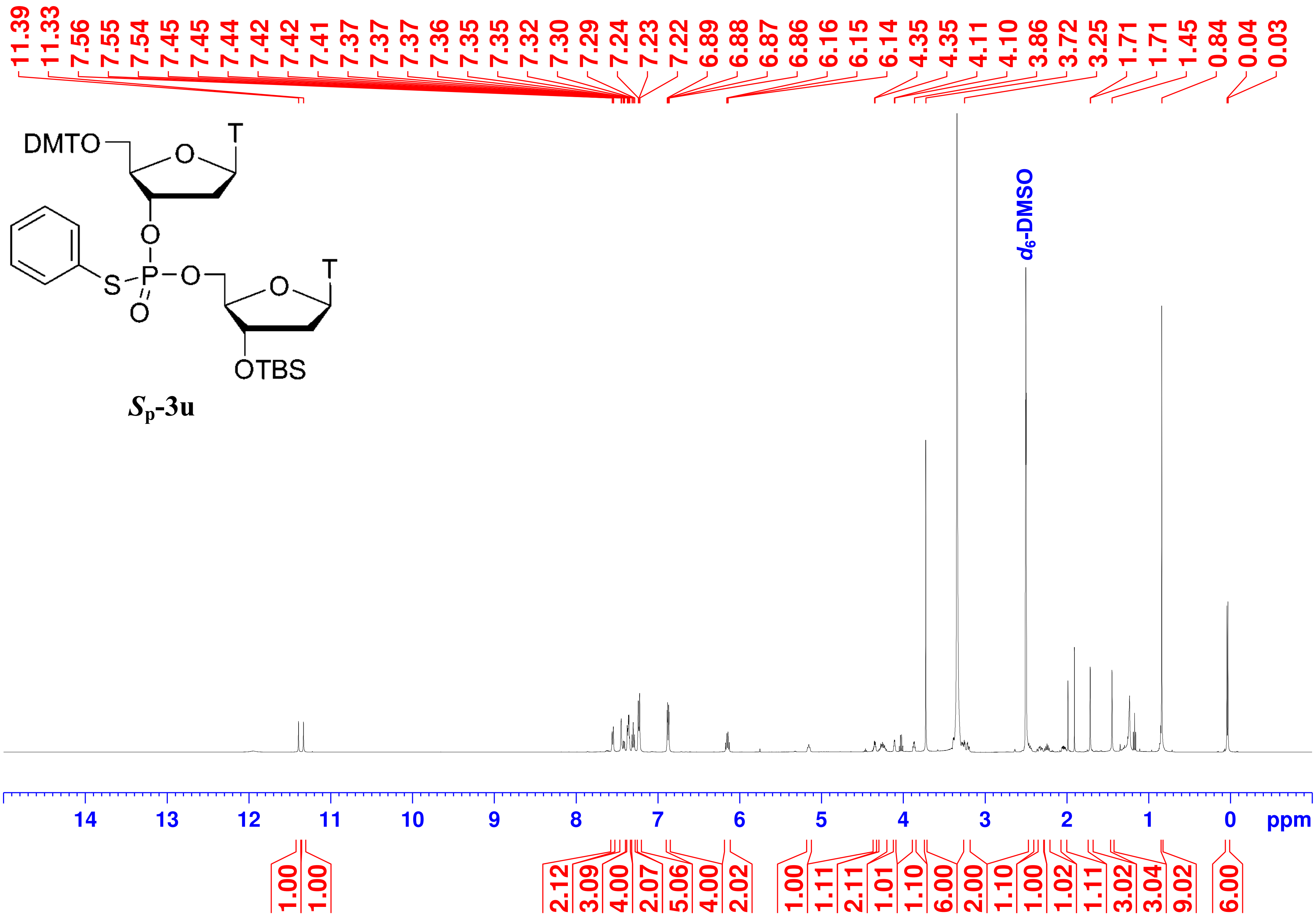

***S*$_p$-3u**

^{13}C NMR, 126 MHz, DMSO-d_6

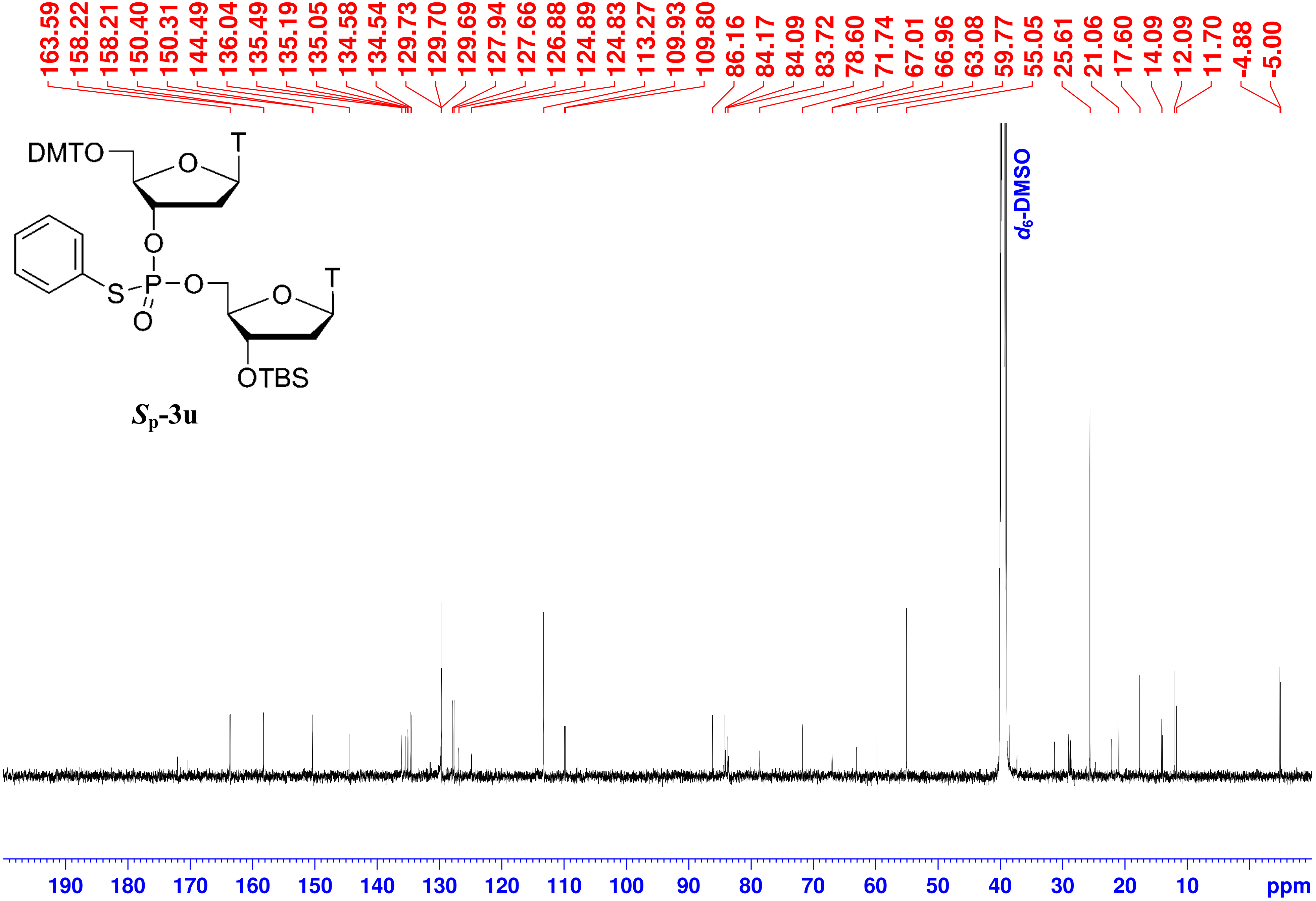

S_p-3u

^{31}P NMR, 203 MHz, DMSO-d_6

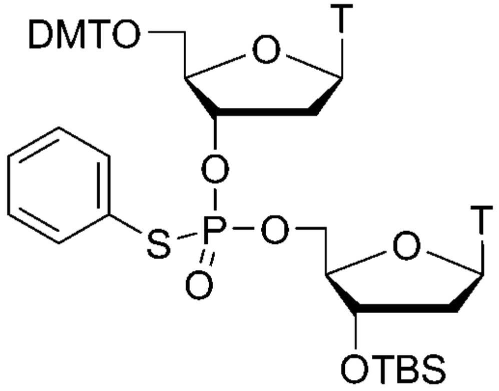

***S*p-3u**

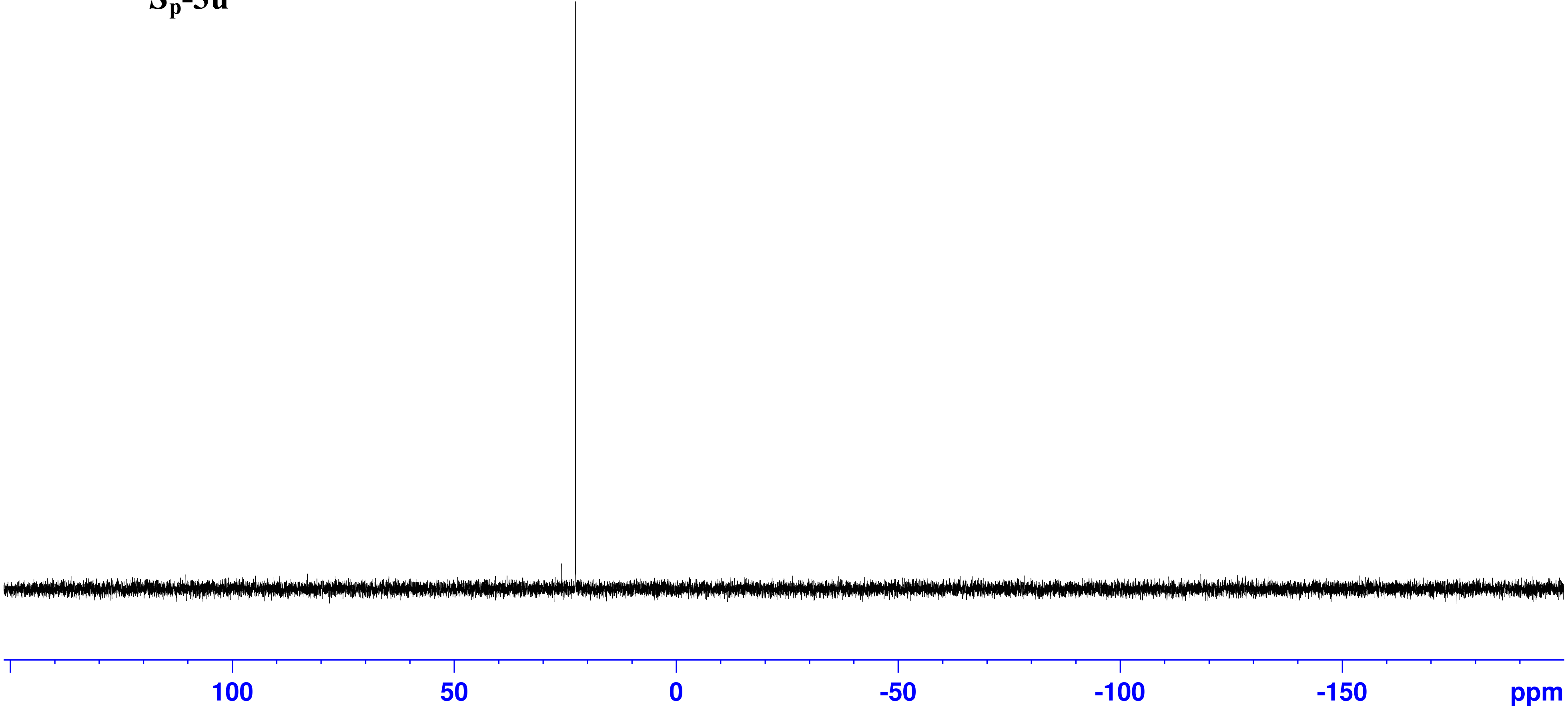

^{1}H NMR, 500 MHz, $CDCl_3$

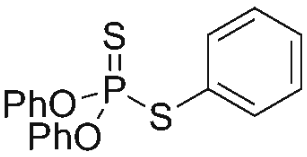

4a

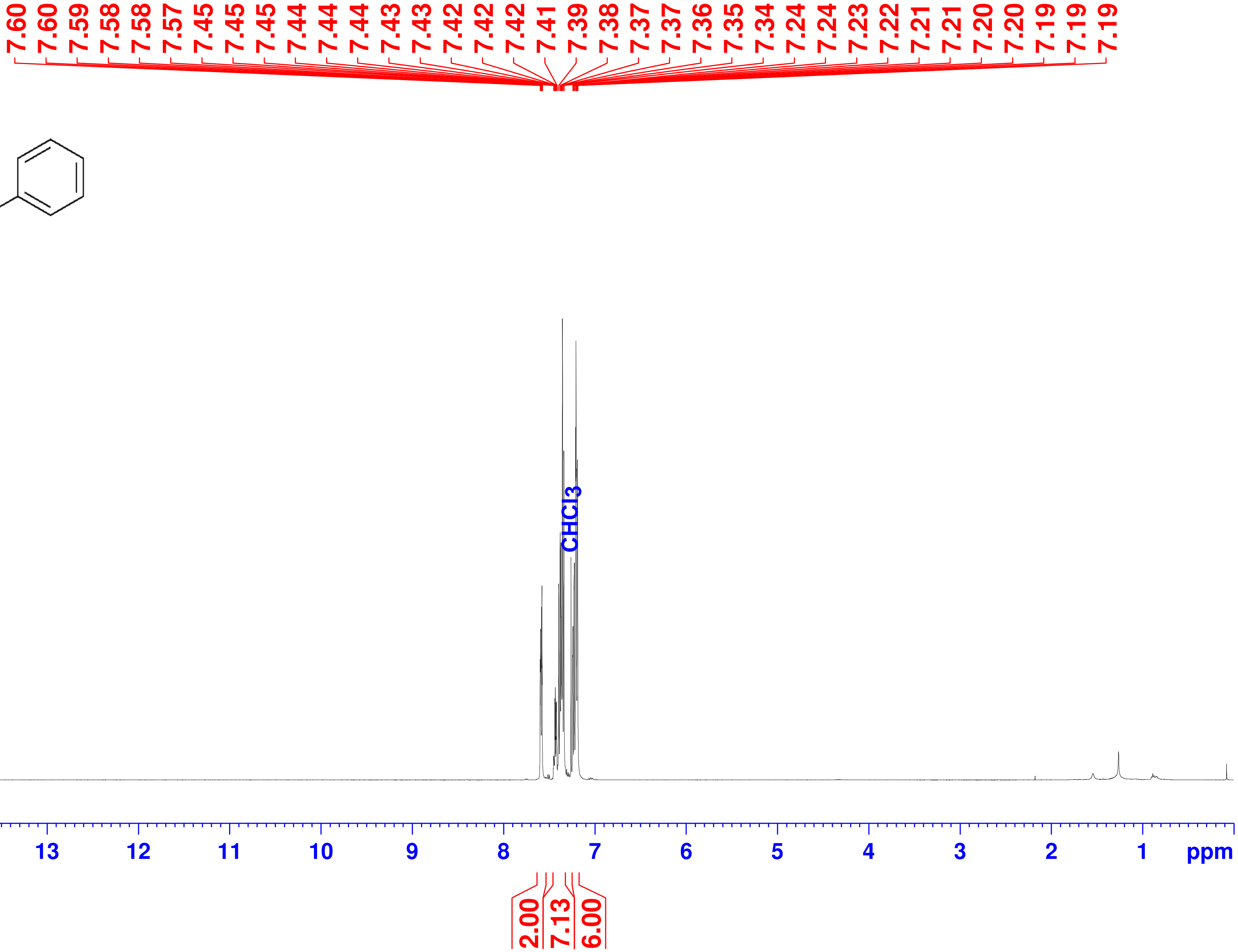

^{13}C NMR, 126 MHz, $CDCl_3$

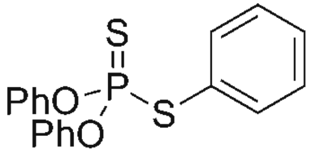

4a

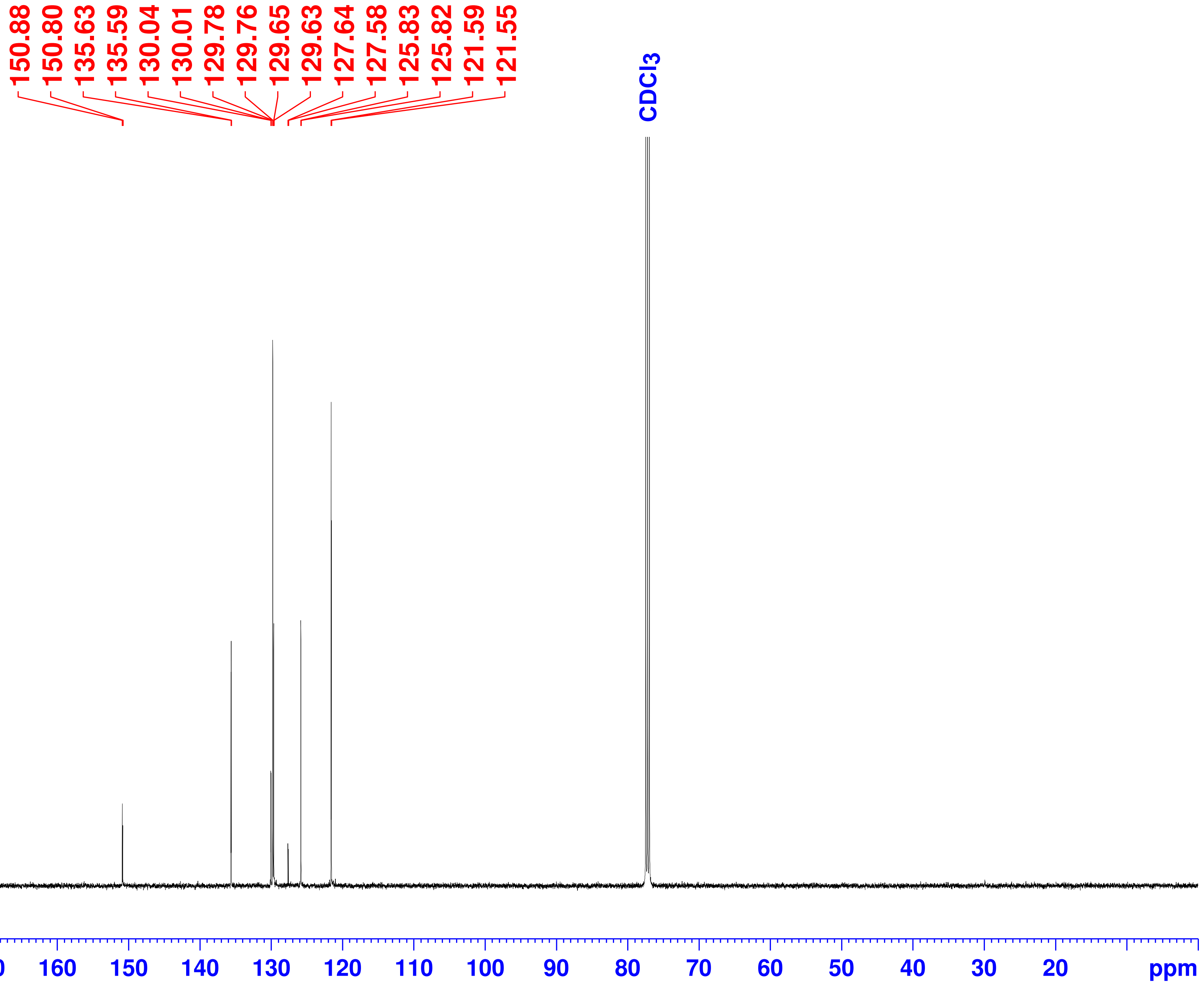

^{31}P NMR, 203 MHz, $CDCl_3$

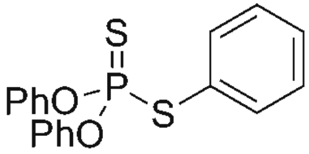

4a

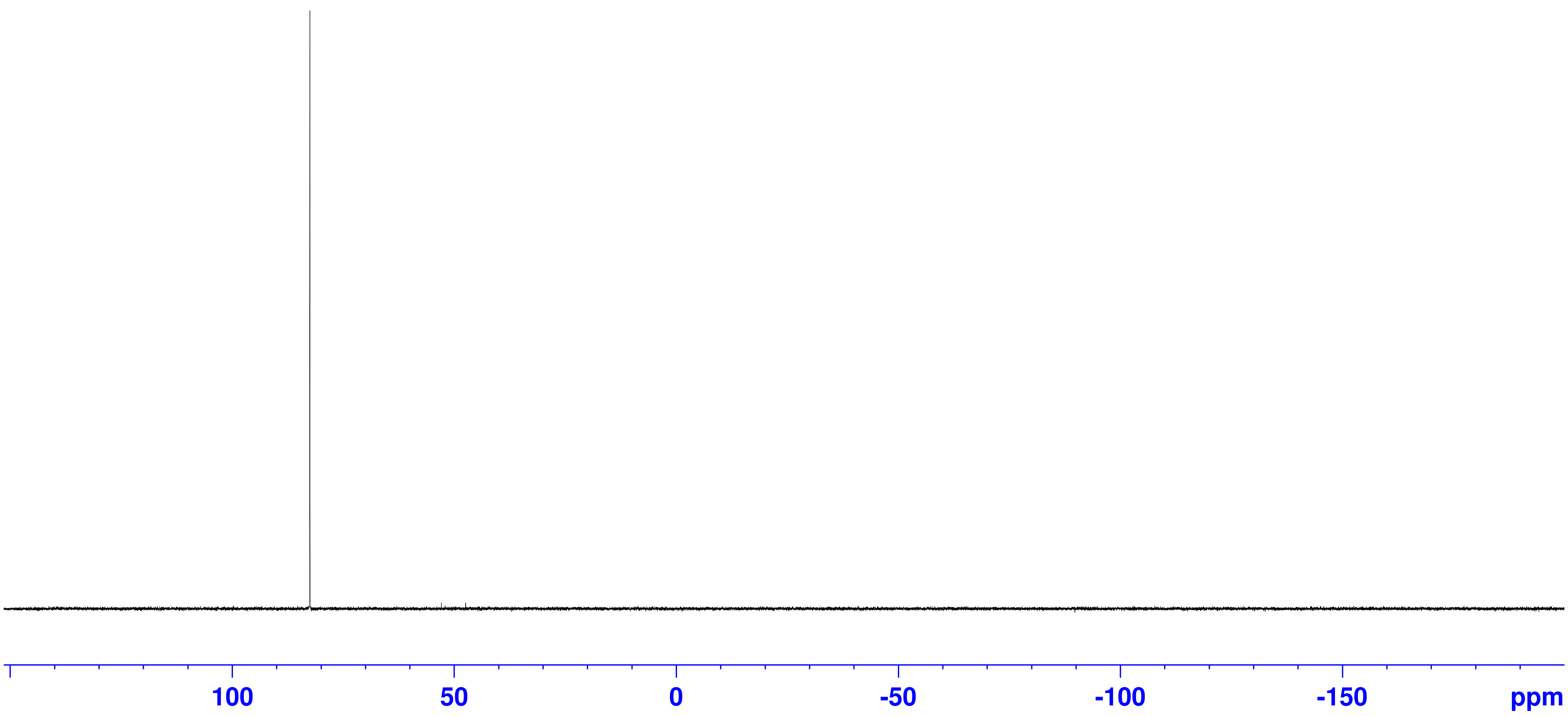

^1H NMR, 500 MHz, $CDCl_3$

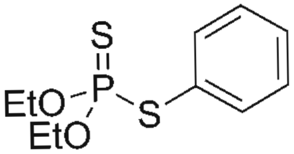

4b

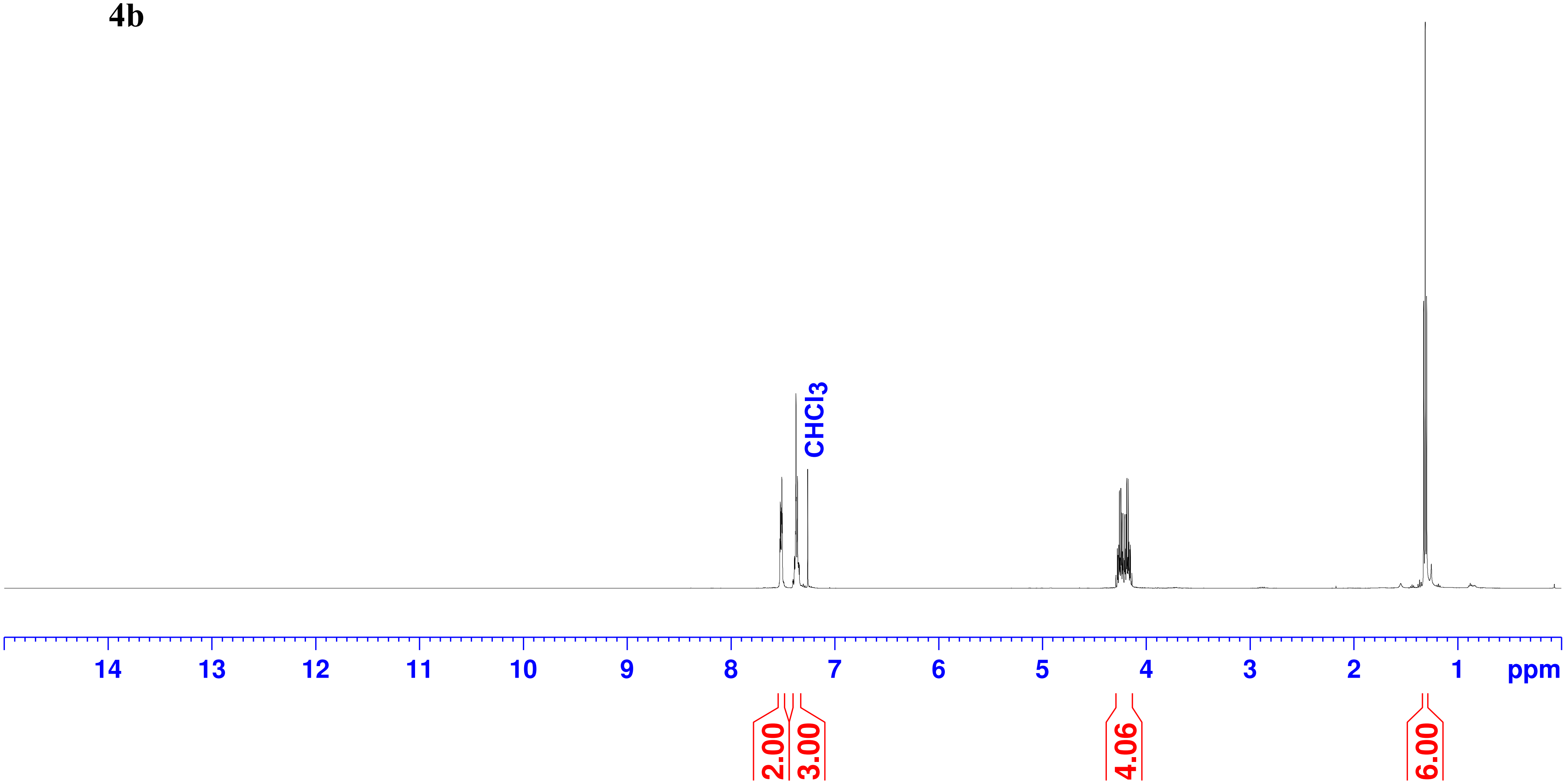

^{13}C NMR, 126 MHz, $CDCl_3$

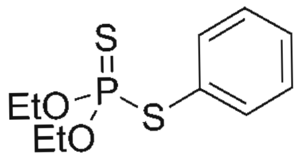

4b

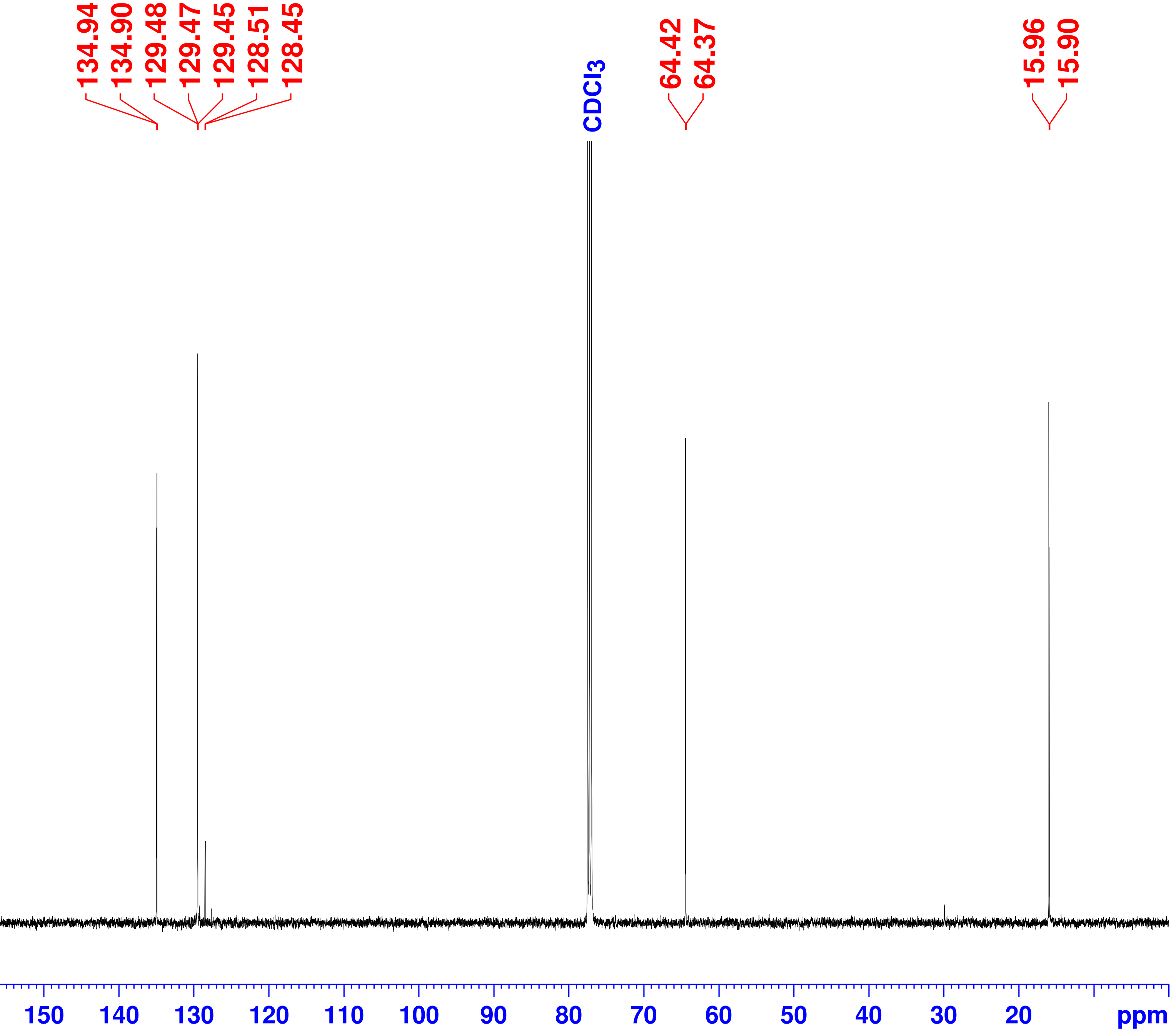

^{31}P NMR, 203 MHz, $CDCl_3$

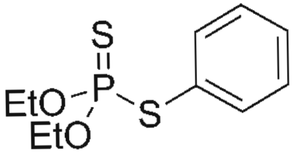

4b

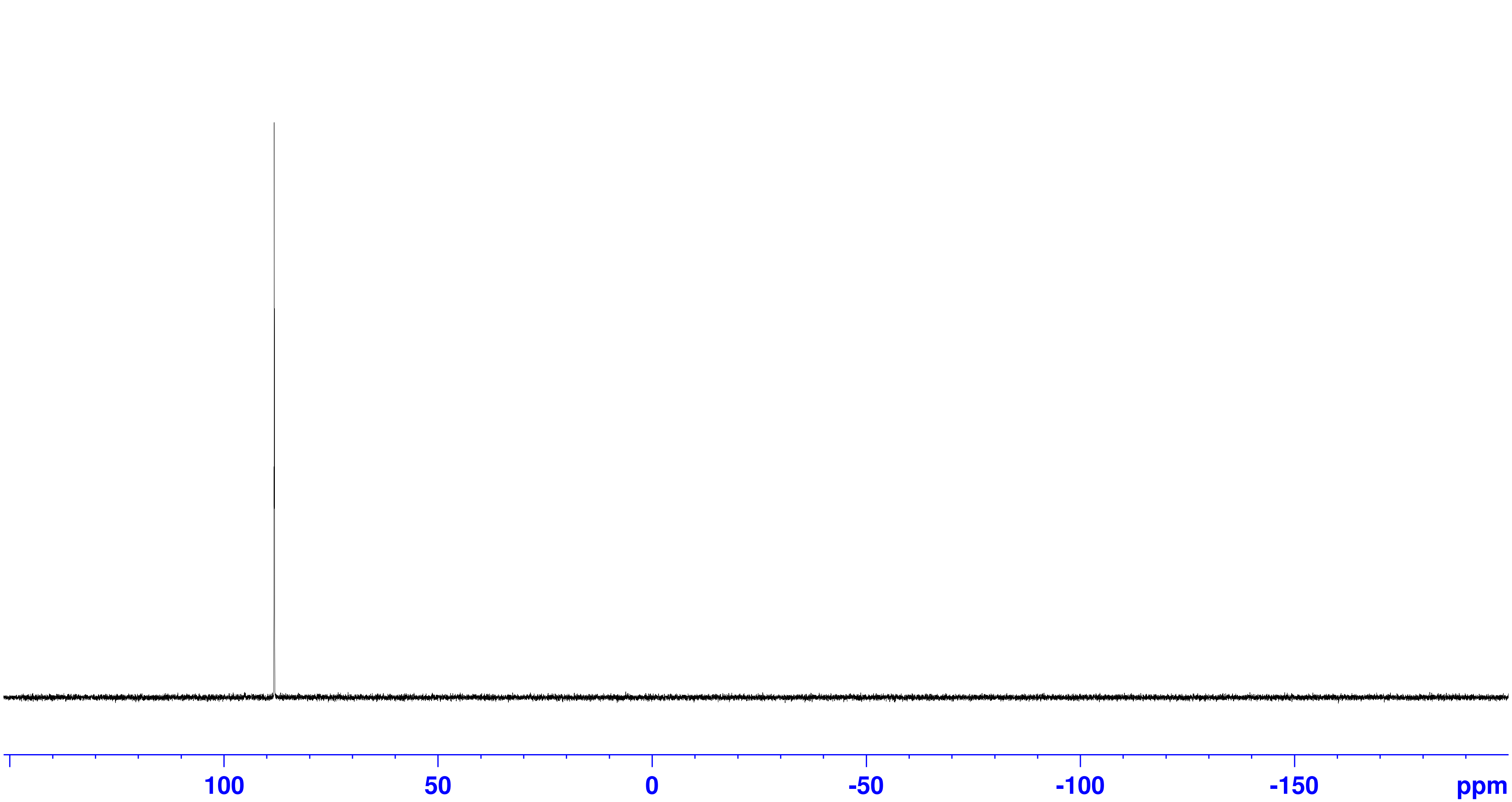

^{1}H NMR, 500 MHz, $CDCl_3$

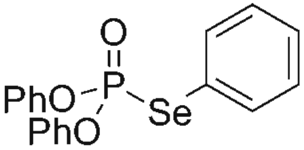

5a

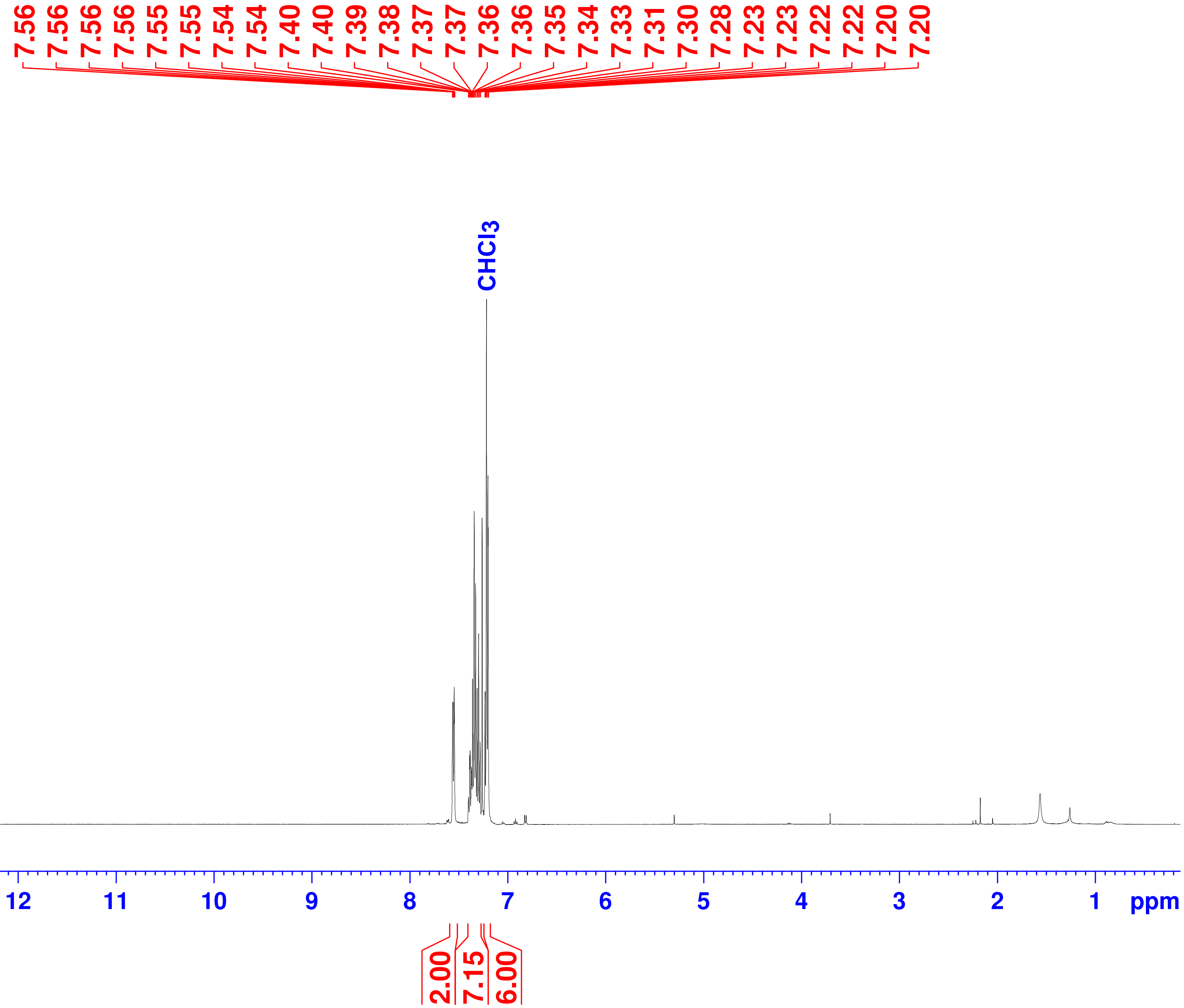

^{13}C NMR, 126 MHz, $CDCl_3$

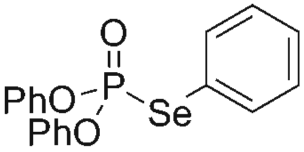

5a

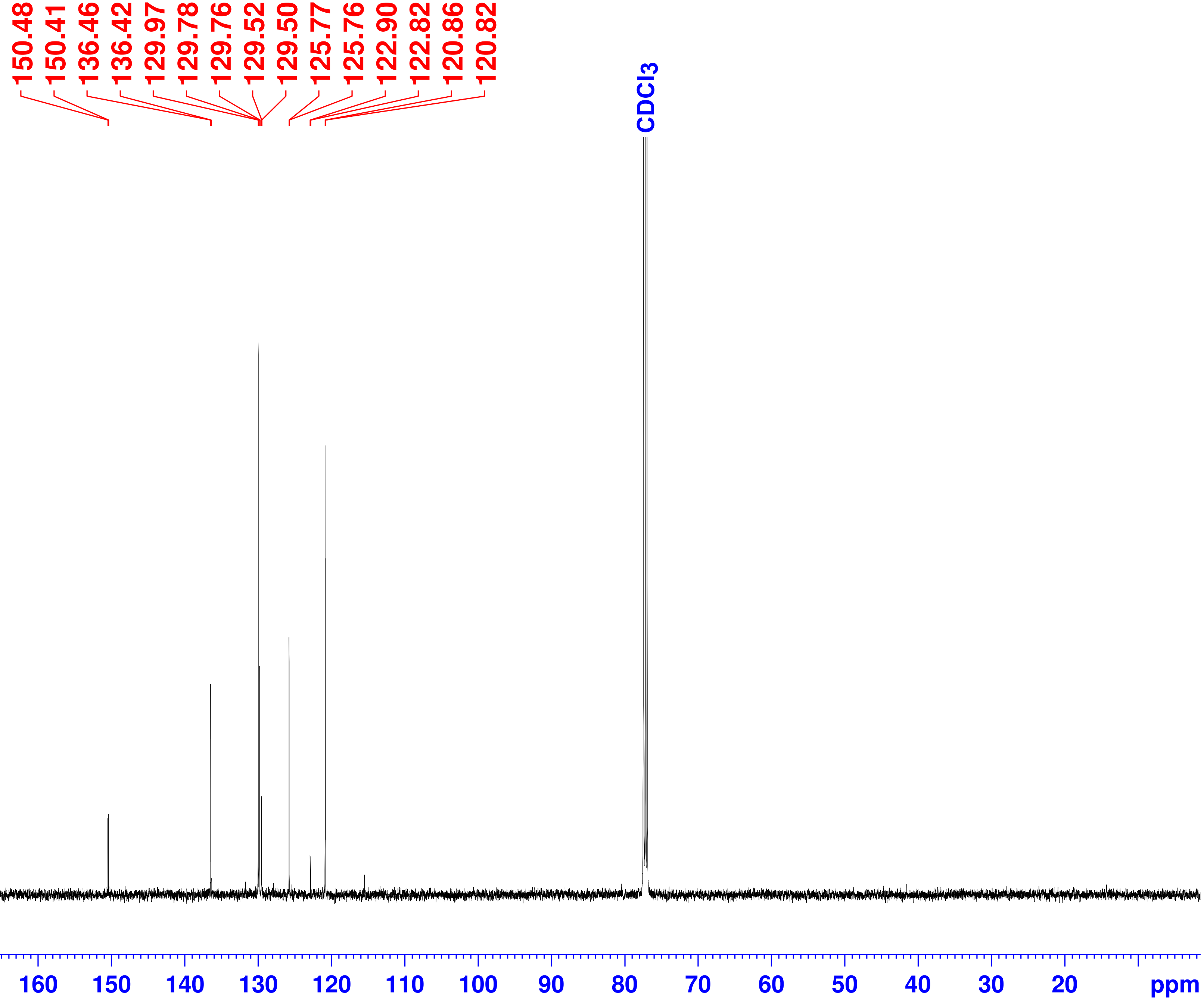

^{31}P NMR, 203 MHz, $CDCl_3$

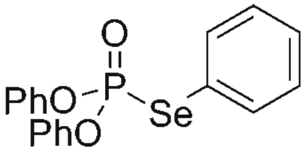

5a

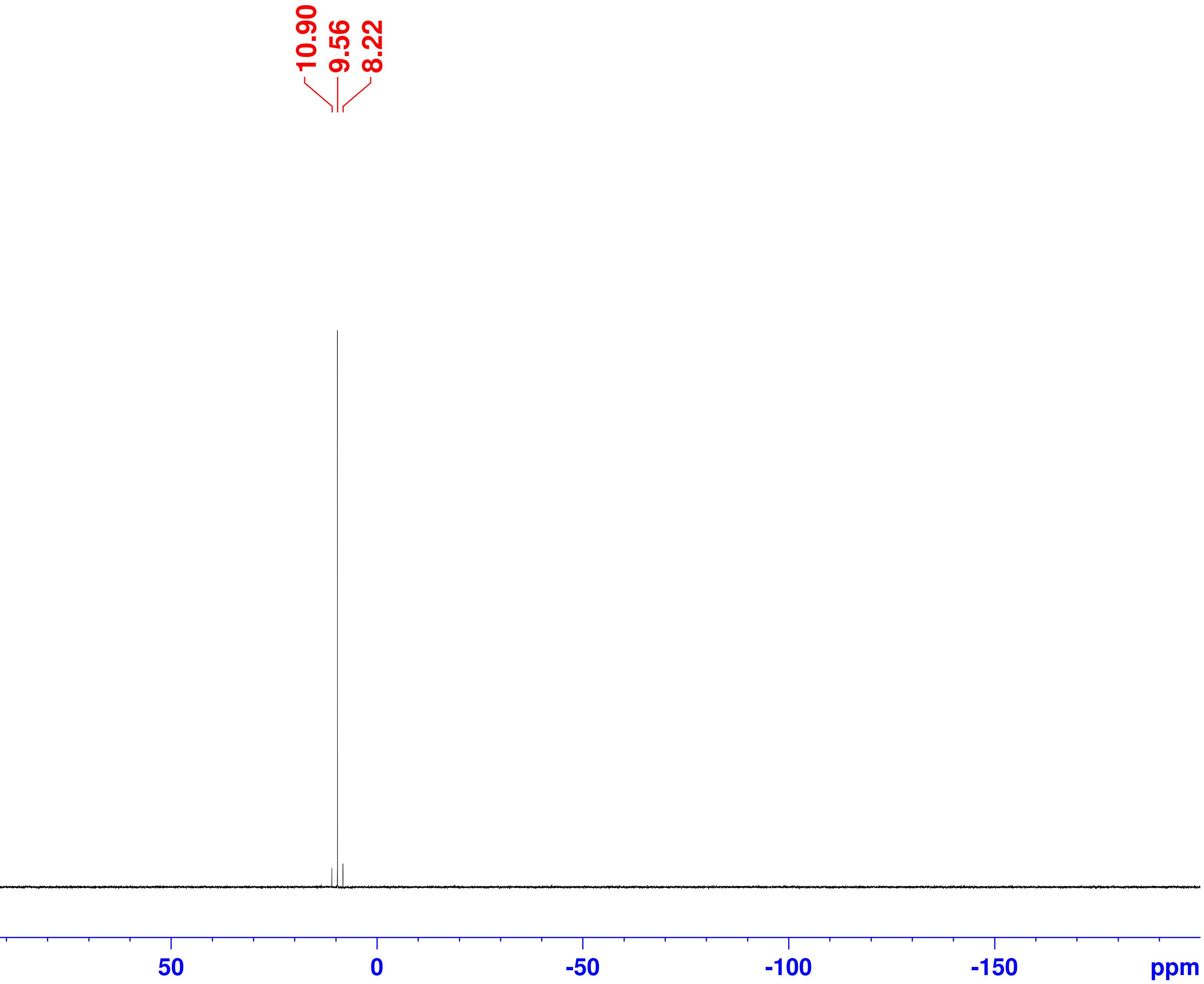

^{1}H NMR, 500 MHz, $CDCl_3$

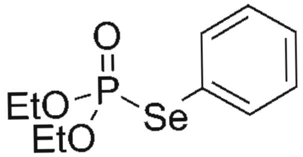

5b

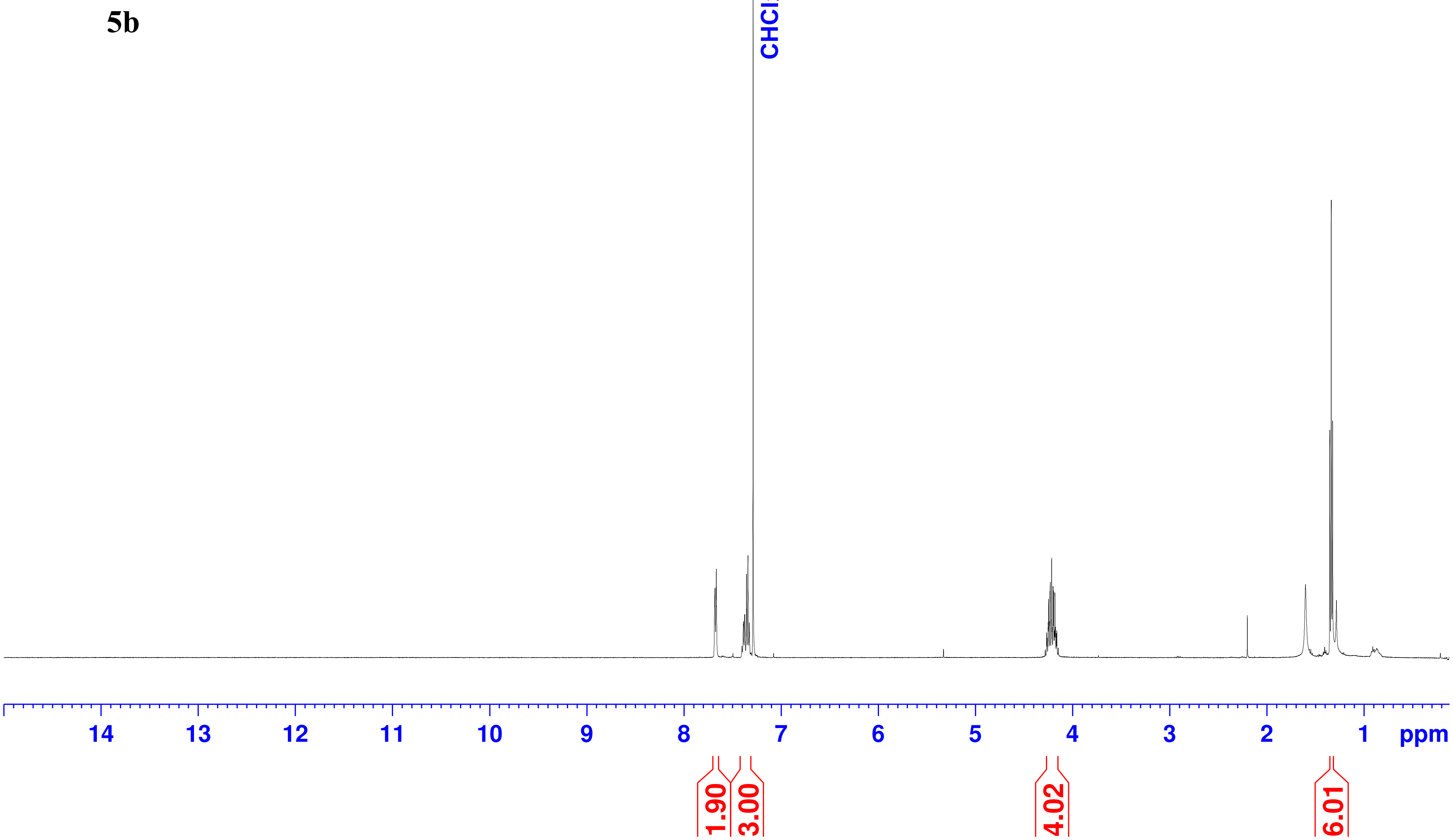

^{13}C NMR, 126 MHz, $CDCl_3$

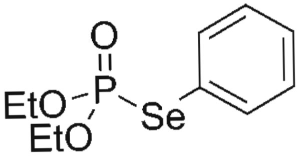

5b

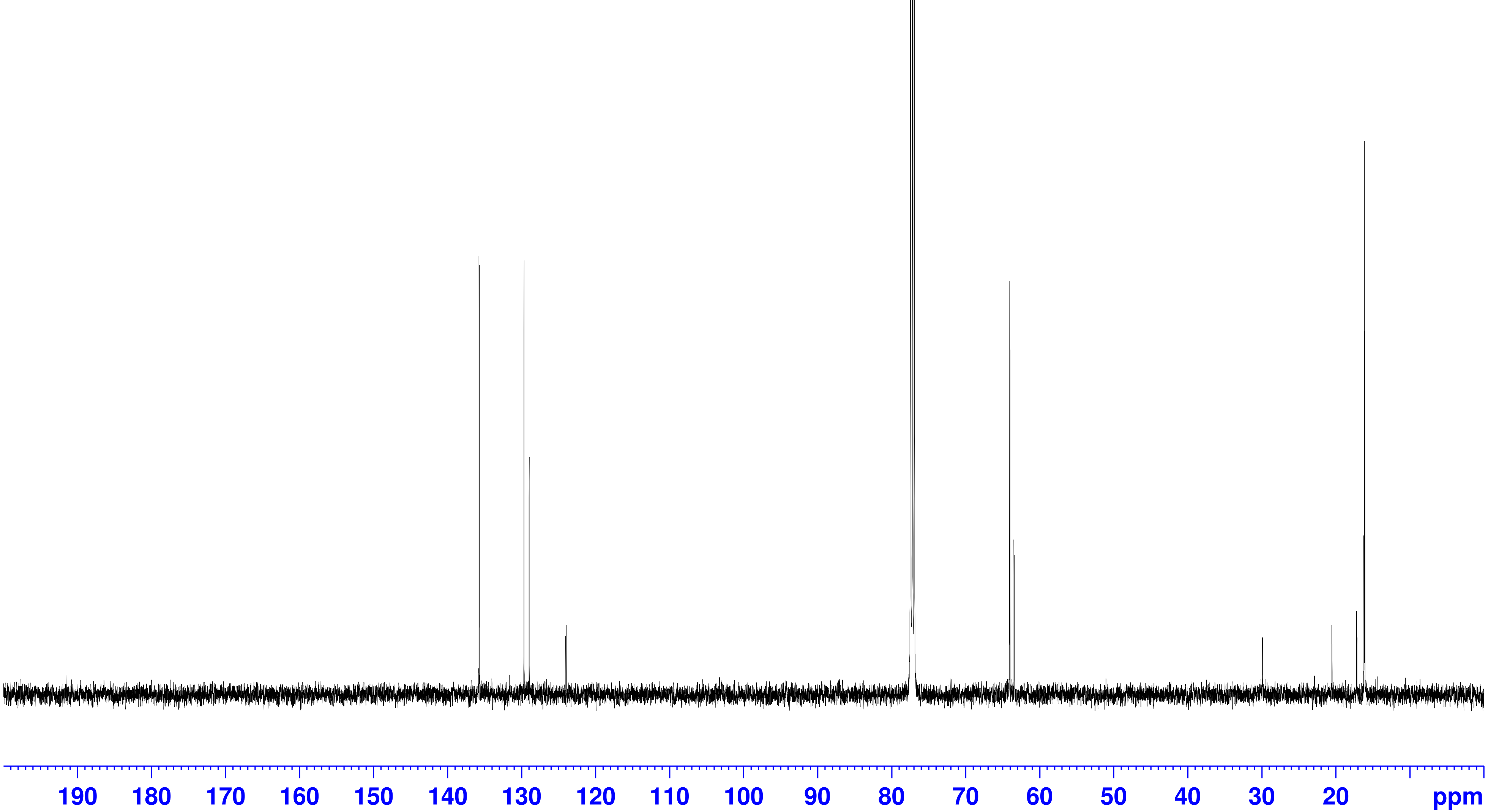

^{31}P NMR, 203 MHz, $CDCl_3$

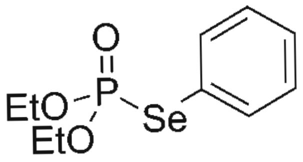

5b

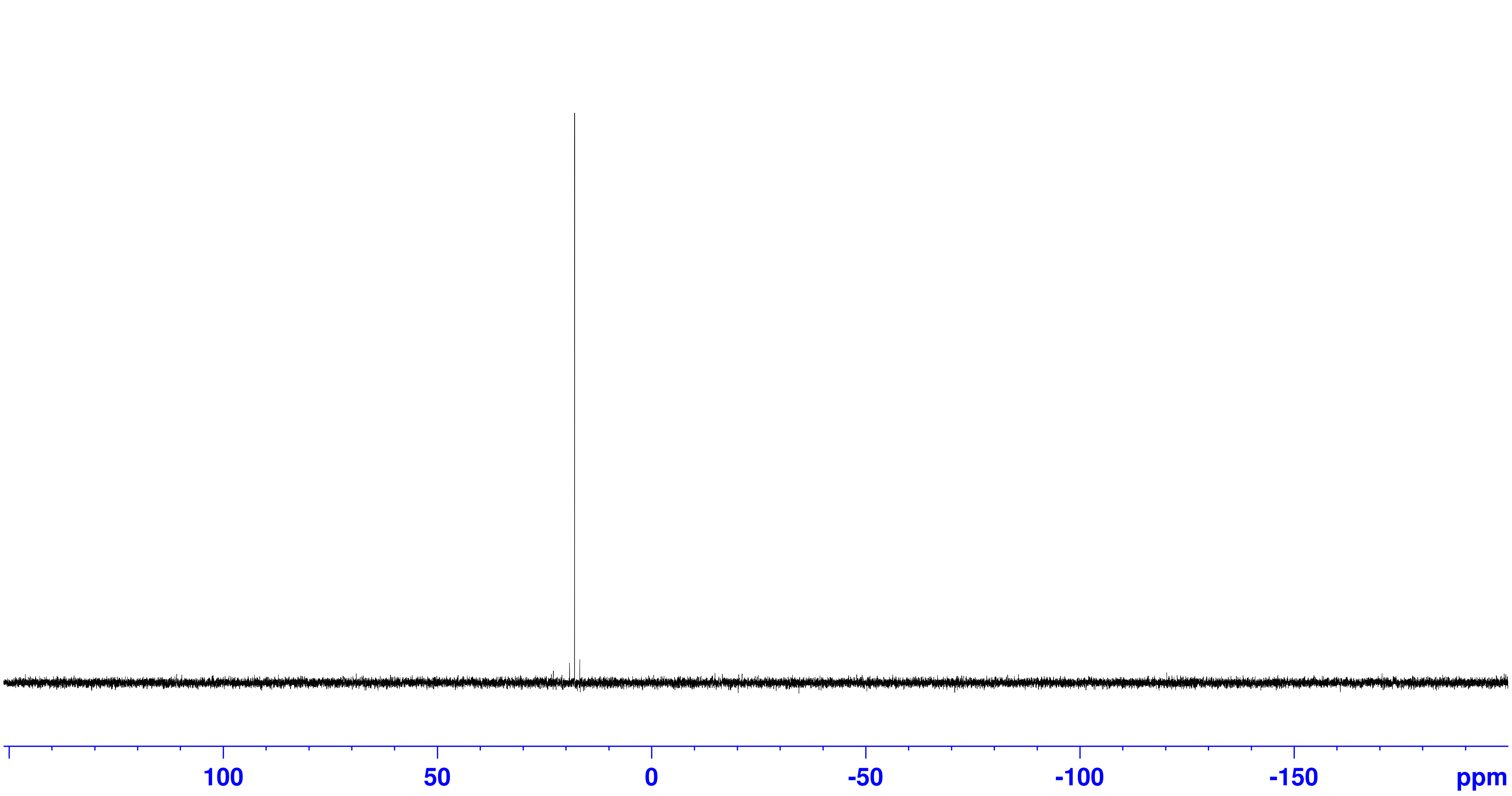

^{1}H NMR, 500 MHz, $CDCl_3$

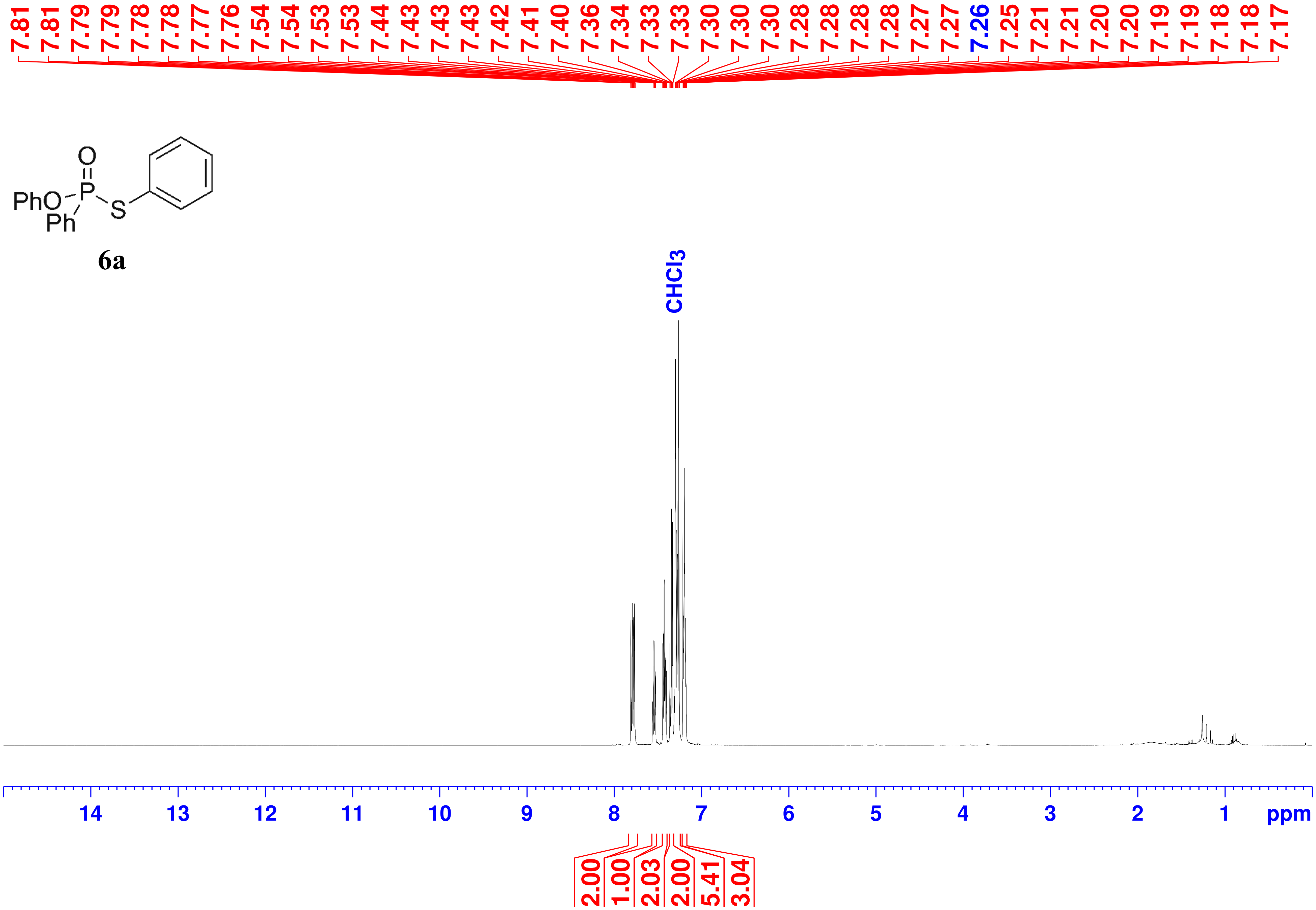

^{13}C NMR, 126 MHz, $CDCl_3$

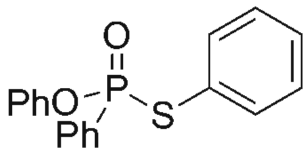

6a

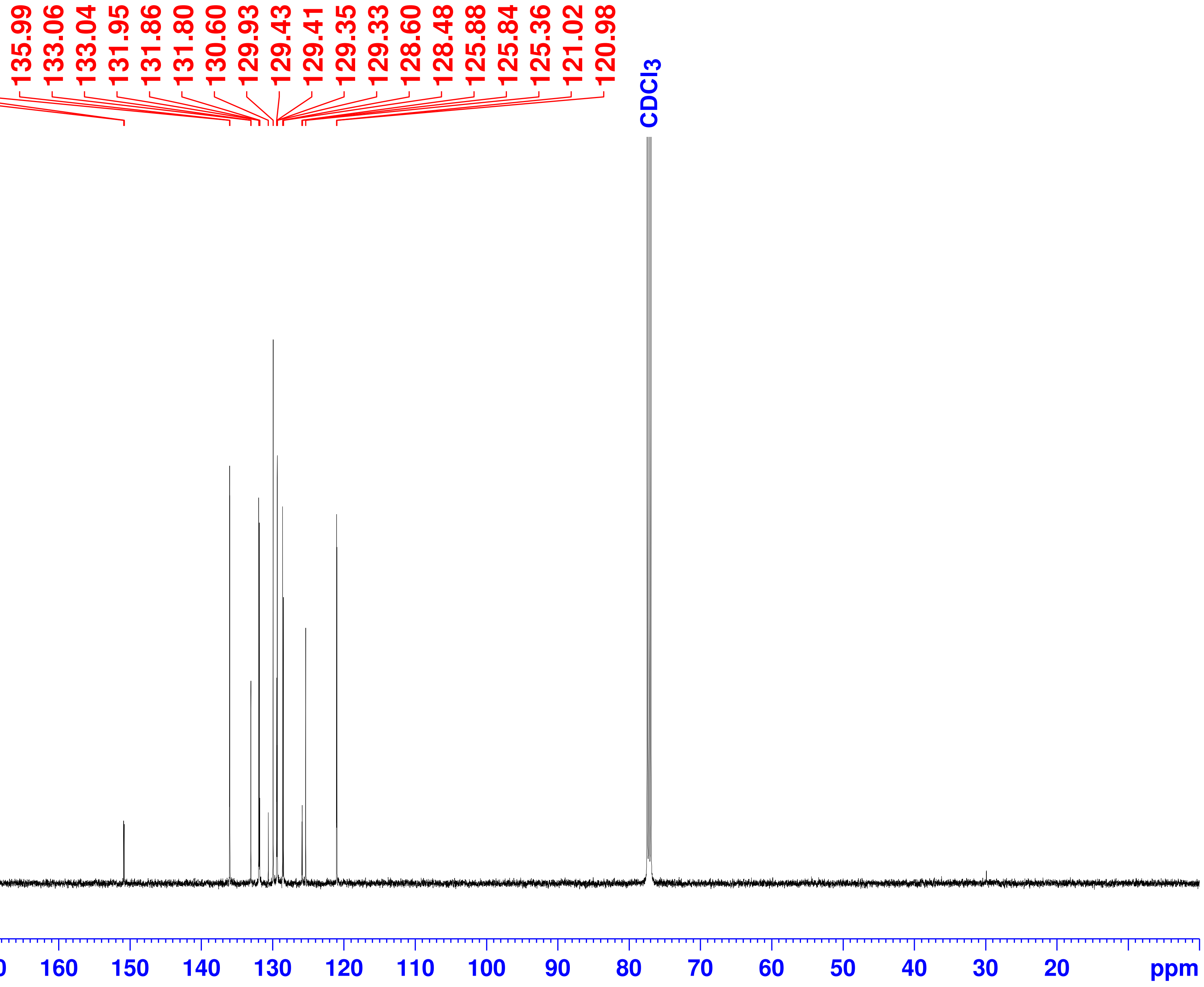

^{31}P NMR, 203 MHz, $CDCl_3$

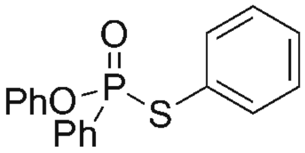

6a

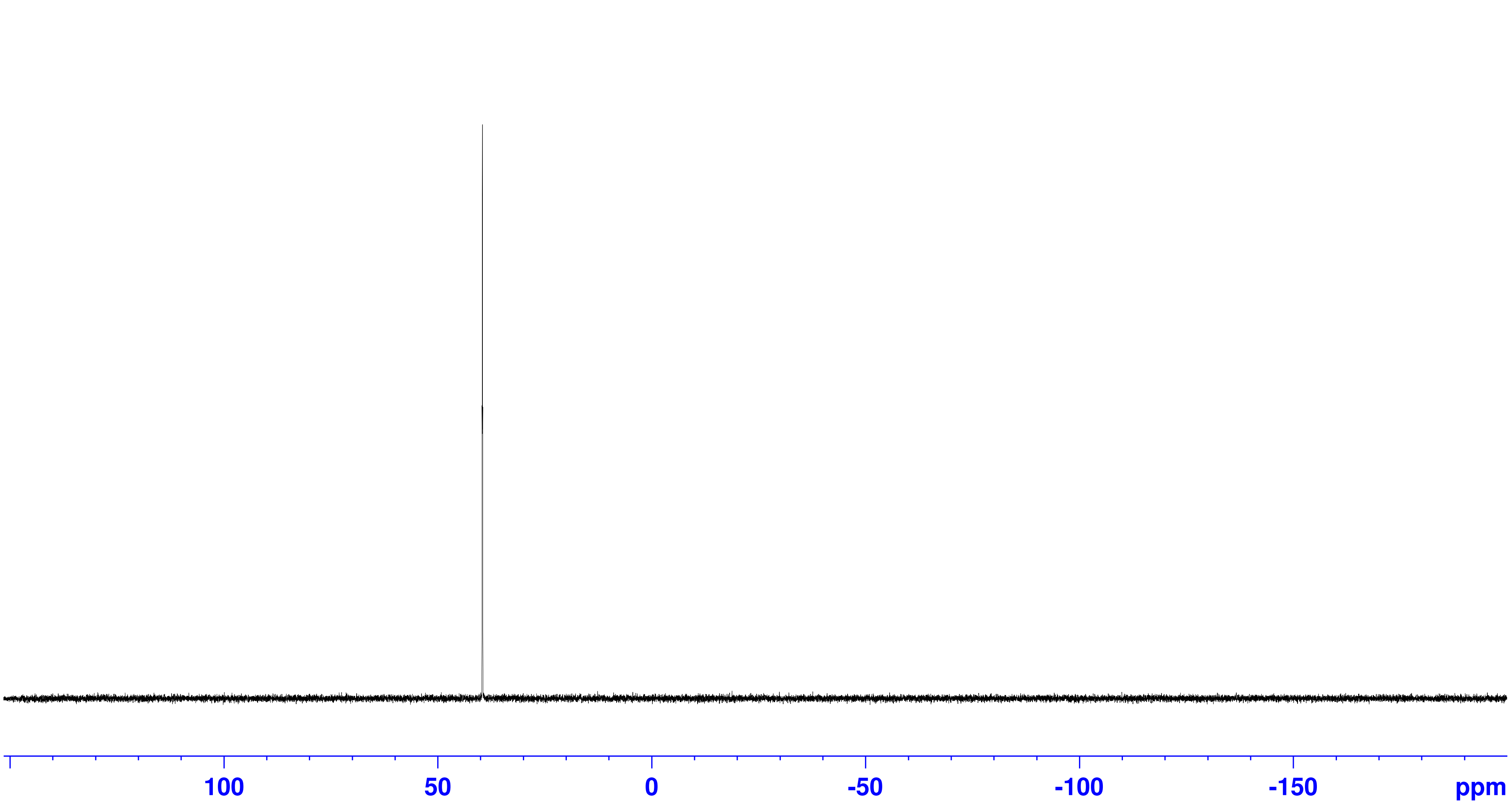

^{1}H NMR, 500 MHz, $CDCl_3$

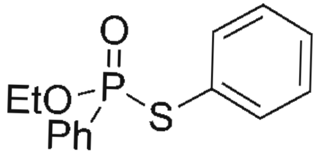

6b

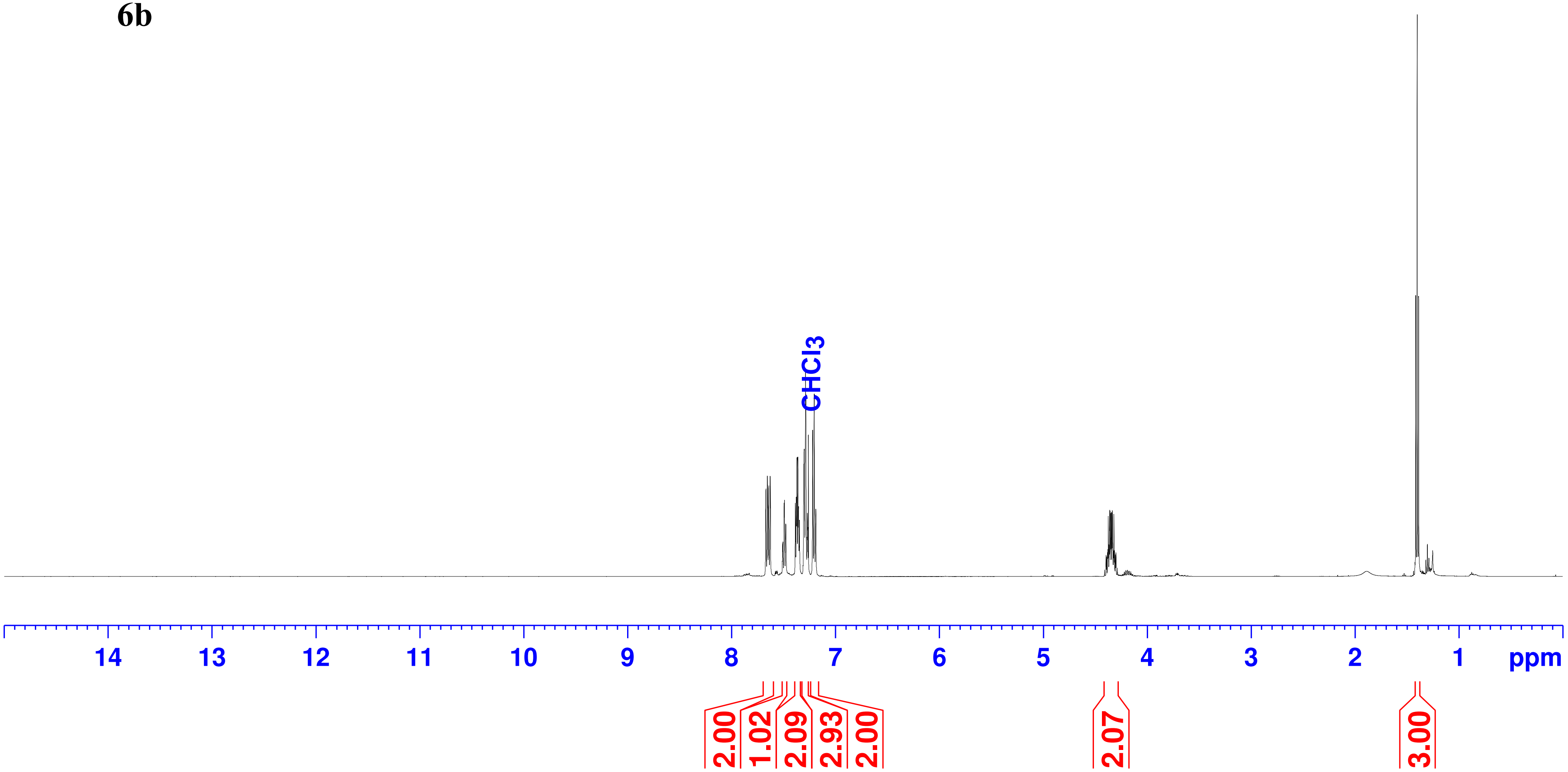

^{13}C NMR, 126 MHz, $CDCl_3$

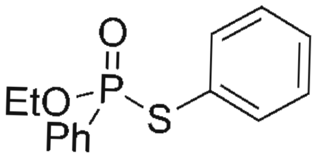

6b

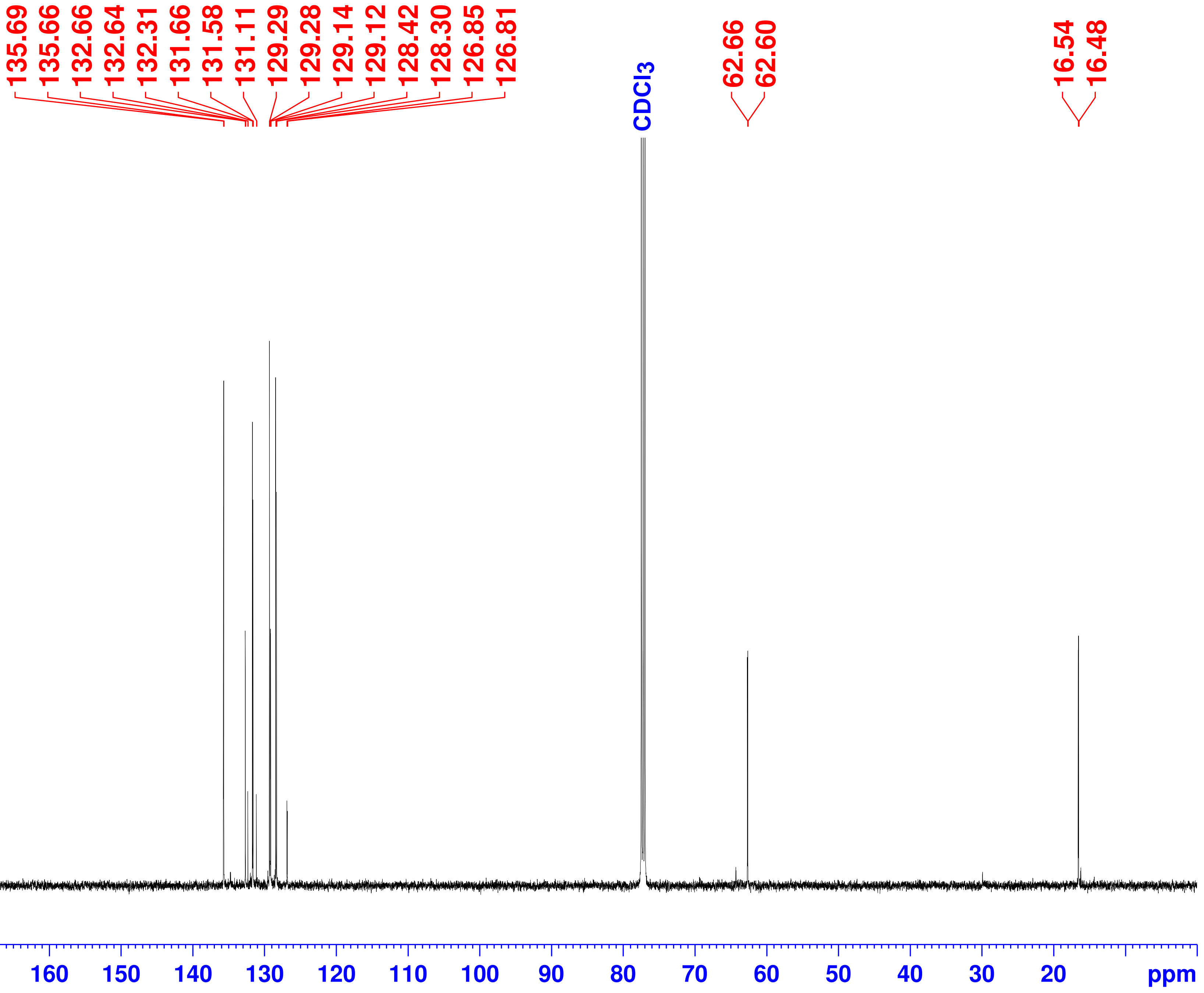

^{31}P NMR, 203 MHz, $CDCl_3$

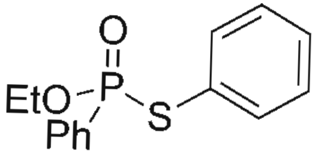

6b

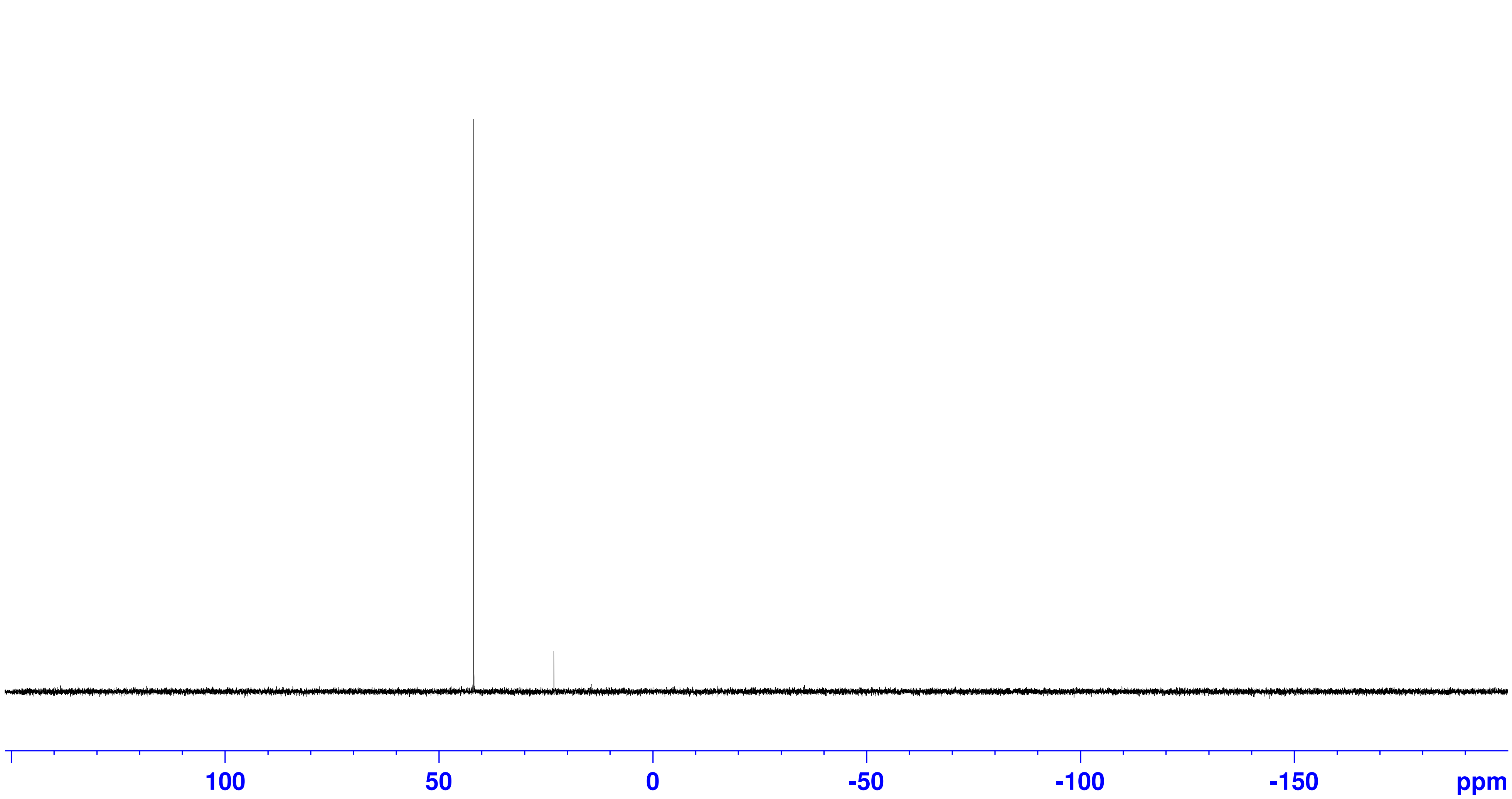

^1H NMR, 500 MHz, $CDCl_3$

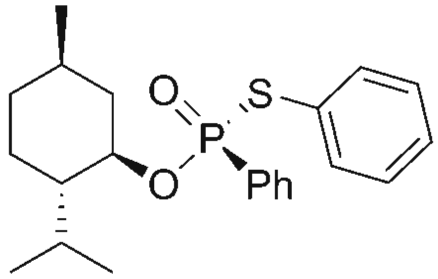

6c

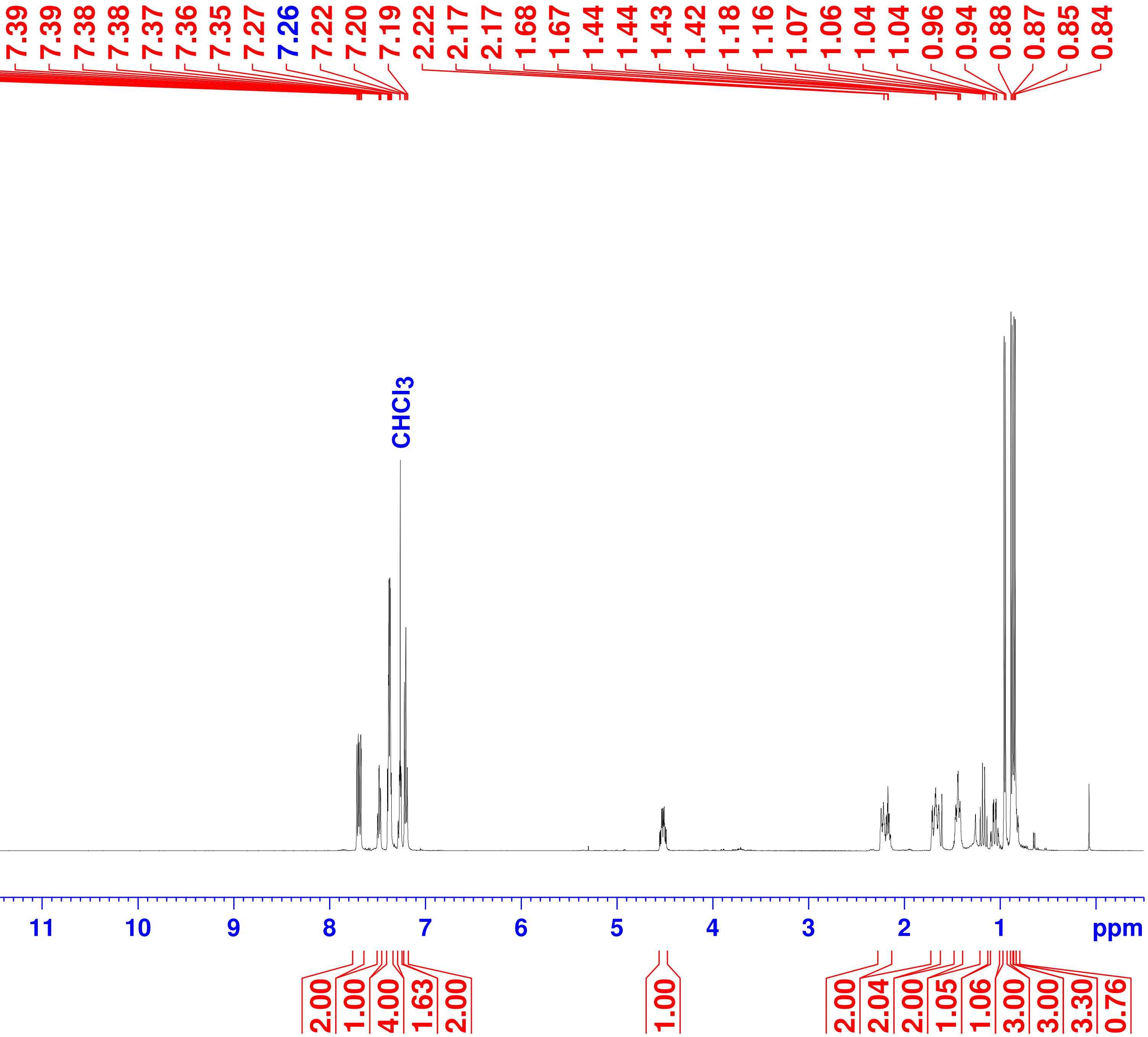

^{13}C NMR, 126 MHz, $CDCl_3$

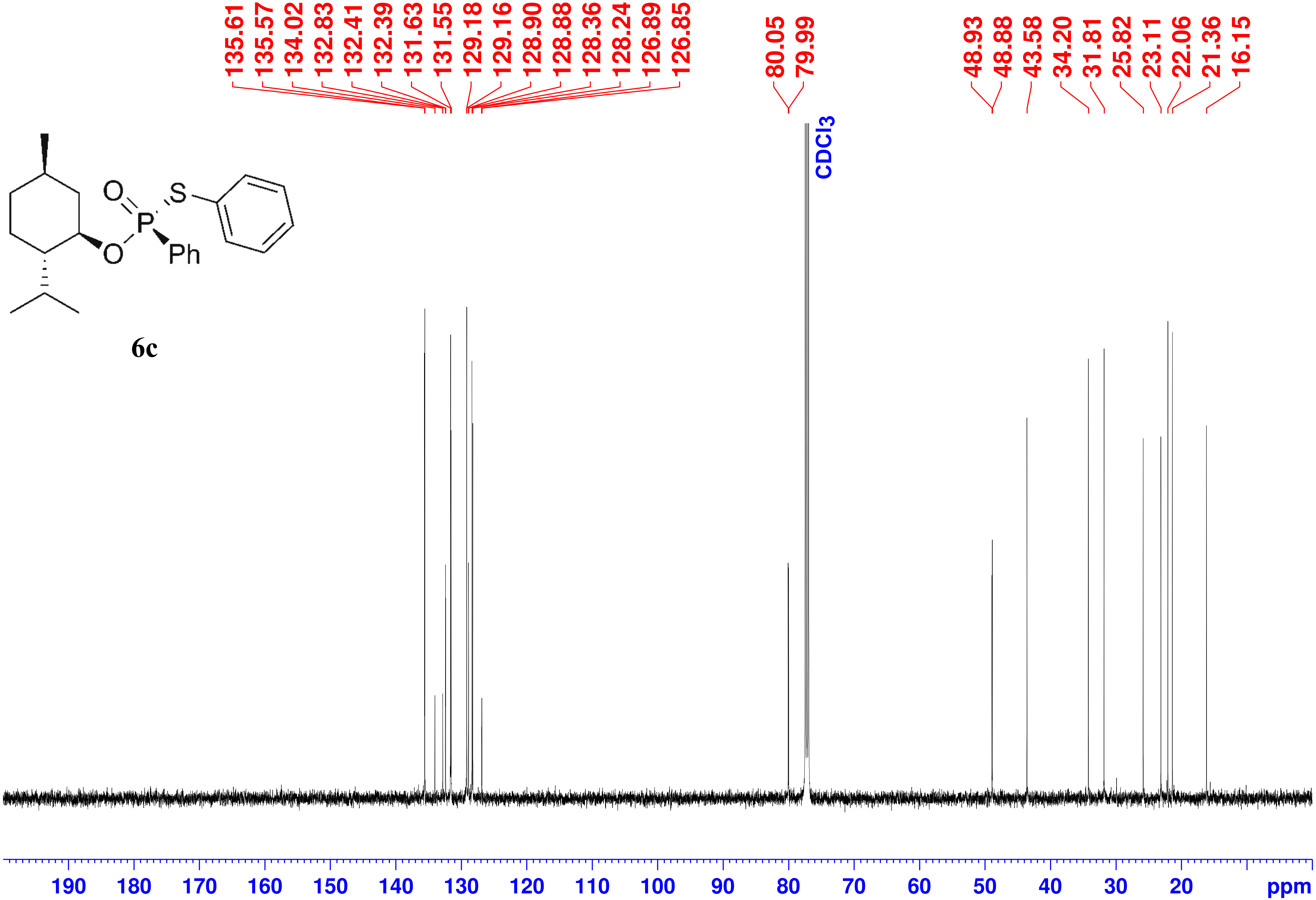

^{31}P NMR, 203 MHz, $CDCl_3$

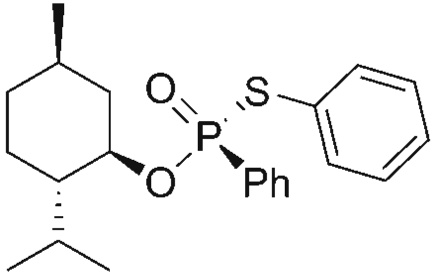

6c

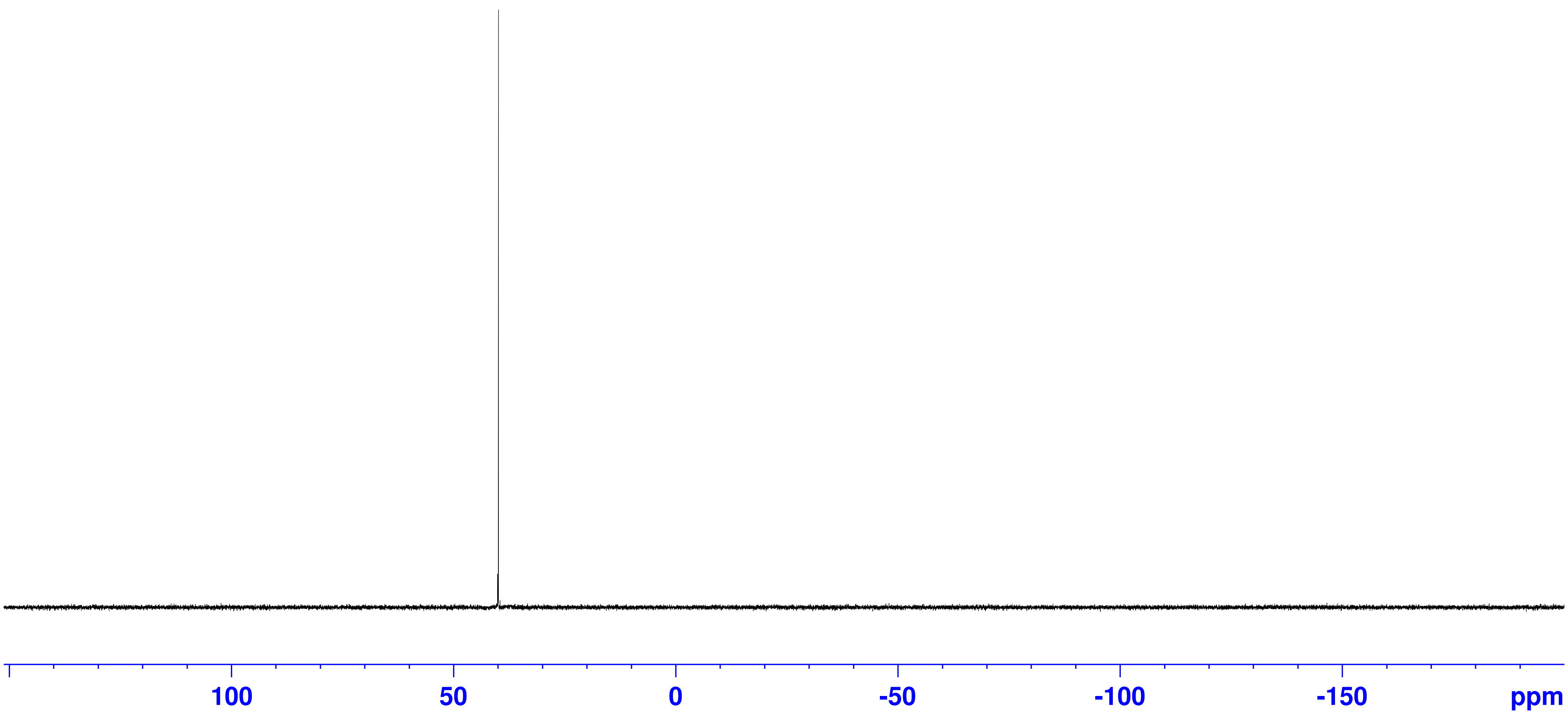

^1H NMR, 500 MHz, $CDCl_3$

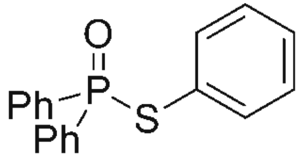

7a

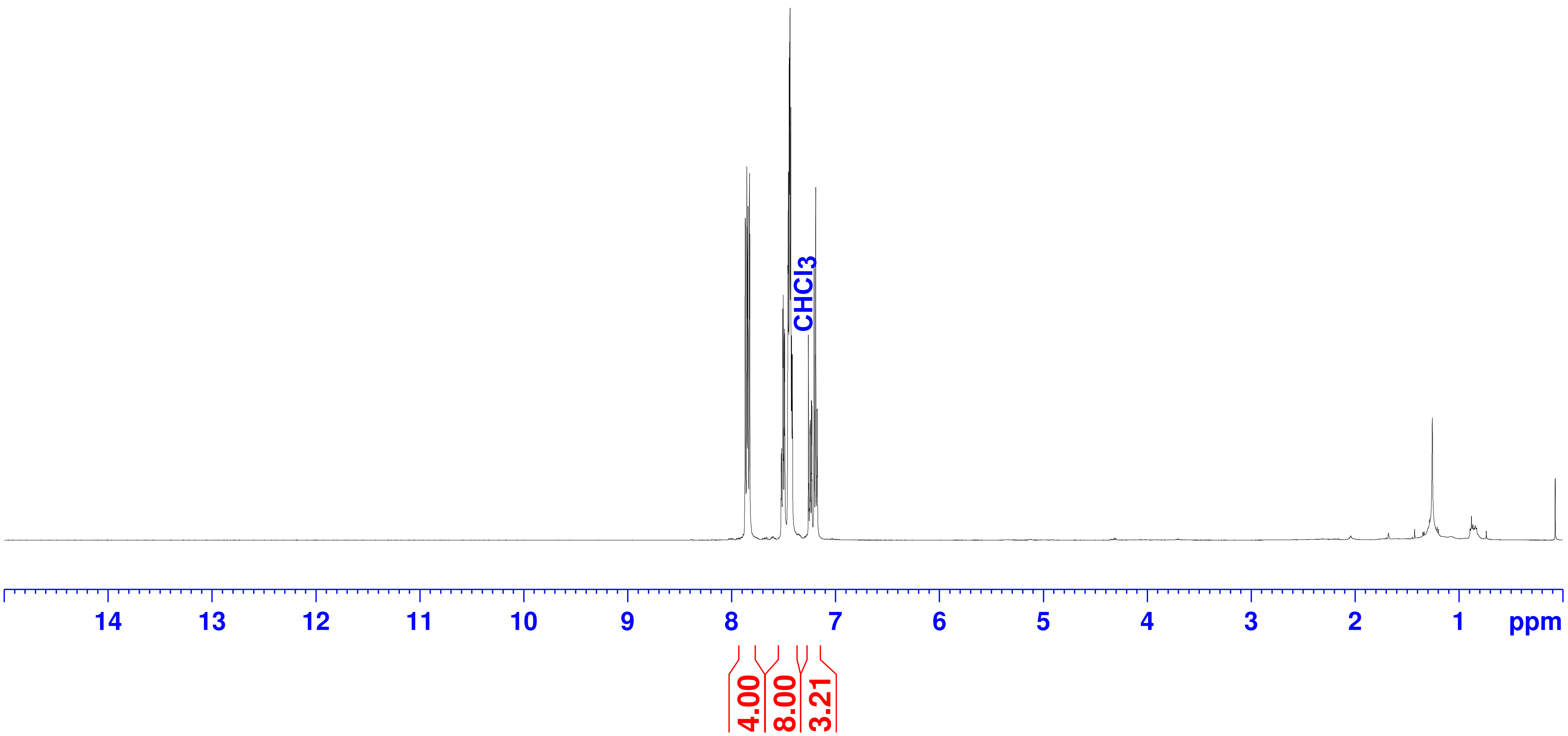

^{13}C NMR, 126 MHz, $CDCl_3$

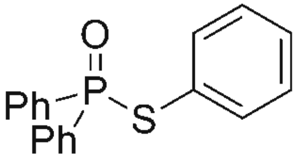

7a

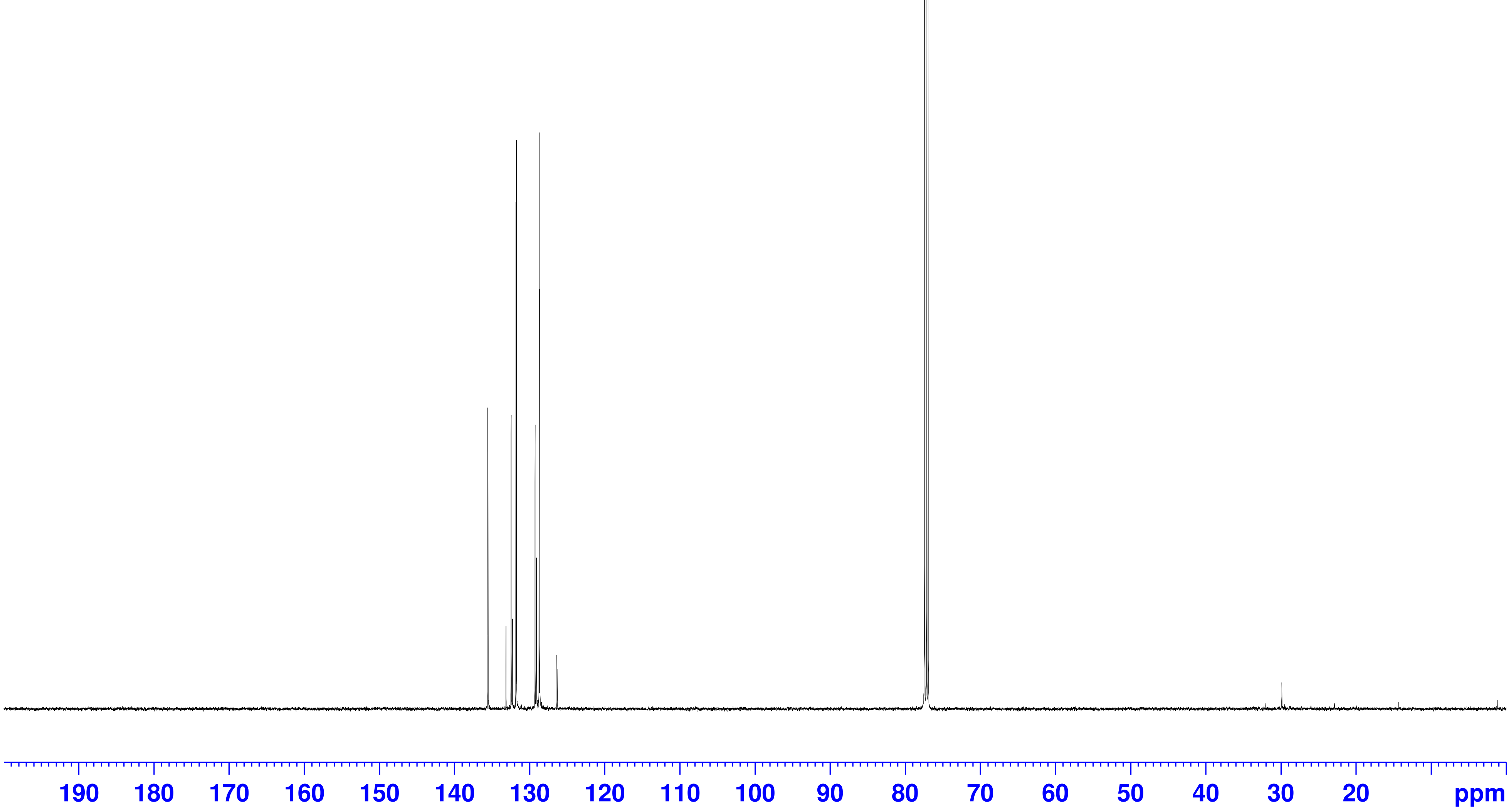

^{31}P NMR, 203 MHz, $CDCl_3$

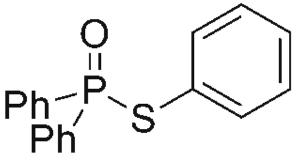

7a

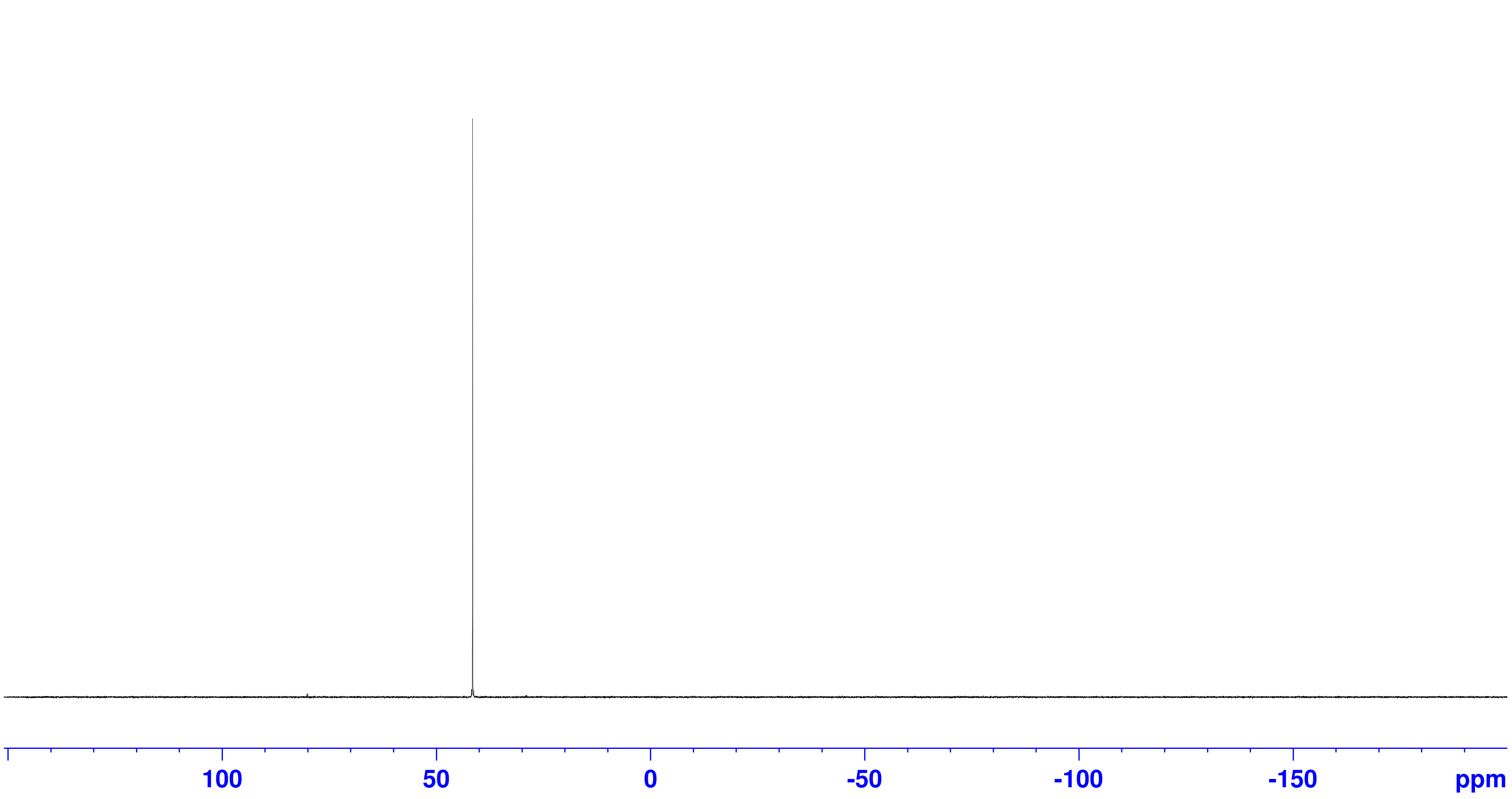

Supplement: Supplementary file 1 — ol2c04310_si_001.pdf [file ol2c04310_si_001.pdf]
